# Oxidative N‐Heterocyclic Carbene Catalysis

**DOI:** 10.1002/chem.202202467

**Published:** 2022-11-22

**Authors:** Carmela De Risi, Arianna Brandolese, Graziano Di Carmine, Daniele Ragno, Alessandro Massi, Olga Bortolini

**Affiliations:** ^1^ Dipartimento di Scienze Chimiche, Farmaceutiche ed Agrarie Università di Ferrara Via L. Borsari, 46 44121 Ferrara Italy; ^2^ Dipartimento di Scienze dell'Ambiente e della Prevenzione Università di Ferrara Via L. Borsari, 46 44121 Ferrara Italy

**Keywords:** domino reactions, N-heterocyclic carbenes, organocatalysis, oxidation, synthetic methods

## Abstract

N‐Heterocyclic carbene (NHC) catalysis is a by now consolidated organocatalytic platform for a number of synthetic (asymmetric) transformations via diverse reaction modes/intermediates. In addition to the typical *umpolung* processes involving acyl anion/homoenolate equivalent species, implementation of protocols under oxidative conditions greatly expands the possibilities of this methodology. Oxidative NHC‐catalysis allows for oxidative and oxygenative transformations through specific manipulations of Breslow‐type species depending upon the oxidant used (external oxidant or O_2_/air), the derived NHC‐bound intermediates paving the way to non‐*umpolung* processes through activation of carbon atoms and heteroatoms. This review is intended to update the state of the art in oxidative NHC‐catalyzed reactions that appeared in the literature from 2014 to present, with a strong focus to crucial intermediates and their mechanistic implications.

## Graphical abstract

**The advances in oxidative N‐heterocyclic carbene (NHC)‐catalysis** are reviewed according to the strategies that rely on typical NHC‐bound intermediates, including acyl/imidoyl azolium, α,β‐unsaturated acyl azolium, and azolium peroxide species, as well as NHC‐derived *ortho*‐quinone methide (*o*‐QM), aza‐fulvene, and triaza‐diene intermediates, with particular focus on mechanistic aspects.

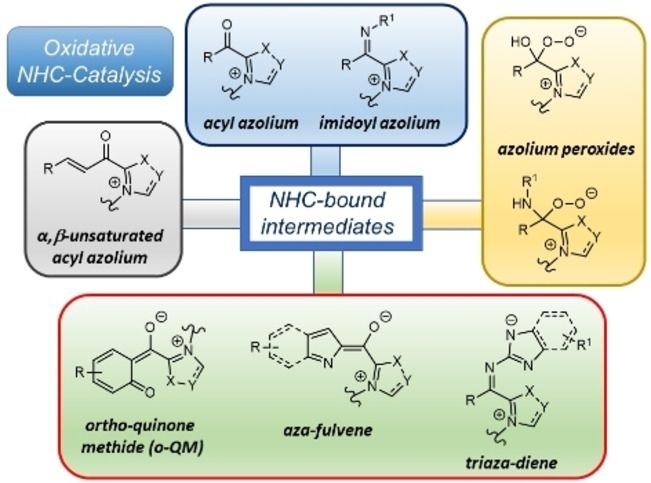

## 1 Introduction

In the realm of organocatalysis, the huge versatility of N‐heterocyclic carbenes (NHCs) as catalysts for new activations and synthetic transformations, asymmetric too, has been amply demonstrated.^[1]^

Mechanistically, diverse reaction modes have been postulated for NHC‐catalyzed processes, which involve typical reactive intermediates to activate carbon atoms and heteroatoms.

**Figure 1:**
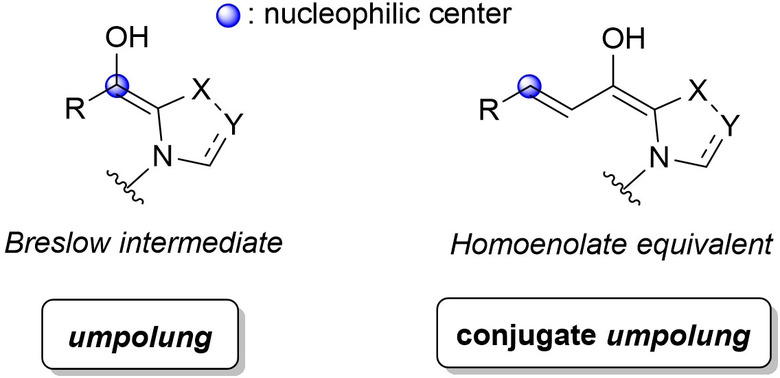
Τypical *umpolung* reactivity of NHCs.

In that respect, classical *umpolung* or conjugate *umpolung* are widely recognized as suitable means of activating carbon atoms as nucleophiles via Breslow intermediates (acyl anion equivalents)^[2]^ and homoenolate equivalent species^[3]^ (Figure 1).

Apart from this, the scope of NHCs chemistry may be further expanded with protocols under oxidative conditions relying on the cooperation of an oxidizing agent, including external (stoichiometric) inorganic/organic oxidants and O_2_ (air).

**Scheme 1:**
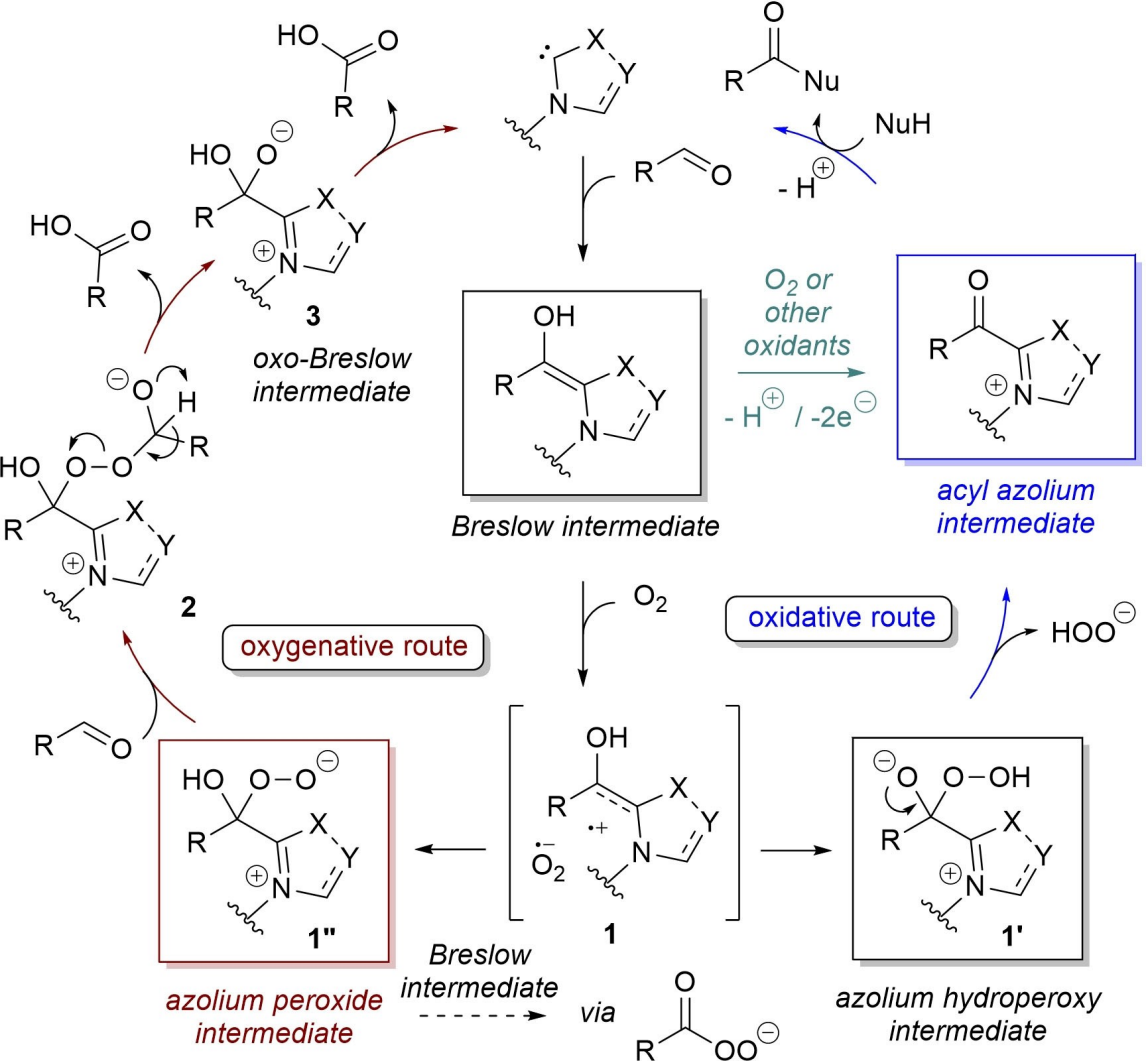
Possible mechanisms for oxidative NHC‐catalysis.

Common pivotal step to these transformations is the oxidation of a Breslow intermediate, which can occur through two possible routes, that is, formation of an acyl azolium ion (electrophilic acylium cation synthon)^[4]^ via a two‐electron transfer to the oxidant species (O_2_ or other oxidants) and/or oxygen atom transfer from the oxidant (O_2_) (Scheme 1). In the latter case, a single‐electron‐transfer (SET) process is believed to take place,^[5]^ with generation of complex **1** between Breslow‐derived radical cation and superoxide radical anion: these recombine to afford two tautomeric peroxo Breslow intermediates **1’** and **1”**, that is to say hydroperoxy/peroxide anions, with switched reactivity.[^6^, ^7^] The one (**1’**) liberates the hydroperoxy anion and converts into an acyl azolium ion, which is prone to nucleophilic substitution at the carbonyl group (acyl group transfer) with regeneration of the NHC catalyst (*oxidative route*, Scheme 1, blue path); the other (**1”**) reacts with a second aldehyde molecule followed by rearrangement of the adduct **2** formed giving rise to a carboxylic acid and a oxo‐Breslow species **3**, with the organocatalytic cycle completed by reformation of free NHC and release of a second molecule of acid (*oxygenative route*, Scheme 1, red brick path). A secondary oxygenative lane implying reaction of **1”** with Breslow intermediate may not be totally excluded, notwithstanding the expected peracid has not been detected in detailed MS experiments.^[6]^

**Scheme 2:**
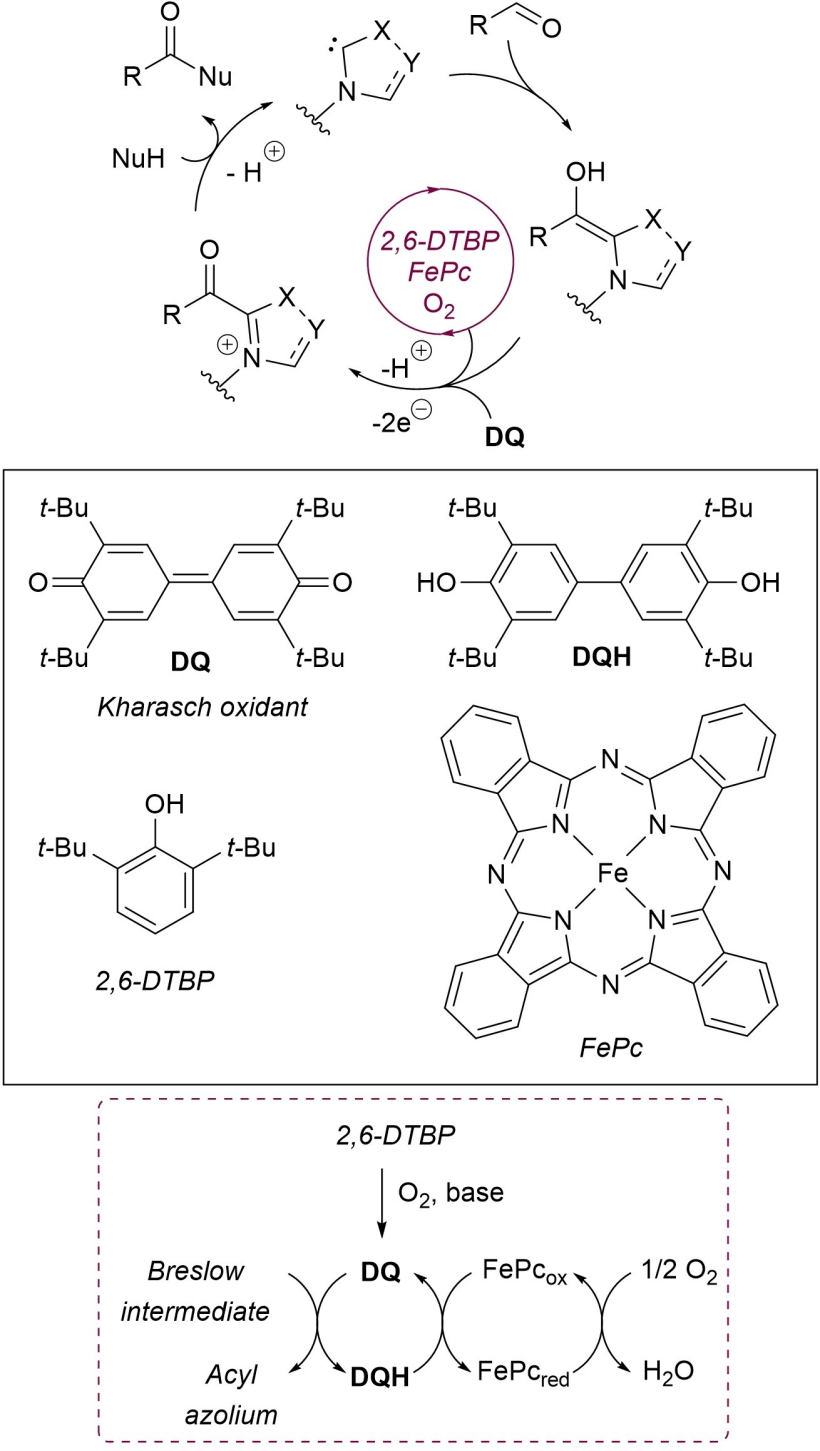
Aerobic oxidative NHC‐catalysis based on ETMs.

From the perspective of the oxidative pathway (Breslow intermediate‐to‐acyl azolium compound), the most often used oxidant is 3,3’5,5’‐tetra‐*tert*‐butyldiphenoquinone (Kharasch reagent, **DQ**, Scheme 2),^[8]^ but many other oxidants have been shown to be feasible, such as MnO_2_, TEMPO, riboflavin, phenazine, and azobenzene, to name as a few. However, the need to be employed in stoichiometric amounts (even greater) has severe issues, especially those relating to scalability (high *E*‐factor), sustainability (separation and disposal of waste) and economic impact (price).

Non‐toxic and inexpensive O_2_, being more beneficial for atom‐efficiency and eco‐friendliness (water as the only by‐product), is an ideal substitute for high‐molecular weight oxidants (particularly **DQ)**, possibly combined with a biomimetic system of electron‐transfer mediators (ETMs) to circumvent the high‐energy barriers required for the direct catalytic oxidation of Breslow intermediate with pure O_2_ (air).^[9]^ Under all such instances, a low‐energy path flowing electrons from the substrate to oxygen is realized through a series of catalytic cycles typically involving **DQ**/iron phthalocyanine (FePc) couple (Scheme 2). To be precise, the electron‐transfer process between Breslow intermediate and **DQ** leads to the formation of the acyl azolium ion and the reduced diol **DQH**: the former is involved in the nucleophilic acyl transfer event and the latter is re‐oxidized by FePc and air (O_2_) as the terminal oxidant. Noteworthily, catalytic 2,6‐di‐*tert*‐butylphenol (2,6‐DTBP) could be introduced as precursor of **DQ** (in situ oxidation).^[8]^

**Scheme 3:**
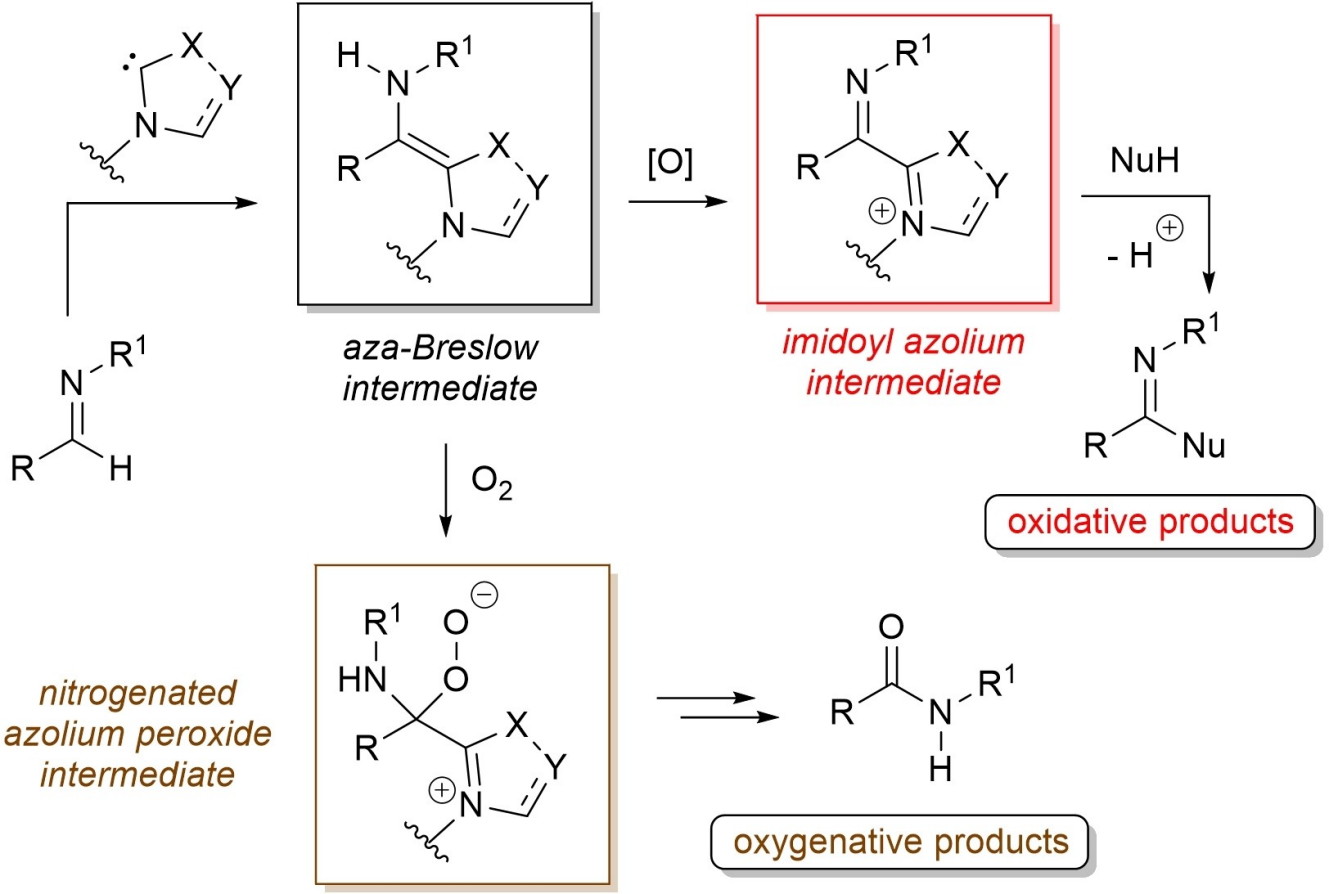
Simplified mechanisms for oxidative NHC‐catalysis starting from aldimines.

It is now well known that similar considerations may apply to aldimines, NHC addition generating nitrogen analogues of the Breslow species, known as *aza*‐Breslow intermediates (Scheme 3).^[10]^ Starting from these, analogous electrophilic imidoyl azoliums (two‐electron oxidation) and nitrogenated azolium peroxidic species (oxygen atom transfer) can be derived as critical intermediates of oxidative‐ and oxygenative‐type processes.

**Scheme 4:**
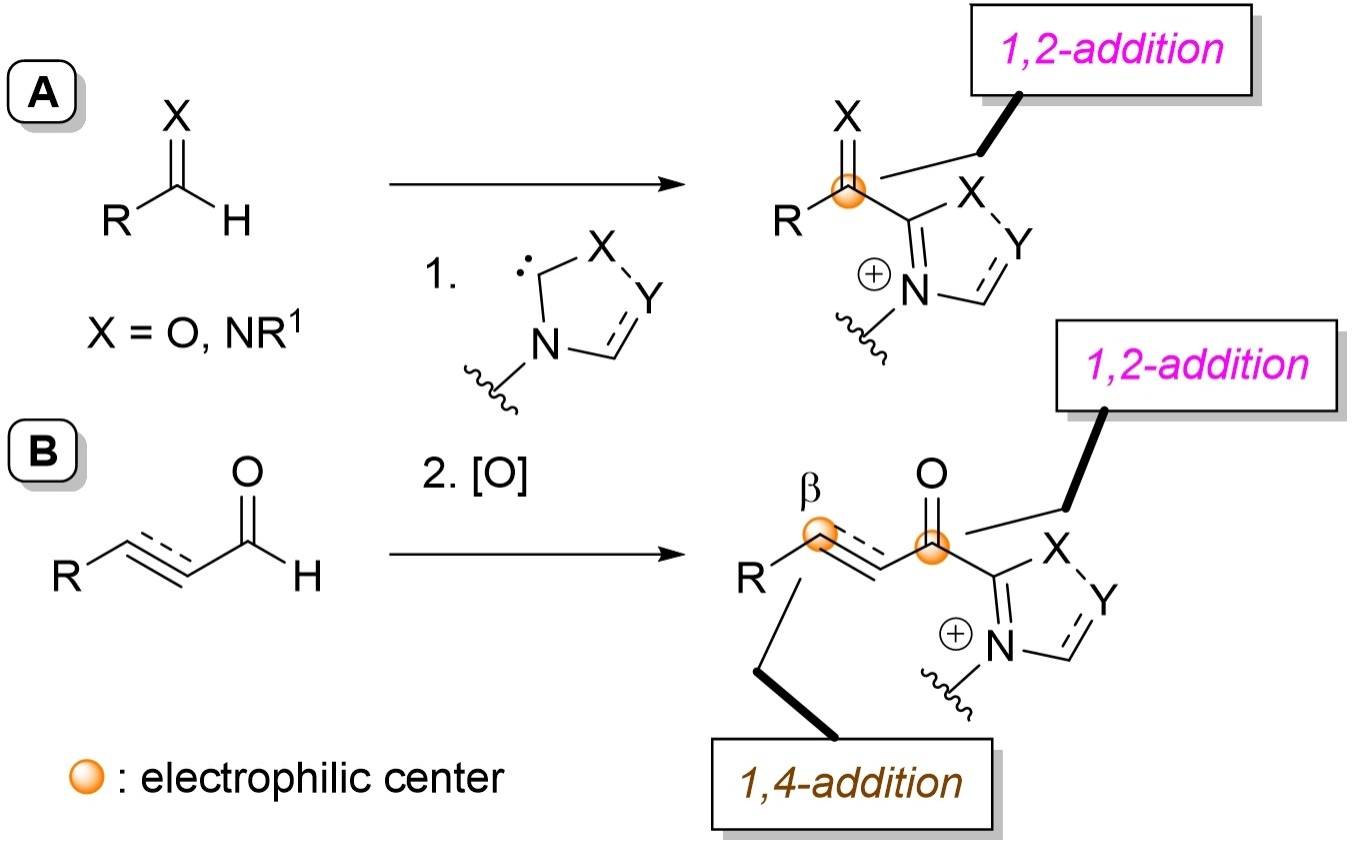
Possible plays for simple acyl/imidoyl azoliums (**A**) and α,β‐unsaturated (alkynyl) acyl azoliums (**B**).

In the aforesaid cases, the fate of the pivoting intermediates in the oxidative/oxygenative transformations fits a validated pattern. This means that acyl/imidoyl azolium ions are expected to go through nucleophilic addition to the electrophilic C=O or C=N groups (*1,2‐addition reactivity*, Scheme 4A); nonetheless, not all of the acyl azolium ions necessarily proceed in this way. Indeed, different reaction paths can take place in the case of α,β‐unsaturated (alkynyl) acyl azolium, given its nature of biselectrophile (Scheme 4B). Here, the most common mechanistic play involves cascade (domino) processes initiated by 1,4‐addition, but also 1,2‐addition can be the starting step. In addition, exclusive 1,2‐addition reactivity towards the carbonyl group is viable.

**Scheme 5:**
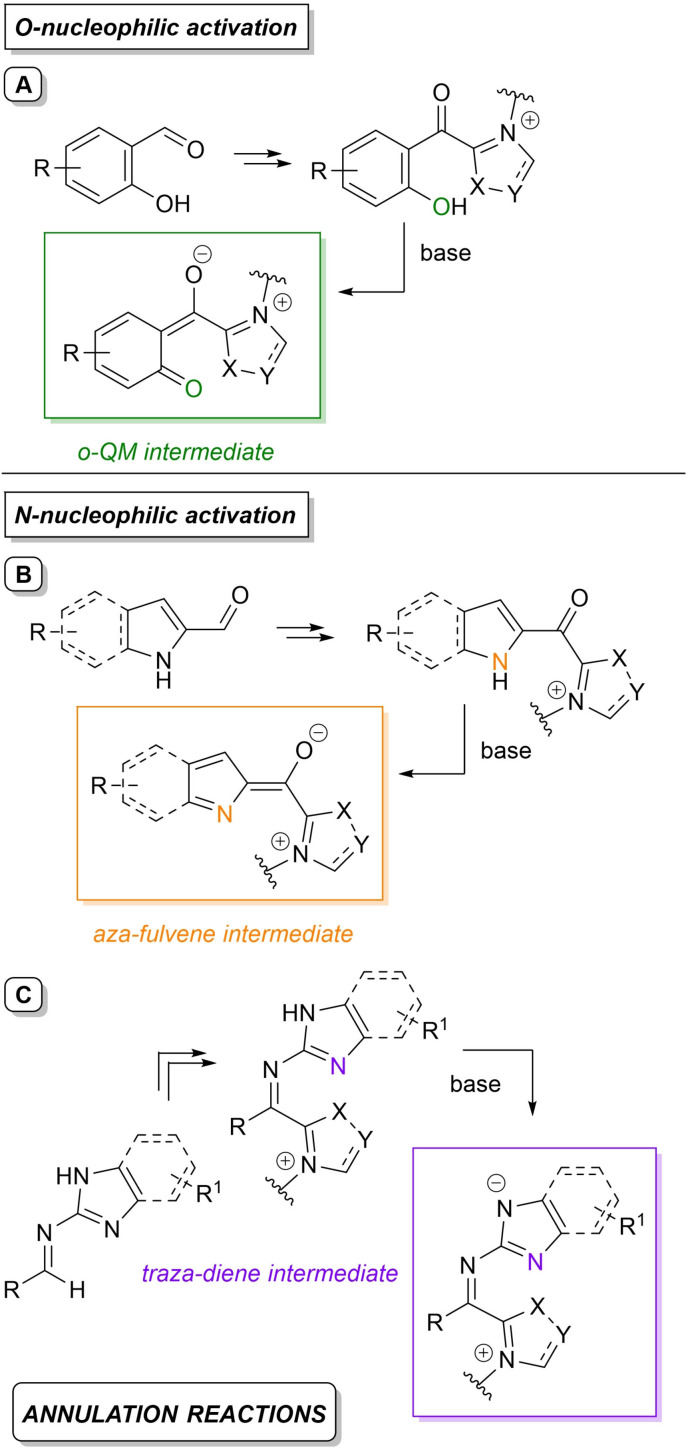
*O*‐ and *N*‐nucleophilic activation by oxidative NHC‐catalysis.

Alongside the most common activation of (sp^2^/sp) carbon atoms, a mode of NHC‐catalyzed heteroatom activation under oxidative conditions can be enacted via acyl azolium‐derived *ortho*‐quinone methide (*o*‐QM) (Scheme 5A) and aza‐fulvene type intermediates (Scheme 5B), as well as from imidoyl azolium‐derived triaza‐diene species (Scheme 5C), with all three reactive dipoles eventually applied in annulation reactions.

**Scheme 6:**
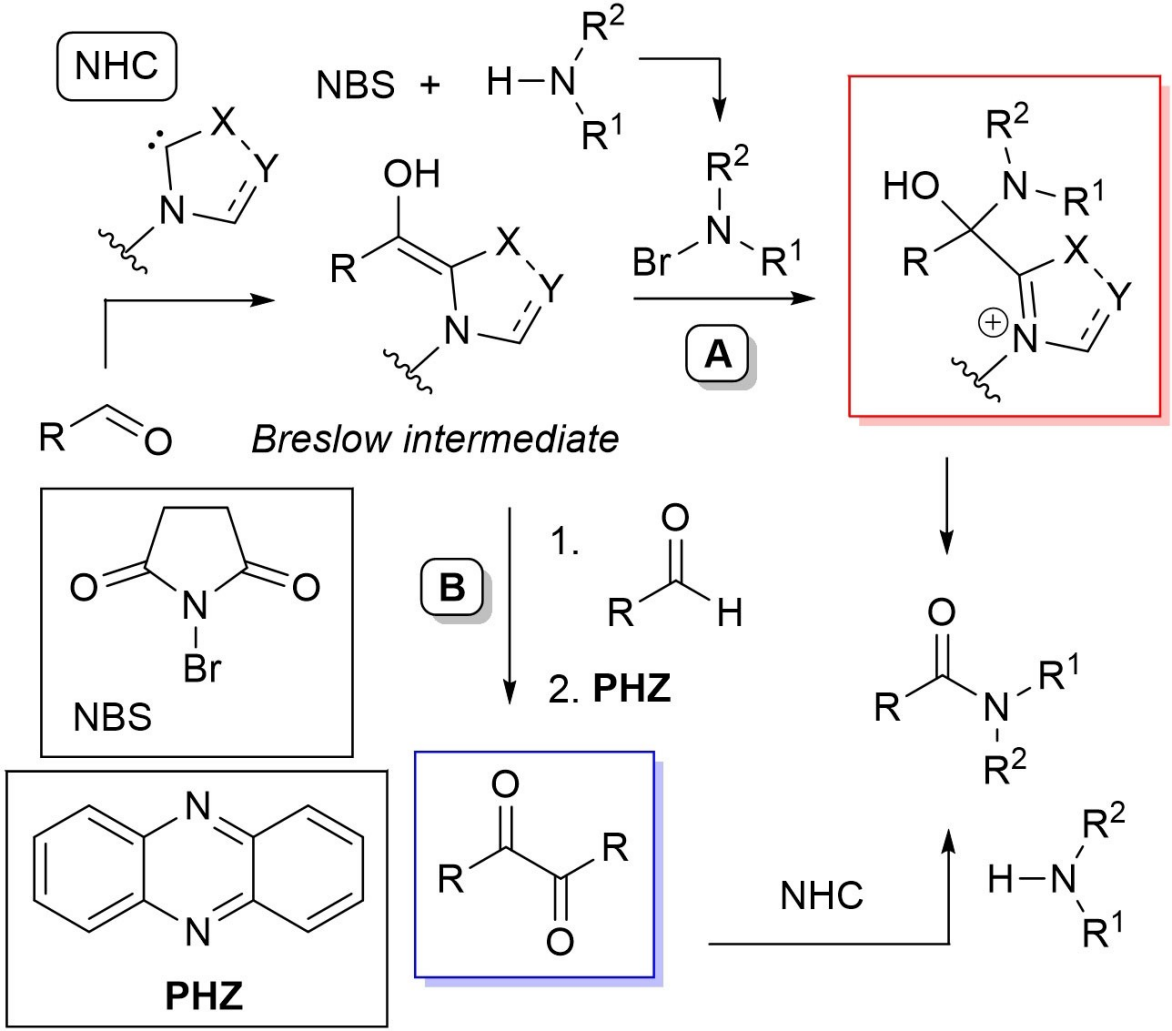
Unconventional reaction intermediates in NHC‐catalyzed processes promoted by external oxidants.

In some cases the acyl azolium species is completely ruled out, and unconventional reaction intermediates/mechanisms have been demonstrated. If one speaks of external oxidants, the NHC‐catalyzed oxidative amidation of aldehydes with amines involves reaction of Breslow intermediate with a transient *N*‐bromoamine (*N*‐electrophile) when using *N*‐bromosuccinimide (NBS) (Scheme 6, *route A*), or action of in situ formed benzyls (still derived from Breslow intermediate) as acylating agents if phenazine (**PHZ**) is used as an oxidant (Scheme 6, *route B*).

**Scheme 7:**
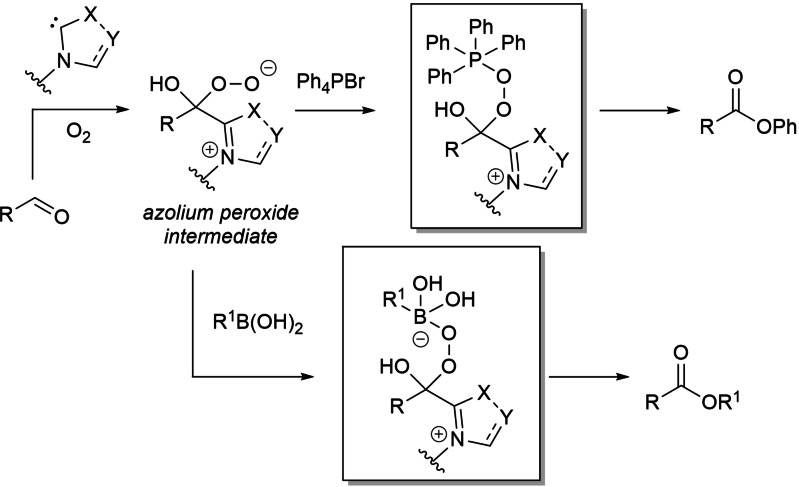
Atypical NHC‐catalyzed aerobic oxidative esterifications.

In the field of aerobic oxidative NHC‐catalysis, on the other hand, aldehyde‐to‐ester conversion was enabled by the introduction of tetraphenylphosphonium bromide and arylboronic acids as uncommon counterparts of the azolium peroxidic intermediate (Scheme 7).

**Scheme 8:**
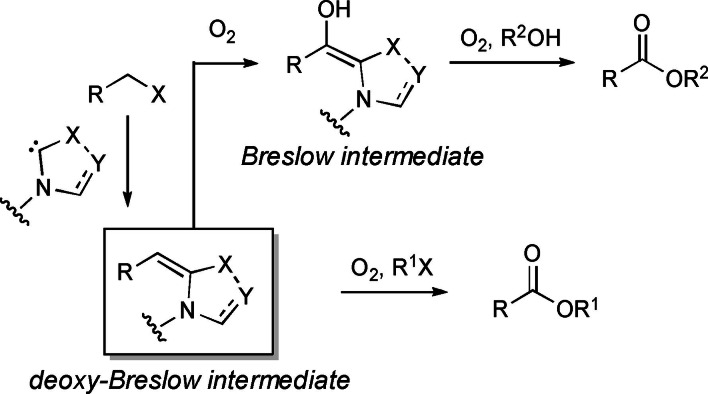
NHC‐catalyzed oxidative ester synthesis from organic halides.

Besides, a novel esterification strategy was developed through incorporation of oxygen atoms into organic halides via a pivotal deoxy Breslow intermediate (Scheme 8). Interestingly, the latter could be taken to a Breslow intermediate for cross‐esterification of organic halides with alcohols.

The value and versatility of oxidative NHC‐catalysis were for the first time reviewed in 2012 by von Wangelin and co‐workers,^[11]^ and one year later a Concept article by Studer group comprehensively illustrated the potential of oxidative carbene catalysis in synthesis.^[12]^ Since then, there has been an ever‐growing interest in oxidative NHC‐catalysis, and a selection of applications has been recently reviewed by Rafiński and Dzieszkowski.^[13]^ In addition, Maheswari and co‐workers focused their attention on representative examples in the field of NHC‐catalyzed oxidative cyclization,^[14]^ while Sundén and co‐workers reviewed their own efforts in the field of aerobic oxidative NHC‐catalysis.^[15]^

However, in our view, advances in oxidative NHC‐catalysis do not seem to have been totally covered by these last three papers, so we aim to give a new contribution to review the literature produced in this flourishing area (from 2014 to date). And in so doing, we have sought to avoid duplication as much as possible with existing reviews. Because of this, we have opted out of covering oxidative γ‐ and δ‐carbon activation of unsaturated aldehydes, as these transformations are broadly discussed in very recent literature overviews.^[16]^

The works being discussed are arranged according to which of the NHC‐bound intermediates plays as the actor, so Section 2 is dedicated to the strategies built around acyl/imidoyl azolium intermediates, with a sub‐classification inserted depending on the type of bond which is assembled in the acyl transfer (*1,2‐addition*) step. Cascade transformations of α,β‐unsaturated acyl azolium intermediates (including alkynyl acyl azoliums) are particularized in Section 3, with sub‐chapters focusing on the sequence of contributing events (bonds formation), while Section 4 deals with the NHC‐bound intermediates involved in remote *O*‐ and *N*‐nucleophilic activation. And finally, Section 5 covers the approaches for which other sorts of situations are implicated, including azolium peroxidic intermediates and unconventional reaction species/mechanisms.

For the sake of clarity, some principles have been pursued, as far as possible, in discussing the papers in each section (sub‐section), for example, substrate category, type of oxidant source fielded for the formation of every single crucial intermediate, and possibly chronological order, with particular emphasis placed on (stereochemical) mechanistic aspects for those suitable works.

## 2 Acyl/Imidoyl Azolium Intermediates

Nucleophilic addition to the electrophilic carbonyl group of oxidatively generated acyl azolium ions has been largely exploited for C−N and C−O (C−S) bond formation, and a similar argument is valid for imidoyl azolium intermediates. Either way, both external (stoichiometric) oxidants as well as O_2_/air are implicated in the key oxidation stage of the aldehyde/aldimine‐derived Breslow/*aza*‐Breslow intermediates. With particular reference to the external oxidants, mainly **DQ**, but also trichloroacetonitrile (CCl_3_N), phenyliodine (II) diacetate (PhI(OAc)_2,_ PIDA), 2,2,6,6‐tetramethylpiperidinyloxy (TEMPO), and *tert*‐butyl hydroperoxide (TBHP) were used.

### 2.1 C−O (C−S) bond formation

#### 2.1.1 External oxidant‐assisted processes

Intermolecular NHC‐catalyzed aldehyde‐to‐ester conversion has been achieved by diverse research groups, mostly using **DQ** as oxidant, typical substrates being aromatic and α,β‐unsaturated aldehydes.

**Scheme 9:**
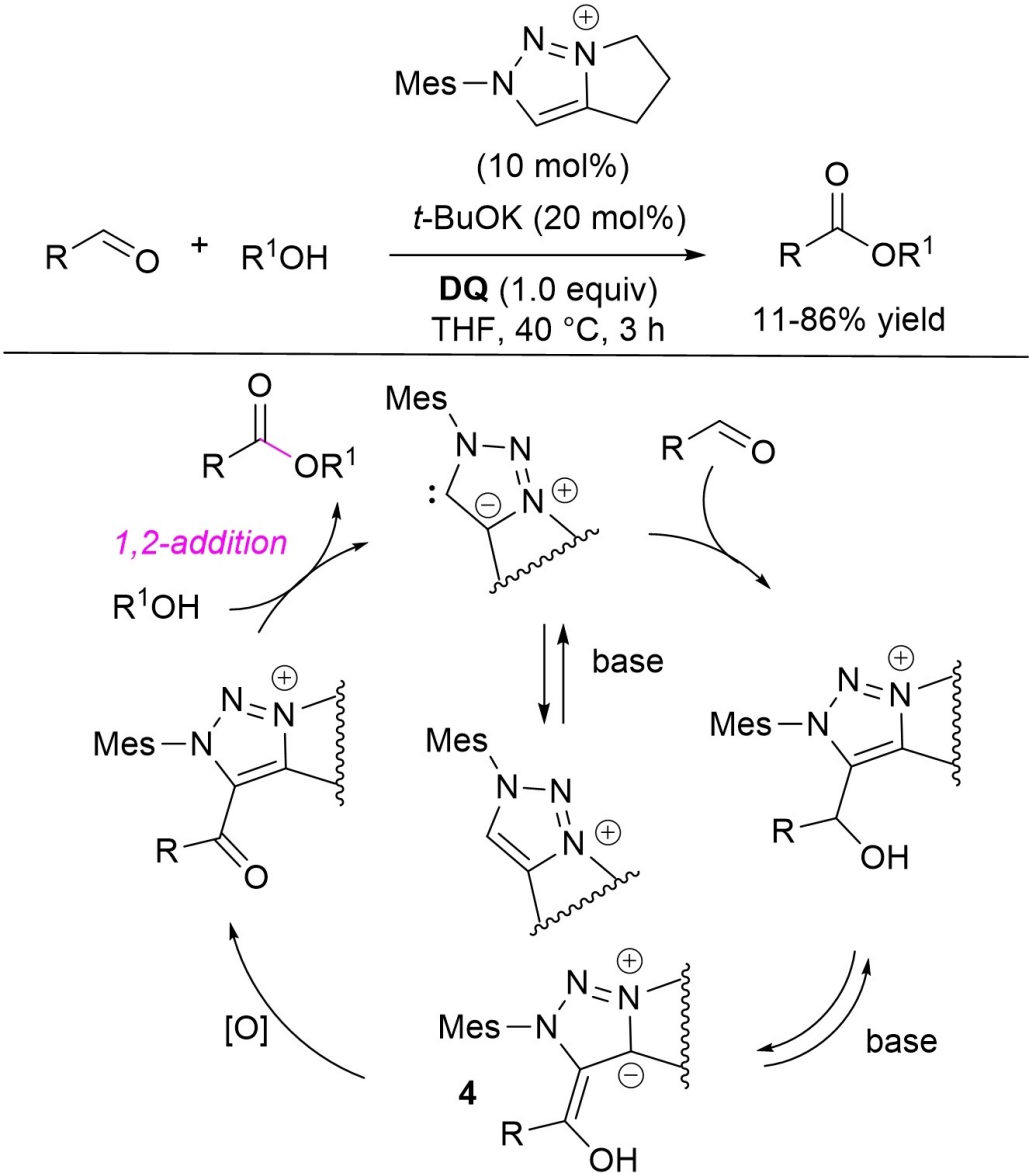
Oxidative esterification of aldehydes promoted by mesoionic 1,2,3‐triazolyl carbene organocatalyst.

Mesoionic 1,2,3‐triazolyl carbene organocatalysts facilitated the oxidative esterification of (hetero)aromatic and α,β‐unsaturated aldehydes in the presence of **DQ**, using *t*‐BuOK as base (Scheme 9).^[17]^ In‐depth NMR investigations have led to elucidate the correlation between reactivity and catalyst acidity, the catalyst containing a saturated 5‐membered ring and the electron‐rich mesityl *N*‐substituent being the most effective. Other than that, kinetic studies along with control experiments have shed light on a possible catalytic cycle, calling for the formation of the key acyl azolium ion via oxidation of the Breslow‐like intermediate **4** rather than by direct oxidation of the initial carbene‐aldehyde adduct.

**DQ**‐assisted coupling of aldehydes with alcohols under NHC‐catalysis has been shown to represent a suitable strategy for controlled functionalization of bio‐based chemicals. So, Studer and co‐workers described the regioselective acylation of differently protected carbohydrates (glucose, mannose, galactose) with *o*,*o*’‐dihalo‐substituted benzaldehydes.^[18]^ Very high stereoselectivity was observed for both *cis*‐ and *trans*‐ secondary diol isomers (isomer ratio 1 : 9 to >99 : 1), also compared to a standard (unselective) acyl chloride/pyridine acylating system. This is likely due to the pivotal double role held by the two *ortho*‐halogen atoms: they electronically activate the aldehyde and on the other hand add steric hindrance to the acyl azolium ion.

**Scheme 10:**
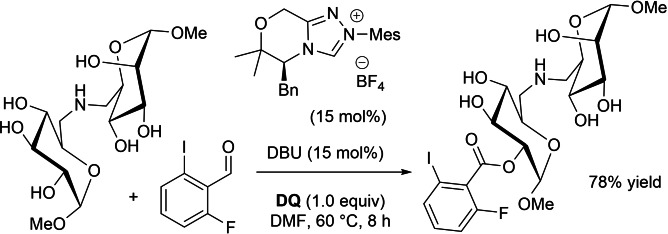
Regioselective acylation of carbohydrates through oxidative NHC‐catalysis.

Particularly worth mentioning is that complete selectivity for *O*‐acylation over *N*‐acylation was fulfilled for amino sugars, including an amino‐bridged neodisaccharide derivative (Scheme 10). Furthermore, both selectivity and efficiency could be improved by cooperation of two different NHC catalysts: one catalyst becomes part of the acylating species while the other (enantiomer, achiral NHC, different chiral NHC) is supposed to activate the alcohol substrate by H‐bonding.^[19]^

**Scheme 11:**
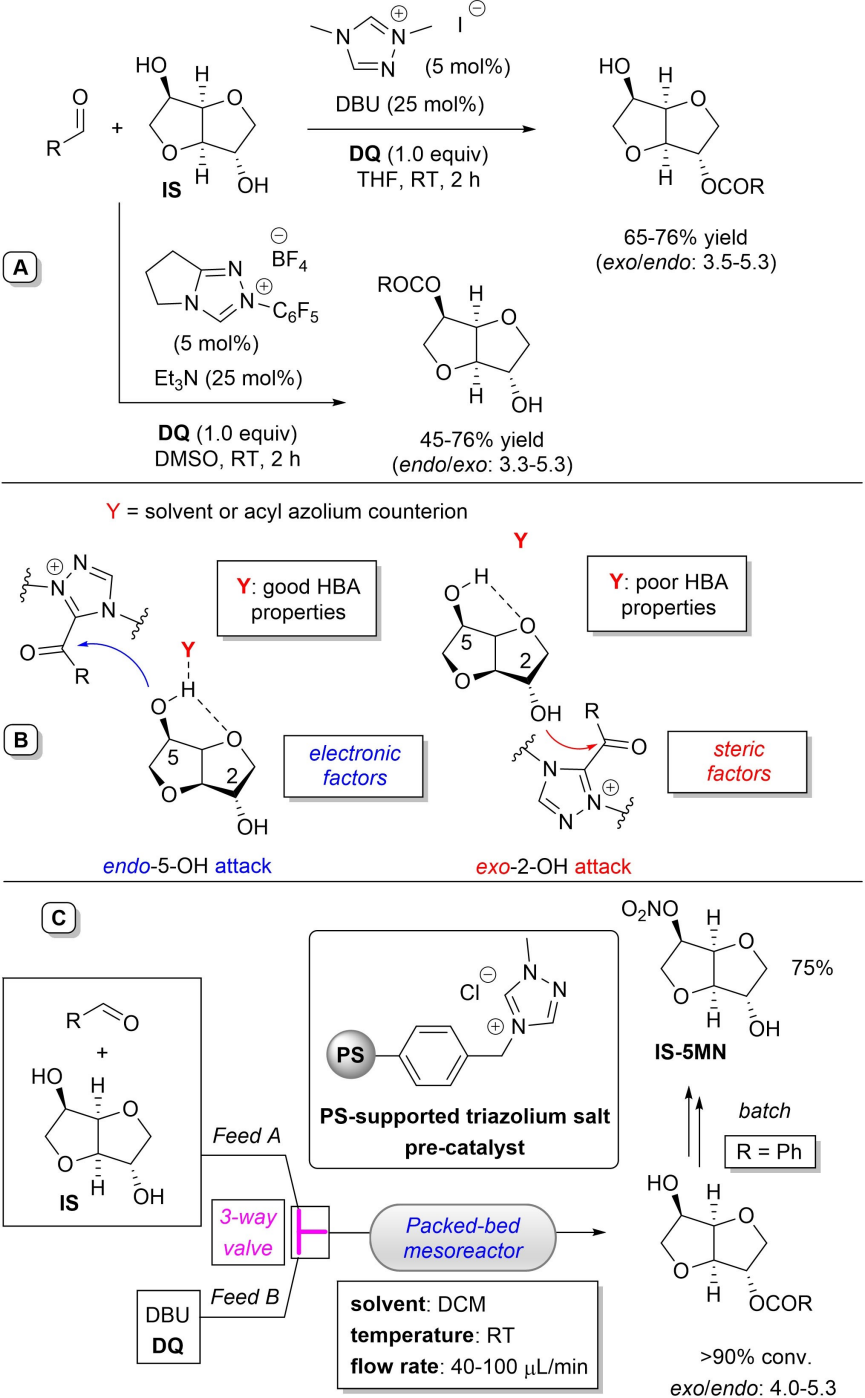
NHC‐catalyzed oxidative acylation of **IS** in batch and continuous‐flow conditions.

In 2021, Massi and Ragno group reported a NHC‐promoted strategy for the regioselective acylation of isosorbide (**IS**, 1,4 : 3,6‐dianhydro‐D‐glucitol) with aldehydes under oxidative conditions in the presence of stoichiometric **DQ**.^[20]^ Optimal reaction conditions were found for the preparation of both *endo*‐ and *exo*‐monoacylisosorbides (MAIs) using aromatic aldehydes, including the two bio‐based congeners furfural (FF) and 5‐hydroxymethyl furfural (HMF), and α,β‐unsaturated aldehydes as proper acylating agents (Scheme 11A). Accordingly, dimethyl triazolium iodide (5 mol%) and 1,8‐diazabiciclo[5.4.0]undec‐7‐ene (DBU, 25 mol%) were effectively used to obtain *exo*‐MAIs (65–76 % yield, *exo*/*endo* selectivity: 3.5–5.3), while the synthesis of *endo*‐MAIs was made possible thanks to *N*‐pentafluorophenyl pyrrolidine‐fused triazolium salt in combination with Et_3_N (45–76 % yield, *endo*/*exo* selectivity: 3.3–5.3).

The regioselectivities observed in the NHC‐catalyzed oxidative esterifications of **IS** have been rationalized in terms of stereoelectronics of the in situ formed acyl azolium, possibly influenced by the nature of the solvent (Scheme 11B). It has been proposed that steric factors prevail in solvents with poor hydrogen‐bond‐accepting (HBA) properties, the bulkiness of the acyl azolium intermediate directing acylation at the more accessible *exo*‐2‐OH, similarly to what it applies for the DCC‐promoted esterification of **IS** via *O*‐acyl isourea intermediates.^[21]^ On the other hand, electronic factors are expected to dominate when suitable HBA solvent and/or acyl azolium counterion are involved, attack by the more nucleophilic *endo*‐5‐OH being privileged.^[22]^

Overall, conventional solvents (THF/DCM, DMSO) have proved to be superior to green solvents (2‐methyltetrahydrofuran, γ‐valerolactone, dimethyl isosorbide, ethyl lactate, acetylcholine chloride‐urea deep eutectic solvent, (*R*)‐(+)‐limonene) in directing the *exo*‐ and *endo*‐ regioselectivities, thus making them ideal for the developed methodology. This plays well for greenness and/or environmental compatibility, in accordance with the guidelines provided by pharmaceutical industries.^[23]^

Further to this, and in the perspective of process intensification, a batch heterogeneous procedure was implemented through fabrication of a polystyrene (PS)‐supported version of the *exo*‐selective triazolium salt pre‐catalyst, providing results comparable to those of the homogeneous parent compound (DCM, RT, 69–79 % yield, *exo*/*endo* selectivity: 3.6–5.3). Added benefits of this approach were the easy recovery of both solvent (distillation) and oxidant, with the latter recycled by FePc/air oxidation of the reduced (diol) form that is produced during the acylation process.

Next, transition to continuous‐flow regime (packed‐bed mesoreactor) led to the production of *exo*‐MAIs with the same level of selectivity (*exo*/*endo*: 4.0–5.3) and high conversion (>90 %) (Scheme 11C). Remarkably, 2‐benzoyl‐IS could be produced in multigram scale (3.2 g) and used as the key precursor of pharmaceutically relevant isosorbide‐5‐mononitrate (IS‐5MN).^[24]^

The PS‐supported triazolium salt/DBU/**DQ** system has been thoroughly designed to study how solvent effect can impact on catalyst activity in NHC‐promoted oxidative esterification reactions, in particular using the coupling of 2‐chlorobenzaldehyde and methanol as model.^[25]^ Diffusion and NMR (*T*_1_/*T*_2_) relaxation studies using polar (THF, DMF, DCM), slightly polar (toluene) and nonpolar (cyclohexane) solvents demonstrated that catalyst activity (reaction rate) strictly depends on interaction of the solvent with the catalyst surface: the stronger the solvent affinity for the surface of the porous solid support, the lower the catalytic activity. Most probably, the high‐affinity solvent prevents access of the reactant molecules to the catalytic sites over the surface, likewise that observed in the supported‐metal catalyzed oxidation of diols.^[26]^ It is noted that these conclusions do not take account of other effects that are typical of homogeneous reactions, among others proticity, polarizability or basicity that might take part in transition state stabilization/destabilization.

**Scheme 12:**
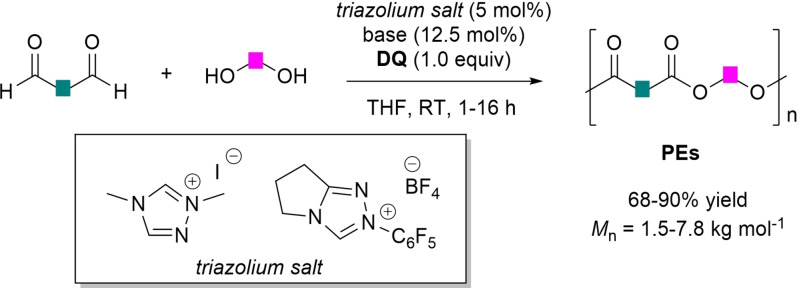
Synthesis of PEs by oxidative NHC‐catalysis.

**DQ**‐aided oxidative NHC‐catalysis was successfully applied to the synthesis of polyester oligomers (PEs) via step‐growth polymerization, starting from fossil‐ and bio‐based dialdehyde and diol monomers (Scheme 12).^[27]^

Optimized reaction conditions were found for the reaction between ethylene glycol and terephthalaldehyde in the presence of dimethyl triazolium iodide (5 mol%), DBU (12.5 mol%) and stoichiometric **DQ** (THF, RT, 16 h), leading to polyethylene terephthalate (PET) (82 % isolated yield, >95 % conv.) with a number‐average molecular weight (*M*_n_) of 6.5 kg mol^−1^, even on a gram‐scale (10.0 mmol of aldehyde, 88 % yield). Importantly, sustainable implementation of the protocol was assessed by recycling both the base (acidic treatment) and the oxidant (FePc/air oxidation of the corresponding diol).

Interestingly, PET was eventually taken to high *M*_n_ PET (78 % yield) by heating at 250 °C (2 h, vacuum) in the presence of equimolar (catalytic) amounts of the same triazolium pre‐catalyst and DBU base (5 mol% each).^[28]^

By analogy, polyethylene isophthalate (PEI) oligomers (*M*_n_=6.5 kg mol^−1^, 77 % yield) could be obtained from ethylene glycol and isophthalaldehyde, and so is bio‐based polymers (*M*_n_=1.5–7.8 kg mol^−1^, 68–81 % yield) have been prepared by various combinations of renewable monomers (glycerol, furan dialdehydes/diols, IS).

The optimized polycondensation strategy also showed good prospect for those substrates which are not very reactive in NHC‐promoted esterification reactions. In this regard, successful results were obtained using benzene‐1,3,5‐tricarboxaldehyde/ethylene glycol and hydroquinone/terephthalaldehyde substrate combinations, with the polymer products formed in 79 % and 88 % yield, respectively.

It is worthy of note that polymer architecture (linear or cross‐linked) may be controlled by a proper variation of NHC structure (steric hindrance) in order to target regioselective activation of the polyol substrate, the mild reaction conditions preserving the polyester microstructure for effect of the total absence of acyl group migration. As a matter of fact, glycerol and terephthalaldehyde were transformed into cross‐linked poly(glycerol terephthalate) (PGT, 90 % yield) using dimethyl triazolium salt (DBU base), while *N*‐pentafluorophenyl pyrrolidine‐based triazolium salt and Et_3_N were adopted to achieve the preparation of linear PGT (*M*_n_=1.5 kg mol^−1^, 71 % yield).

**Scheme 13:**
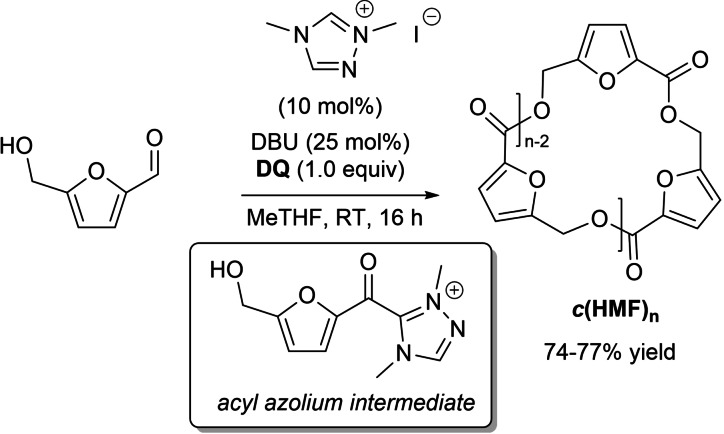
NHC‐catalyzed synthesis of *c*(HMF)_n_ under oxidative conditions.

Stoichiometric **DQ** and the catalytic system formed by dimethyl triazolium iodide (10 mol%) and DBU (25 mol%) promoted the polycondensation of HMF (via acyl azolium intermediate) to hydroxymethylfuroate macrocyclic oligoesters (*c*(HMF)_n_, mainly trimer/tetramer species) (Scheme 13),^[29]^ building blocks for the synthesis of high molecular weight poly(hydroxymethylfuroate) (PHMF, *M*_n_=5.1–48.6 kg mol^−1^) through entropically‐driven ring‐opening polymerization (ED‐ROP) promoted by 1,5,7‐triazabicyclo[4.4.0]dec‐5‐ene (TBD).^[30]^

Satisfactory selectivity for the desired *c*(HMF)_n_ (74–77 % yield) has been met under high dilution conditions in the green solvent 2‐methyltetrahydrofuran (MeTHF), providing the advantage that it could be recycled (distilled) when used in gram‐scale preparations (6.5 mmol of HMF).

**Scheme 14:**
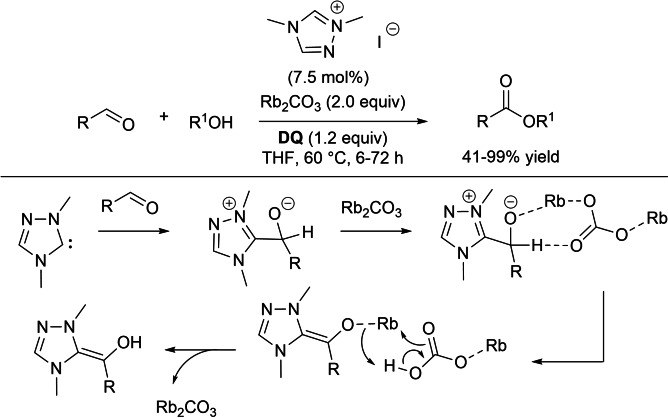
Rb_2_CO_3_‐promoted oxidative esterification of aliphatic aldehydes.

With aliphatic aldehydes, it is well established that the oxidation process with **DQ** is very unyielding, stimulating new study and research to inverse such behaviour. Thus, Samanta and Studer were able to find a suitable method for the oxidative esterification of aliphatic aldehydes using dimethyl triazolium iodide pre‐catalyst (7.5 mol%), rubidium carbonate (2.0 equiv.), and **DQ** (1.2 equiv.) (Scheme 14).^[31]^

A broad substrate scope was demonstrated in terms of both linear and α‐ and β‐branched aliphatic aldehydes, the reactivity of the former strictly depending on the length of the alkyl chain. What is remarkable is that the inorganic base seems to play a key role in catalyzing a two‐step 1,2‐proton transfer towards the formation of Breslow intermediate, due to the average cation size and consequent moderate basicity.

It goes sure said that the Rb_2_CO_3_‐promoted oxidative esterification method showed to have potential for intramolecular lactonization, however no possible use for stereospecific transformations has been proved.

**Scheme 15:**
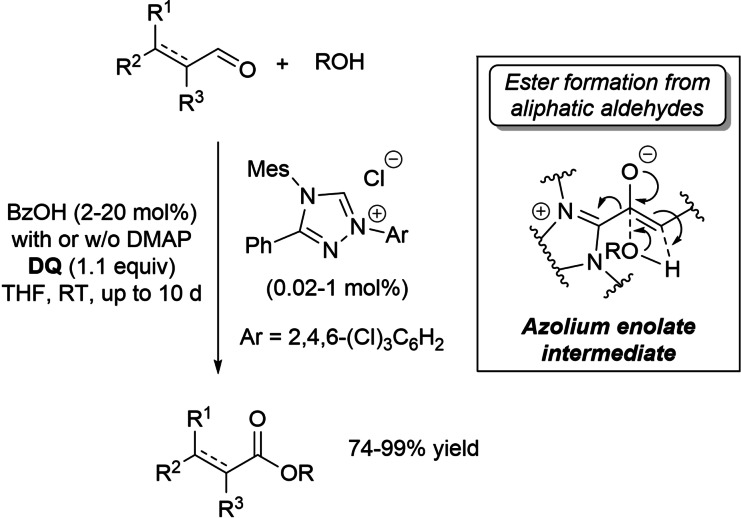
Oxidative esterification of demanding aldehydes/enals through NHC/BzOH co‐catalysis.

Recently, the group of Berkessel and Harnying has achieved the NHC‐catalyzed oxidative esterification of a large series of demanding aldehydes/enals with alcohols by the means of NHC/carboxylic acid cooperative catalysis.^[32]^ Best conditions were disclosed to couple methanol, as well as primary, allylic, (hetero)benzylic alcohols of different lengths with unramified and α/β‐branched aliphatic aldehydes/enals, using the low‐basicity *N*‐mesityl‐*N*‐2,4,6‐trichlorophenyl 1,2,4‐triazolium salt in combination with benzoic acid (BzOH) co‐catalyst (with or without DMAP base promoter) (Scheme 15). Typical catalyst loadings of 0.02–1 mol% (2–20 mol% of BzOH, 74–99 % yield) could be used, and dramatically reduced to 0.005 mol% (50 ppm) in the case of the more reactive benzaldehyde (0.05 mol% of BzOH, 93 % yield).

Mechanistically, kinetic studies on the role of BzOH for the esterification reactions have suggested that ester formation from the (enolizable) aliphatic aldehydes goes through an azolium enolate intermediate,^[33]^ the acid co‐catalyst accelerating acyl transfer to the alcohol substrate. On the contrary, reaction rate is little affected in the case of benzaldehyde (enals): ester formation inevitably proceeds via acyl azolium species, and BzOH thus acts to preclude (retard) catalyst decomposition.

**Scheme 16:**
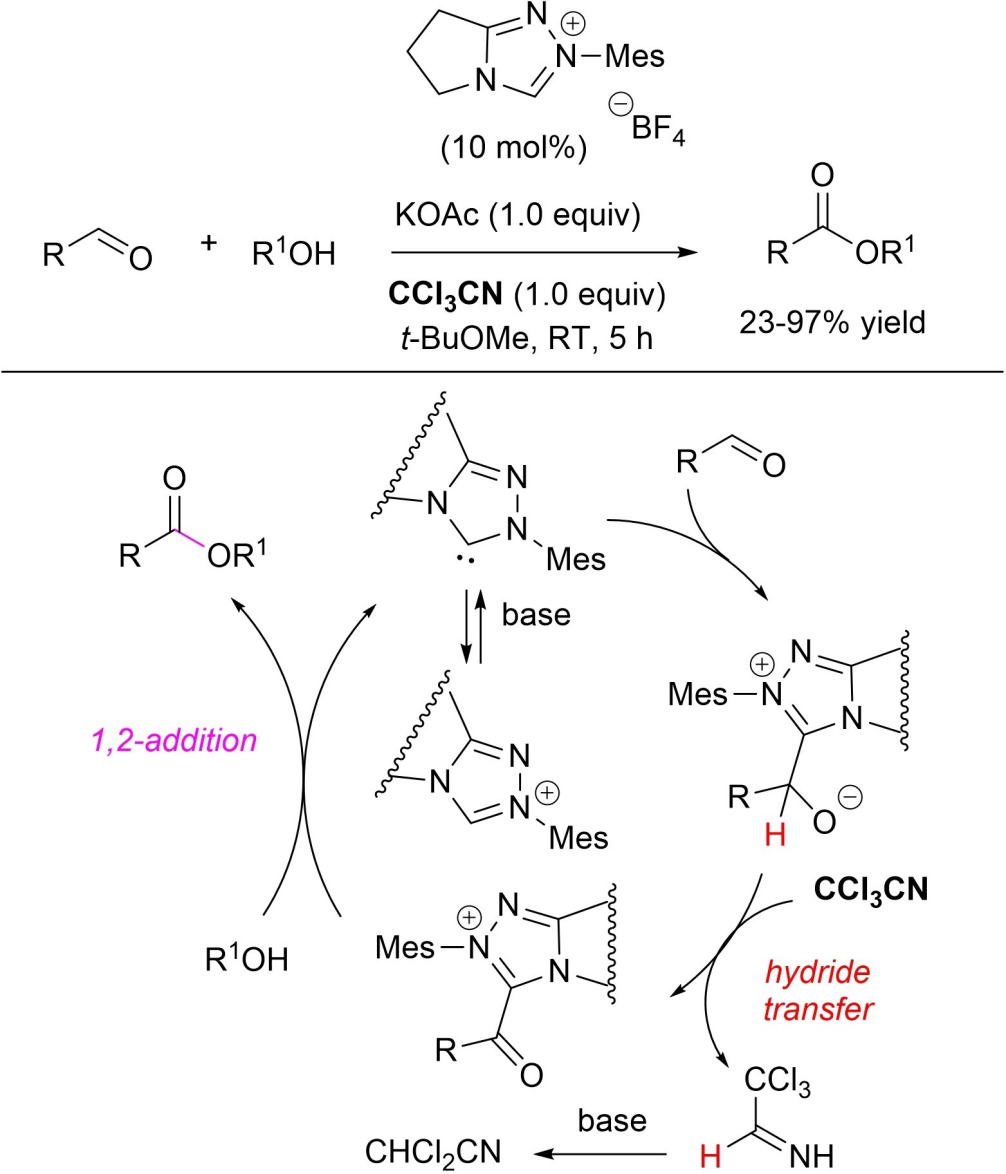
Oxidative esterification of aldehydes promoted by CCl_3_CN.

An atypical NHC‐catalyzed intermolecular *O*‐acylation reaction protocol has been demonstrated with CCl_3_CN,^[34]^ giving access to a wide variety of esters from a diverse set of aldehydes (aliphatic, aromatic, heteroaromatic, enals, ynals) and primary/secondary alcohols, including naturally sourced ones (steroids, terpenes, carbohydrates), other than phenols, hemiacetals, hemiaminals, and hydroxylamines (Scheme 16). A plausible mechanistic pathway calls for formation of the reactive acyl azolium intermediate by hydride transfer from the initially formed NHC/aldehyde adduct to CCl_3_CN, its reduced form being eventually isolated from the reaction mixture upon base‐induced elimination of in situ generated 2,2,2‐trichloroethan‐1‐imine.

**Scheme 17:**
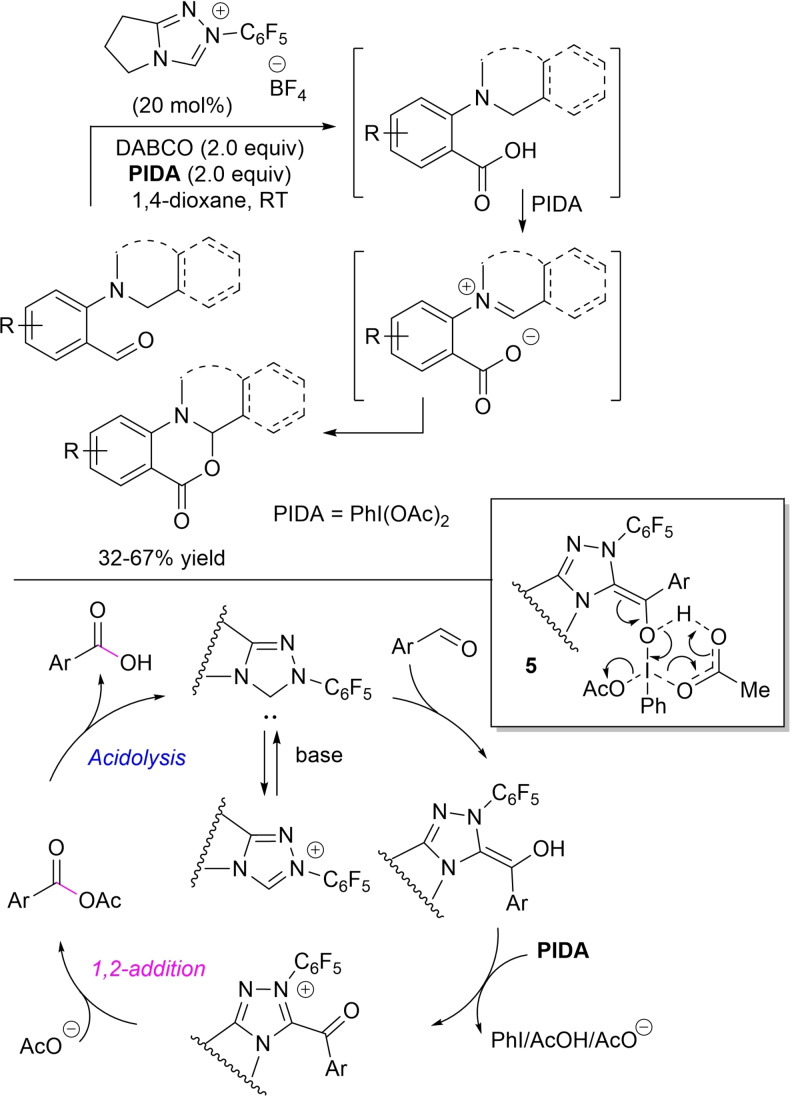
NHC‐catalyzed oxidation of aldehydes to carboxylic acids promoted by PIDA.

As part of a project for intramolecular α‐oxygenation of amines with aldehydes by NHC‐catalysis, tetrahydroisoquinoline‐, pyrrolidine‐, piperidine‐, azepane‐, and morpholine‐derived benzaldehydes were subjected to the action of *N*‐pentafluorophenyl pyrrolidine‐based triazolium salt (20 mol%), 1,4‐diazabicyclo[2.2.2]octane (DABCO, 2.0 equiv.) and PIDA (2.0 equiv.) as the oxidant, turning into carboxylic acids eventually converted to iminium‐carboxylates, keys to the intramolecular cyclization step (Scheme 17).^[35]^

Density functional theory (DFT) calculations have indicated that Breslow intermediate suffers electrophilic attack on its hydroxyl group by PIDA via the six‐membered transition state **5**, resulting in the formation of the decisive acyl azolium ion with concomitant release of iodobenzene, acetic acid and acetate anion. Then, addition‐elimination by this last one and acidolysis form the carboxylic acid product.

**Scheme 18:**
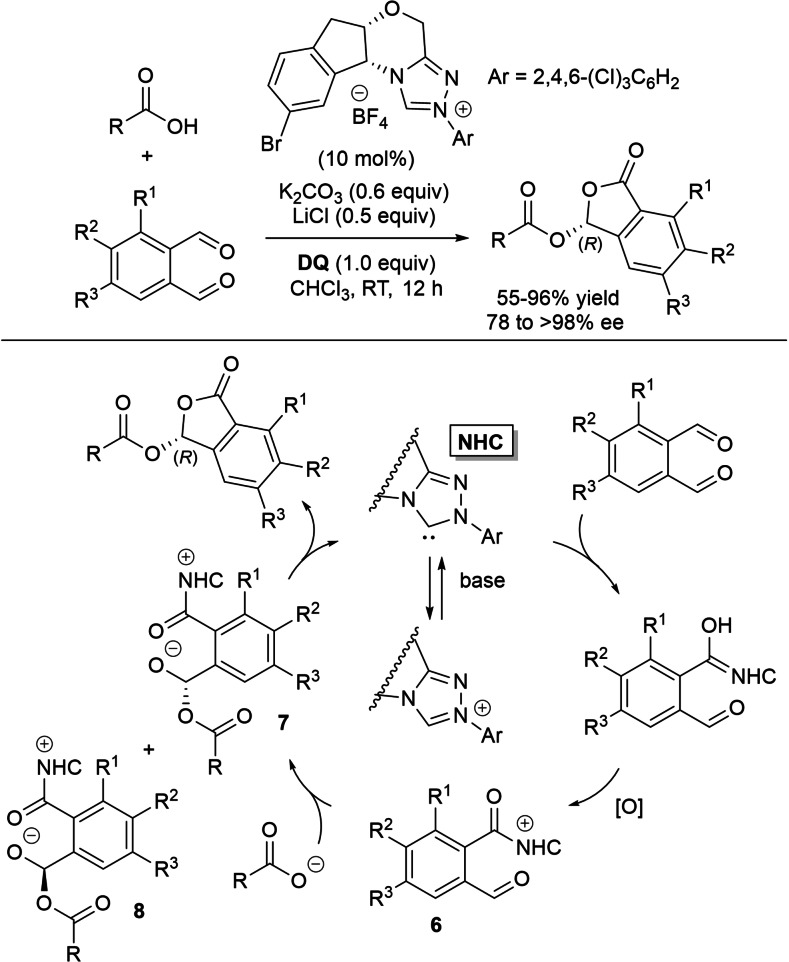
NHC‐catalyzed oxidative asymmetric acetalization of carboxylic acids.

Synthesis of chiral phthalidyl esters has been accomplished from *o*‐phthalaldehydes and carboxylic acids through NHC‐catalyzed intramolecular acetalization reactions in the presence of **DQ** (Scheme 18).^[36]^

Both saturated/unsaturated aliphatic and (hetero)aryl carboxylic acids performed well with *o*‐phthalaldehyde to give the end products with good to excellent yields (55–96 %) and enantioselectivities (82–96 % ee), application of the process on gram‐scale (1.48 g of product) being demonstrated with 6‐bromohexanoic acid (1 mol% NHC, 85 % yield, 94 % ee).

Equally successful were the reactions of substituted and unsymmetric dialdehydes with 2‐(4‐bromophenyl)acetic acid (78–86 % yield, 90–96 % ee), and what is notable is that natural products (*R*‐hydratropic acid, sorbic acid, abietic acid) and commercially used drugs (dehydrocholic acid, naproxen, valproic acid, nicotinic acid, chlorambucil) proved to be suitable carboxylic acid counterparts of phthalaldehyde (62–90 % yield, 78 to >98 % ee).

In connection with reaction mechanism, it is assumed that a single aldehyde moiety of phthalaldehyde is converted into (chiral) acyl azolium **6**, while the other aldehyde group enters into nucleophilic addition on the part of the in situ formed carboxylate anion. As a result, diastereomeric alkoxides **7** and **8** are formed, the former preferentially triggering the final intramolecular annulation.

**Scheme 19:**
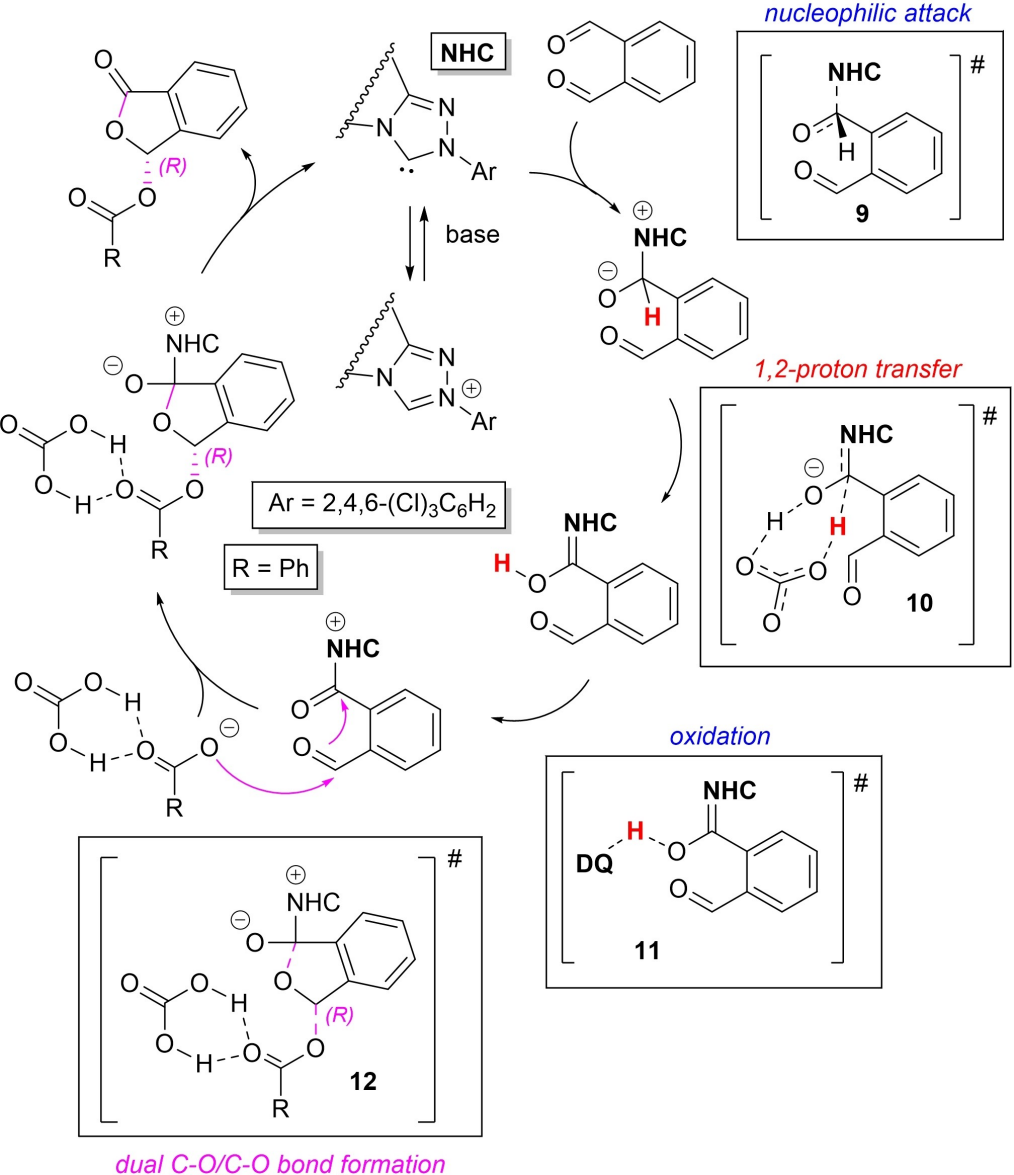
Detailed mechanism for NHC‐catalyzed oxidative acetalization of benzoic acid.

On the subject, a recent computational study by Qiao and Wei on the reaction between *o*‐phthalaldehyde and benzoic acid has helped clarify both the detailed mechanism and the origin of stereoselectivity for the NHC‐catalyzed asymmetric acetalization reaction (Scheme 19).^[37]^

Preferential *Si*‐face attack of NHC on the aldehyde group of phthalaldehyde (transition state **9**) is followed by *i)* HCO_3_^−^‐ assisted 1,2‐proton transfer (transition state **10**)^[38]^ and *ii)* oxidation of the Breslow intermediate thus formed (transition state **11**). Next, dual C−O/C−O bond assemblage comes in a concerted fashion, the route moving towards the *R*‐configured isomer being energetically more favorable than that for the *S*‐configurational one. This is likely the result of O−H‐ ‐ ‐O and C−H‐ ‐ ‐O hydrogen bond interactions in the key transition state **12**, as revealed by quantitative atom‐in‐molecule (AIM) and qualitative non‐covalent interaction (NCI) analyses.

**Scheme 20:**
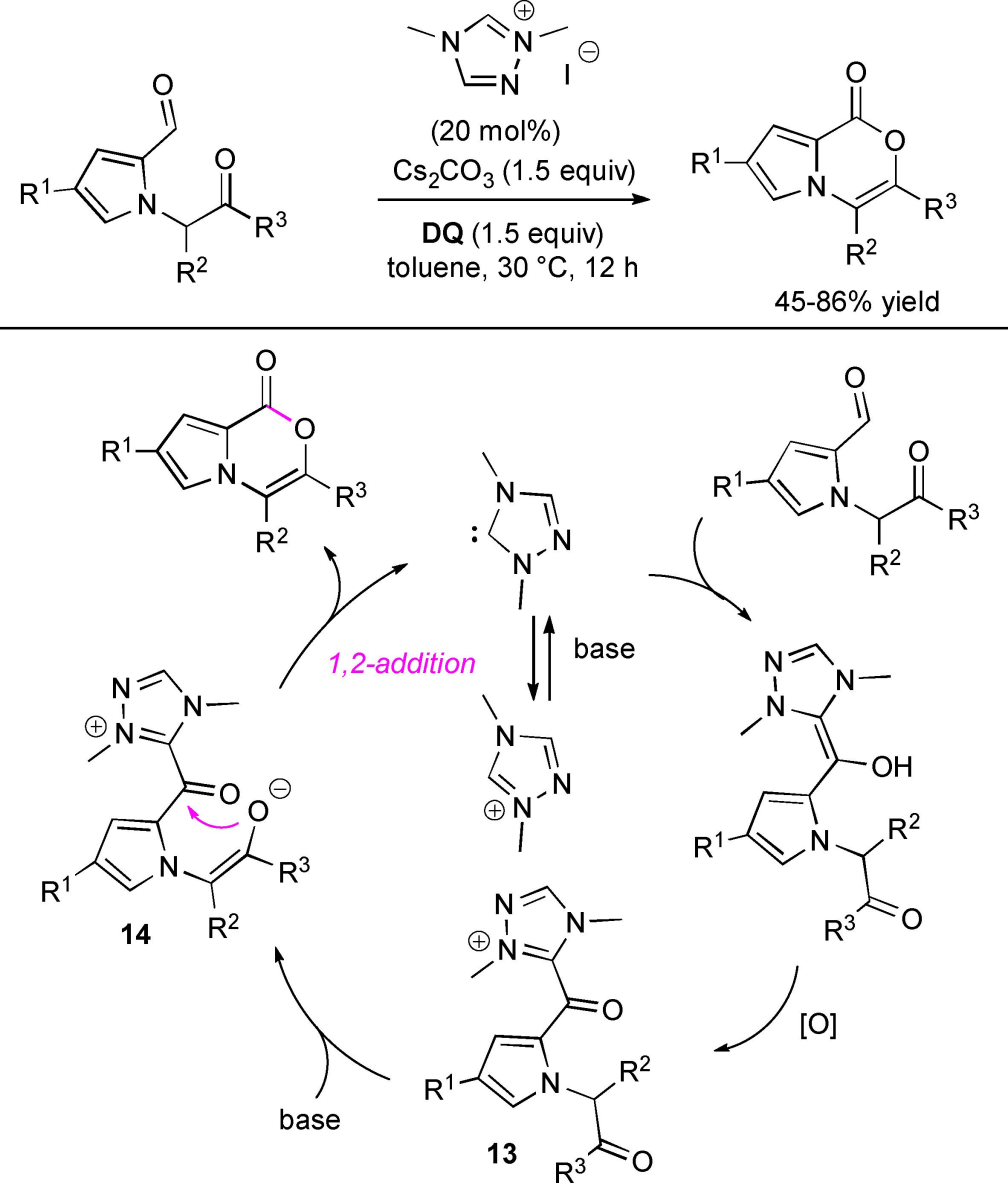
Intramolecular acylation of enolates with acyl azoliums.

Intramolecular acylation of a ketone enolate was the key step in the synthesis of functionalized pyrrolo‐oxazinone derivatives starting from suitable *N*‐substituted pyrrole 2‐carboxaldehydes, dimethyl triazolium iodide pre‐catalyst (20 mol%), cesium carbonate (1.5 equiv.) and **DQ** (1.5 equiv.) (Scheme 20).^[39]^ In addition to mild reaction conditions, the annulation reaction showed good performance (45–86 % yield), scalability (1.0 mmol scale, 70 % yield), and broad functional group compatibility at the level of both *C*‐ and *N*‐substitution on the pyrrole ring.

A series of control experiments have supported the hypothesis that the usual nucleophilic attack/proton transfer/oxidation sequence, starting from NHC and the pyrrole carboxaldehyde, yields acyl azolium intermediate **13**, precursor of enolate **14** undergoing the decisive intramolecular 1,2‐nucleophilic addition with NHC fragmentation.

**Scheme 21:**
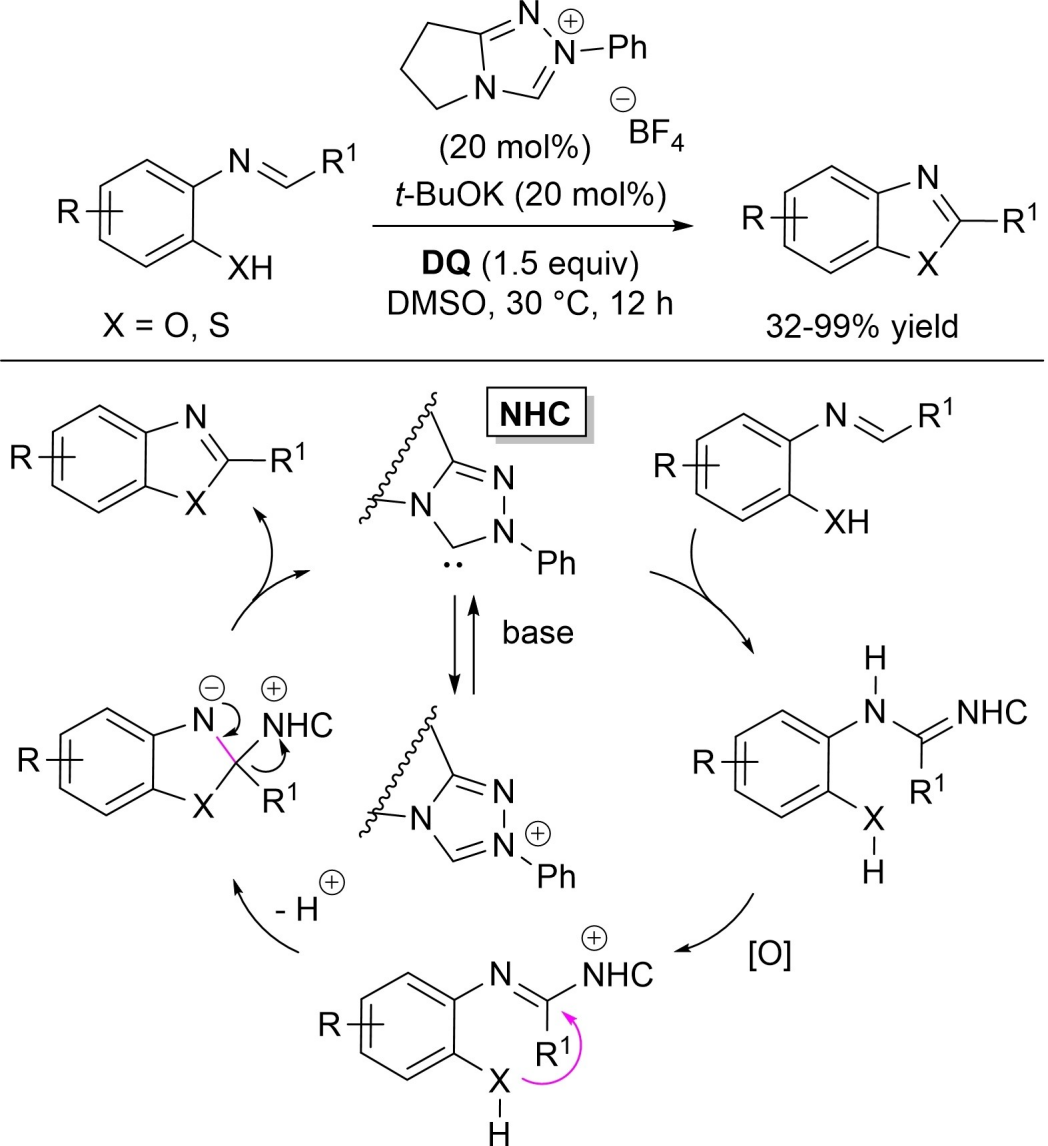
Synthesis of 2‐arylbenzoxazoles and 2‐arylbenzothiazoles via imidoyl azolium intermediate.

In 2018, Biju and co‐workers implemented oxidative NHC‐catalysis to generate imidoyl azoliums for use in intramolecular 1,2‐addition reactions of *O*‐ and *S*‐nucleophiles, leading the way to 2‐arylbenzoxazole and 2‐arylbenzothiazole products.^[40]^ On this matter, the aldimines generated from 2‐amino(thio)phenols and (hetero)aromatic aldehydes/ferrocenecarboxaldehyde were exposed to *N*‐phenyl‐substituted pyrrolidine‐fused triazolium pre‐catalyst (20 mol%), *t*‐BuOK (20 mol%) and **DQ** (1.5 equiv.), with the ultimate heterocyclic compounds obtained in yields of 32–99 % (Scheme 21). The proposed mechanism of the reaction envisages that the imine‐derived *aza*‐Breslow intermediate gets oxidized to imidoyl azolium, that undergoes an intramolecular nucleophilic addition to the C=N bond. Then, the so formed zwitterion gives place to desorption of the NHC catalyst with release of the aromatic bicyclic product.

**Scheme 22:**
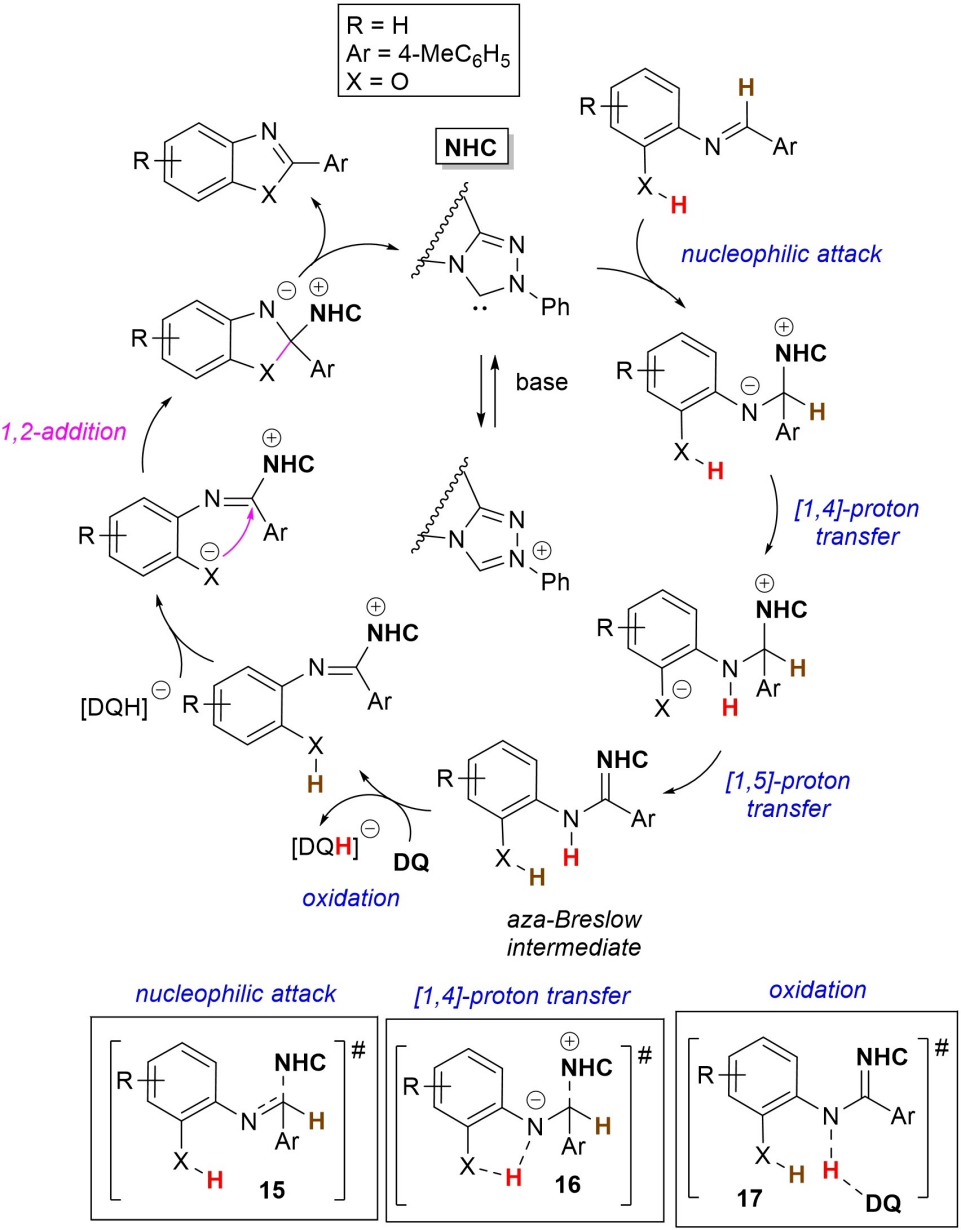
DFT‐based mechanism for conversion of aldimines to benzoxazoles under oxidative NHC‐catalysis.

Recently, an in‐depth DFT study was conducted relative to this catalytic cycle, in order to substantiate it by verification of both rationality and feasibility (Scheme 22).^[41]^

Taking the transformation of the imine derived from 2‐aminophenol and *p*‐tolylaldehyde as the model, it has been demonstrated that *Si*‐face attack of NHC on the imine reagent (transition state **15**) is followed by sequential [1,4]‐proton transfer (transition state **16**) and spontaneous [1,5]‐proton transfer (tautomerization) to yield the *aza*‐Breslow intermediate. This takes then part in the oxidation step (hydride transfer to **DQ**, transition state **17**), after which deprotonation of the hydroxy/thiol group traces the way for the intramolecular cyclization and final product formation. This five‐step route from the NHC‐imine adduct has been computed as the more energetically advantageous compared to those that involve the formation of the imidoyl azolium by direct oxidation or by [1,2]‐proton transfer/oxidation.

In continuation of the work on the **DQ**‐promoted NHC‐catalyzed synthesis of MAIs,^[20]^ the regiodivergent synthesis of *endo*‐ and *exo*‐ monoimidate‐isosorbides (MIIs) equipped of biologically important *N*‐heterocycles (benzothiazole, benzoxazole, thiazole, isoxazole) was recently studied by Bortolini group.^[42]^

**Scheme 23:**
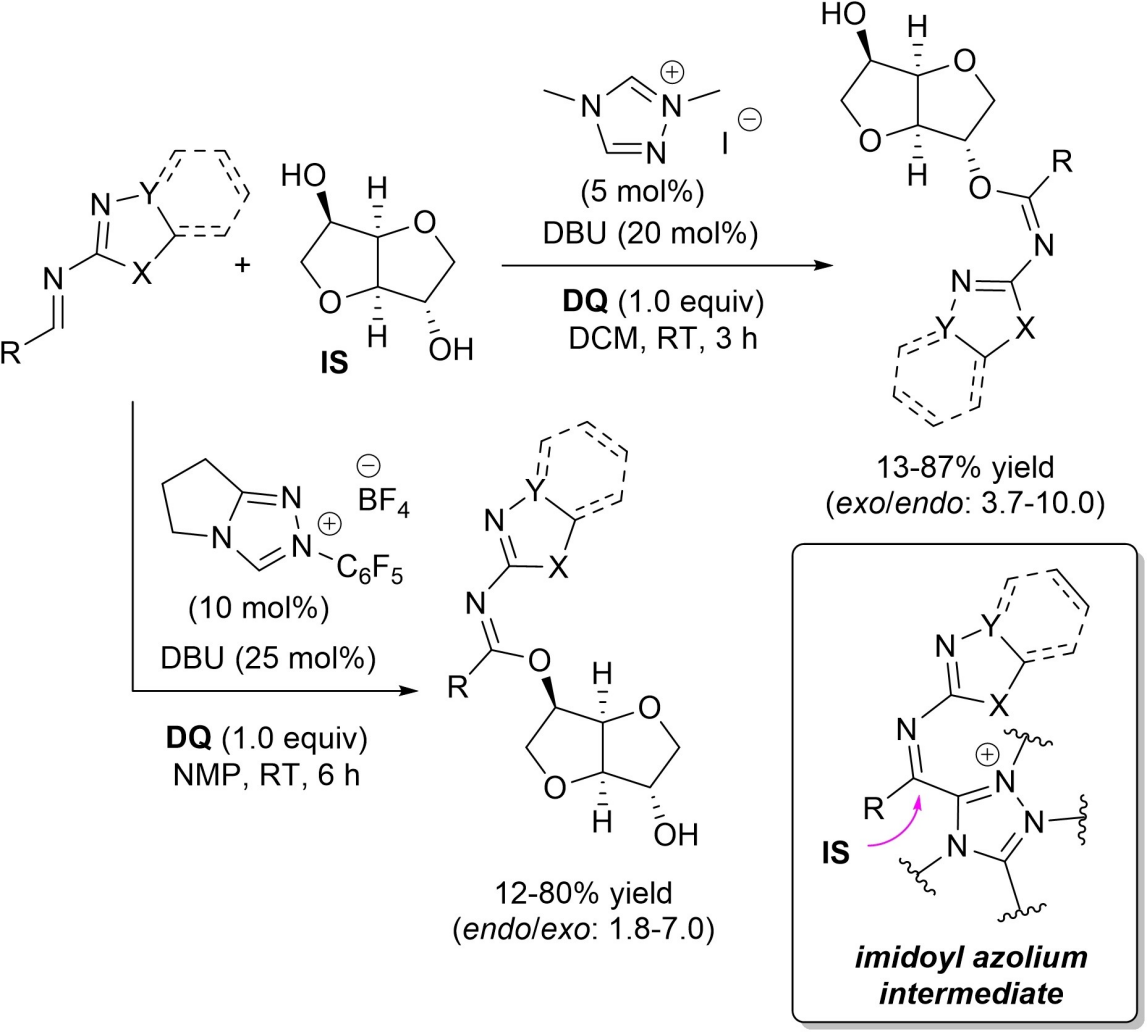
NHC‐catalyzed imidation of **IS** under oxidative conditions.

This strategy relied on oxidative NHC‐catalyzed reactions of **IS** with (hetero)aromatic aldimines derived from 2‐aminobenzothiazole and congeners (Scheme 23). Most performing conditions for the production of *endo*‐MIIs (12–80 % yield, *endo*/*exo* selectivity: 1.8–7.0) involved the use of *N*‐pentafluorophenyl pyrrolidine‐based triazolium salt (10 mol%), DBU (25 mol%) and stoichiometric **DQ** in *N*‐methyl‐2‐pyrrolidone (NMP) solvent, its strong HBA character helping preferential reaction of the more nucleophilic 5‐OH of **IS** (dominant electronic factors). Conversely, matching dimethyl triazolium iodide (5 mol%) with DBU (20 mol%) in DCM assisted in the attainment of *exo*‐MIIs (13–87 % yield, *exo*/*endo* selectivity: 3.7–10.0), with involvement of the less sterically hindered 2‐OH of **IS** determined by the scanty solvent HBA features (prevailing steric factors).

As anticipated, the oxidative route to MIIs takes place through imine‐to‐imidoyl azolium conversion and successive nucleophilic addition of **IS** to the C=N bond (with departure of NHC catalyst).

It should be noted that preliminary studies with air as terminal oxidant were attempted, using the reaction of **IS** with benzothiazole‐containing benzaldimine as the benchmark. Under the best conditions for the synthesis of *exo*‐ and *endo*‐derivatives, promising findings in terms of *exo*‐selectivities (*exo*‐MII, 77 %; *endo*‐MII, 16 %) and *endo*‐selectivities (*exo*‐MII, 14 %; *endo*‐MII, 54 %) have emerged by applying the ETMs system, which minimized formation of the oxygenative amide product.

Of no less importance is the synthetic opportunity given by the NHC‐catalyzed oxidative esterification strategy for desymmetrization of prochiral substrates, founded upon stereodiscrimination during the nucleophilic attack to the reactive acyl azolium intermediate.^[43]^

**Scheme 24:**
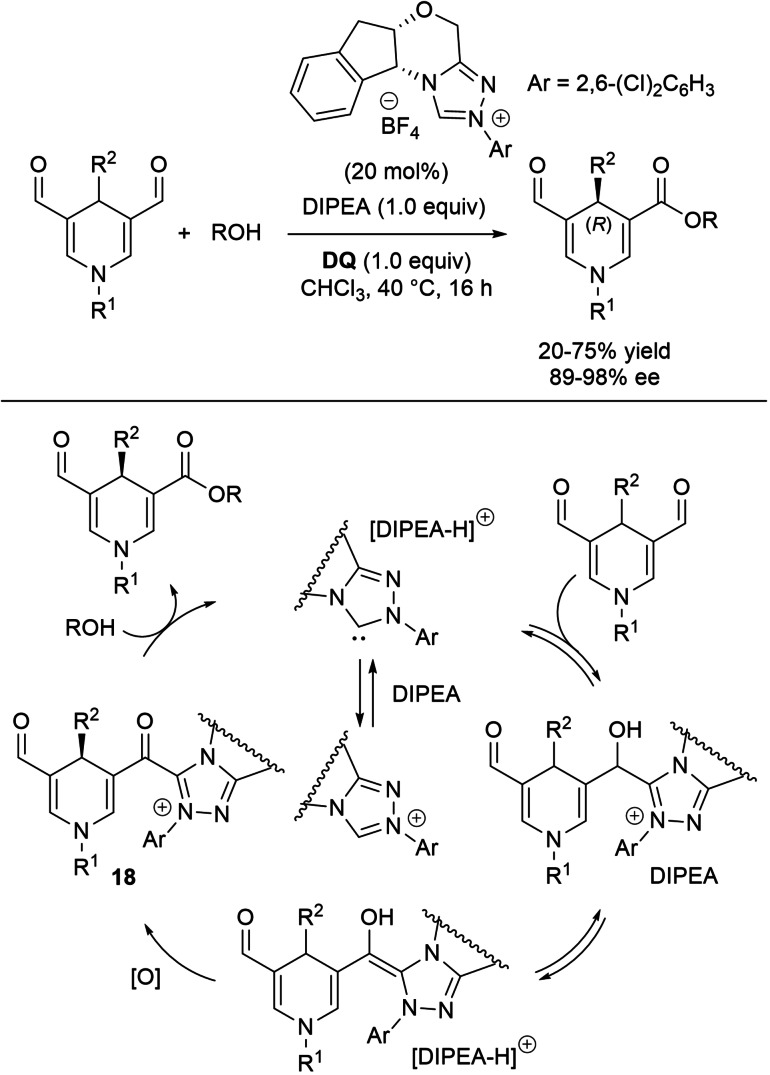
NHC‐catalyzed enantioselective desymmetrization of 1,4‐DHPs under oxidative conditions.

This was the concept behind the enantioselective synthesis of 5‐formyl‐1,4‐dihydropyridine‐3‐carboxylates from 1,4‐dihydropyridine‐3,5‐dicarboxaldehydes.^[44]^ The latter underwent the action of (1*R*,2*S*)‐1‐amino‐2‐indanol‐derived triazolium salt (20 mol%), diisopropylethylamine (Hünig's base, DIPEA, 1.0 equiv.) and **DQ** (1.0 equiv.) in the presence of aliphatic saturated/unsaturated alcohol nucleophiles (Scheme 24). These conditions gave enantioenriched 1,4‐dihydropyridines (DHPs) with (4*R*)‐configuration in poor to good yields (20–75 %) and very good to excellent enantioselectivities (89–98 % ee). Both alkyl and (substituted) phenyl groups at C4 were suited to the desymmetrization process, while the N1 position was more sensitive to the nature of the substituents, an alkyl one considerably lowering yield (20 %) but without affecting selectivity (98 % ee).

Investigation of the reaction mechanism by DFT calculations have permitted to formulate a quite probable catalytic cycle and support the observed enantioselection. So, the acidic ammonium ion [DIPEA−H]^+^ (derived from the organic base) activates the aldehyde carbonyl group towards addition of NHC catalyst, with concerted H‐transfer from the ammonium ion and C−C bond formation. Deprotonation of the alcohol so formed by the free base and oxidation of the released Breslow intermediate (H‐transfer to the oxygen of **DQ**) bring to acyl azolium **18**, taken to the chiral C4‐substituted DHPs by nucleophilic 1,2‐addition/elimination.

The stereoselectivity observed in the synthesis of C4‐substituted DHPs is most probably realized during the oxidation step: the geometry of the transition state associated to the pro‐(*R*) route is more stabilized *versus* the pro‐(*S*) one, owed to favourable π‐π stacking interactions involving *i)* the *N*‐aryl moiety of the Breslow intermediate and one phenyl group of **DQ**, and *ii)* the dihydropyridine ring and the 2,6‐dichlorophenyl residue.

It should be highlighted that the NHC‐catalyzed oxidative desymmetrization of 1,4‐DHPs turned out to work with *S*‐ and *N*‐nucleophiles, specifically, ethanethiol (EtSH) and trimethylsilyl azide (TMSN_3_), however lower efficiency was observed (EtSH: 64 % yield, 79 % ee; TMSN_3_: 40 % yield, 39 % ee).

#### 2.1.2 O_2_ (air)‐assisted processes

**Scheme 25:**
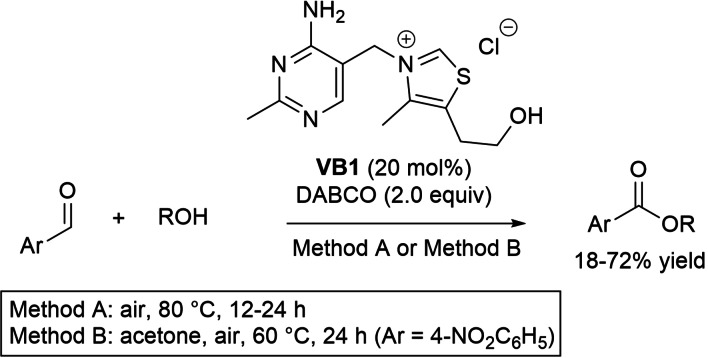
Aerobic oxidative esterification of aldehydes with alcohols catalyzed by **VB1**.

Acyl azolium intermediates for 1,2‐addition reactions with *O*‐ or *S*‐nucleophiles were catalytically formed by means of oxygen used as such or in combination with ETMs. Thus, aerobic oxidative esterification of aryl aldehydes with benzylic, heterocyclic, alkyl, and allyl alcohols was attained with vitamin B1 (thiamine, **VB1**) to furnish a range of ester derivatives in yields of 18–72 % (Scheme 25), an equally effective outcome being observed in a model scale‐up experiment (10 mmol of 4‐nitrobenzaldehyde, 61 % yield).^[45]^ On the strength of control experiment, the aldehyde‐to‐acyl azolium route has been confirmed, then followed by *O*‐acylation of the alcohol counterpart.

**Scheme 26:**
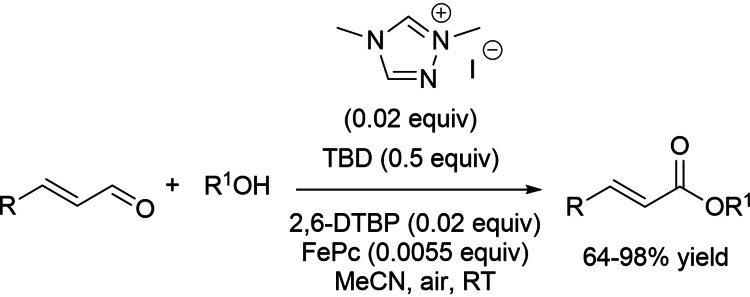
Aerobic oxidative esterification of enals with alcohols by NHC/ETMs system.

In 2016, Sundén group introduced aerobic NHC‐catalysis coupled to ETMs system for the oxidative esterification of α,β‐unsaturated aldehydes.^[46]^ The perfect catalytic system was formed by dimethyl triazolium iodide (0.02 equiv.), TBD (0.5 equiv.), 2,6‐DTBP (0.02 equiv.), FePc (0.0055 equiv.), which allowed to get an assortment of cinnamate esters in good to excellent yields (64–98 %) (Scheme 26).

**Scheme 27:**
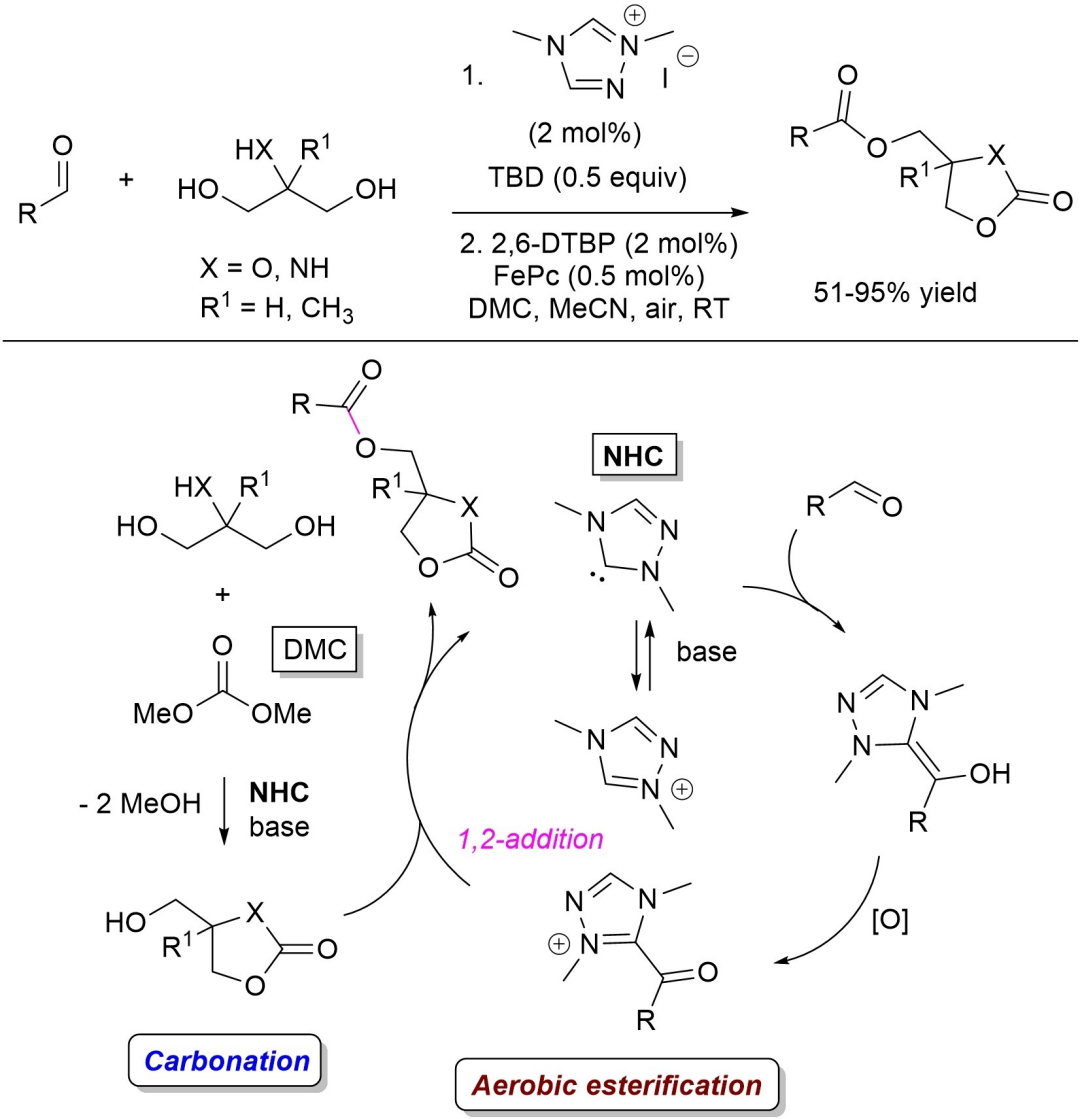
NHC‐catalyzed process for sequential carbonation and aerobic esterification of 1,3‐diols.

Based on this work, the same author has developed a NHC‐catalyzed telescopic approach for selective dual modification of 1,3‐diols (i. e., glycerol and 2‐amino‐2‐methyl‐propane‐1,3‐diol) through one‐pot combination of carbonation and aerobic esterification (Scheme 27).^[47]^ A large family of glycerol carbonate esters and 2‐oxooxazolidine esters were prepared in good to excellent yields (51–95 %) starting from dimethyl carbonate (DMC), aliphatic/aromatic enals and (hetero)aryl aldehydes, in the presence of dimethyl triazolium iodide (2 mol%), TBD (0.5 equiv.), 2,6‐DTBP (2 mol%), FePc (0.5 mol%) and aerial oxygen. In this process, the NHC/TBD‐catalyzed carbonation step delivers the nucleophilic partner of the acyl azolium intermediate deriving from oxidation of the Breslow intermediate by the coupled system of ETMs.

**Scheme 28:**
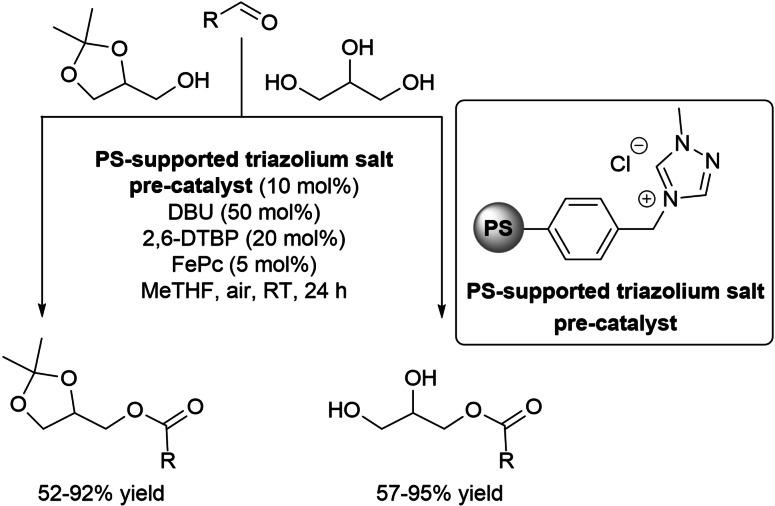
Aerobic oxidative esterification of glycerol and solketal by heterogeneous NHC‐catalysis.

Soon later, esterification of glycerol and its derivative solketal (1,2‐isopropylideneglycerol) under aerobic oxidative NHC‐catalysis has been leveraged to obtain monoacylglycerols (MAGs), with the aid of PS‐supported triazolium salt pre‐catalyst^[20]^ combined with DBU and 2,6‐DTBP/FePc pair (Scheme 28).^[48]^ High yield (57–95 %) and selectivity (>95 : 5 monoester/diester ratio) of MAGs could be reached in MeTHF, and very similar results arose from solketal, its esters being produced in 52–92 % yield. Anyway, the reactions benefited from a very broad substrate scope, including aromatic, α,β‐unsaturated, long chain aliphatic, and also biogenic (vanillin, citronellal) and biomass‐derived (FF, HMF) aldehydes.

Rewardingly, benchmark experiments under continuous‐flow regime (packed‐bed microreactor, reaction between glycerol and 1‐naphthaldehyde) gave disappointing findings (15 % conv.), probably owing to low oxygen concentration inside the reactor. However, use of air‐recyclable **DQ** overturned the result, optimized conditions (50 mol% DBU, 100 mol% oxidant, MeTHF, RT) getting the ester product with total conversion (>95 %) and full selectivity (monoester/diester >95 : 5). Just like, glycerol and solketal esters derived from FF, HMF, vanillin and citronellal were produced (>90 % conv., complete selectivity).

All these achievements have been the start of further studies on heterogeneous NHC‐catalyzed oxidative transformation of HMF into 5‐hydroxymethyl‐2‐furancarboxylic acid (HMFCA) and its derivatives.^[49]^ After screening different conditions, the best found system was the one combining PS‐supported triazolium pre‐catalyst and DBU with atmospheric air and solely FePc (MeTHF solvent), the low energy barrier of the latter (*E*=+0.74 V vs. SCE)^[50]^ favouring the oxidative pathway thanks to fast reaction with Breslow intermediate compared to oxygen.

**Scheme 29:**
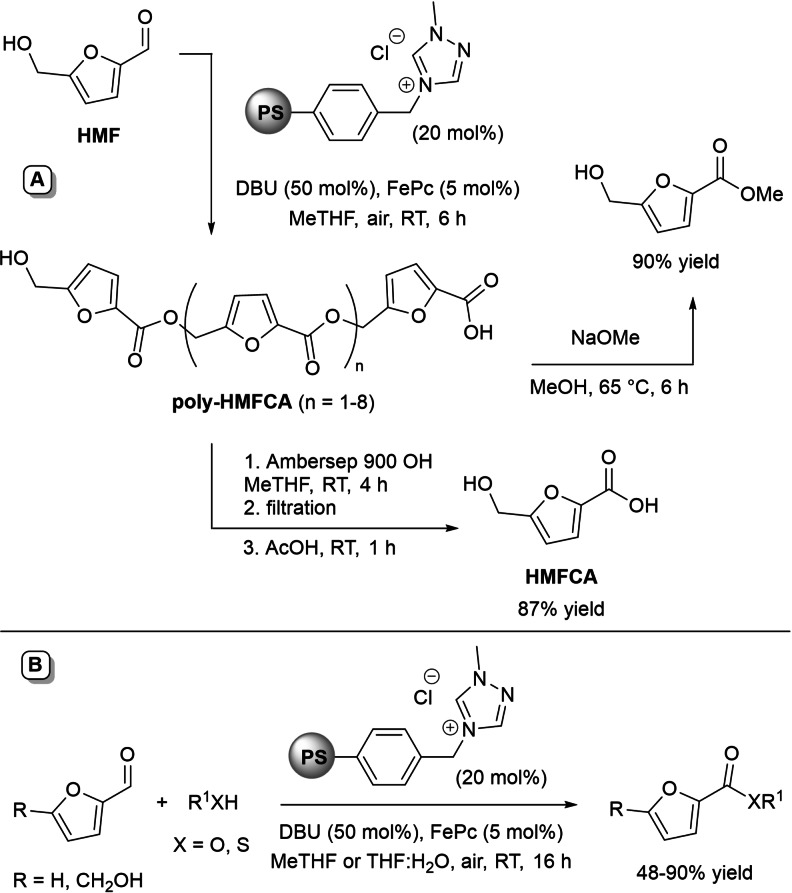
One‐pot two‐step approach to HMFCA and its methyl ester via aerobic oxidative NHC‐catalysis (**A**) and aerobic oxidation of FF and HMF under heterogeneous NHC‐catalysis (**B**).

The disclosed catalytic oxidation system has rendered possible the oxidative esterification of HMF to an oligomeric polyester (poly‐HMFCA), which took part in in situ sequential basic hydrolysis (ionic supported base) and acidification (“catch and release” technique) to produce HMFCA in 87 % overall yield (Scheme 29A). Moreover, HMFCA methyl ester (90 % yield) could be obtained upon nucleophilic depolymerization of poly‐HMFCA with methanol.

In parallel, the heterogeneous NHC catalyst/FePc/air system permitted the direct conversion of HMF to the corresponding methyl and butyl esters (64 % and 62 % yield, respectively), provided that excess amount (5.0 equiv.) of *O*‐nucleophile was used to minimize concomitant polycondensation reactions. In like manner, furoic acid and its butyl ester have been derived from FF (90 % yield) (Scheme 29B).

Here it is worth noting that exchange of the *O*‐nucleophile with a sulphured one (EtSH) has led to access thioester derivatives of HMF and FF in reasonable 48 % and 52 % yield, given the oxidation of the thiol substrate. And again, attempted production of HMF and FF ester products under continuous‐flow was precluded using air as the terminal oxidant, successful results being possible only after replacing it with **DQ** (90 to >95 % conv.).

**Scheme 30:**
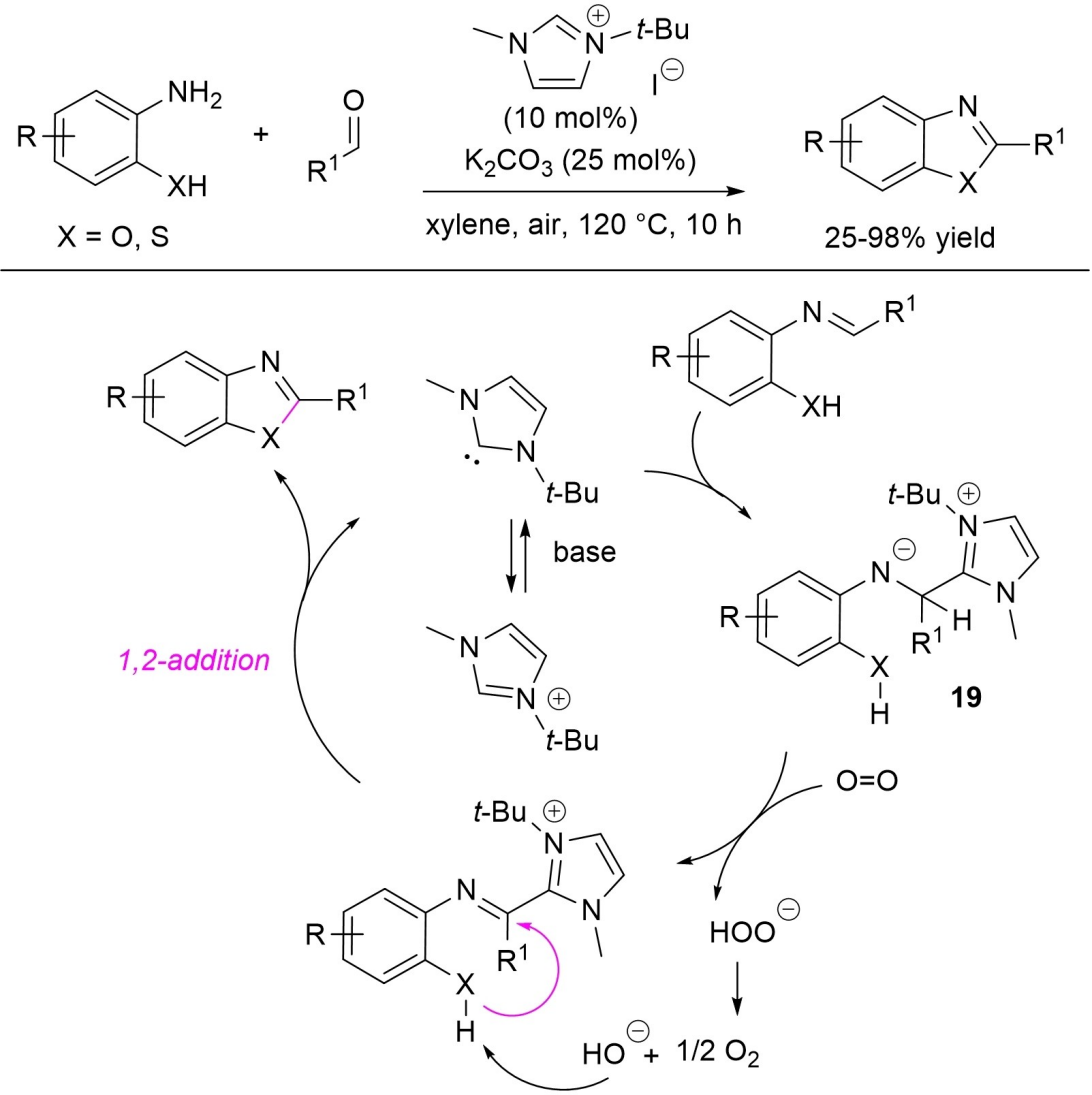
NHC‐promoted aerobic oxidative synthesis of 2‐substituted benzoxazoles/benzothiazoles.

NHC‐catalyzed one‐pot aerobic oxidative cyclization between aldehydes and 2‐aminophenols/2‐aminothiophenol has been gained by Hou and co‐workers using *N*‐*tert*‐butyl‐substituted imidazolium pre‐catalyst (10 mol%), K_2_CO_3_ (25 mol%), and air as the terminal oxidant (Scheme 30).^[51]^ This protocol has succeeded in synthesizing 2‐substituted benzoxazoles (25–98 % yield) and benzothiazoles (43–90 % yield) with ample substrate scope, including (hetero)aromatic/alkyl aldehydes and cinnamaldehyde. Furthermore, gram‐scale practicability (10 mmol, 70–84 % yield) was demonstrated under prolonged reaction time and/or O_2_ atmosphere.

A number of experiments indicated that there is the initial formation of a zwitterionic adduct **19** between NHC and the imine generated in situ from aldehyde and amine. **19** is then intercepted by O_2_ giving the imidoyl azolium intermediate and hydroperoxide anion, which decomposes to O_2_ and hydroxide anion. Deprotonation of hydroxy/thiol moiety and following intramolecular 1,2‐nucleophilic addition/fragmentation lead to forming the 2‐substituted heterocycles.

In 2020, Fu and Huang introduced NHC‐catalyzed aerobic oxidative reactions of alcohols with imines derived from (hetero)aromatic/α,β‐unsaturated aldehydes and varied heteroaryl amines, including 2‐aminobenzothiazole (and substituted analogues), 2‐aminothiazole, 2‐aminobenzimidazole, 2‐aminobenzoxazole.^[52]^

**Scheme 31:**
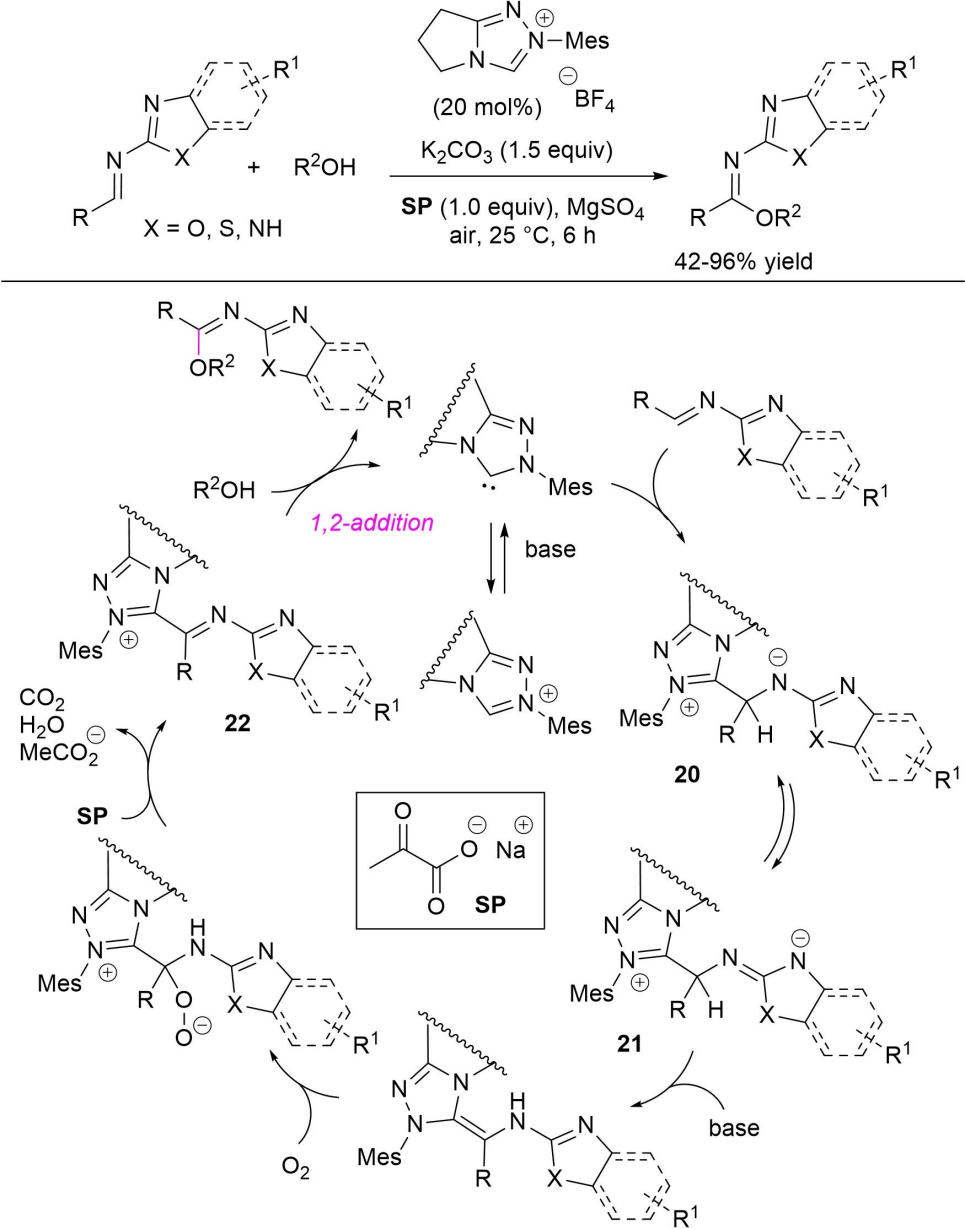
NHC‐promoted aerobic oxidative reactions of imines and alcohols with **SP** as peroxide scavenger.

This strategy has the special characteristic of harnessing sodium pyruvate (**SP**) as an unprecedented peroxide scavenger to force the catalytic cycle towards the oxidative route. Using ambient air as the unique oxidant, *N*‐mesityl pyrrolidine‐based triazolium pre‐catalyst (20 mol%), K_2_CO_3_ (1.5 equiv.) and equimolar **SP** in anhydrous conditions (MgSO_4_), a library of imidate compounds was prepared in moderate to excellent yields (42–96 %) (Scheme 31), and practicability of the method was shown by large‐scale reaction of methanol with the imine deriving from 2‐aminobenzothiazole and benzaldehyde (2 mmol, 68 % yield).

The mechanism formulated by the authors sees the formation of the NHC‐bound species **20**, which equilibrates to the dearomatized zwitterion **21**, having expectable increased acidity at the C−H bond bound to the NHC residue. Deprotonation of **21** leads to forming the *aza*‐Breslow intermediate, which adds to O_2_ to give a peroxidic species losing the peroxide moiety through the intervention of **SP**. This makes available imidoyl azolium **22** for the conclusive reaction with the alcohol nucleophile.

It can be added that **SP** has also been applied in aerobic oxidative esterification of aromatic aldehydes and cinnamaldehydes under similar experimental conditions (24 h reaction time), giving excellent yields of methyl ester products (66–96 % yield). On this point it is worth highlighting the worst results observed in lack of **SP** (15–66 % yield), supporting its key role in these transformations.

Very recent years have seen the emergence of dual NHC/photocatalysis as alternative strategy to classical approaches.^[53]^

With particular focus on oxidative NHC‐catalysis, an important contribution came from Ye and co‐workers, who realized the synthesis of aryl salicylates from *O*‐aryl salicylaldehydes via Smiles rearrangement,^[54]^ using oxygen as the terminal oxidant.^[55]^

**Scheme 32:**
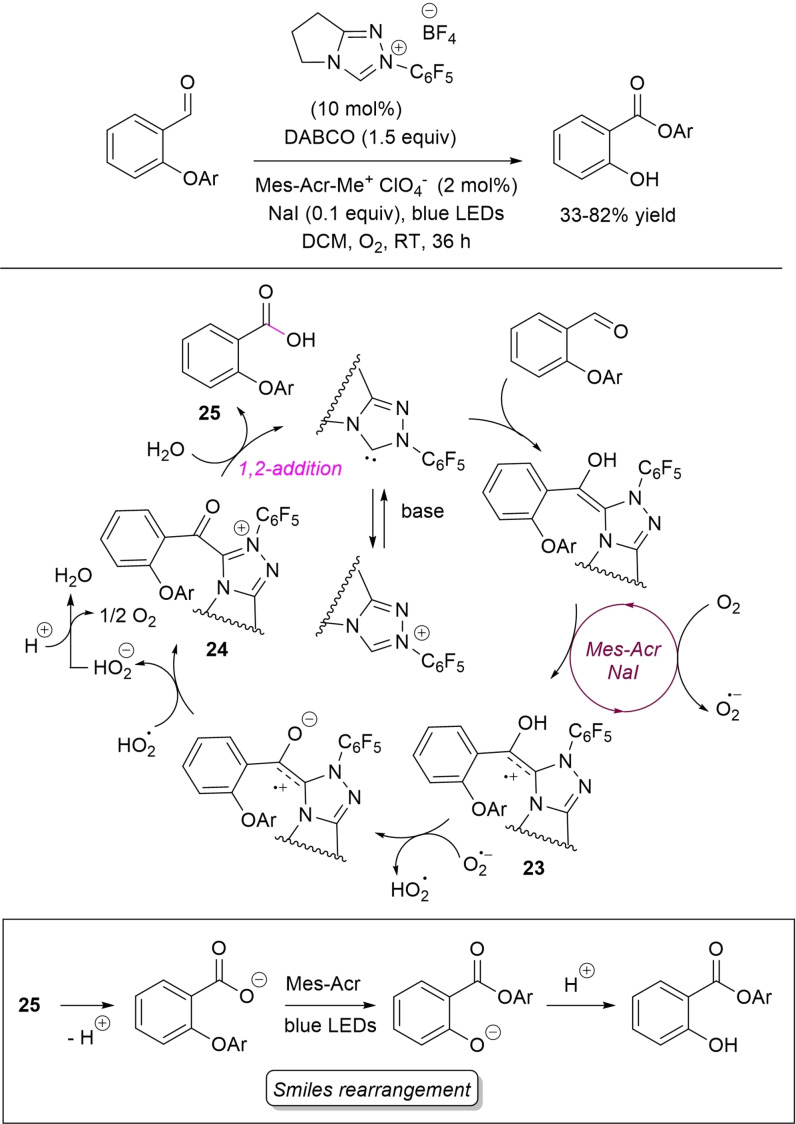
Synthesis of aryl salicylates from *O*‐aryl salicylaldehydes via cooperative NHC‐catalysis/photocatalysis.

*O*‐aryl‐4‐methylsalicylaldehydes, *O*‐tolyl‐4‐substituted salicylaldehydes (4‐MeO, 4‐Br, 4‐Cl), and *O*‐tolyl‐5‐methylsalicylaldehyde gave the corresponding rearranged products in moderate to good yields (33–82 %) by reaction with *N*‐pentafluorophenyl pyrrolidine‐based triazolium salt (10 mol%), DABCO (1.5 equiv.) and O_2_, together with 9‐mesityl‐10‐methyl‐acridin‐10‐ium perchlorate (Mes‐Acr‐Me^+^ClO_4_^−^) as the photocatalyst (blue LED irradiation) and NaI as an additive (Scheme 32).

In the light of mechanistic investigations, it may be assumed that a first oxidative NHC‐catalyzed pathway takes place to generate acyl azolium **24** via the radical cation **23**, in turn obtained from oxidation (SET) of the initially formed Breslow intermediate by O_2_/Mes‐Acr/NaI system. Next, hydrolysis of **24** closes the organocatalytic cycle resulting in the formation of *O*‐aryl salicylic acid **25**, key starting material for the successive photocatalyzed rearrangement.

**Scheme 33:**
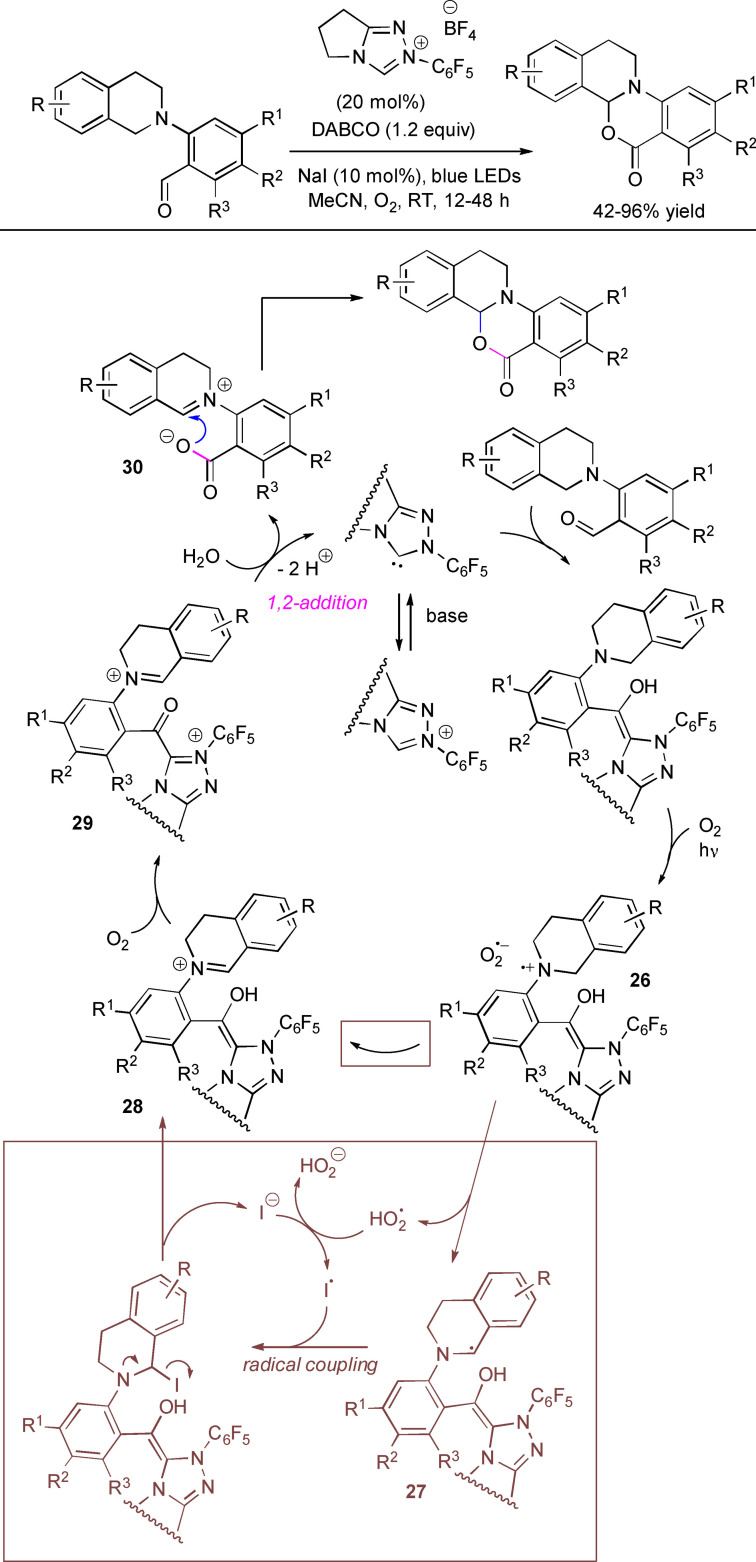
NHC‐catalyzed photooxidation through intramolecular CDC of tetrahydroisoquinoline‐based aldehydes.

Shortly after, the same authors extended the NHC‐catalyzed photo‐induced oxidative strategy to the intramolecular cross‐dehydrogenative coupling (CDC)^[56]^ of tetrahydroisoquinoline‐tethered aldehydes, with the advantage of avoiding the use of an external photocatalyst.^[57]^ More into detail, treatment of differently substituted tetrahydroisoquinoline‐derived benzaldehydes with *N*‐pentafluorophenyl pyrrolidine‐based triazolium salt (20 mol%), DABCO (1.2 equiv.), NaI (10 mol%) and O_2_ under blue LEDs gave the target cyclization products in 42–96 % yield (Scheme 33). Notably, gram‐scale reaction (5 mmol, 72 % yield) could be carried out by using half the pre‐catalyst loading (10 mol%) and equimolar amounts of base.

Control experiments and fluorescence spectra led the authors to postulate a plausible mechanism that builds upon SET oxidation of Breslow intermediate to amino radical cation **26**, followed by 1,2‐H shift and H‐abstraction by superoxide radical. The α‐amino radical **27** which is formed is then turned into an α‐iodoamine species (*iodide catalysis*), progenitor of iminium ion **28**, eventually oxidized to acyl azolium **29**. Its hydrolysis regenerates the NHC catalyst and likely forms a non‐isolated carboxylic acid that is instantly deprotonated to provide the iminium carboxylate **30**. The latter eventually participates in an intramolecular nucleophilic addition to afford the final product.

### 2.2 C−N bond formation

#### 2.2.1 External oxidant‐assisted processes

The transformation of aldehydes into amides (or amide‐like compounds) by *N*‐acylation under oxidative NHC‐catalysis is proven to be a valid option to conventional methods based on the use of carboxylic acids/derivatives as acylating agents, on account of greater practicability, which is expressed, among other issues, with mild reaction conditions, chemoselectivity, and no‐use of coupling reagents.

**Scheme 34:**
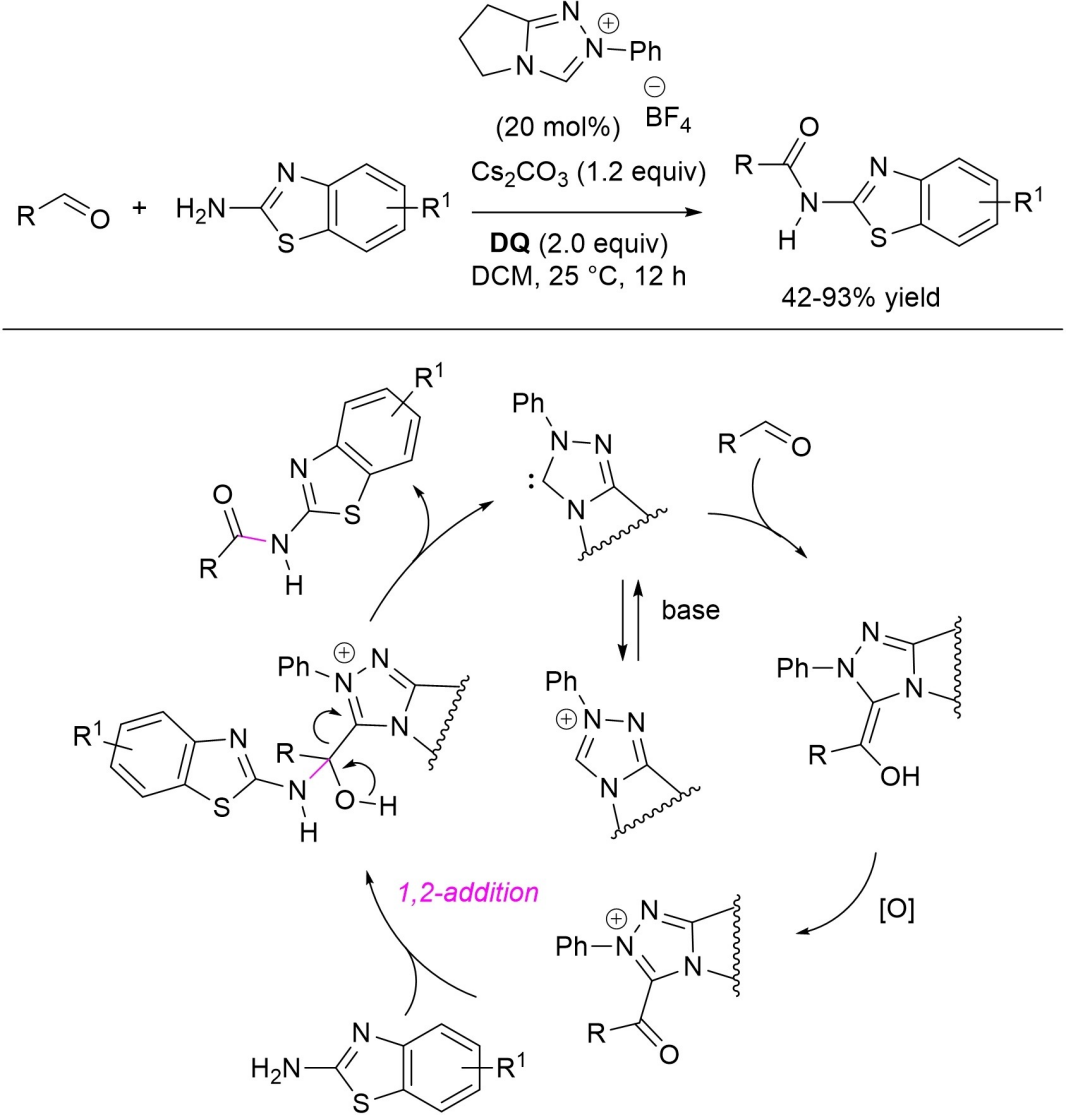
Direct oxidative amidation of aldehydes with 2‐aminobenzothiazoles.

Direct *N*‐acylation of aldehydes has been carried out using amines as the nucleophiles, but also unconventional counterparts (imines, amides) have been exploited. So, Biju and Yetra group communicated the oxidative amidation of 2‐aminobenzothiazoles with (hetero)aromatic/α,β‐unsaturated aldehydes and ferrocenecarboxaldehyde using *N*‐phenyl pyrrolidine‐based triazolium salt (20 mol%), Cs_2_CO_3_ (1.2 equiv.) and **DQ** (2.0 equiv.) (Scheme 34),^[58]^ giving rise to a large array of *N*‐acyl‐2‐aminobenzothiazoles (42‐93 % yield) including a few biologically relevant analogues (anti‐infective/herbicidal, antioxidant/anticonvulsant, antitubercular, anti‐cancer, protein‐protein interaction inhibitors, ligands for nuclear hormone receptors). Detailed experiments have led the authors to propose a tentative organocatalytic cycle where 1,2‐addition of the *N*‐nucleophile onto the acyl azolium ion yields a key aminal intermediate releasing the amide product concurrently with the NHC catalyst.

**Scheme 35:**
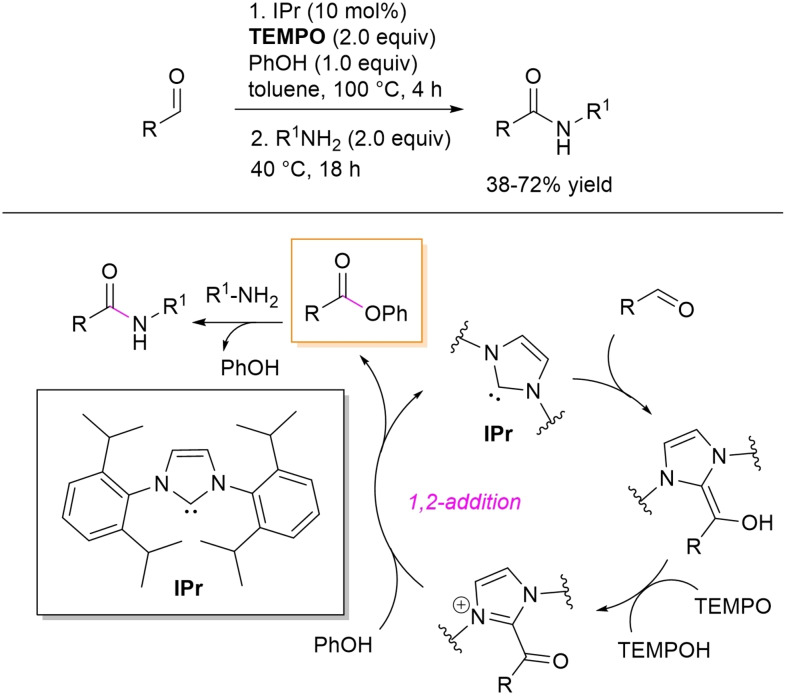
NHC‐catalyzed oxidative amidation of aldehydes via phenolic ester intermediates.

However, it must be said that NHC‐catalyzed direct aldehyde‐to‐amide conversion is very often impeded by competitive imine formation, which can be minimized (prevented) by a two‐step procedure via activated ester intermediates.[^19a^, ^59^] In this context, phenolic esters of cinnamaldehyde, benzaldehydes, heteroaromatic and aliphatic aldehydes were prepared by NHC‐catalyzed oxidative esterification using IPr catalyst and TEMPO oxidant, and coupled with a diverse range of primary amines, including allyl, aliphatic and heteroaromatic members, to afford amide compounds in modest to good yield (38–72 %) (Scheme 35).^[60]^ The phenolic ester is formed by the consolidated organocatalytic route (Breslow intermediate formation/oxidation), then followed by aminolysis to provide the secondary amide.

**Scheme 36:**
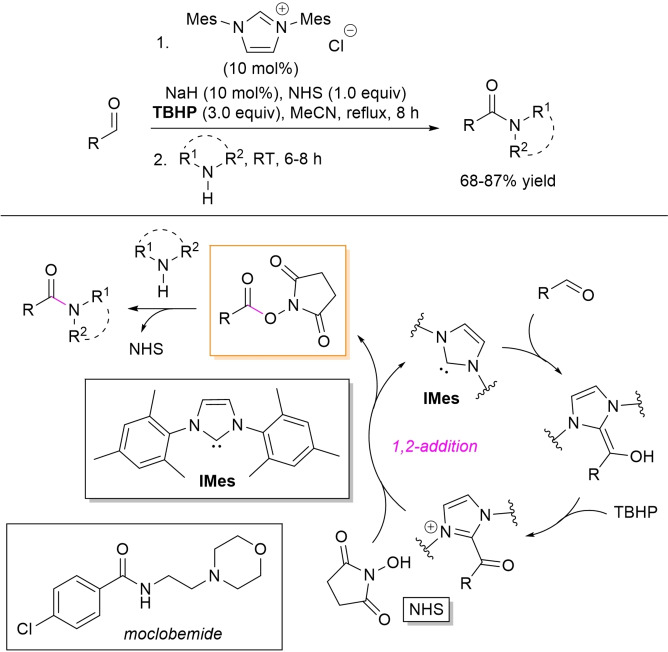
NHC‐catalyzed oxidative amidation of aldehydes via NHS esters.

Instead, access to both secondary and tertiary amides was realized by a one‐pot oxidative esterification/aminolysis protocol based on *N*‐hydroxysuccinimide (NHS) esters derived from alkyl and (hetero)aryl aldehydes, utilizing TBHP as the oxidant and IMes promoter, in turn generated from the parent azolium chloride (10 mol%) and NaH (10 mol%) (Scheme 36).^[61]^ Mono‐ and polysubstituted anilines, alkyl amines, and cyclic amines (morpholine, piperidine, pyrrolidine) gave very good results (68–87 % yield), with the anti‐depressant (MAO‐inhibitor) moclobemide obtained by combination of 4‐chlorobenzaldehyde and 2‐morpholinoethan‐1‐amine (87 % yield).

**Scheme 37:**
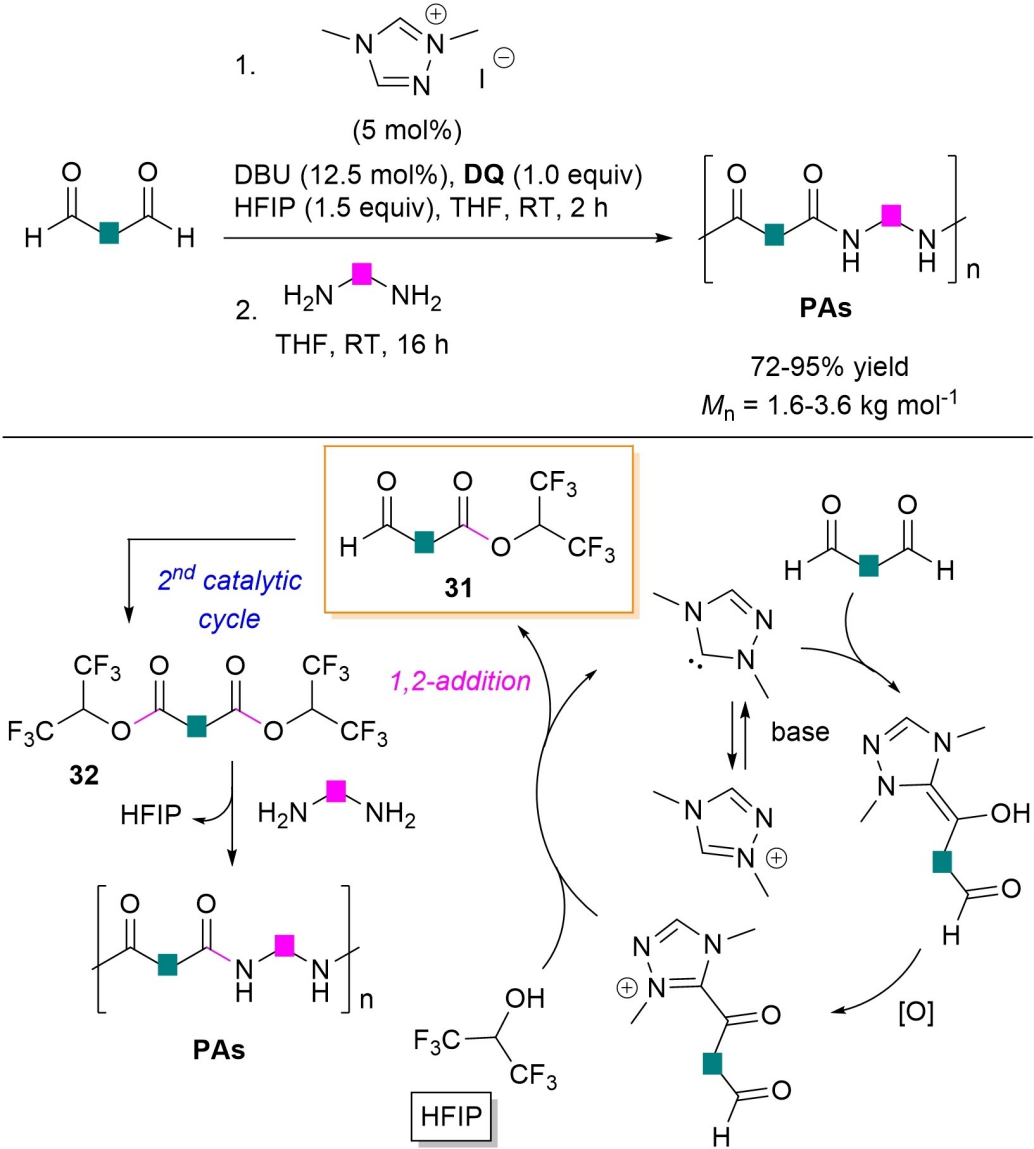
NHC‐catalyzed oxidative polyamidation of dialdehydes via hexafluoroisopropyl esters.

Recently, Massi and co‐workers took advantage of hexafluoroisopropyl esters as central species to make possible the NHC‐catalyzed polycondensation of diamines and dialdehydes for the assemblage of oligomeric polyamides (PAs).^[62]^ In this approach, the same reaction conditions used for the synthesis of PEs (Scheme 12)^[27]^ were slightly modified by adding hexafluoro‐2‐propanol (HFIP, 1.5 equiv.) to ensure ester formation (THF, 2 h), then followed by reaction with the diamine nucleophile (1.1 equiv., THF, 16 h), as detailed in Scheme 37. In such a way, it was possible to get poly(*p*‐ethylene terephthalamide) (*M*_n_=1.9 kg mol^−1^, 90 % yield) from ethylene diamine and terephthalaldehyde (even on gram‐scale: 10 mmol of dialdehyde, 92 % yield), and also a series of semi‐aromatic and fully aromatic PAs. These include terephthalaldehyde‐ and isophthalaldehyde‐derived PAs based on 1,10‐decanediamine (1,10‐DDA) and 1,6‐hexanediamine (1,6‐HDA) (*M*_n_=1.7–2.6 kg mol^−1^, 78–88 % yield), and bio‐based PAs (*M*_n_=1.6–3.6 kg mol^−1^, 72–95 % yield). The latter were produced by coupling the aliphatic 1,10‐DDA/1,6‐HDA with a furanic dialdehyde monomer (2,5‐diformylfuran) and bisfuranic 5,5’‐[oxybis(methylene)]bis[2‐furaldehyde], or starting from 2,5‐bis(aminomethyl)furan and terephthalaldehyde.

In accordance with Studer's mechanistic proposal^[59]^ and the consolidated mechanism of oxidative NHC‐catalysis, it has been assumed that a first catalytic cycle occurs where the acyl azolium intermediate is attacked by nucleophilic HFIP to form the hexafluoroisopropyl monoester **31**, with concomitant NHC release. Next, a second catalytic cycle involving the aldehyde moiety of **31** gives diester **32**, finally attacked by the diamine nucleophile to install the amide linkage and restore HFIP, iteration of this last step accounting for the formation of PAs.

**Scheme 38:**
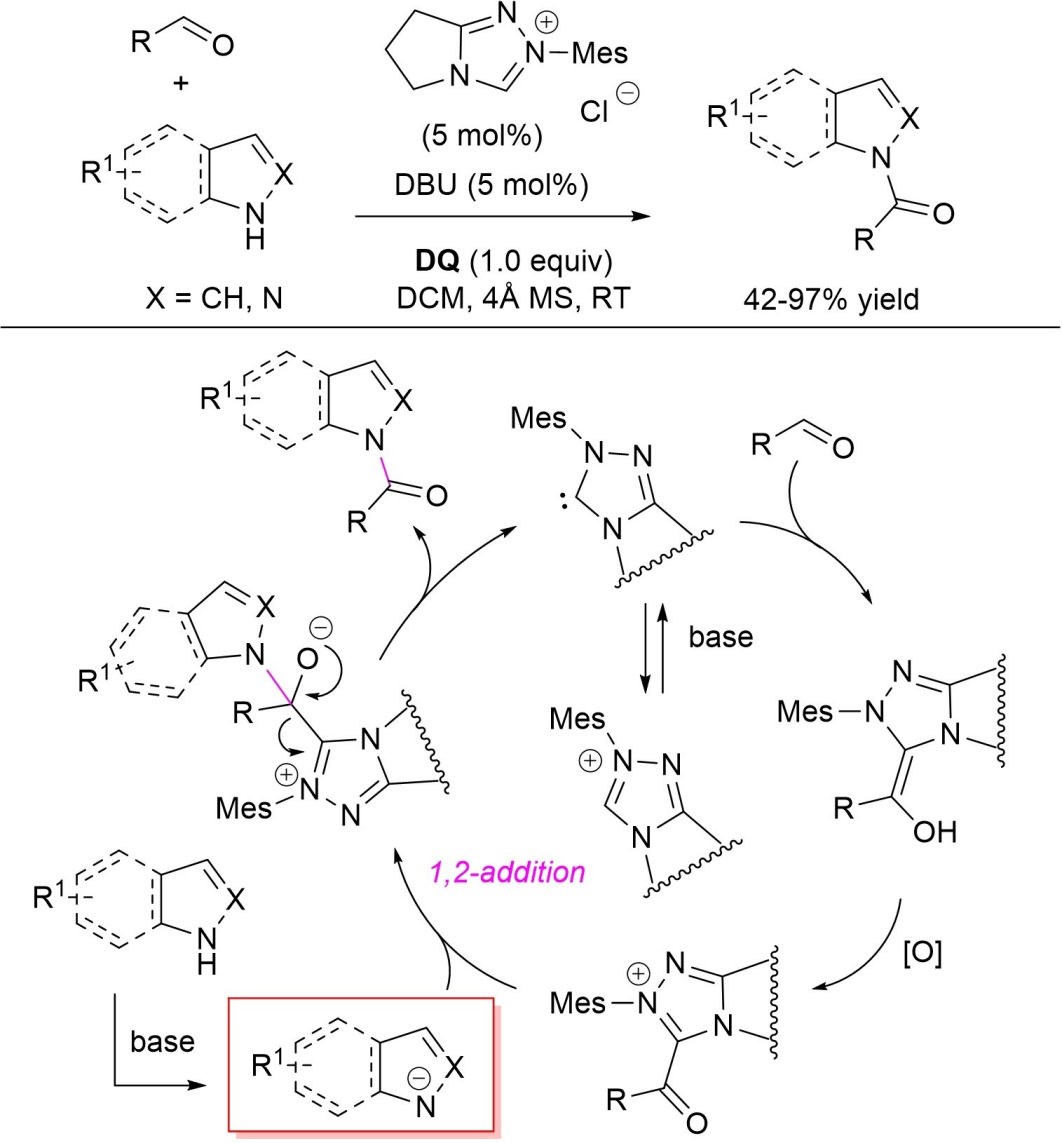
NHC‐promoted *N*‐acylation of heterocycles with aldehydes under oxidative conditions.

Due to the biological relevance of *N*‐acylated heterocycles, much efforts have been spent into their preparation through NHC‐catalyzed oxidative processes. In this connection, selective N−H amidation of indoles, pyrroles and indazoles with aldehydes has been developed using pyrrolidine‐based *N*‐mesityl triazolium salt (5 mol%) and DBU base (1.0 equiv.), in the presence of stoichiometric **DQ** as the external oxidant (Scheme 38).^[63]^

Wide‐ranging substrate scope and functional group compatibility were evidenced for both the acylating (aldehyde) and the heterocyclic substrate, benzaldehydes and α,β‐unsaturated/aliphatic conjugated aldehydes being converted into the corresponding amide congeners in good to excellent yields (42–97 %). And what is also important is the high chemoselectivity of the reactions, as concurrent C−H amidation was avoided altogether.

From a mechanistic point of view, it is expected that the crucial event is the nucleophilic addition of the deprotonated *N*‐heterocycle to the in situ formed acyl azolium ion, eventually followed by elimination of NHC catalyst.

It is worth noticing that a possible alternative aerobic route (aerobic oxygen as the terminal oxidant) has been explored to ameliorate the *E*‐factor of the process, stoichiometric **DQ** being replaced with the ETMs system formed by **DQ** (25 mol%) and FePc (3 mol%) in the benchmark reaction between indole and *trans*‐4‐(*N*,*N*‐dimethyl)cinnamaldehyde (90 % yield).

**Scheme 39:**
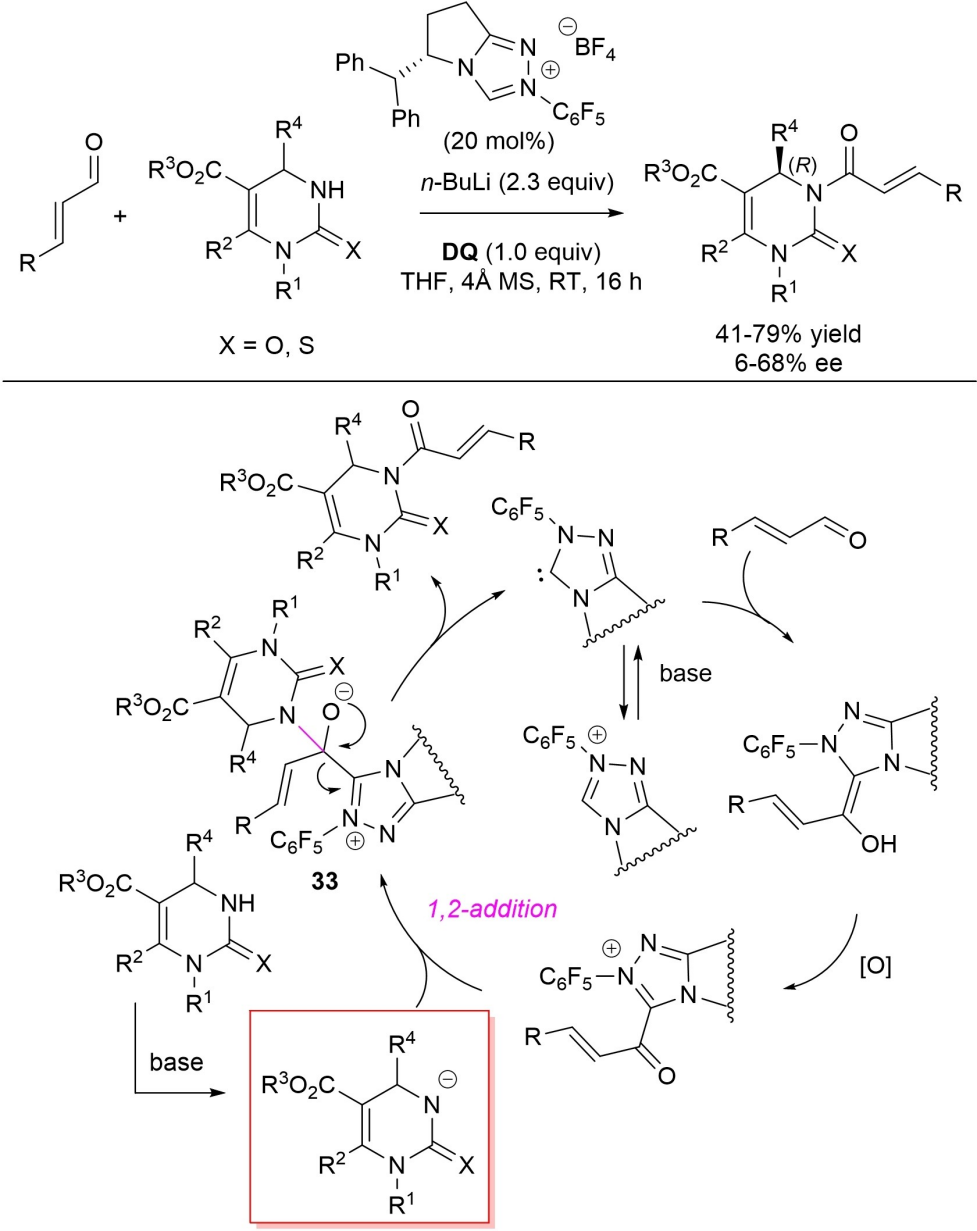
Enantioselective *N*‐acylation of DHPMs by oxidative NHC‐catalysis.

Cinnamaldehydes have been profitably employed as acylating partners of racemic 3,4‐dihydropyrimidin‐2‐(1*H*)‐ones (Biginelli dihydropyrimidines, DHPMs) in asymmetric NHC‐catalyzed oxidative reactions, leading access to enantioenriched synthetically and pharmaceutically important N3‐acylated products.^[64]^ Under optimum conditions, pyrrolidine‐based chiral triazolium pre‐catalyst (20 mol%) was used together with *n*‐BuLi (2.3 equiv.) and **DQ** (1.0 equiv.) to produce (*R*)‐configured amide‐like compounds with moderate to good enantioselectivity (6–68 % ee) (Scheme 39). It is important to stress that this protocol was very tolerant to variation of substituents at C4 (alkyl, aryl, heteroaryl), C5 (CO_2_Me, CO_2_Et) and N1 (Me, Ph, Bn), and could be also extended to a thio‐DHPM derivative (79 % yield, 16 % ee).

A feasible mechanism is accounted for by formation of α,β‐unsaturated acyl azolium intermediate, then nucleophilic 1,2‐addition of the deprotonated DHPM follows with formation of the target compound and catalyst turnover through intermediate **33**.

**Scheme 40:**
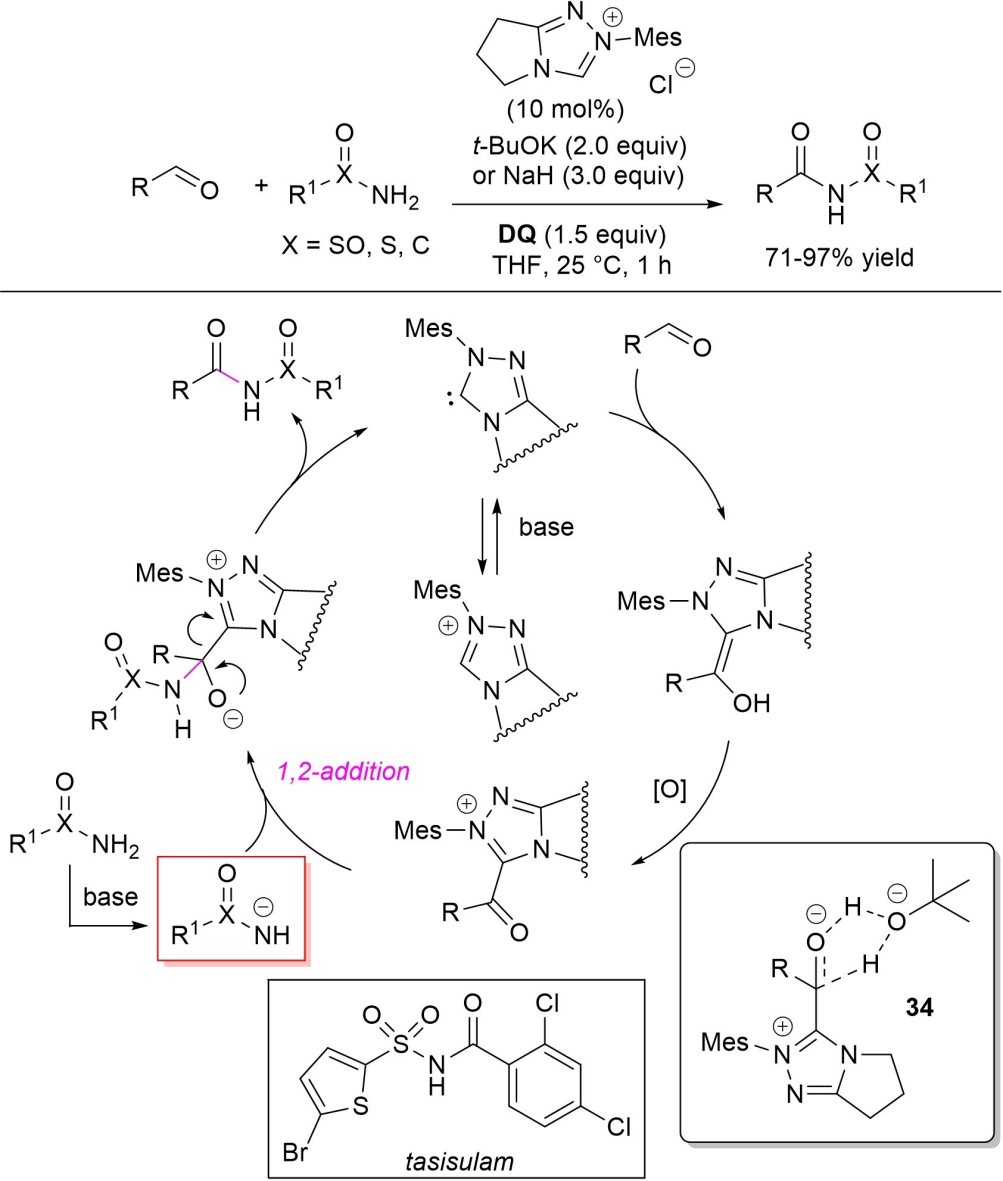
*N*‐acylation of primary amides with aldehydes by oxidative NHC‐catalysis.

The concept of using the conjugate base of a low nucleophilic species as the counterpart of an acyl azolium ion has been further exploited in the direct *N*‐acylation of amides by oxidative NHC‐catalysis. On such a basis, *N*‐sulfonylcarboxamides, *N*‐sulfinylcarboxamides, and dicarboxyimides have been derived from the parent primary amides and (hetero)aryl/α,β‐unsaturated aldehydes by action of the NHC arising from *N*‐mesityl pyrrolidine‐fused triazolium salt (10 mol%) and *t*‐BuOK (2.0 equiv.) or NaH (3.0 equiv.), always with **DQ** oxidant (1.5 equiv.) (Scheme 40).^[65]^ Interestingly, this system proved suitable to multigram synthesis of the antitumor agent tasisulam (1.09 g, 87 % yield) starting from 5‐bromothiophene‐2‐sulfonamide and 2,4‐dichlorobenzaldehyde.

It being understood that the catalytic cycle follows the general scheme repeatedly described, DFT studies on the model reaction between *p*‐chlorobenzaldehyde and *p*‐tolylsulfonamide have made it possible to clarify two key events, that is the deprotonation of the amide substrate and the formation of Breslow intermediate.^[66]^ With regard to the first of these, either *t*‐BuOK or the reduced form of the oxidant may be the possible bases involved, with no energy barrier, while the formation of Breslow intermediate through a concerted 1,2‐proton transfer is assisted by *t*‐BuOH via transition state **34**.

Further reference should be made to the acylation of NH‐sulfoximines with aldehydes, which was reported just a few months apart in 2016 by Bolm^[67]^ and Guin groups.^[68]^

**Scheme 41:**
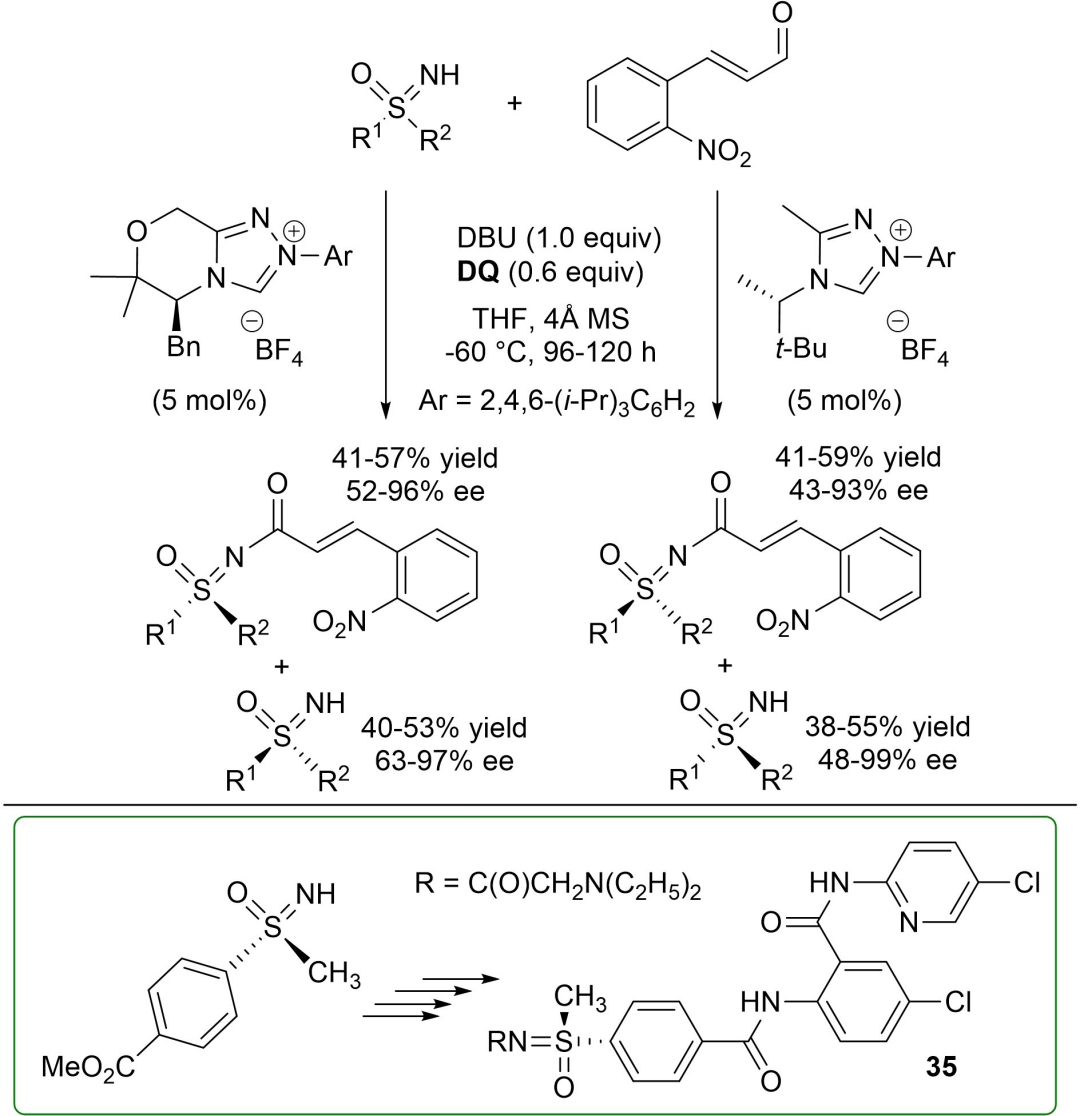
NHC‐catalyzed KR of sulfoximines via oxidative amidation.

Bolm and co‐workers have studied the NHC‐catalyzed oxidative amidation strategy as the means for attaining the kinetic resolution (KR) of racemic (hetero)aryl/alkyl substituted sulfoximines by reaction with 2‐nitrocinnamaldehyde in the presence of a chiral triazolium pre‐catalyst (*N*‐2,4,6‐triisopropylphenyl substituent, 5 mol%), DBU (1.0 equiv.) and **DQ** (0.6 equiv.) (Scheme 41).^[67]^ This protocol gave both enantiomers of the sulfoximines with fair to excellent enantioselectivities (48–99 % ee and 63–97 % ee, respectively), and was proven effective on a gram‐scale (5.6 mmol, 43 % yield, 90 % ee) for the KR of the sulfoximine bearing methyl and 4‐carbomethoxyphenyl substituents. Of importance is the application of the recovered recrystallized (+)‐enantiomer (95 % ee) in the asymmetric synthesis of the human Factor Xa inhibitor compound **35** (Scheme 41, green box).

**Scheme 42:**
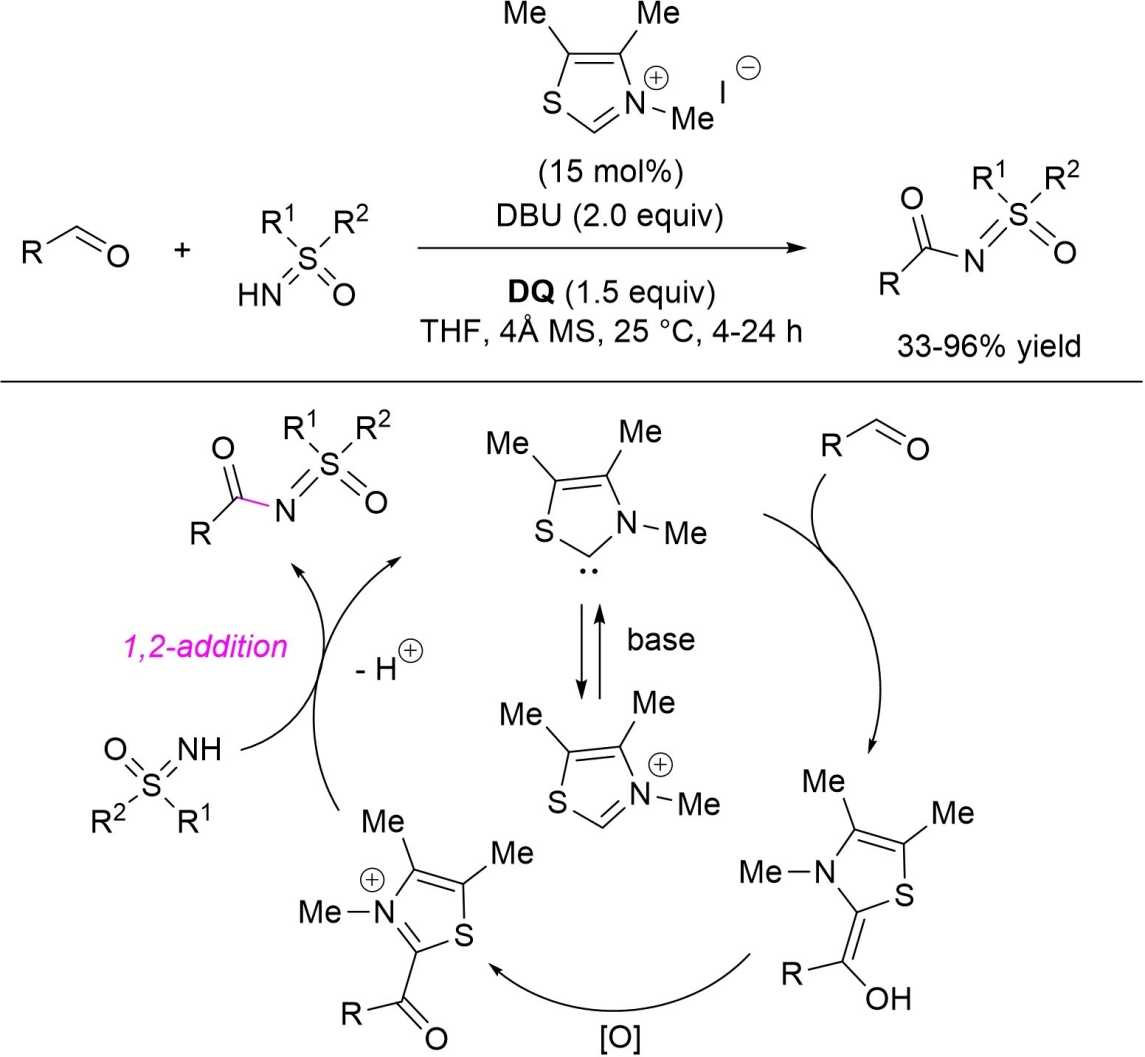
Oxidative *N*‐acylation of sulfoximines by NHC‐catalysis.

In the work of Guin and co‐workers, a vast library of *N*‐acylsulfoximines has been obtained in moderate to good yields (33–96 %) by reaction of (hetero)aromatic, α,β‐unsaturated, and aliphatic aldehydes with NH‐sulfoximines, catalyzed by a simple thiazolium salt (15 mol%)/DBU (2.0 equiv.) in alliance with **DQ** (1.5 equiv.) (Scheme 42), also on preparative scale (1.4–2.2 g of target product, 83–89 % yield).^[68]^ It has been proposed that the target compounds are formed by sequential acyl transfer from acyl azolium ion to NH‐sulfoximine and deprotonation.

#### 2.2.2 O_2_ (air)‐assisted processes

**Scheme 43:**
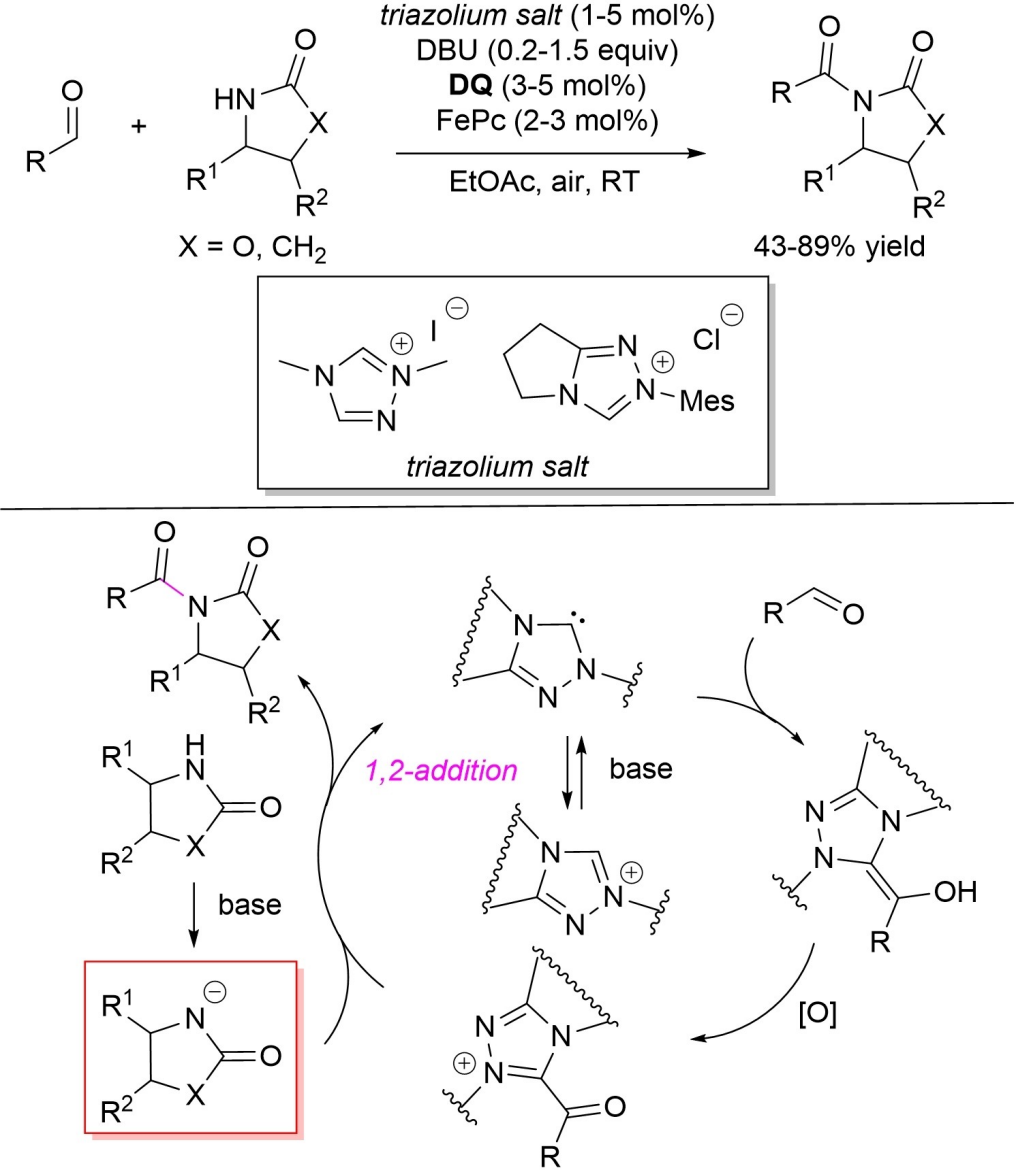
*N*‐acylation of oxazolidinones and pyrrolidinone by aerobic oxidative NHC‐catalysis.

In the field of aerobic oxidative NHC‐catalysis, *N*‐acylation of oxazolidinones and pyrrolidinone with aldehydes has been realized with the assistance of **DQ**/FePc combination as effective ETMs system (Scheme 43).^[69]^

The reaction showed ample generality with regard to both the acylating agent (aromatic/non aromatic enals, benzaldehydes, saturated aliphatic aldehydes) and the nucleophilic counterpart, 2‐oxazolidinone along with substituted achiral/chiral oxazolidinones working well to deliver the *N*‐acylated products in 43–89 % yield. Besides, it was possible to apply the synthetic strategy for coupling 2‐pyrrolidinone with cinnamaldehyde and 4‐methoxycinnamaldehyde producing two naturally occurring products, namely Piperlotine F (48 % yield)^[70]^ and the Nrf2 activator Piperlotine G (61 % yield),^[71]^ each in order.

It can be assumed that the deprotonated oxazolidinone (pyrrolidinone) is the species that actually intercepts the acyl azolium ion formed from Breslow intermediate by the O_2_‐assisted multistep electron transfer.

**Scheme 44:**
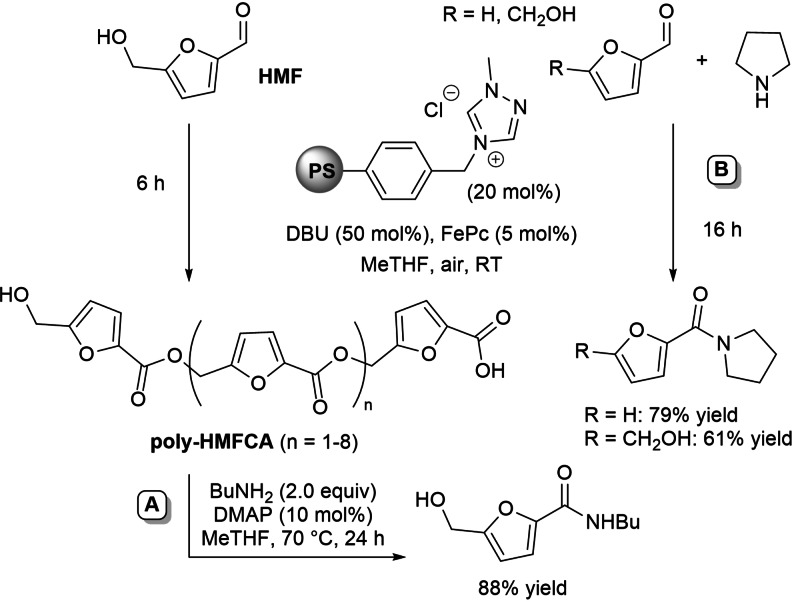
One‐pot two‐step approach to HMFCA amide (**A**) and direct amidation of HMF/FF (**B**) via aerobic oxidative NHC‐catalysis.

The heterogeneous NHC‐catalyzed aerobic oxidative strategy (acyl azolium intermediate) which applied in the preparation of HMFCA (thio)esters (Scheme 29)^[49]^ has been extended to arrive at the synthesis of the corresponding amide derivatives, both through the one‐pot two‐step protocol passing through poly‐HMFCA and the direct amidation of HMF (Scheme 44). In the one case, in situ formed poly‐HMFCA was directly treated with butylamine to give the corresponding secondary amide (Scheme 44A), on the other hand pyrrolidine was used as the nucleophile in the NHC‐catalyzed reaction of HMF promoted by FePc/air, with a similar transformation effected on FF (Scheme 44B).

**Scheme 45:**
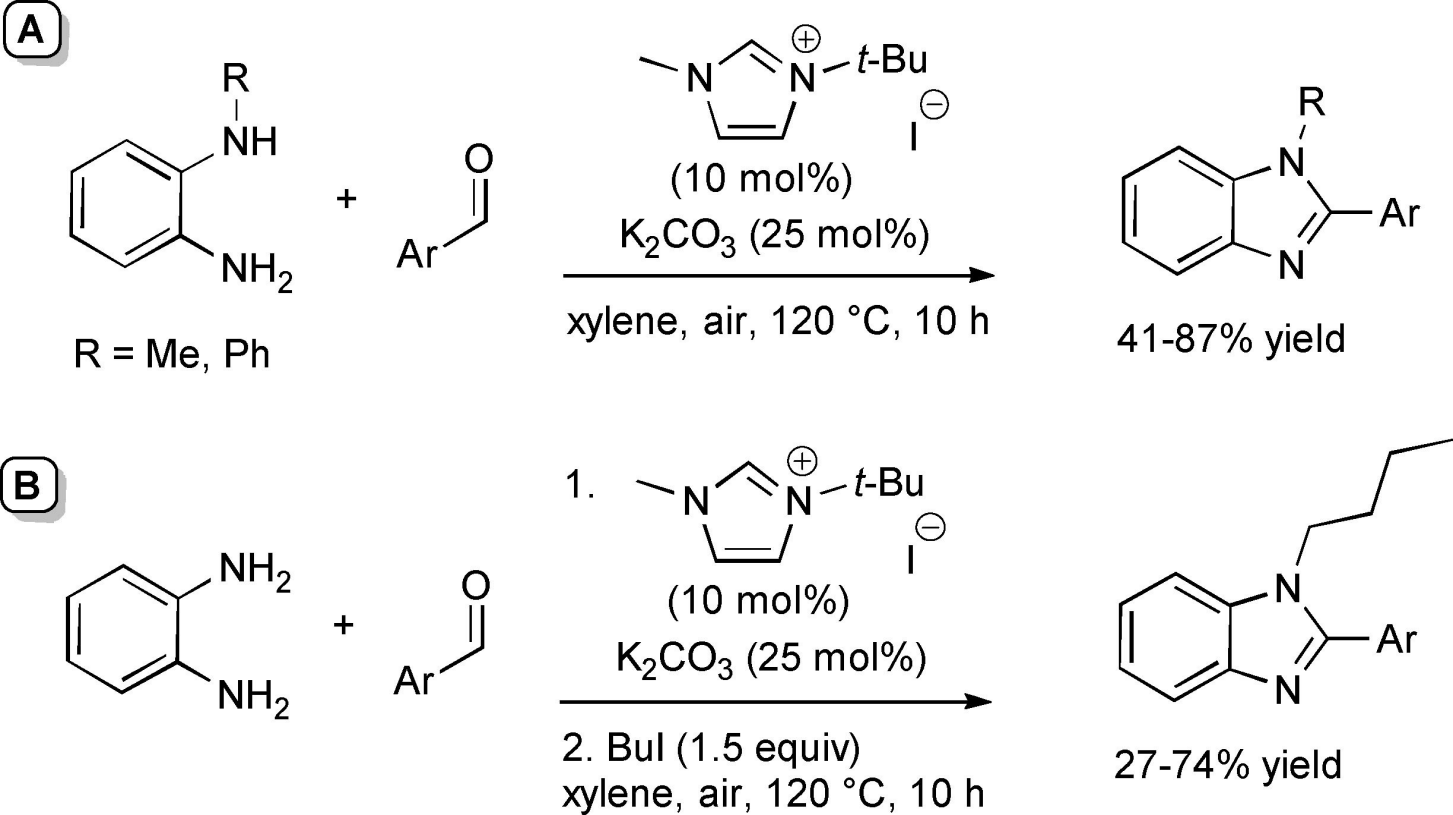
NHC‐promoted aerobic oxidative synthesis of 1,2‐disubstituted benzimidazoles.

The aerobic oxidative NHC‐catalyzed one‐pot methodology developed for the synthesis of 2‐substituted benzoxazoles and benzothiazoles via imidoyl azolium intermediate (Scheme 30)^[51]^ has found further application in the preparation of 1,2‐disubstituted benzimidazoles from monoalkylated *o*‐phenylenediamine and substituted benzaldehydes (Scheme 45A). Significant note is that this transformation was also devised as a one‐pot three‐component approach involving a combination of *o*‐phenylenediamine, (hetero)aromatic aldehyde and 1‐iodobutane, the latter promoting in situ formation of the necessary *N*‐alkylated *o*‐diaminobenzene unit (Scheme 45B).

**Scheme 46:**
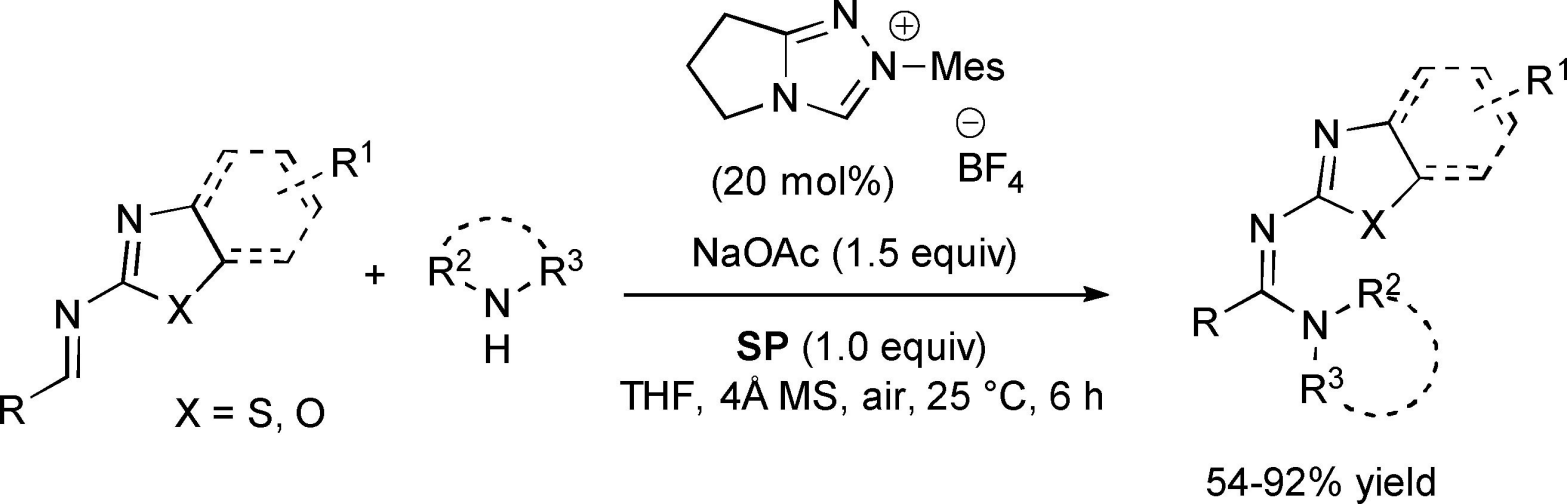
NHC‐promoted aerobic oxidative reactions of imines and amines with **SP** as peroxide scavenger.

Investigation of NHC‐catalyzed aerobic oxidative reactions of imines assisted by **SP** (imidoyl azolium intermediate, Scheme 31)^[52]^ has brought to identify amines (primary alkyl, cyclic secondary, anilines) as alternative nucleophiles to alcohols, opening the door for preparing a set of amidine derivatives in moderate to high yields (54–92 %) (Scheme 46).

## 3 α,β‐Unsaturated Acyl Azolium Intermediates

α,β‐Unsaturated acyl azoliums stemming from NHC‐catalyzed activation of enals and ynals under oxidative conditions have been much used as counterparts of dinucleophiles and/or three/four fold reactive reagents to prime cascade (domino) processes which comprise an initiating 1,4‐addition step (*Michael addition*) and a final intramolecular acylating (*1,2‐addition*) step. This brings to the construction of cyclic scaffolds through the formation of multiple bonds: some of the most common examples include *i)* dual bond formation, that is C−C/C−O bonds (Michael/lactonization, Michael/elimination/lactonization sequences), C−C/C−N, C−N/C−N and C−S/C−N bonds (Michael/lactamization sequence), and *ii)* three bond formation, namely C−C/C−C/C−O bonds (Michael/aldol/lactonization, Michael/Michael/lactonization sequences) and C−N/C−C/C−O bonds (Michael/aldol/lactonization sequence). This chapter gives some examples which are considered to be more representative of these transformations.

### 3.1 C−C/C−O bond formation (Michael/lactonization and Michael/elimination/lactonization sequences)

Lactone derivatives of the pyran series were built by way of NHC‐catalyzed oxidative reactions of α,β‐unsaturated aldehydes with benzofuran‐3‐ones, benzyl ketones and pyrrolin‐4‐ones through a Michael addition/lactonization route.

**Scheme 47:**
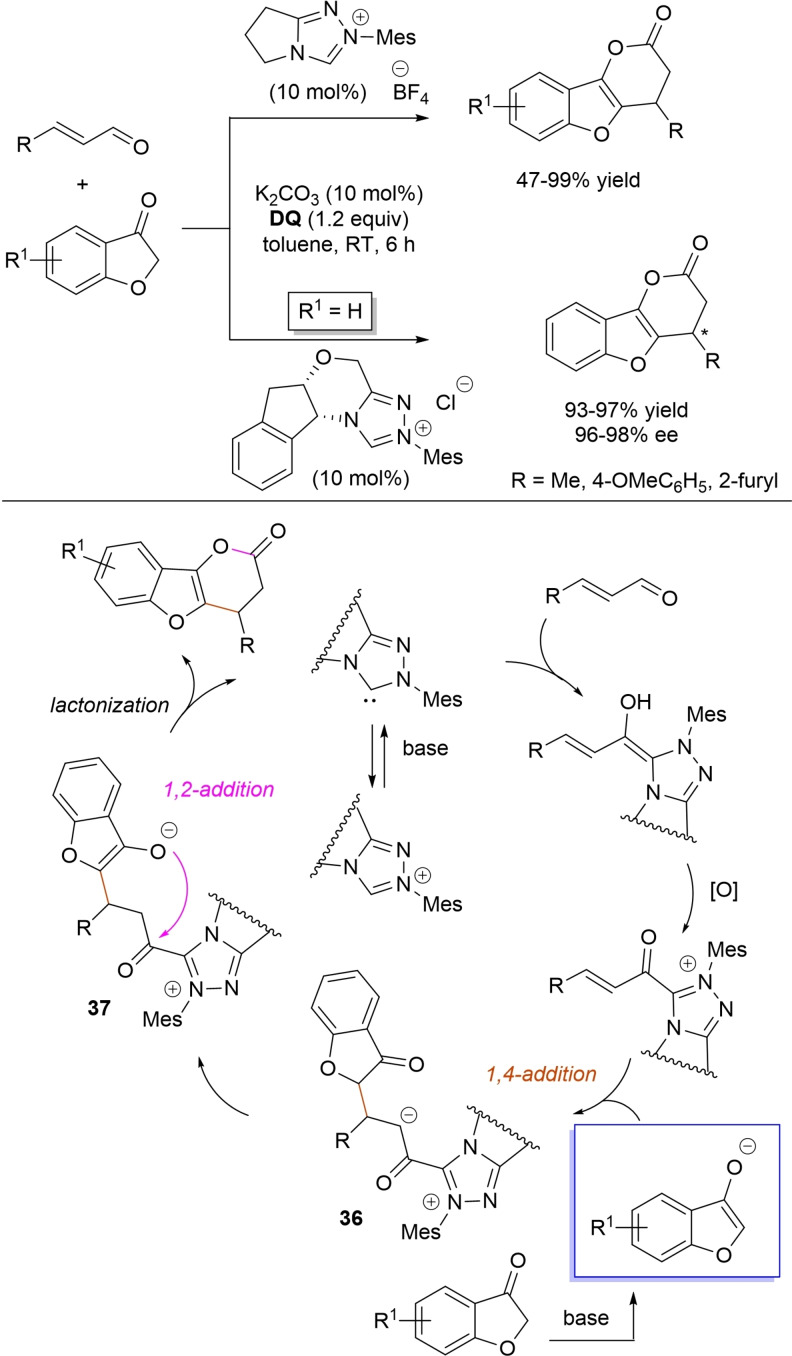
NHC‐catalyzed oxidative reaction of enals with benzofuran‐3‐ones.

Thus, benzofuran‐3‐ones have been coupled with (hetero)aryl‐ and alkyl‐substituted enals for the synthesis of benzofuran‐fused pyrones.^[72]^ Using *N*‐mesityl pyrrolidine‐based triazolium pre‐catalyst (10 mol%), K_2_CO_3_ (10 mol%) and **DQ** (1.2 equiv.), it was possible to obtain the target compounds in 47–99 % yield, with excellent enantiocontrol (96–98 % ee) ensured by moving to a chiral NHC catalyst (Scheme 47).

It is likely that the initially formed α,β‐unsaturated acyl azolium is intercepted by benzofuranone enolate giving rise to Michael adduct **36**, which isomerizes to enolate **37** by internal proton transfer. At last, intramolecular lactonization allows to assemble the tricyclic heterocyclic scaffold.

**Scheme 48:**
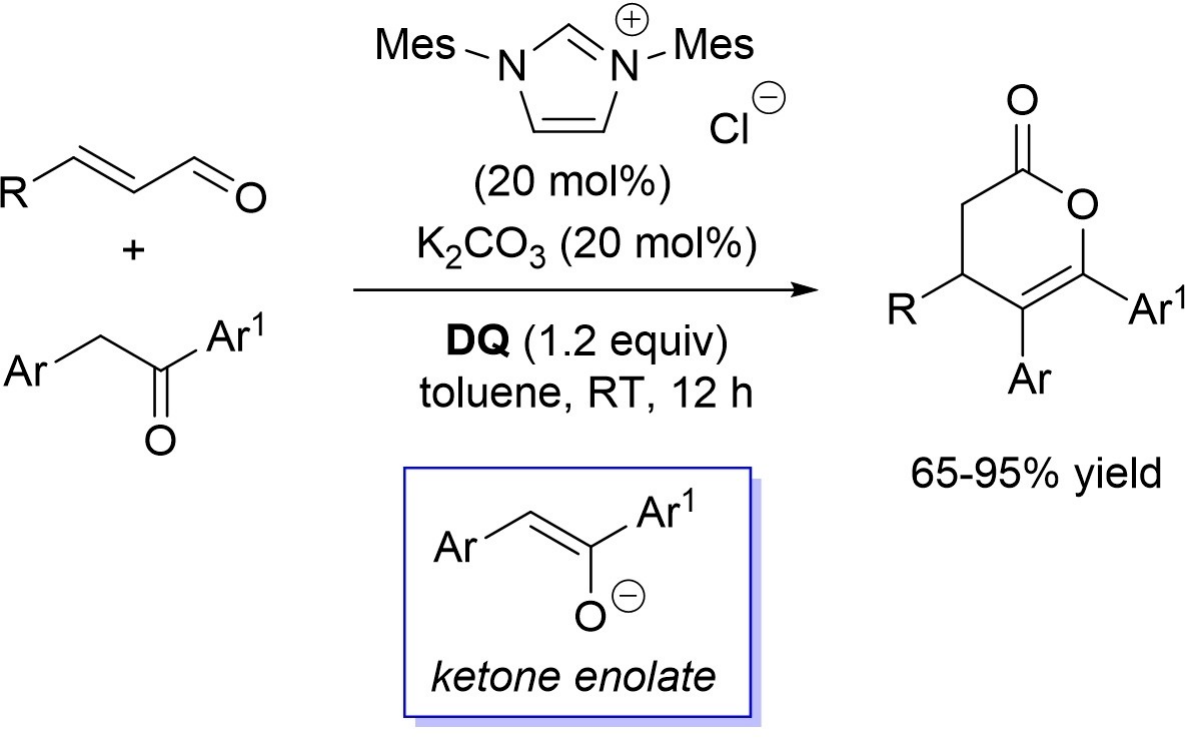
NHC‐catalyzed oxidative reaction of enals with benzyl ketones.

A very similar approach moved from benzyl ketones and α,β‐unsaturated aldehydes appended with (hetero)aromatic and long‐chain alkyl substituents.^[73]^ Good to excellent yields of 4,5,6‐trisubstituted dihydropyranones were obtained using 1,3‐dimesityl imidazolium salt (20 mol%) and K_2_CO_3_ (20 mol%), alongside **DQ** (1.2 equiv.) (Scheme 48). Applicability of this methodology on large‐scale should be highlighted, reaction of cinnamaldehyde (1.2 mmol) with benzyl phenyl ketone yielding 80 % of the annulated product.

Here too, conjugate addition of ketone enolate to the in situ formed α,β‐unsaturated acyl azolium followed by sequential proton transfer and lactonization should explain the construction of the intended product.

**Scheme 49:**
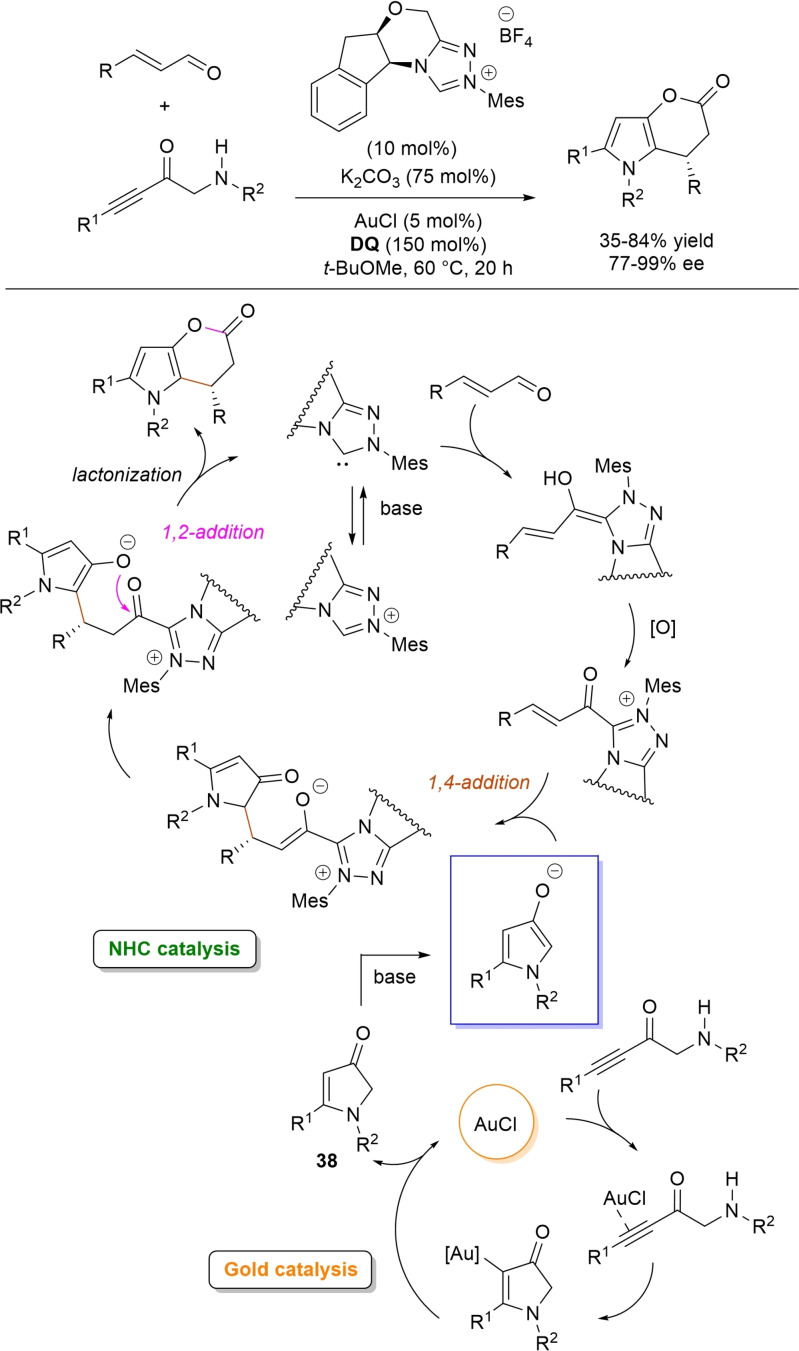
Synthesis of pyrrole‐fused lactones by gold and oxidative NHC catalysis.

Very recently, the merge of gold and oxidative NHC‐catalysis enabled pyrrolin‐4‐ones (obtained in situ from α‐amino‐ynones) to engage with enals to supply pyrrole‐fused lactones in high yield and excellent enantioselectivity.^[74]^ The experimental conditions have been optimized using AuCl (5 mol%), chiral aminoindanol‐based triazolium salt (10 mol%), K_2_CO_3_ (75 mol%) and **DQ** (150 mol%) (Scheme 49), many variations on enals (aryl, heteroaryl, vinyl, alkyl units in β‐position) and α‐amino‐ynones (H and phenyl/alkyl substituents on the sp terminal carbon, Cbz/Ts‐protected nitrogen) being well tolerated.

Based upon deep investigations, a mechanism was proposed which provides for the formation of pyrrolin‐4‐one **38** by Au (I)‐catalysis via sequential activation of α‐amino‐ynone, intramolecular cyclization and protonolysis. Next, deprotonation of **38** gives a doubly nucleophilic enolate that reacts with the enal‐derived α,β‐unsaturated acyl azolium via the usual Michael/lactonization sequence.

**Scheme 50:**
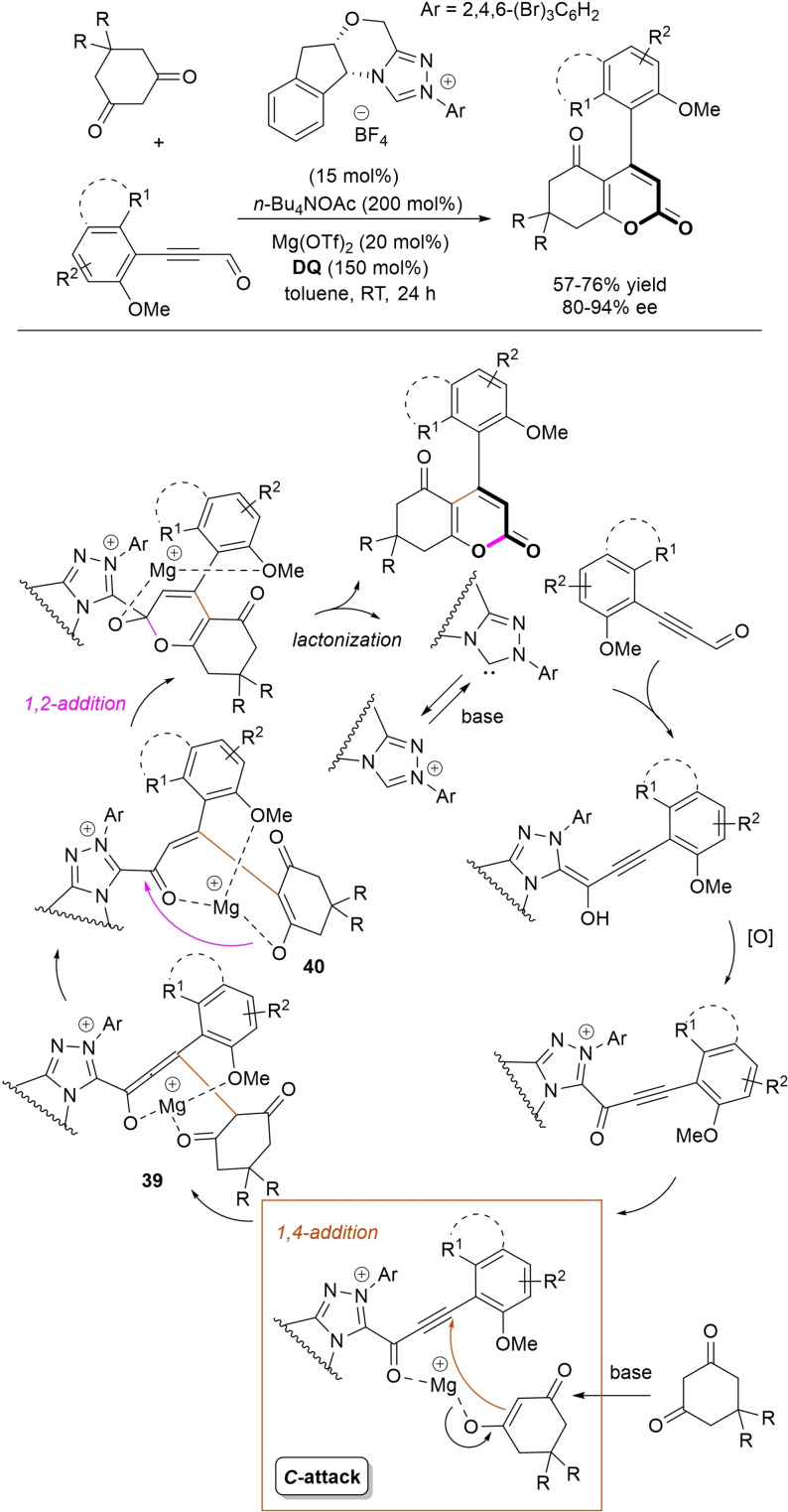
NHC‐catalyzed annulation of cyclic 1,3‐diones with ynals under oxidative conditions.

Oxidative NHC‐catalysis was put to the test in annulation reactions of cyclic 1,3‐diones with ynals, demonstrating that axially chiral α‐pyrone‐aryls could be obtained by the use of a chiral aminoindanol‐derived triazolium salt (2,4,6‐tribromophenyl *N*‐substituent, 15 mol%), *n*‐Bu_4_NOAc (200 mol%), **DQ** (150 mol%), and Lewis acid Mg(OTf)_2_ (20 mol%) as promoter (Scheme 50).^[75]^ In the annulation reactions of cyclic 1,3‐diones with ynals, alkyl/cycloalkyl substituted 1,3‐dione substrates and ynals with naphthyl/phenyl units worked well, furnishing moderate to good yields (57–76 %) of the finished products, with high enantioselectivities (80–94 % ee).

Arguably, ynal‐to‐alkynyl acyl azolium transformation via Breslow intermediate initiates the organocatalytic cycle, followed by Michael addition of the ketoenolate generated from 1,3‐dione under the basic conditions. The allenolate species **39** that forms passes through an intramolecular proton transfer, and the resulting α,β‐unsaturated acyl azolium **40** turns into the pyrone product by O−C bond formation and NHC undocking. This Michael addition/lactonization strategy is driven by the co‐present Lewis acid,^[76]^ as concurrent coordination of magnesium cation to the ketoenolate and the alkynyl acyl azolium favours the *C*‐attack over the *O*‐attack in the crucial 1,4‐addition step.

Michael addition/elimination/lactonization sequences were applied for the synthesis of α‐pyrones, too. For these specific cases, the choice fell on a pronucleophile component incorporating a group that served the dual purpose of stabilizing the in situ formed enolate and acting as a good (ionic) leaving group in the elimination step.

**Scheme 51:**
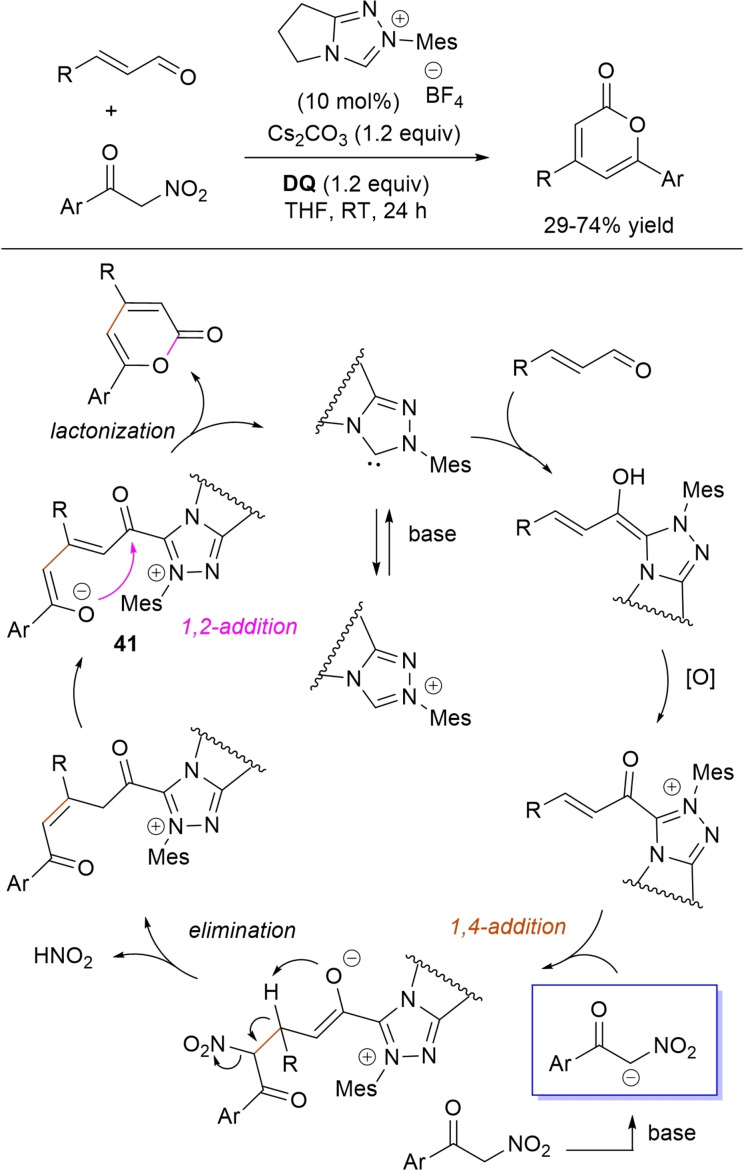
NHC‐catalyzed oxidative reaction of enals with aryl α‐nitro ketones.

Specifically, 4,6‐disubstituted α‐pyrones were obtained by Studer and Bera starting from aryl α‐nitro ketones and enals.^[77]^ The best reaction conditions called for the use of the NHC derived from *N*‐mesityl pyrrolidine‐based triazolium salt (10 mol%; Cs_2_CO_3_ as base, 1.2 equiv.) along with **DQ** oxidant (1.2 equiv.) (Scheme 51), achieving moderate to good yields (29–74 %).

The suggested mechanistic path involves the conjugate addition of deprotonated nitro compound to the α,β‐unsaturated acyl azolium intermediate, followed by HNO_2_ elimination. Subsequent deprotonation leads to the enolate **41** that then gives rise to lactonization.

**Scheme 52:**
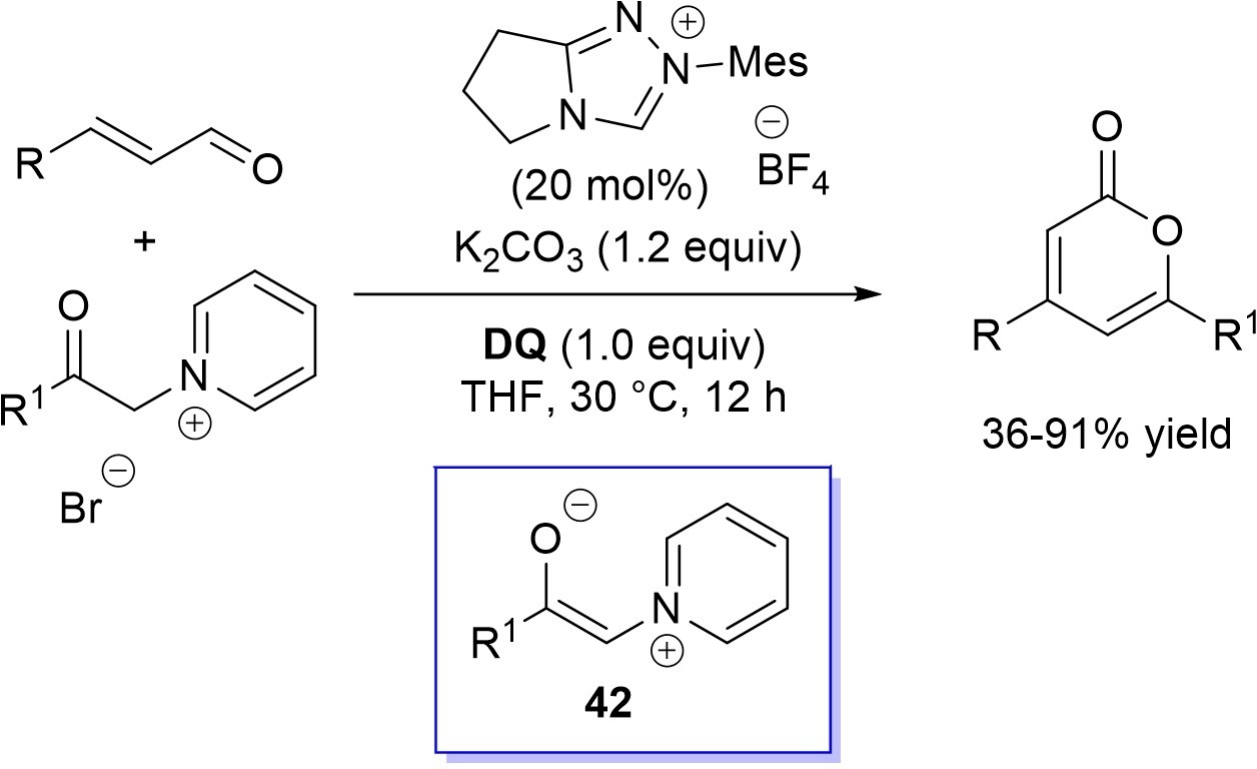
NHC‐catalyzed oxidative reaction of enals with nitrogen ylides.

A very similar organocatalytic system was tested for access to 4,6‐disubstituted α‐pyrones by reaction between aromatic/heteroaromatic enals and pyridinium bromide salts (Scheme 52),^[78]^ the latter becoming the ambident nucleophilic nitrogen ylides **42** triggering the domino Michael addition/elimination/lactonization.

### 3.2 C−C/C−N, C−N/C−N, C−S/C−N bond formation (Michael/lactamization sequence)

Use of 2‐(*N*‐arylhydrazono)acetates and 2‐(arylhydrazono)ketones as *C*‐nucleophiles towards aromatic and aliphatic α,β‐unsaturated aldehydes was applied to synthesize both enantioenriched 4,5‐dihydropyridazin‐3‐ones and pyridazin‐3‐ones under cooperative NHC/**DQ** catalysis, with catalytic and reaction conditions carefully regulated to favor the formation of one or the other compounds.^[79]^

**Scheme 53:**
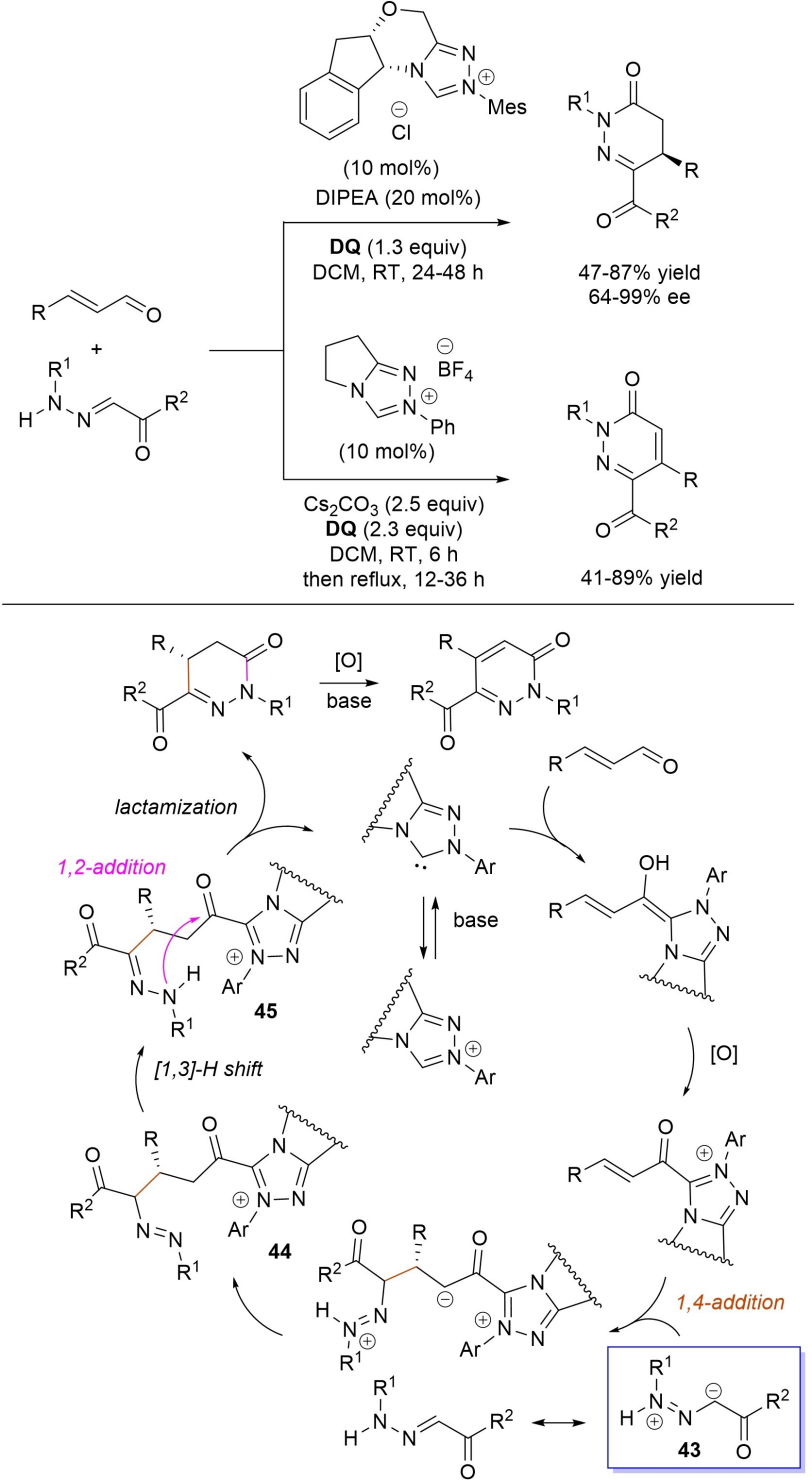
NHC‐catalyzed oxidative reaction of enals with hydrazones.

As depicted in Scheme 53, chiral aminoindanol‐derived triazolium salt (10 mol%), DIPEA (20 mol%) and **DQ** (1.3 equiv.) was the most efficient system for forming chiral 4,5‐dihydropyridazin‐3‐one derivatives (47–87 % yield, 64–99 % ee), while cooperation of achiral pyrrolidine‐fused triazolium salt (10 mol%), Cs_2_CO_3_ (2.5 equiv.) and **DQ** (2.3 equiv.) was beneficial for preparation of the oxidized pyridazin‐3‐one compounds (41–89 % yield). In this last case, dihydropyridazinones initially formed (RT, 6 h), then they were oxidized to the desired pyridazinones (solvent reflux).

It must certainly be said that the enantioselective process was the one that most depended on the nucleophilicity of the hydrazone component, both chemical yields and enantioselectivities being influenced by variation of the *N*‐substituent.

It was speculated that the in situ formed α,β‐unsaturated acyl azolium participates in a *carba*‐Michael addition (preferential *Re*‐face attack) from the hydrazone reagent via the resonance structure **43**, with generation of diazene species **44**. Afterwards, base‐promoted [1,3]‐H migration causes the formation of amino‐substituted imine **45**,^[80]^ which is implicated in the conclusive intramolecular *N*‐acylation/fragmentation (lactamization) leading to the 4,5‐dihydropyridazin‐3‐one scaffold. Its eventual base‐catalyzed oxidation accounts for the formation of the pyridazin‐3‐one derivative.

**Scheme 54:**
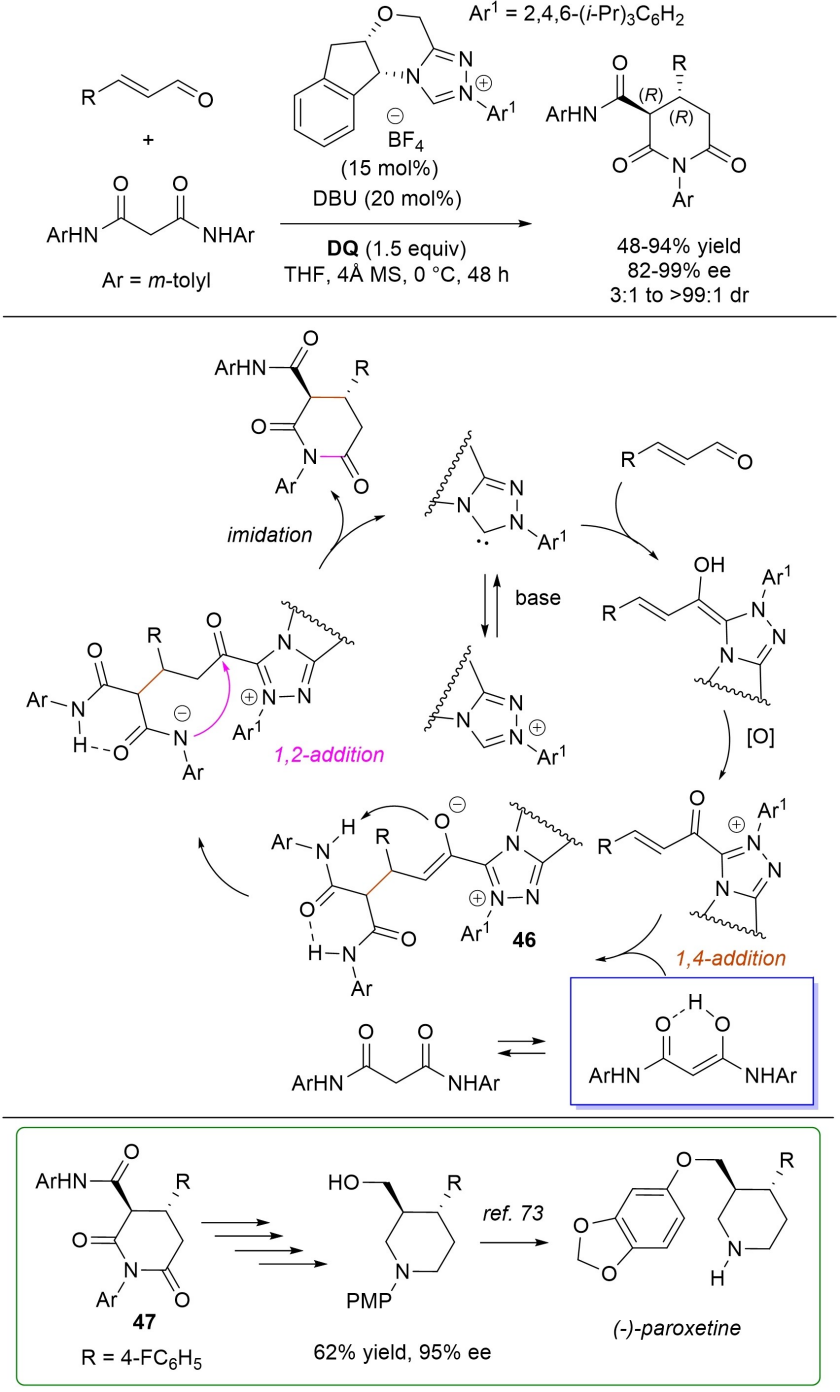
NHC‐catalyzed oxidative reaction of enals and *N* ^1^,*N* ^3^‐disubstituted malonamide, and formal synthesis of (‐)‐paroxetine.

*trans*‐3,4‐Disubstituted glutarimides were obtained in highly stereoselective fashion (82–99 % ee, 3 : 1 to >99 : 1 dr) starting with (hetero)aromatic/aliphatic enals and *N* ^1^,*N* ^3^‐di‐*m*‐tolylmalonamide, and utilizing chiral azolium pre‐catalyst with 1‐amino‐2‐indanol structure (*N*‐2,4,6‐triisopropylphenyl substituted, 15 mol%), DBU base (20 mol%) and **DQ** oxidant (1.5 equiv.) (Scheme 54).^[81]^

Formation of enolate **46** from the enal‐derived α,β‐unsaturated acyl azolium via conjugate addition of the enolizable diamide, internal proton transfer/tautomerization and imide formation are salient steps of the postulated organocatalytic cycle.

Notably, the glutarimide product **47** arising from *p*‐fluorocinnamaldehyde (1 g scale, 58 % yield, 87 % ee) represented a profitable building block for the formal synthesis of the biologically relevant (−)‐paroxetine (Scheme 54, green box) via the parent enantioenriched 3‐hydroxymethyl‐substituted piperidine (95 % ee).^[82]^

**Scheme 55:**
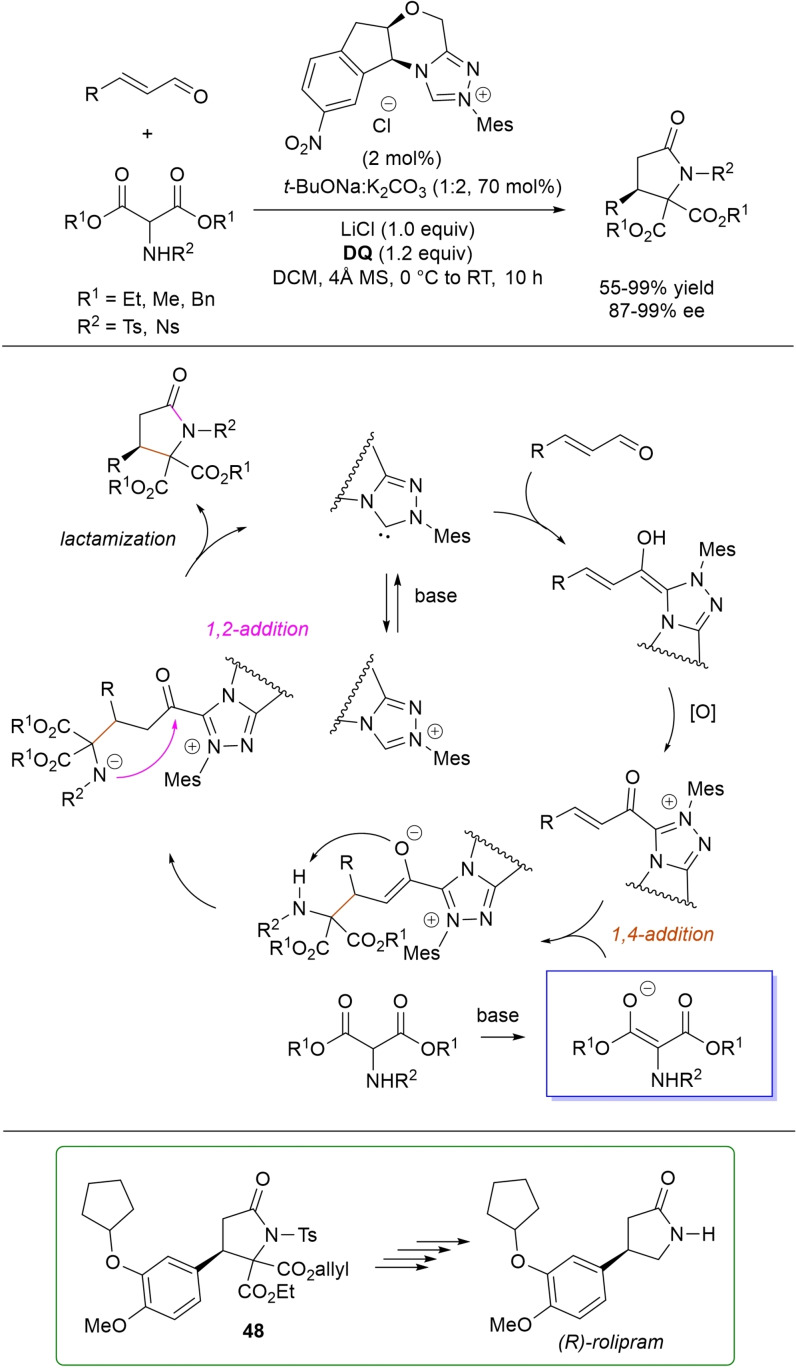
NHC‐catalyzed oxidative annulation of enals and *N*‐protected dialkyl aminomalonates.

By the same token, *N*‐tosyl (nosyl) dialkyl aminomalonates have been deployed in asymmetric NHC‐catalyzed oxidative annulation reactions with α,β‐unsaturated aldehydes bearing aryl, heteroaryl, naphthyl, fluorenyl, alkyl, and ester groups at β‐position, generating a huge library of (*R*)‐configured 4,5,5‐trisubstituted γ‐lactams.^[83]^ The very best results in terms of yields (55–99 %) and enantioselectivities (87–99 % ee) came from using only 2 mol% of a chiral aminoindanol‐derived triazolium pre‐catalyst (*N*‐mesityl substituent, nitro substituent on the indane moiety), in association with *t*‐BuONa/K_2_CO_3_ base mixture (1 : 2, 70 mol%), LiCl additive (1.0 equiv.) and **DQ** (1.2 equiv.) (Scheme 55).

Expectably, base‐promoted malonate C−H deprotonation creates the nucleophilic partner of α,β‐unsaturated acyl azolium for the C−C bond forming step (*1,4‐addition*). Then, intramolecular proton transfer/tautomerization and C−N bond assemblage (with regeneration of free NHC) point towards the lactam product.

The ability to scale‐up the reaction (1.0 g of *N*‐Ts diethyl aminomalonate, 81 % yield, 94 % ee) and turn γ‐lactam **48** into a medicinally sound molecule (i. e., the antidepressant, phosphodiesterase inhibitor (*R*)‐rolipram, Scheme 55, green box) were additional strengths of the methodology that deserve to be underlined.

**Scheme 56:**
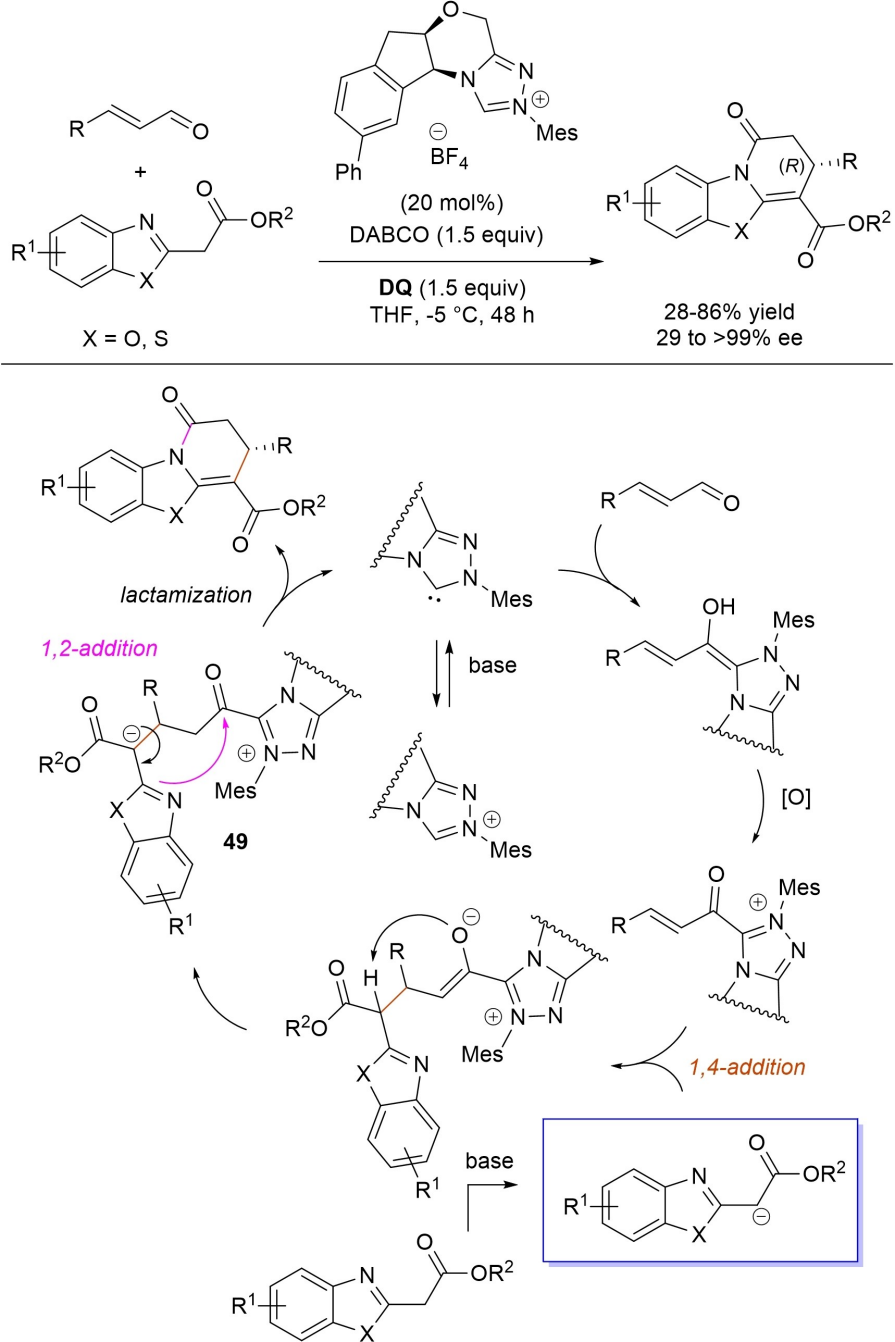
NHC‐catalyzed oxidative annulation of benzoxazole and benzothiazole esters with enals.

Very recently, oxidative NHC‐catalysis has found application for asymmetric dearomatizing annulation of benzoxazole‐ and benzothiazole‐derived esters with α,β‐unsaturated aldehydes fitted with (substituted) phenyl/naphthyl/furan‐2‐yl/alkenyl/ethyl formate groups.^[84]^ Dearomatized fused tricyclic heterocycles with (*R*)‐configuration have been produced in moderate to good yields (28–86 %) and moderate to excellent enantioselectivities (29 to >99 % ee) using the catalytic system composed of phenyl‐substituted chiral aminoindanol‐based triazolium salt (20 mol%), DABCO (1.5 equiv.), and **DQ** (1.5 equiv.) (Scheme 56).

Synthesis was tested in a large scale for the reaction between ethyl 2‐(benzo[*d*]oxazol‐2‐yl)acetate (1.0 mmol) and cinnamaldehyde (1.5 mmol), bringing the expected annulation adduct with 93 % ee (48 h: 60 % yield, 60 h: 66 % yield).

It is conceivable that the conjugate base of benzoxazole (benzothiazole) ester is captured by the α,β‐unsaturated acyl azolium acceptor, then proton transfer/tautomerization gives origin to the *C*,*N*‐bisnucleophile **49** that causes intramolecular lactamization (with regeneration of NHC catalyst).

**Scheme 57:**
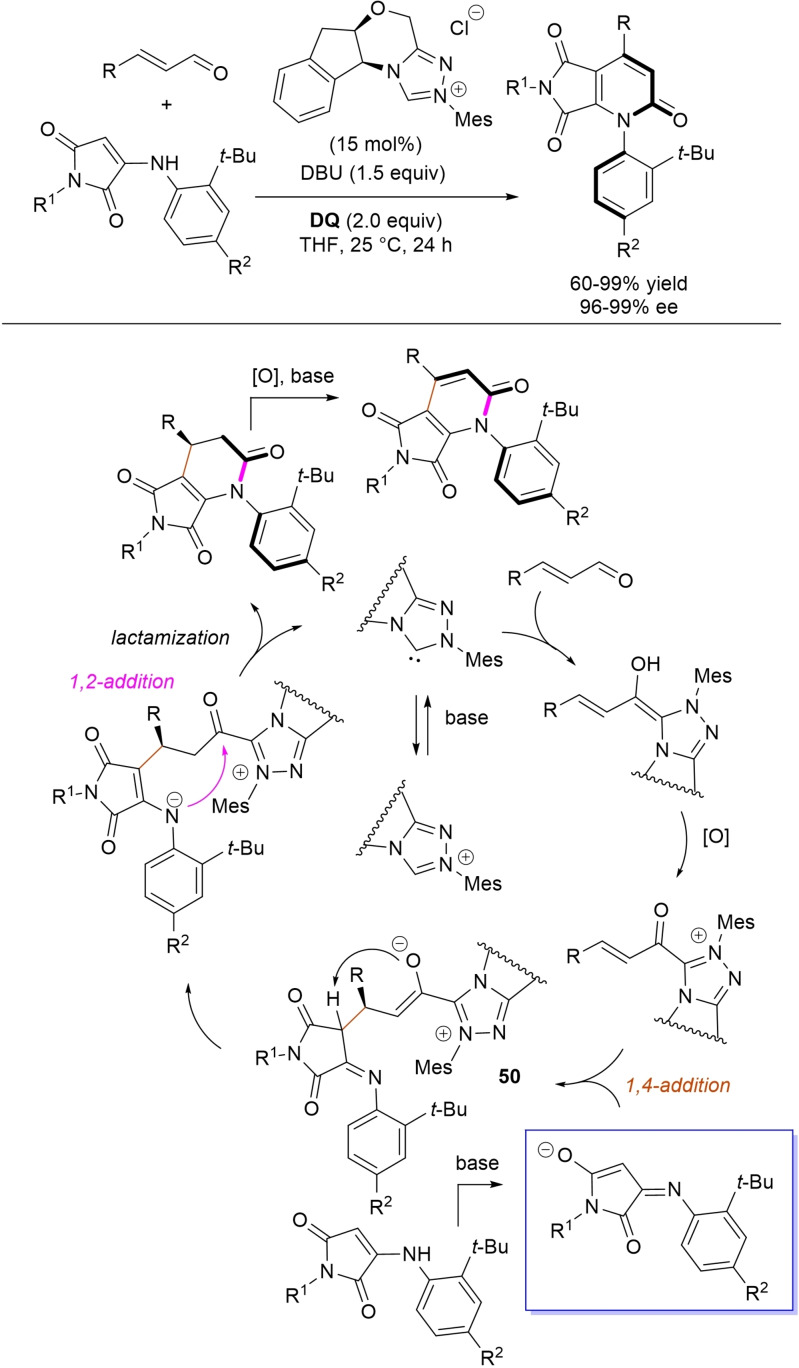
NHC‐catalyzed oxidative annulation of maleimides with enals.

Oxidative NHC‐catalysis proved to be a winning ticket for atroposelective synthesis of pyrrolo[3,4‐*b*]pyridines by Michael/lactamization reaction of enals with 3‐arylamino‐substituted maleimides.^[85]^ When these two components were placed in presence of chiral aminoindanol‐derived triazolium pre‐catalyst (15 mol%), DBU (1.5 equiv.), and **DQ** (2.0 equiv.), axially chiral adducts (C−N axis, *R*‐configuration) were formed in good to high yields (60–99 %) (Scheme 57). These results were irrespective of the type of substituents on both the α,β‐unsaturated aldehyde (aryl, heteroaryl, naphthyl) and the endocyclic nitrogen atom of maleimide (e. g., benzyl, cyclopropyl, phenylethyl, heteroarylmethyl). By contrast, enantioselectivities were very dependent on the presence of an *ortho* bulky (*t*‐Bu) group in the 3‐arylamino substituent of maleimide. In such cases, outstanding 96–99 % ee values have been reached, with a severe drop in enantioselectivities (9‐34 % ee) caused by smaller groups (i. e., OMe, *i*‐Pr, Br, I).

It is believed that the α,β‐unsaturated acyl azolium which is formed from enal, NHC and **DQ** interacts with maleimide‐derived enolate in a 1,4‐fashion to yield adduct **50** from attack on the *Re*‐face. Subsequently, proton transfer and tautomerization give the requisite *N*‐nucleophile for lactam formation, and lastly oxidation of this one leads to the desired product.

It is considered important to highlight that an alternative was reported for the usual mechanistic scheme describing dual C−C/C−N bond formation, specifically for reactions of alkenyl and alkynyl acyl azolium intermediates with nucleophilic 2‐aminoacrylates towards unsaturated lactam derivatives.

**Scheme 58:**
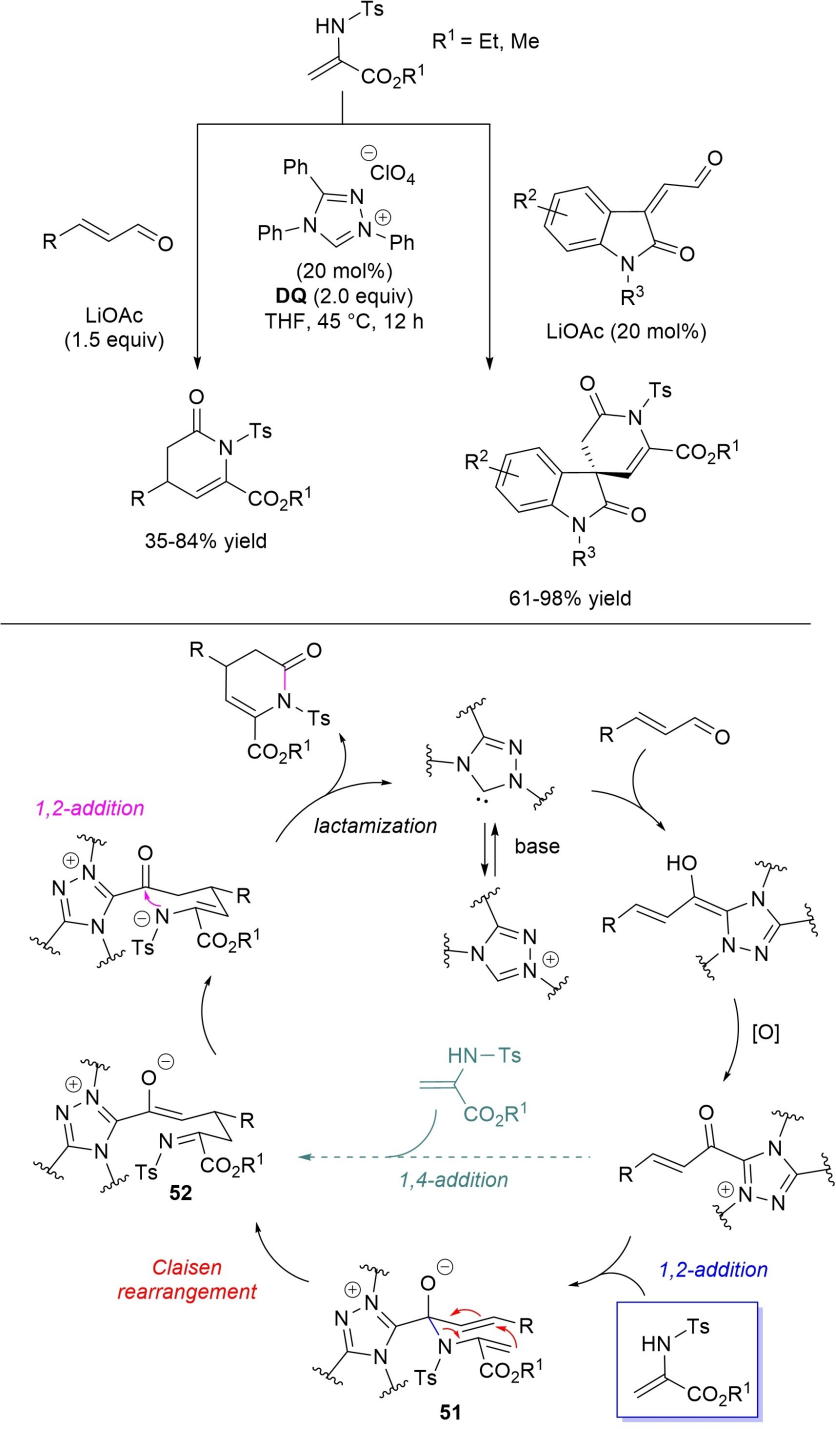
NHC‐catalyzed oxidative annulation of *N*‐tosyl 2‐aminoacrylates with enals.

In this matter, Qi and co‐workers illustrated the oxidative NHC‐catalyzed annulation of *N*‐tosyl 2‐aminoacrylates with enals having (hetero)aryl/naphthyl/styryl groups at β‐position, using triphenyl‐substituted triazolium pre‐catalyst (20 mol%), LiOAc as the base (1.5 equiv.) and **DQ** (2.0 equiv.).^[86]^ Accordingly, 5,6‐dihydropyridinones could be obtained (35–84 % yields), and slightly adjusted conditions were applied to isatin‐derived enals (20 mol% of base) to produce spirooxindole derivatives in good to excellent yield (61–98 %) (Scheme 58). These results have been explained by assuming that the 2‐aminoacrylate component attacks the carbonyl group of α,β‐unsaturated acyl azolium (*1,2‐addition*) forming intermediate **51**, that experiences Claisen rearrangement to give **52**.^[87]^ Thereafter, proton transfer, tautomerization and lactamization yield the final pyridinone compound. However, the path occurring via the usual Michael/lactamization steps cannot be totally excluded (Scheme 58, green route).

**Scheme 59:**
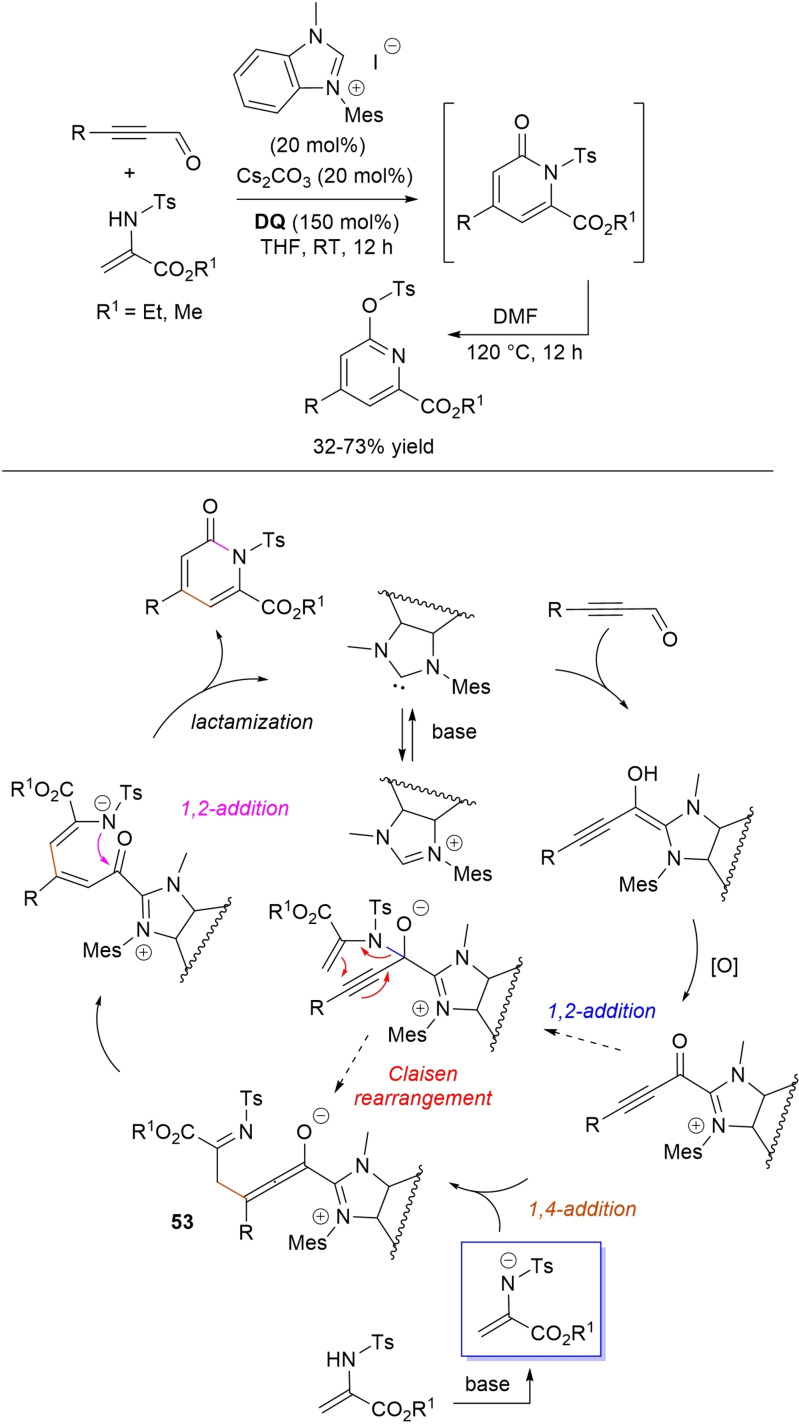
NHC‐catalyzed oxidative annulation of *N*‐tosyl 2‐aminoacrylates with ynals.

Later, similar mechanistic explanations were raised for the reactions between *N*‐tosyl 2‐aminoacrylates and ynals, bearing (hetero)aromatic/naphthyl/vinyl/aliphatic/indole groups, with *N*‐mesityl‐*N*‐methyl‐substituted benzimidazolium pre‐catalyst/Cs_2_CO_3_ (20 mol% each) and **DQ** (150 mol%) (Scheme 59).^[88]^ The deprotonated aminoacrylate component may promote either a 1,2‐addition/Claisen rearrangement or conjugate addition, with allenolate species **53** created in both cases. Following, proton transfer/lactamization supplies *N*‐protected 4,6‐disubstituted pyridin‐2(1*H*)‐ones, prone to be converted into the corresponding pyridines by heating (120 °C, DMF).

**Scheme 60:**
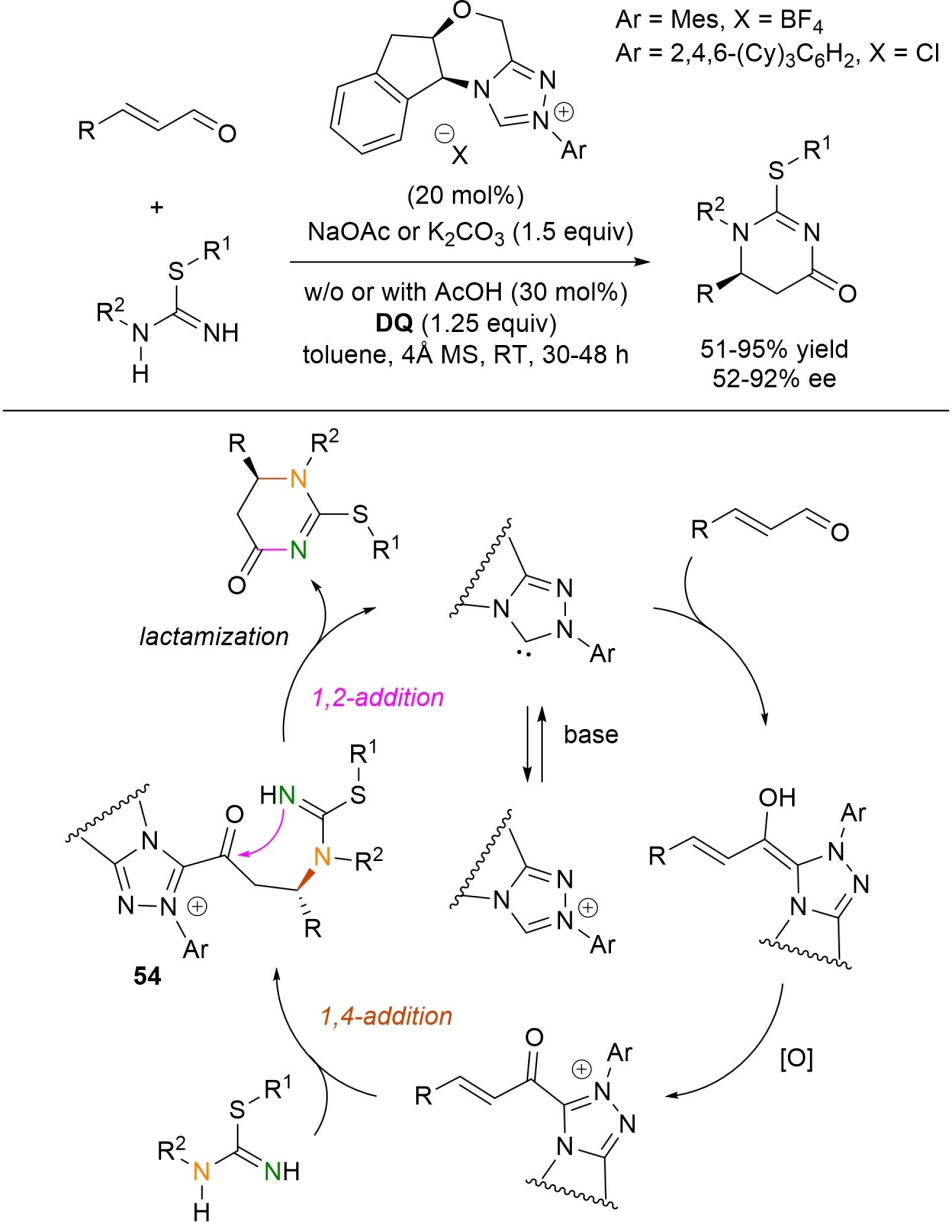
NHC‐catalyzed oxidative annulation of isothioureas with enals.

Concomitant C−N/C−N bond formation was the mark of the NHC‐catalyzed oxidative addition of *S*‐alkylated isothioureas to (hetero)aryl/naphthyl/alkyl/aliphatic‐substituted enals.^[89]^ Under the presence of chiral aminoindanol‐fused triazolium pre‐catalyst (20 mol%), NaOAc (1.5 equiv.), AcOH additive (30 mol%) and **DQ** (1.25 equiv.), sulphured 5,6‐dihydropyrimidin‐4‐one frameworks were formed as single regioisomers (Scheme 60). The observed outcomes much depended on the *N*‐protecting group of isothiourea: an acceptable 74 % ee value (66 % yield) was secured for the heterocyclic product derived from unprotected *S*‐methylated isothiourea as long as sterically hindered NHC precursor (*N*‐2,4,6‐tricyclohexylphenyl substituent, 20 mol%) was used, along with K_2_CO_3_ as base and no additive.

Successful wide‐scale experiments were run using the reaction between cinnamaldehyde and *S*‐methyl phenylisothiourea (1.66 g) as the model, the due annulated product being obtained with preserved efficiency (72 % yield) and enantioselectivity (86 % ee) compared to the typical reaction conditions (78 % yield, 90 % ee).

The process is proposed to start with the *aza*‐Michael addition of the protected nitrogen atom of the isothiourea to the catalytically generated α,β‐unsaturated acyl azolium to provide acyl azolium **54**. Then, lactamization via acylation of the unprotected nitrogen atom of isothiourea completes the organocatalytic cycle.

Cascade transformations leading to heterocyclic structures via sequential C−N/C−N bond formation have involved ynal‐derived acyl azolium intermediates, too.

**Scheme 61:**
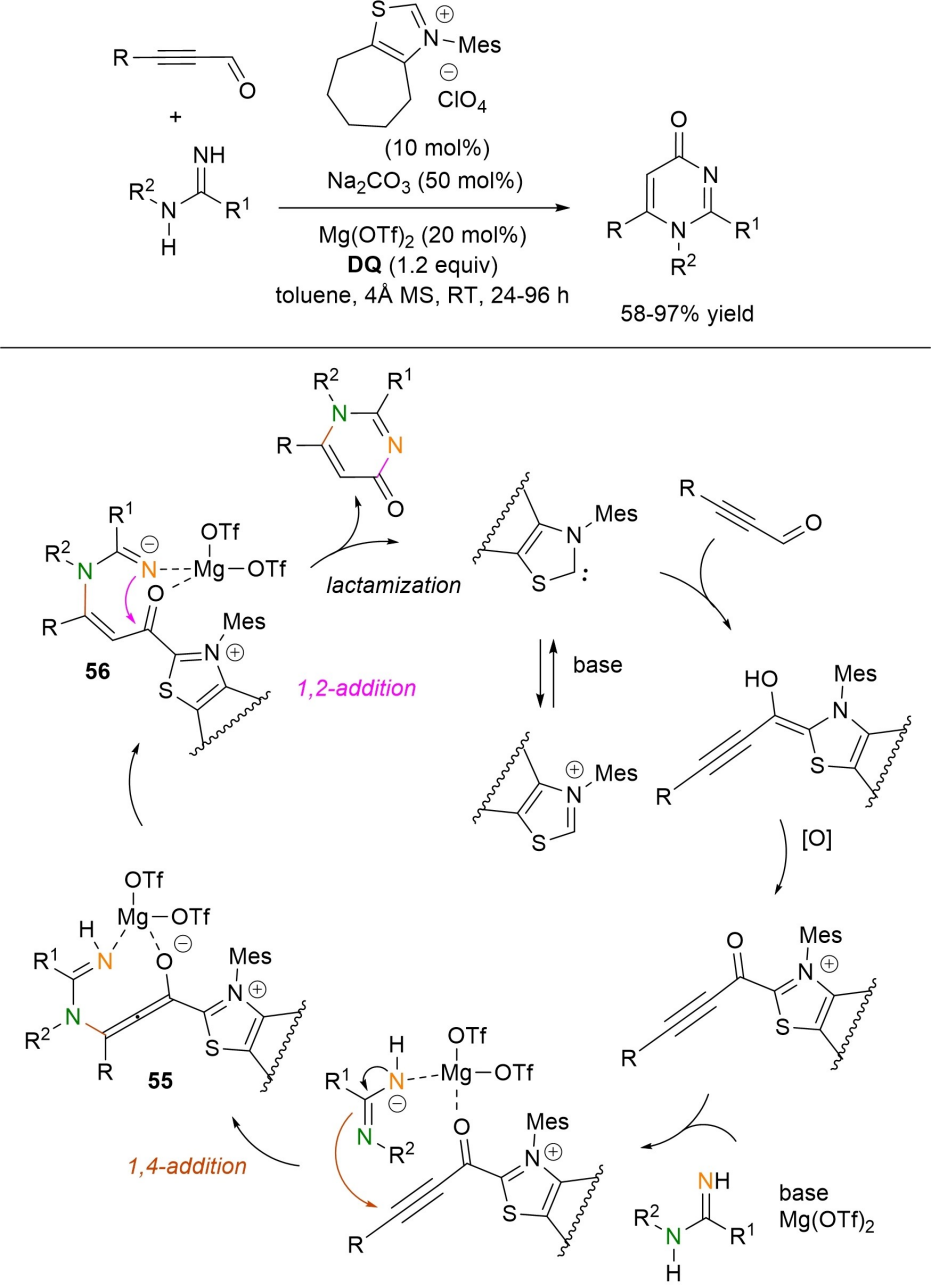
NHC‐catalyzed oxidative annulation of *N*‐protected amidines with ynals.

As a case in point, NHC‐catalyzed condensation of ynals and *N*‐substituted amidines under oxidative conditions opened the avenue to the assembly of 1,2,6‐trisubstituted pyrimidin‐4‐ones.^[90]^ The optimal protocol was based on the use of cycloheptane‐fused thiazolium pre‐catalyst (10 mol%), Na_2_CO_3_ (50 mol%), Mg(OTf)_2_ (20 mol%) and **DQ** (1.2 equiv.) (Scheme 61), good to high yields (58–97 %) and excellent regioselectivity being distinctive elements to mention. And apart from this, broad substrate scope and tolerance of functional groups was exhibited, aryl, heteroaryl, naphthyl, and alkyl groups being well tolerated on both ynal and amidine structures.

It is presumed that simultaneous activation of the amidine and the ynal‐derived alkynyl acyl azolium by the Lewis acid sets the stage for *aza*‐Michael addition, which results in the *N*‐nucleophile **56** via allenolate **55**. Then, intramolecular *N*‐acylation and NHC detachment liberate the pyrimidin‐4‐one product.

**Scheme 62:**
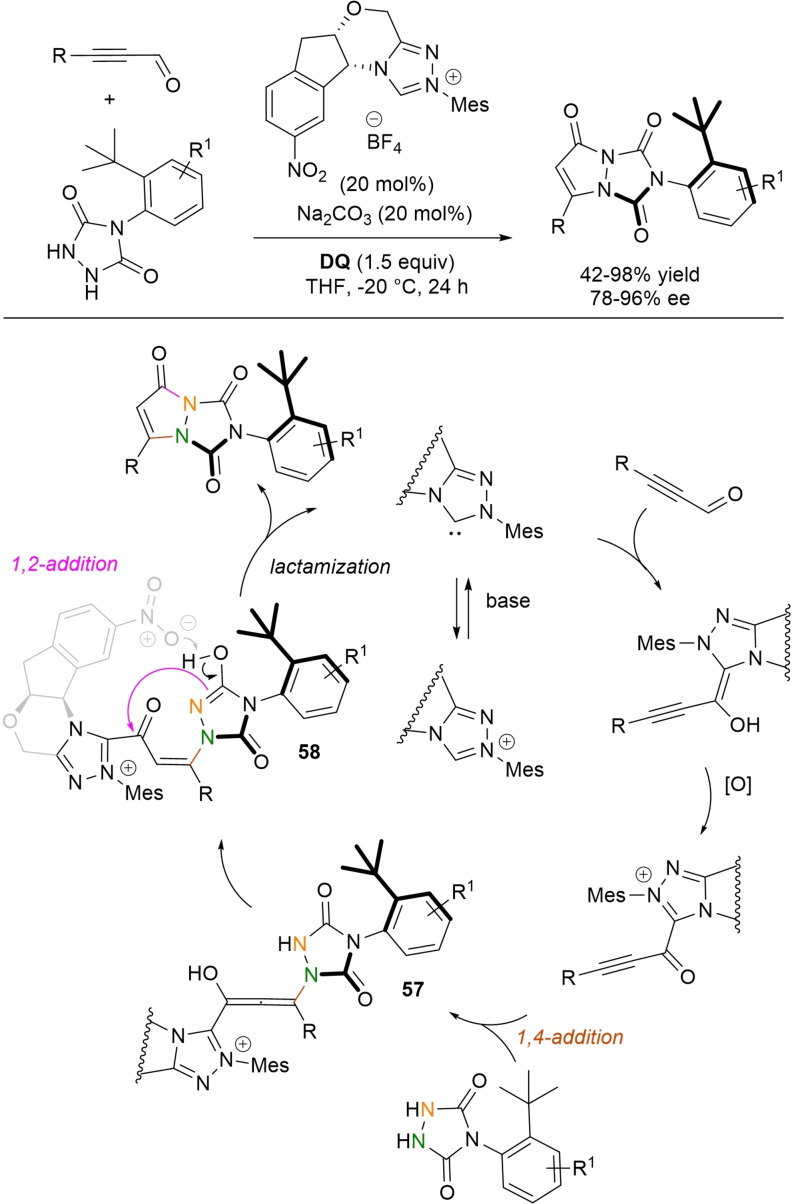
NHC‐catalyzed oxidative annulation of *N*‐aryl urazoles with ynals.

Instead of that, partnering ynals with 4‐aryl urazoles under oxidative NHC‐catalysis gave access to axially chiral (C−N axis) pyrazolo[1,2‐*a*]triazole derivatives with good to excellent yields (42–98 %) and enantioselectivities (78–96 % ee), best suited conditions being established by combination of **DQ** (1.5 equiv.) with nitro‐substituted chiral aminoindanol‐derived triazolium pre‐catalyst (20 mol%) and Na_2_CO_3_ (20 mol%) (Scheme 62).^[91]^ Various substitution patterns on both ynals and urazoles were compatible with this reaction, an *ortho t*‐Bu group on the urazole *N*‐aryl ring proving essential to maintain the stereochemical stability of the products.

A simplified mechanistic path can be given that brings into play an atroposelective *aza*‐Michael addition of the urazole substrate with the alkynyl acyl azolium ion to afford **57** as the major adduct. Next, isomerization to α,β‐unsaturated acyl azolium **58** and lactam formation yield the desired axially chiral bicyclic product recovering the free NHC catalyst. It cannot be hidden that the process goes together with desymmetrization of the urazole starting material through dynamic KR between **57** and a diastereomeric 1,4‐addition adduct which reverts to the alkynyl acyl azolium and urazole for steric reasons (*not shown*).

**Scheme 63:**
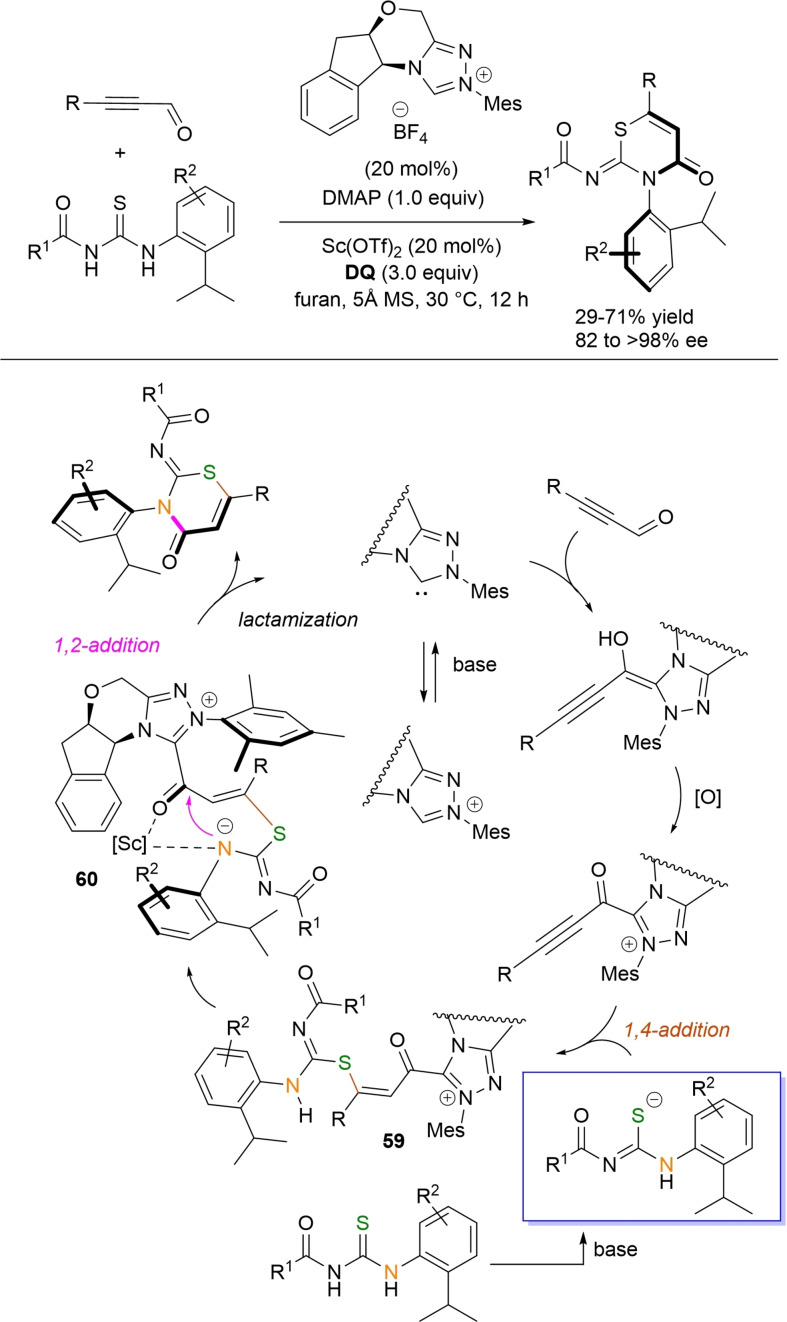
NHC‐catalyzed oxidative annulation of *N*‐acyl‐*N*‐aryl thioureas with ynals.

A similar logic applied for the NHC‐catalyzed annulation between *N*‐acyl‐*N*‐aryl thioureas and ynals to access thiazine compounds bearing chiral C−N axes, through the medium of aminoindanol‐based triazolium pre‐catalyst (20 mol%), DMAP (1.0 equiv.), Sc(OTf)_3_ additive (20 mol%) and **DQ** (3.0 equiv.) (Scheme 63).^[92]^ This method was compatible with aryl (naphthyl) groups on both ynal and the thiourea acyl moiety, and also with heteroaryl/alkyl units on the thiourea acyl group. Last but not least, the presence of 2‐isopropyl group on the *N*‐aryl substituent of thiourea was fundamental to both chirality induction and stereochemical stability of the final products.

In terms of reaction mechanism, NHC‐promoted ynal‐to‐alkynyl acyl azolium conversion brings forward the installation of C−S bond through *thio*‐Michael addition to create the acyl azolium **59**. At this stage, Lewis acid‐assisted face‐selective C−N bond formation takes place for means of the preferred transition state **60**, which relieves steric interactions between the congested *N*‐(2‐isopropylphenyl) group and the NHC skeleton.

### 3.3 C−C/C−C/C−O and C−N/C−C/C−O bond formation (Michael/aldol/lactonization and Michael/Michael/lactonization sequences)

NHC‐catalyzed strategies triggered by reaction of α,β‐unsaturated acyl azolium species with threefold reactive reagents have been uncovered by several researchers. This group includes all annulation processes that proceed through domino Michael/aldol/lactonization sequences, optionally accompanied by further synthetic elaborations, typically CO_2_‐fragmentation, CO_2_‐fragmentation/oxidation, and dehydration.

**Scheme 64:**
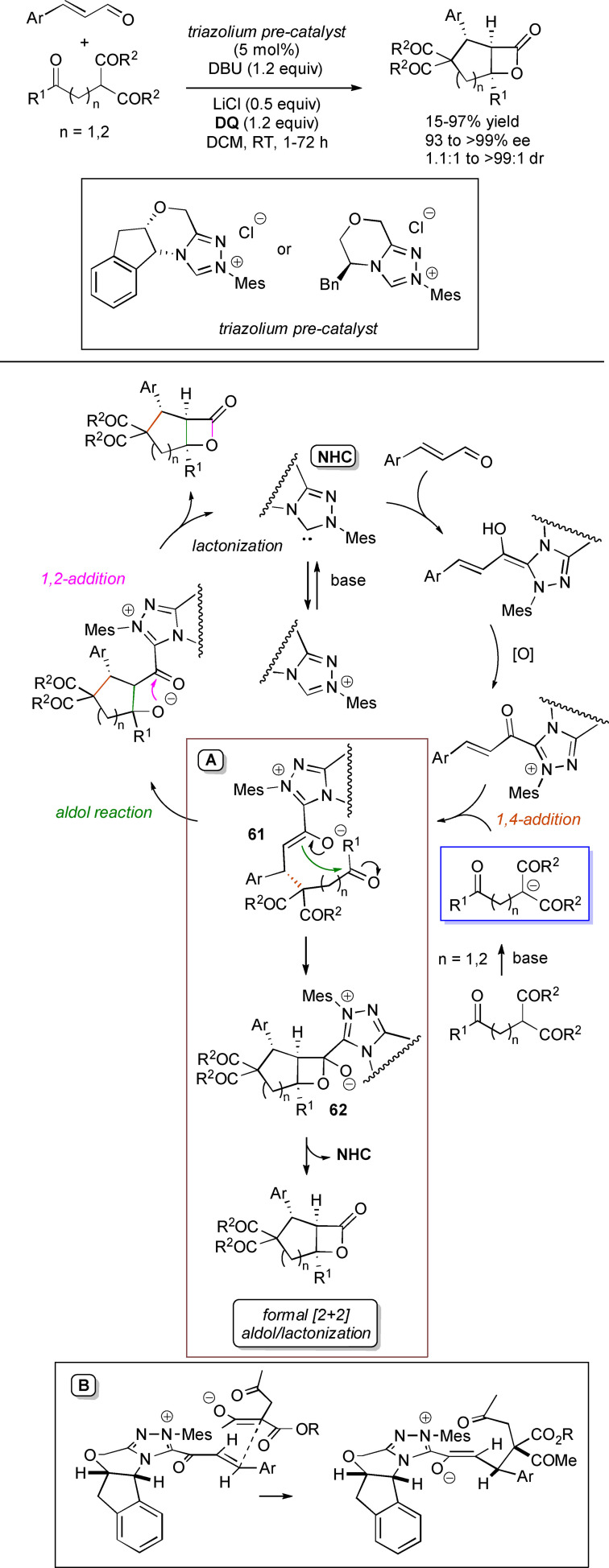
NHC‐catalyzed oxidative annulation of β‐diketones, β‐ketoesters, and malonates with enals.

In this respect, Studer and co‐workers reported cascade reactions of α,β‐unsaturated aldehydes (i. e., cinnamaldehyde, 4‐methoxycinnamaldehyde, 4‐nitrocinnamaldehyde) with β‐diketones, β‐ketoesters, and malonates featuring a β‐oxyalkyl moiety at α‐position,^[93]^ with highly substituted carbocycle‐fused β‐lactones bearing up to four contiguous stereogenic centers (two quaternary ones) obtained (Scheme 64). It was found that cyclopentane‐annulated β‐lactones formed with excellent stereoselectivities (93 to >99 % ee, 1.1 : 1 to >99 : 1 dr) with aminoindanol‐derived chiral triazolium salt (5 mol%), DBU (1.2 equiv.), LiCl (0.5 equiv.), and **DQ** (1.2 equiv.). Switch to morpholine‐based chiral NHC demonstrated the feasibility of assembling congeneric cyclohexane derivatives, as proved by the model reaction between dimethyl 2‐(3‐oxobutyl)malonate and cinnamaldehyde (54 % yield, 80 % ee, >99 : 1 dr).

It has been argued that Michael addition of deprotonated dicarbonyl compound to α,β‐unsaturated acyl azolium gives enolate **61**, which can follow either one of two routes. The first option is a concerted, asynchronous formal [2+2] aldol lactonization via intermediate **62**,^[94]^ later turned into β‐lactone product by releasing active NHC (Scheme 64A). Otherwise, **61** may take part in sequential intramolecular aldol reaction and lactonization. In all cases, the Lewis acid seems to favour complexation of the *O*‐atom of the α,β‐unsaturated acyl azolium ion helping to lower the LUMO and, as a result, activate the Michael acceptor.

With special attention to the annulation reactions of β‐ketoesters and in line with the calculated transition state for the addition of acetylacetone anion to an acyl azolium ion,^[95]^ it is likely that conjugate addition of the tertiary *C*‐nucleophile occurs on the C=C face opposite to the NHC catalyst core, so as to bring the bulky ester closest to the β‐H atom of the Michael acceptor (Scheme 64B). On the one hand such an organization of the transition state accounts for the greater enantioselectivity observed with the bulkier *t*‐butyl‐ and mesityl‐esters, on the other it can explain the diastereoselectivity in the addition stage.

**Scheme 65:**
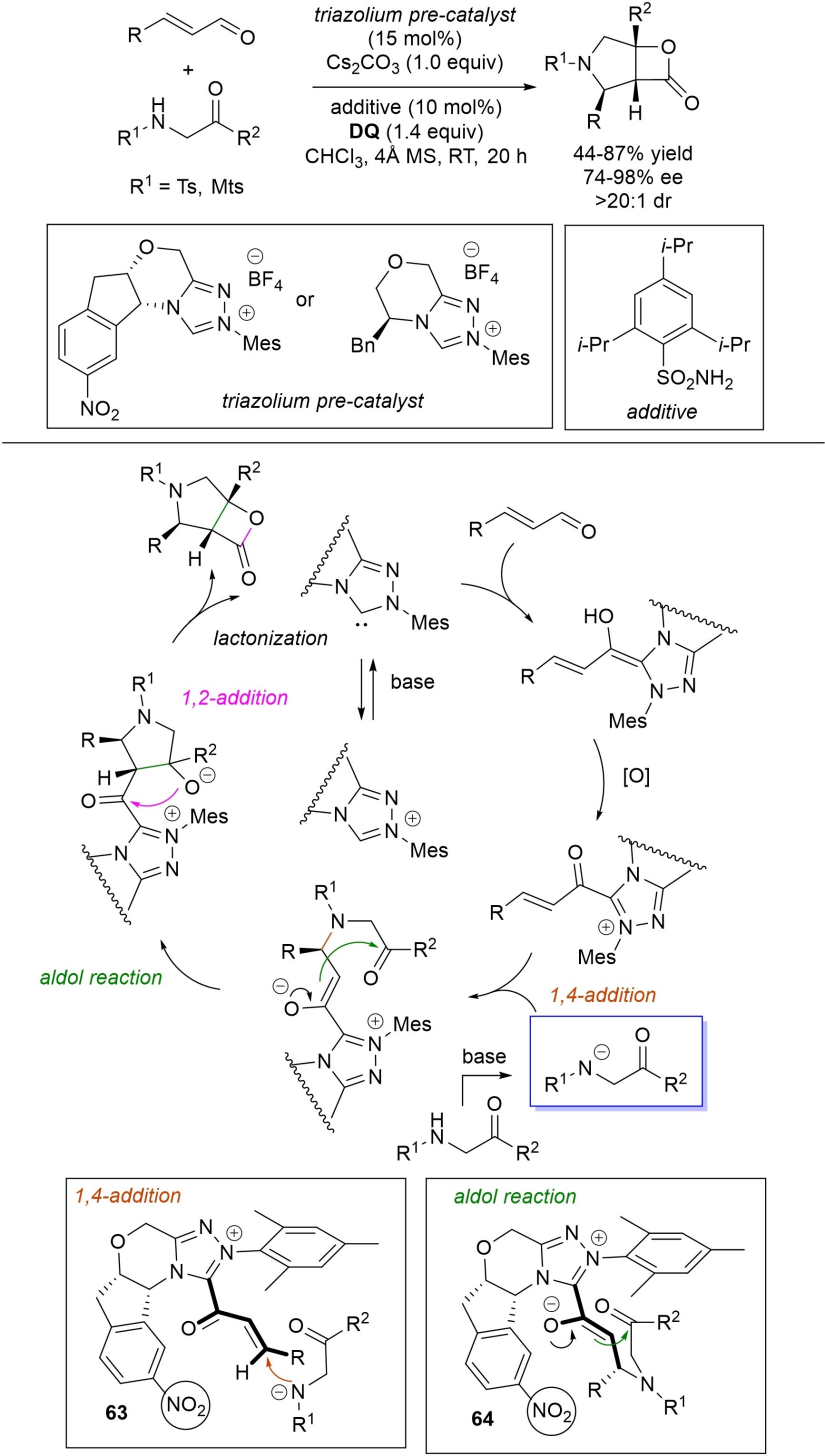
NHC‐catalyzed oxidative annulation of *N*‐protected α‐aminoketones with enals.

A simple variation of this approach involves utilization of *N*‐protected α‐aminoketones in the NHC‐catalyzed oxidative reaction with enals, thereby enabling a domino C−N/C−C/C−O bond‐forming sequence for the synthesis of pyrrolidine‐annulated β‐lactones (Scheme 65).^[96]^

Fair to good yields (44–87 %) and good to excellent stereoselectivities (74–98 % ee, >20 : 1 dr) of pyrrolidine‐fused β‐lactones were brought about through the use of the chiral NHC derived from nitro‐substituted aminoindanol‐based triazolium pre‐catalyst (15 mol%) and Cs_2_CO_3_ (1.0 equiv.), together to 2,4,6‐triisopropylbenzenesulfonamide additive (10 mol%) and **DQ** (1.4 equiv.) (Scheme 65).

The speculated mechanism requires an *aza*‐Michael addition of the amide nucleophile to the α,β‐unsaturated acyl azolium, with the adduct obtained involved in a successive intramolecular aldol reaction. The following lactonization (with release of the NHC catalyst) furnishes the bicyclic β‐lactone. One can predict that shielding of the back face of the unsaturated acyl azolium by the indanol backbone directs addition of the nucleophile from the *Si*‐face (transition state **63**), explaining perhaps the enantioselection of the reaction. On the other hand, high diastereoselectivity may arise from the intramolecular aldol reaction step, possibly via the envelope‐type conformation of transition state **64**.

A broad scope of β‐aryl/heteroaryl/alkyl enals and alkyl α‐aminoketones was screened, *N*‐tosyl protection being apt except for β‐alkyl enals, for which the 2,4,6‐trimethylbenzenesulfonyl (Mts) group at *N*‐atom proved to be more suited. It is also the case to underline that benzyl‐substituted morpholine‐derived chiral triazolium salt needed to be used with a bulky‐ketone, while reactions of aryl α‐aminoketones occurred with formation of 3‐pyrroline products (73–82 % yield, 90 to >98 % ee) as a result of spontaneous decarboxylation of the initially formed β‐lactone.

Pairing of a decarboxylation step with the oxidative NHC‐catalyzed Michael/aldol/lactonization sequence resulted in the building of both 1,2‐dihydronaphthalenes and fused heterocyclic systems, such as 5,6‐dihydroindolizines.

**Scheme 66:**
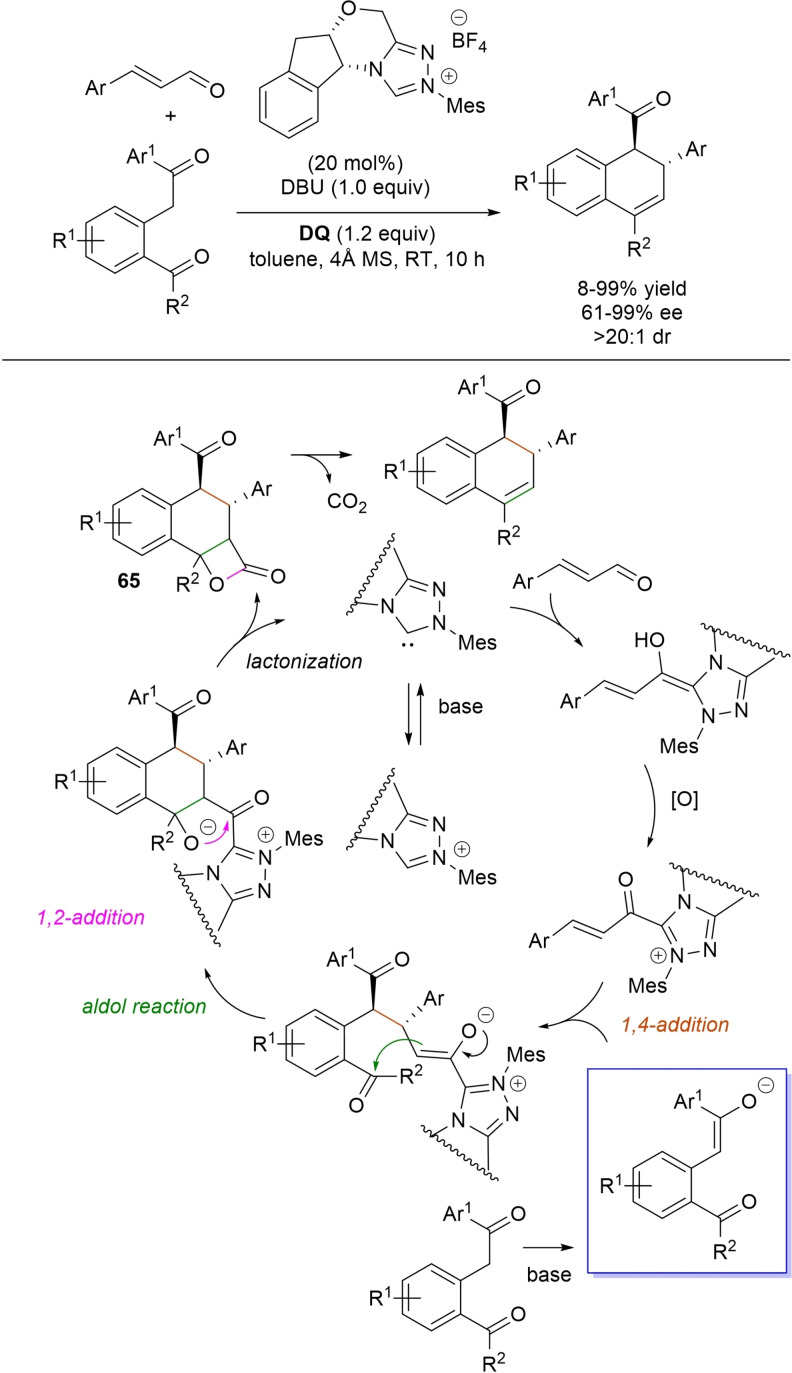
NHC‐catalyzed oxidative annulation of benzodiketones with enals.

Namely, differently substituted benzodiketones and β‐aryl/naphthyl enals were reacted with chiral aminoindanol‐derived triazolium pre‐catalyst (20 mol%), DBU (1.0 equiv.) and **DQ** (1.2 equiv.) to afford a series of diastereomerically/enantiomerically enriched *trans*‐disubstituted‐1,2‐dihydronaphthalenes (8‐99 % yield, 61–99 % ee, >20 : 1 dr) (Scheme 66), with a 0.5 g scale reaction of 2‐(2‐benzoylphenyl)‐1‐phenylethan‐1‐one with cinnamaldehyde successfully run (91 % yield, >20 : 1 dr, 94 % ee).^[97]^

The enal‐derived α,β‐unsaturated acyl azolium undergoes Michael reaction with the ketone enolate to install the first C−C bond, then aldol reaction and intramolecular *O*‐acylation (with NHC regeneration) assemble the remaining C−C and C−O bonds. This gives birth to the anticipated β‐lactone intermediate **65** that spontaneously decarboxylates to the final dihydronaphthalene scaffold.

**Scheme 67:**
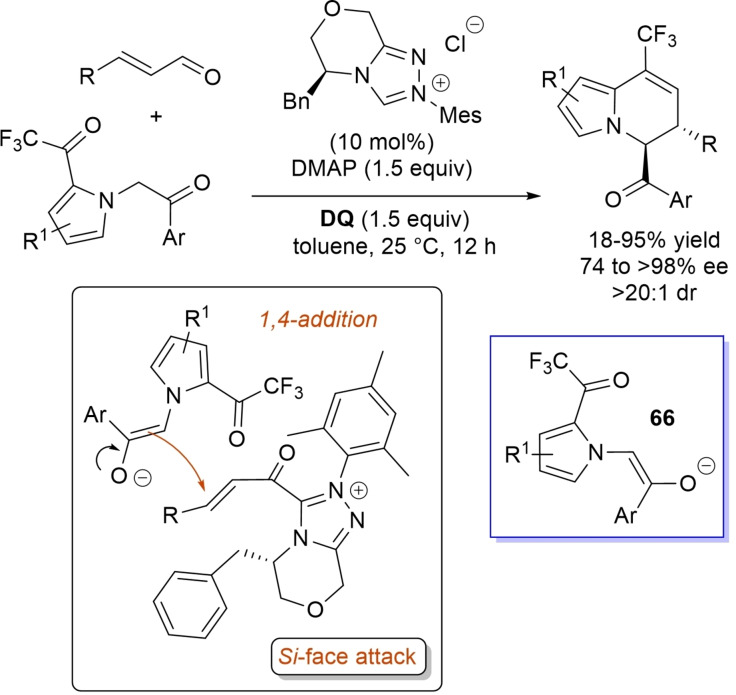
NHC‐catalyzed oxidative annulation of *N*‐substituted 2‐(trifluoroacetyl)pyrroles with enals.

In a similar mechanistic fashion, the entrapment of enal‐derived α,β‐unsaturated acyl azoliums with enolate donors **66**, having *N*‐substituted 2‐(trifluoroacetyl)pyrroles as precursors, was the initiation of a domino Michael/aldol/lactonization process for tricyclic β‐lactones, which afforded trifluoromethylated 5,6‐dihydroindolizines after CO_2_‐fragmentation.^[98]^ The best catalytic system proved to be the one formed by benzyl‐substituted morpholine‐derived chiral triazolium salt (10 mol%), DMAP (1.5 equiv.), and **DQ** (1.5 equiv.) (Scheme 67), with wide scope of enals and pyrroles as well as scalability (1.0 mmol scale) shown.

It must be pointed out that the asymmetric induction reasonably takes place in the conjugate addition step, favoured approach of the enolate from the *Si*‐face of the Michael acceptor adjusting the aryl (alkyl) and aroyl residues in *anti* positions relative to the new formed C−C bond.

Under certain circumstances, the β‐lactone decarboxylation step is accompanied by further oxidation to construct an aromatic ring. This is the case of NHC‐catalyzed formal [4+2]‐benzannulation of enals with cyano‐bearing enones, 2‐methyl‐3‐oxoacetate indoles, and pyrimidine‐2,4‐diones for access to benzonitriles, carbazoles and quinazoline‐2,4‐diones, respectively.

**Scheme 68:**
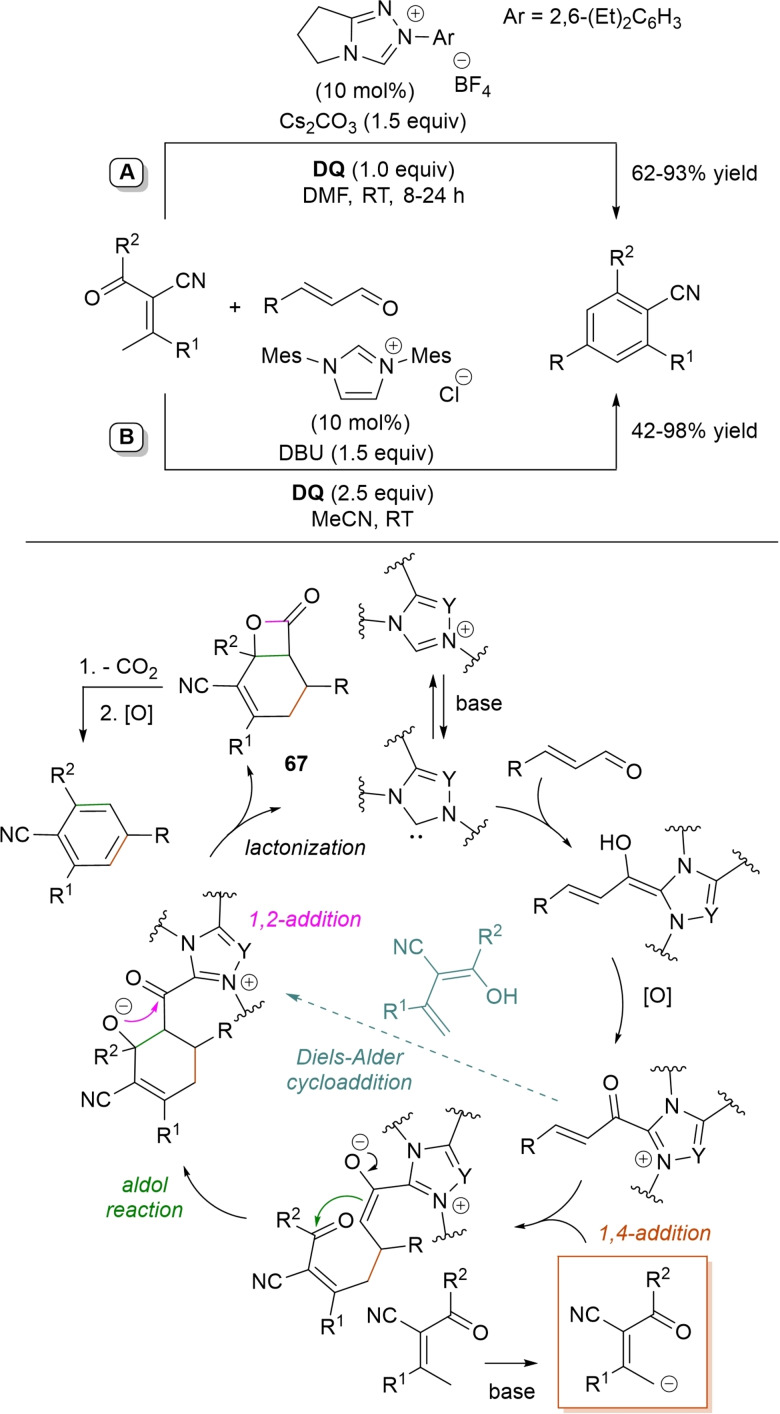
NHC‐catalyzed oxidative annulation of α‐cyano‐β‐methylenones with enals.

In 2016, Wang and Ye independently capitalised on oxidative NHC‐catalysis for the synthesis of polysubstituted benzonitriles from α‐cyano‐β‐methylenones and enals.[^99^, ^100^] In Wang's work, these substrates gave efficacious reactions (62–93 % yield) with enals containing phenyl, anthracenyl, heteroaryl, and alkyl units, as also alkenyl, alkynyl, ether, and ester groups at β‐position, using *N*‐2,6‐diethylphenyl‐substituted pyrrolidine‐based triazolium pre‐catalyst (10 mol%), Cs_2_CO_3_ (1.5 equiv.) and stoichiometric **DQ** (Scheme 68A).^[99]^

On the other hand, Ye's method was based on the use of 1,3‐dimesityl imidazolium chloride (10 mol%) and DBU (1.5 equiv.), with best results observed when 2.5 equiv. of **DQ** were used (Scheme 68B).^[100]^ Alkyl or (hetero)aryl enones appended with β‐(hetero)aryl or naphthyl groups reacted smoothly with β‐(hetero)aryl‐α,β‐unsaturated aldehydes to afford benzonitrile products in moderate to good yields (42–98 %) and full regioselectivity.

The deprotonated α‐cyano enone enters the usual organocatalytic cycle initiated by Michael addition to the α,β‐unsaturated acyl azolium, leading to the creation of bicyclic β‐lactone adduct **67**, then consecutive decarboxylation and oxidation complete the synthesis of the benzonitrile framework.^[99]^ However, an alternative path entailing a Diels‐Alder cycloaddition between enone‐derived dienolate and acyl azolium (dienophile) was also put forward.^[100]^

**Scheme 69:**
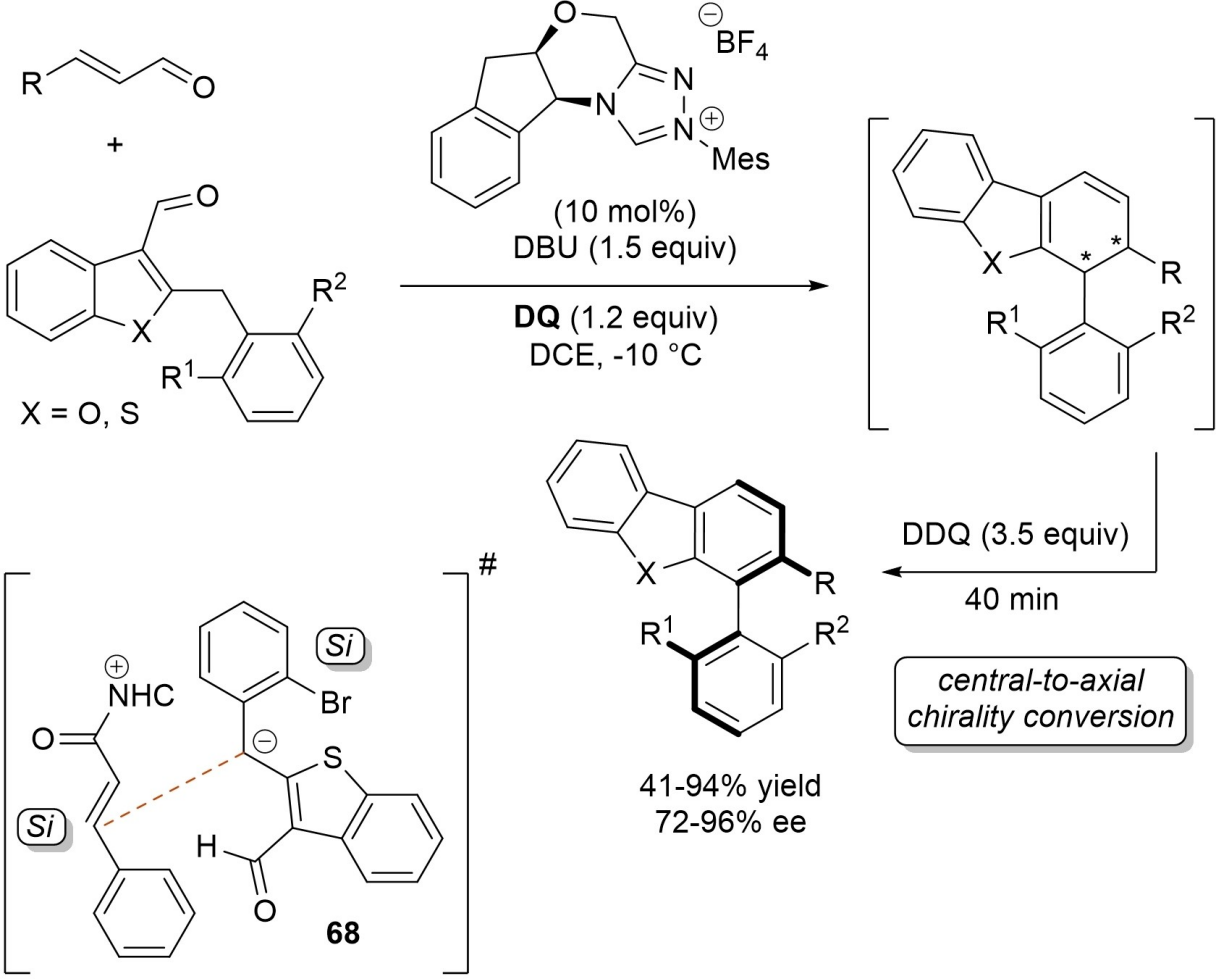
NHC‐catalyzed synthesis of axially chiral biaryls under oxidative conditions.

Recently, still Ye and co‐workers enacted the asymmetric variant of the NHC‐promoted arene formation reaction for the enantioselective synthesis of axially chiral benzothiophene/benzofuran‐fused biaryls from 2‐benzyl‐benzothiophene/benzofuran‐3‐carboxaldehydes and enals.^[101]^ The catalytic system formed by chiral aminoindanol‐derived triazolium salt (10 mol%) and DBU (1.5 equiv.), in conjunction with **DQ** (1.2 equiv.) and 2,3‐dichloro‐5,6‐dicyano‐1,4‐benzoquinone (DDQ) as a co‐oxidant (for oxidative aromatization) was ideal for reacting a great variety of enals and 2‐benzyl‐benzothiophene‐3‐carboxaldehydes (Scheme 69). This gave a wide library of enantioenriched tri‐ and tetra‐*ortho*‐substituted benzothiophene‐fused biaryls (a*R*‐configuration) with complete chemoselectivity, moderate to good yields (44‐94 %), and good to high enantioselectivities (72‐96 % ee).

Similarly, reaction of β‐aryl enals with 2‐(2‐bromobenzyl)benzofuran‐3‐carboxaldehyde and its 5‐ or 6‐substituted analogs gave the expected axially chiral benzofuran‐fused biaryl adducts with good enantioselectivities (78–91 % ee), but with moderate yields (41–55 %).

It's notable that switching from lab‐scale to gram‐scale synthesis (2.8–3.5 mmol of benzothiophene aldehyde) has demonstrated the high practicability of the developed protocol, with good to high yields (75–92 %) and high enantioselectivities (92–98 % ee) observed. Meanwhile, the synthetic utility of the products obtained has been proved by a number of chemical transformations (e. g., metal‐catalyzed cross coupling, reduction, processing to potential organocatalysts).

Very recent DFT studies on the model reaction between cinnamaldehyde and 2‐(2‐bromobenzyl)benzothiophene‐3‐carboxaldehyde have made clear the mechanism and the source of both chemo‐ and stereoselectivities.^[102]^ It could be established that the overall organocatalytic path involves the compulsory steps already described, that is, generation of the Breslow intermediate, subsequent oxidation by **DQ**, C−C bond formation (*1,4‐addition*), dual C−C/C−O bond assemblage (*intramolecular [2+2] cyclization*), NHC dissociation, decarboxylation, oxidative aromatization (with conclusive central‐to‐axial chirality conversion). What is important is that the route leading to the *R*‐configured axially chiral product is energetically favourable, as a consequence of non‐covalent interactions (C−H‐ ‐ ‐O, C−H‐ ‐ ‐S, C−H‐ ‐ ‐Br, C−H‐ ‐ ‐π) in the key transition state **68** related to the first C−C bond forming step (the *Si* face of the enolate attacks the *Si* face of the electrophilic alkene).

**Scheme 70:**
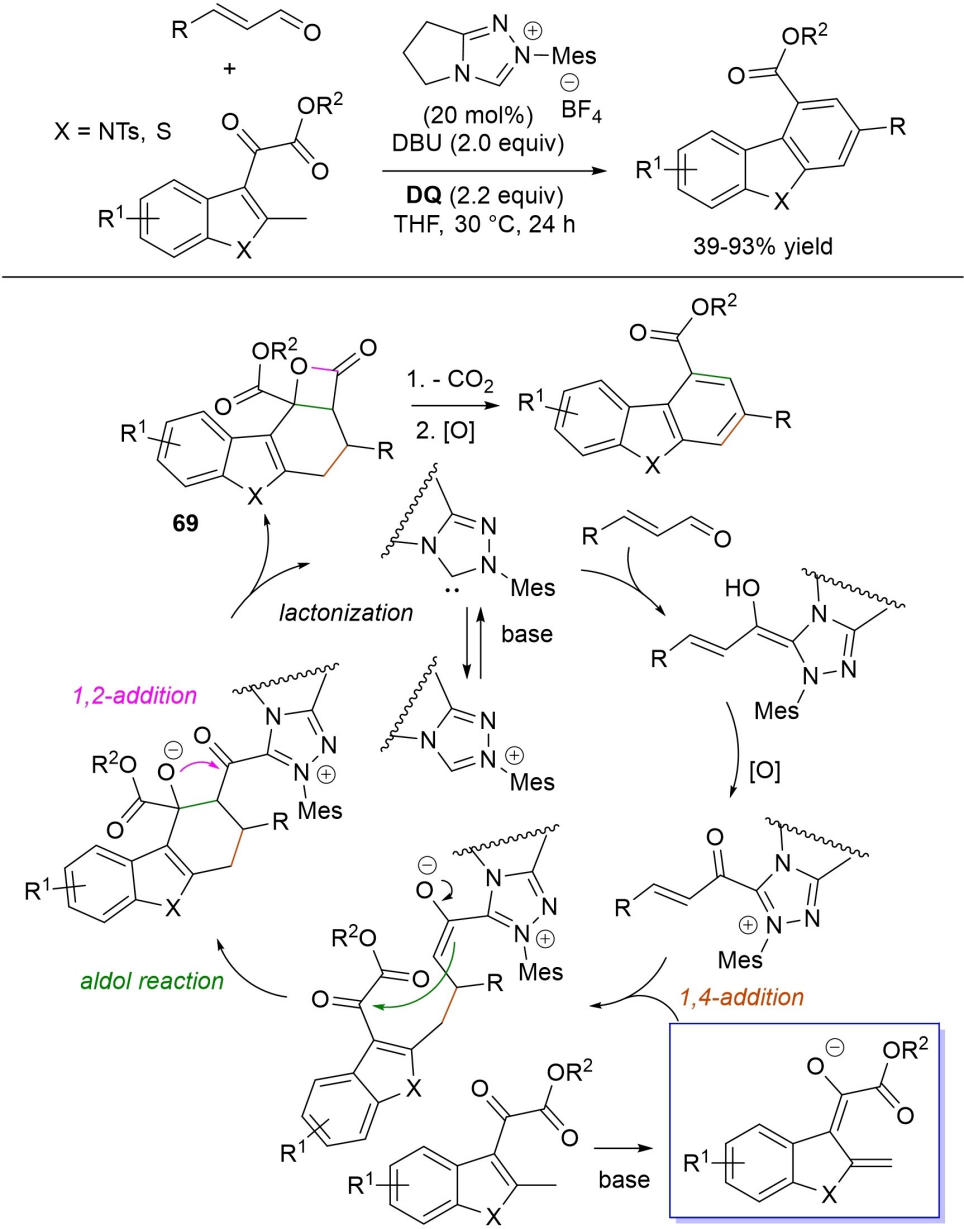
NHC‐catalyzed oxidative annulation of 2‐methyl‐3‐oxoester indoles with enals.

Carbazole skeletons were assembled from enals and *N*‐tosyl 2‐methyl‐3‐oxoacetate indoles, under the help of *N*‐mesityl‐substituted pyrrolidine‐based triazolium pre‐catalyst (20 mol%), DBU (2.0 equiv.) and **DQ** (2.2 equiv.) (Scheme 70).^[103]^ In respect of the enal substrates, the presence at β‐position of aryl moieties with electronically and sterically different groups, as well as heteroaromatic rings, styryl, alkyl and indolyl/carbazolyl groups was well supported (39–93 % yield), and so were diverse substitution arrangements of the indole companion, including replacement of methyl ester with ethyl‐, isopropyl, and benzyl ones (50–89 % yield). It is worthy of remark that the protocol was suitable for broad‐scale preparation (5 mmol of indole, 79 % yield), and could be further extended to 2‐methyl‐3‐oxoacetate benzo[*b*]thiophene, with the related dibenzo[*b,d*]thiophene obtained in 64 % yield.

The annulation process is believed to initiate by reaction of the nucleophilic indole enolate with the in situ generated α,β‐unsaturated acyl azolium, and the resultant adduct becomes converted to the fused β‐lactone **69**, that generates the carbazole derivative through CO_2_ release and oxidative aromatization.

**Scheme 71:**
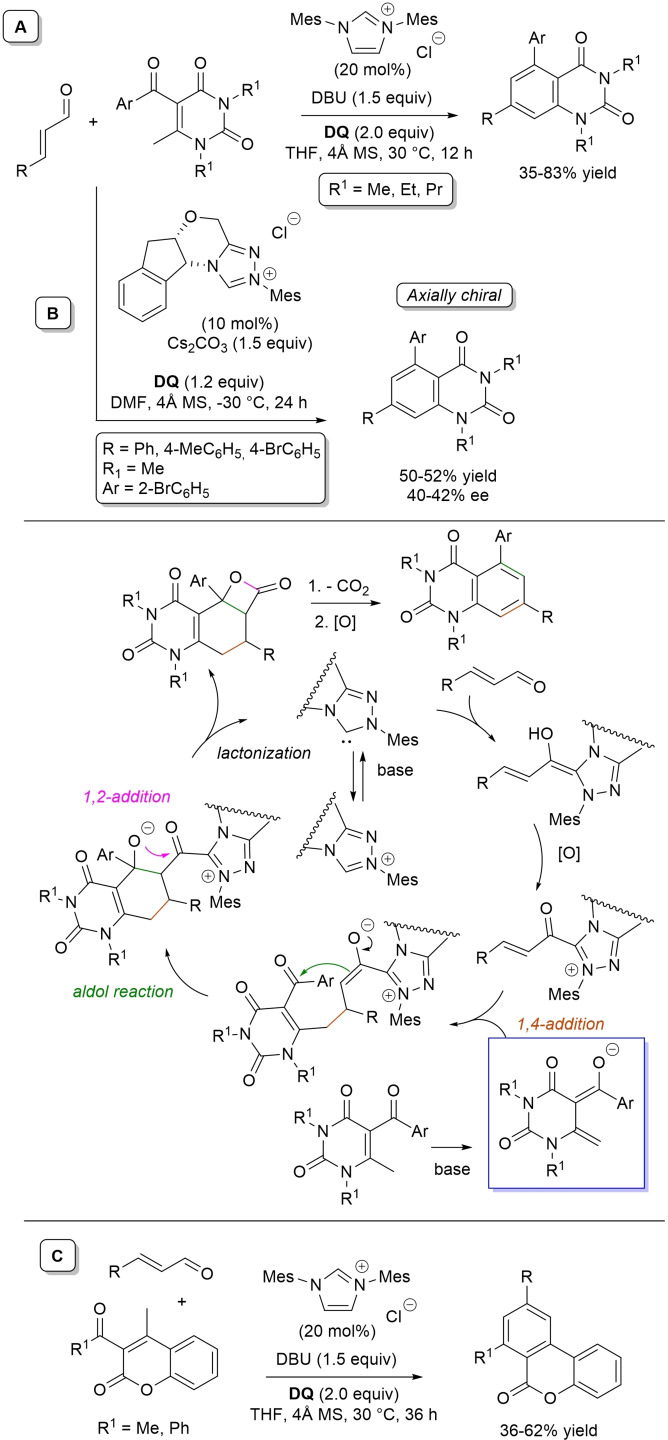
NHC‐catalyzed oxidative annulation of enals with pyrimidine‐2,4‐diones (**A**,**B**) and substituted coumarins (**C**).

Very recently, vinylogous Michael addition to alkenyl acyl azoliums at the hands of dienolates engendered from pyrimidine‐2,4‐diones has been envisioned as the entry point of a cascade transformation foreseeing intramolecular aldol reaction and lactonization as successive stages.^[104]^ This strategy provided access to β‐lactone intermediates, suitable precursors of quinazoline‐2,4‐diones (Scheme 71).

Unsubstituted and substituted cinnamaldehydes, β‐naphthyl/heteroaryl/styryl enals, and variedly *N*‐protected/benzoyl‐substituted pyrimidine diones were very proper substrates for reactions in THF promoted by 1,3‐dimesityl imidazolium chloride (20 mol%) and DBU (1.5 equiv.), in the presence of **DQ** (2.0 equiv.) (Scheme 71A). This approach was feasible for upscaling experiments (1.0 mmol scale, 69 % yield), and could be implemented to obtain heterocyclic congeners with axially chiral C−C bond by switching to the catalytic system formed by aminoindanol‐derived chiral triazolium salt as the NHC pre‐catalyst (10 mol%) and Cs_2_CO_3_ as the base (1.5 equiv.), in DMF solvent (Scheme 71B).

It should also be said that the same conditions used for the preparation of quinazoline‐2,4‐diones proved to be effective for synthesizing benzochromen‐6‐ones from 3‐acetyl‐ and 3‐benzoyl‐substituted 4‐methylcoumarins and β‐aryl/heteroaryl/alkyl/alkenyl enals (Scheme 71C).

**Scheme 72:**
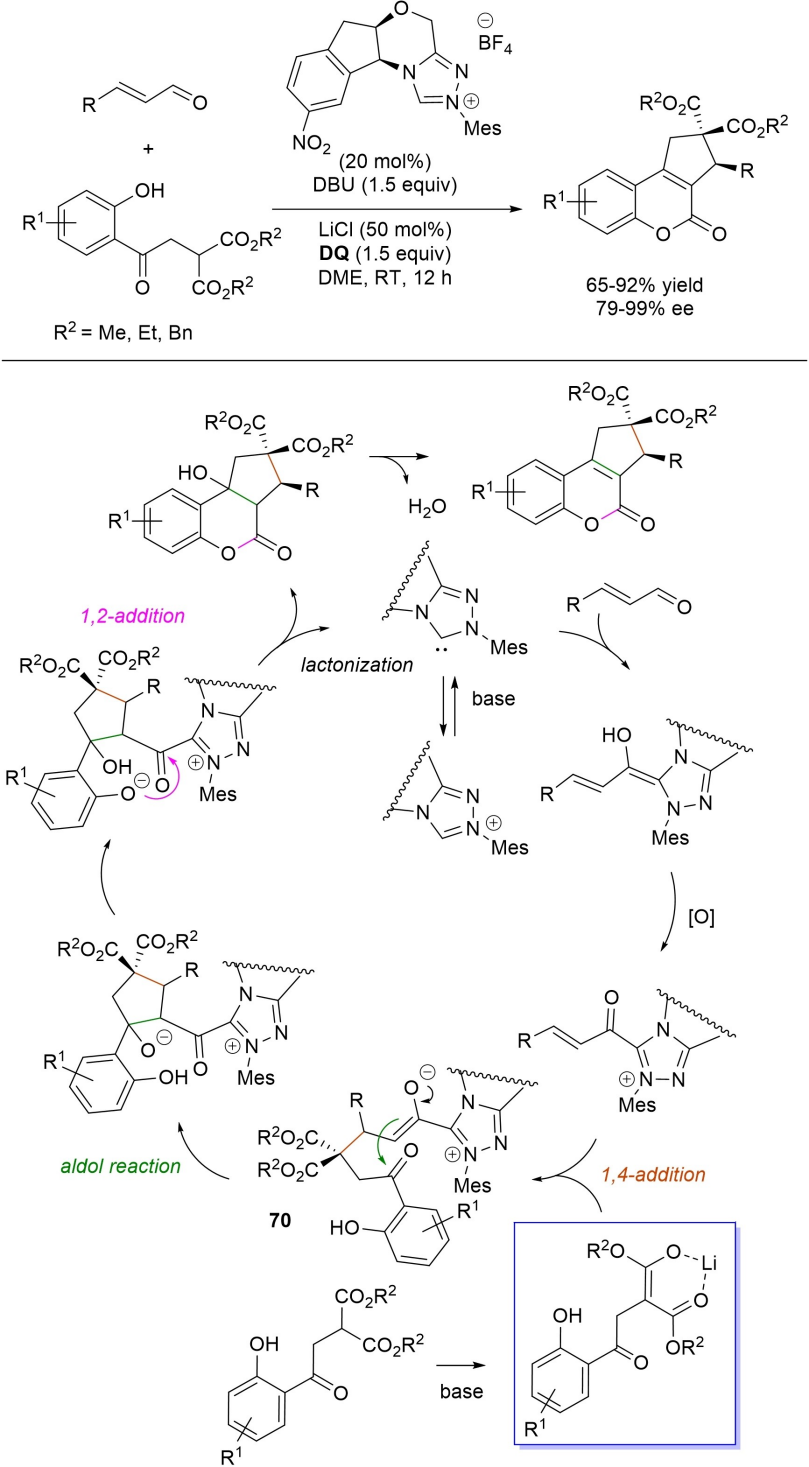
NHC‐catalyzed oxidative annulation of *o*‐hydroxyaryl malonates with enals.

Illustration of use of an α,β‐unsaturated acyl azolium with reaction partners which bear four various reactive centres came from Enders group.^[105]^ In the present case, enals carrying (hetero)aromatic, naphthyl, and alkenyl groups at β‐position were reacted with *o*‐hydroxyaryl malonates to trigger domino Michael/aldol/lactonization/dehydration processes for the NHC‐catalyzed asymmetric synthesis of cyclopenta[*c*]‐fused chromenones in oxidative conditions. For this purpose, nitro‐substituted chiral tetracyclic triazolium salt (aminoindanol structure, 20 mol%) was effective together with DBU (1.5 equiv.), LiCl (50 mol%) and **DQ** (1.5 equiv.) (Scheme 72) in giving the tricyclic products with good yields (65–92 %) and good to high enantioselectivities (79–99 % ee).

Consistent with DFT calculations, control experiments and the azolium homoenolate route provided by Biju and co‐workers for the annulation of enals with *o*‐hydroxychalcones,^[106]^ a well‐grounded mechanism for the synthesis of cyclopenta[*c*]‐fused chromenones calls for conjugate addition of malonate enolate to the NHC‐derived unsaturated acyl azolium to give **70**, followed by intramolecular aldol reaction, H‐shift and δ‐lactonization, with final regeneration of active NHC. A last dehydration stage completes the way towards the wanted molecules, potential starting points for enantioenriched all‐*trans*‐substituted cyclopentanes subsequent to reductive ring opening of the lactone moiety.

NHC‐catalyzed oxidative cascade reactions forging two C−C bonds and one C−O bond through Michael/Michael/lactonization sequences were planned by the use of two different Michael acceptors as reaction components.

**Scheme 73:**
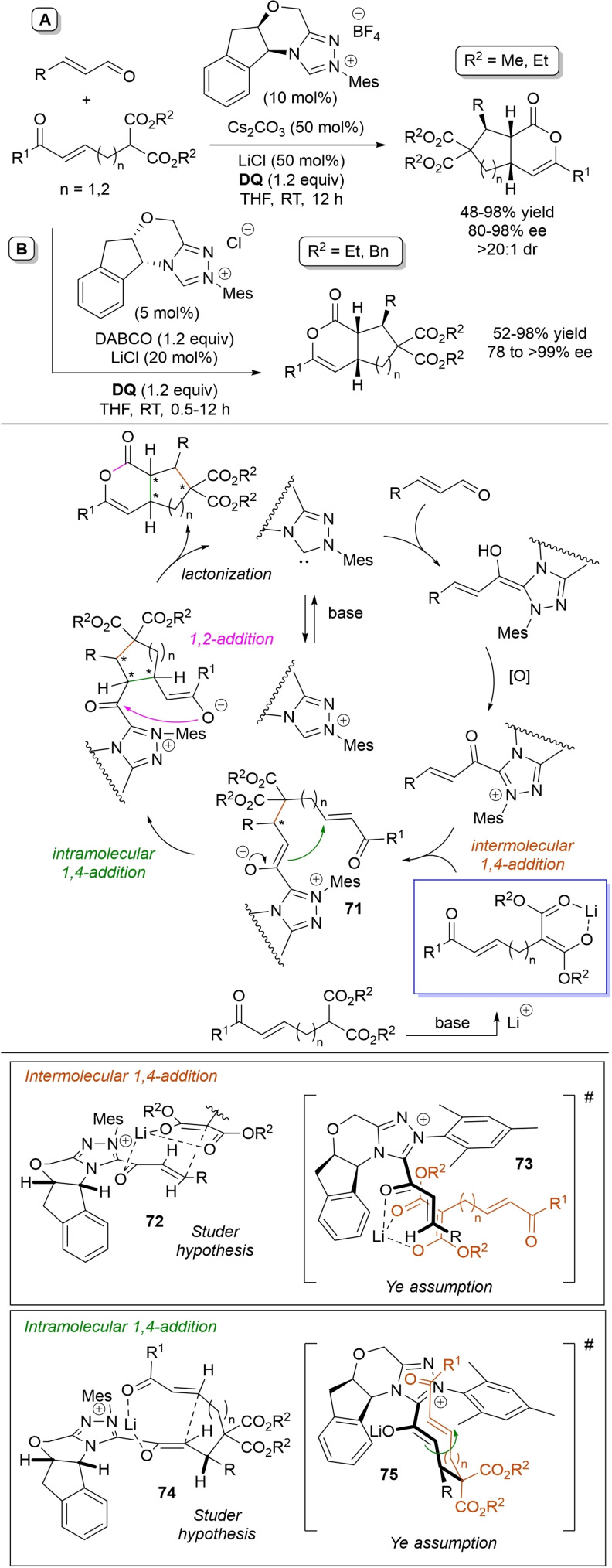
NHC‐catalyzed oxidative annulation of unsaturated ketomalonates with enals.

In 2015, this kind of processes were independently reported by the Ye^[107]^ and Studer^[108]^ groups for the enantioselective synthesis of bicyclic δ‐enollactones (iridoid core structures) with three adjacent stereocentres: enals and ϵ‐oxo‐γ,δ‐unsaturated malonates were involved in chiral NHC/Lewis acid cooperative catalysis^[76]^ to obtain almost the same cyclopentane‐ and cyclohexane‐fused δ‐lactones, but as opposite enantiomeric products, as a result of each author using optical antipodes of the same carbene catalyst (Scheme 73).

In Ye work,^[107]^ cinnamylmethyl malonates and cinnamylethyl malonates were reacted with β‐aryl/heteroaryl/alkenyl/alkyl enals using the triazolium NHC pre‐catalyst derived from (1*S*,2*R*)‐1‐amino‐2‐indanol (10 mol%), in combination with Cs_2_CO_3_ (50 mol%), LiCl (50 mol%) and **DQ** (1.2 equiv.) (Scheme 73A). The target heterocyclic products were obtained in fair to high yields (50–98 %), very good enantioselectivities (80–94 % ee) and excellent diastereoselectivity (>20 : 1 dr), and very similar results occurred using a methylmalonate homologue with one more carbon atom (48–96 % yield, 88–98 % ee).

Shortly after, Studer and co‐workers found that the system formed by (1*R*,2*S*)‐aminoindanol‐derived triazolium pre‐catalyst (5 mol%), DABCO (1.2 equiv.), LiCl (20 mol%) and **DQ** (1.2 equiv.) fostered completely diastereoselective reactions of cinnamylethyl malonates and cinnamylbenzyl malonates with β‐aryl/heteroaryl/alkyl enals (52–97 % yield, 78–93 % ee) (Scheme 73B).^[108]^ In such a method, use of an ethylmalonate homologue (one more carbon atom, methyl ketone) showed better reactivity (76–98 % yield) and selectivity (94 to >99 % ee).

Whatever the catalyst used, the plausible catalytic cycle consists of an initial Michael addition of malonate enolate to the α,β‐unsaturated acyl azolium, the new generated enolate **71** engaging in a Michael‐type cyclization which assembles the cyclopentane or cyclohexane ring part. At this point, *O*‐acylation/fragmentation regenerates the NHC catalyst finalizing the lactone products. It should be underlined that two different pathways, namely cascade nucleophilic 1,2‐addition/Claisen rearrangement/Michael addition/lactonization^[87a]^ and Michael addition/*endo*‐hetero‐Diels‐Alder reaction were not excluded.

The stereochemical control in the bicyclic δ‐enollactones forming reaction is thought to be operated by contemporaneous coordination of the unsaturated acyl azolium and enolate by lithium ion, which directs the enolate to attack the Michael acceptor from the sterically less impeded face. On the point, model **72** and transition state **73** have been postulated by Studer and Ye, respectively. Added to this is the fact that *cis*‐selectivity of the second (intramolecular) Michael addition may be determined by lithium ion complexation of both enolate and the oxygen atom of the enone acceptor (model **74**, Studer hypothesis), but it is not excluded that a chair‐type conformation of the intermediate enolate is operating (transition state **75**, Ye assumption). And, no less important, the higher selectivities observed for the 6,6‐bicyclic products in Studer work maybe result from faster cyclization of the major diastereomeric enolate formed in the intramolecular Michael addition, the minor one probably undergoing a retro‐Michael addition.

**Scheme 74:**
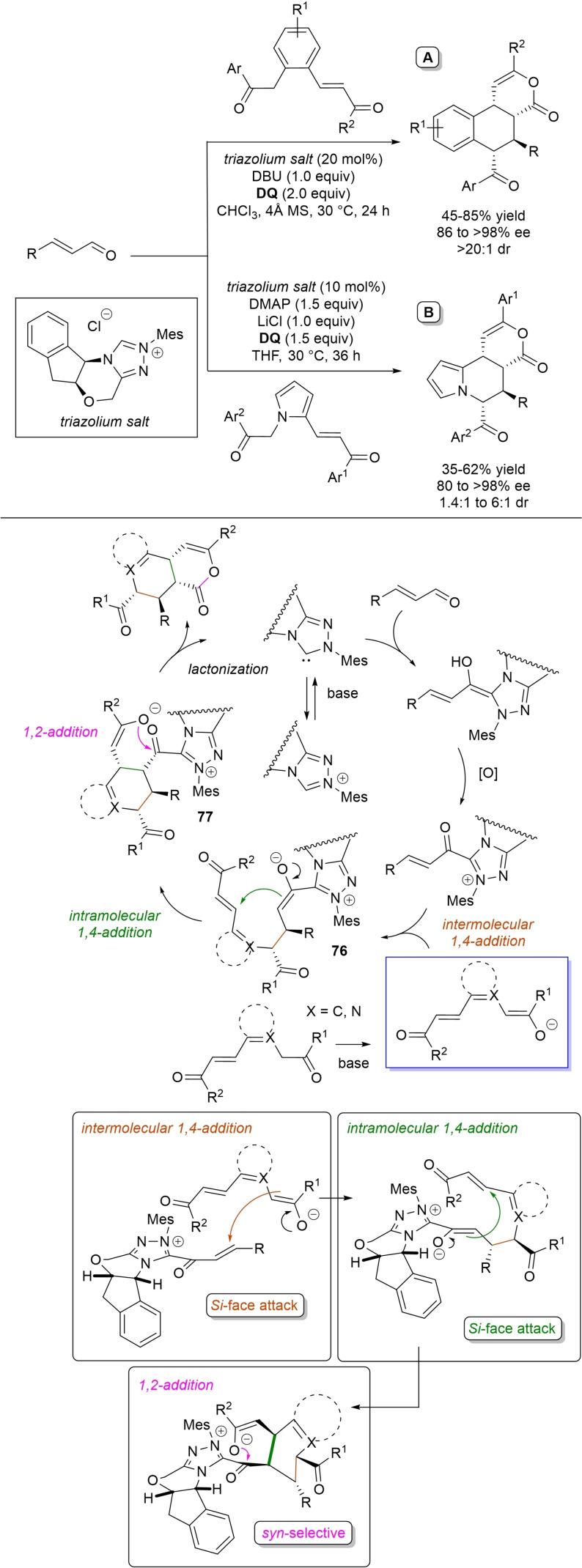
NHC‐catalyzed oxidative annulation of β‐(hetero)aryl enones with enals.

In 2021, Biju and co‐workers have made use of a NHC‐catalyzed oxidative Michael/Michael/lactonization cascade reaction to demonstrate the asymmetric synthesis of tricyclic enollactones with tetraline and tetrahydroindolizine structure (four adjoining stereocenters).^[109]^ To that end, α,β‐unsaturated aldehydes having (hetero)aryl, naphthyl, and styryl groups at β‐position were put in presence of suitable β‐aryl enones, aminoindanol‐based chiral triazolium salt (20 mol%), DBU (1.0 equiv.), and **DQ** (2.0 equiv.) (Scheme 74A), with optically enriched products obtained with yields of 45–85 % and very good to excellent enantioselectivities (86 to >98 % ee). These represented convenient starting points for tetrasubstituted tetralines through elaboration of the lactone moiety, that is ring opening with *O*‐ and *N*‐nucleophiles (i. e., methanol, benzylamine) and one‐pot ring contraction in oxidative conditions.

Paralleled studies have enabled pyrrole‐based enones to react with enals in slightly different conditions, thus with half amount of pre‐catalyst (10 mol%), DMAP as base (1.5 equiv.), LiCl as additive (1.0 equiv.), and 1.5 equiv. of the oxidant (Scheme 74B). δ‐Lactone‐fused indolizines were derived in moderate yields (35–62 %) and diastereoselection (1.4 : 1 to 6 : 1 dr), in contrast enantioselectivities were excellent (80 to >98 % ee).

When it comes to the reaction pathway, the starting conjugate addition of the in situ born ketone enolate to the α,β‐unsaturated acyl azolium generates NHC‐bound enolate **76**, its successive Michael cyclization leading to the formation of the acyl azolium enolate **77** for the ending lactonization. Analogously as seen for annulation reactions of enals with *N*‐substituted 2‐(trifluoroacetyl)pyrroles via domino Michael/aldol/lactonization,^[98]^ *Si*‐face intermolecular (first) conjugate addition is favored for steric reasons, which is responsible for the stereochemistry of Michael adduct **76**. This steers the *Si*‐face intramolecular (second) Michael addition pathway and *syn*‐selective lactonization (this latter mode of stereoselection may come from a highly *cis*‐selective hetero Diels‐Alder reaction, too).

**Scheme 75:**
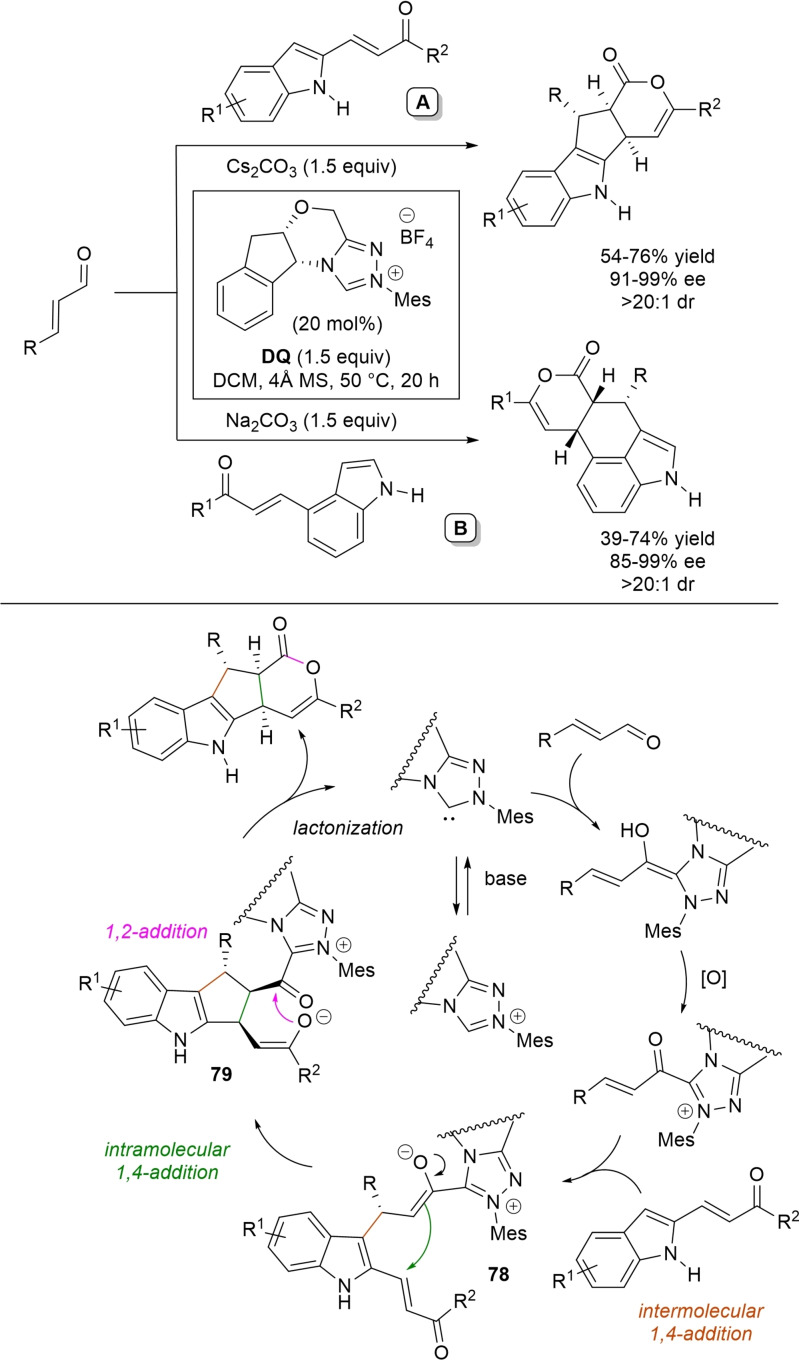
NHC‐catalyzed oxidative annulation of indole enones with enals.

An α,β‐unsaturated acyl azolium was the key intermediate in Michael/Michael/lactonization processes initiated by a *C*‐nucleophile other than typical stabilized carbanions (enolates), in which case indole enones were selected as the partners of enal substrates.^[110]^ Depending on the positioning of the enone portion, reactions performed with aminoindanol‐derived chiral triazolium salt (20 mol%), Cs_2_CO_3_ or Na_2_CO_3_ (1.5 equiv.) and **DQ** (1.5 equiv.) gave 1,2,3,4‐tetrahydrocyclopenta[*b*]indoles (Scheme 75A) and 1,3,4,5‐tetrahydrobenzo[*cd*]indoles (Scheme 75B) with built‐in enollactone nucleus. A wide range of substituent combinations on enal and indole enone (i. e., aryl/aryl, aryl/heteroaryl, heteroaryl/heteroaryl, aryl/alkyl, heteroaryl/alkyl, alkyl/alkyl) have been shown to be effective in providing the tetracyclic heterocyclic products (39–76 % yield) with excellent stereoselectivities (85–99 % ee, >20 : 1 dr).

Thanks to a series of control experiments, well‐founded pathways for the assemblage of both type of indole compounds have been offered. As shown in the mechanism of formation of 1,2,3,4‐tetrahydrocyclopenta[*b*]indoles as representative example, the beginning is a Friedel‐Crafts alkylation at position C3 of indole via conjugate addition to the enal‐derived α,β‐unsaturated acyl azolium. The enolate **78** which is formed sets off the cascade Michael/lactonization sequence bringing to the polyannulated products through enolate **79**.

## 4 NHC‐Bound Ortho‐Quinone Methide, Aza‐Fulvene, and Triaza‐Diene Intermediates

**Scheme 76:**
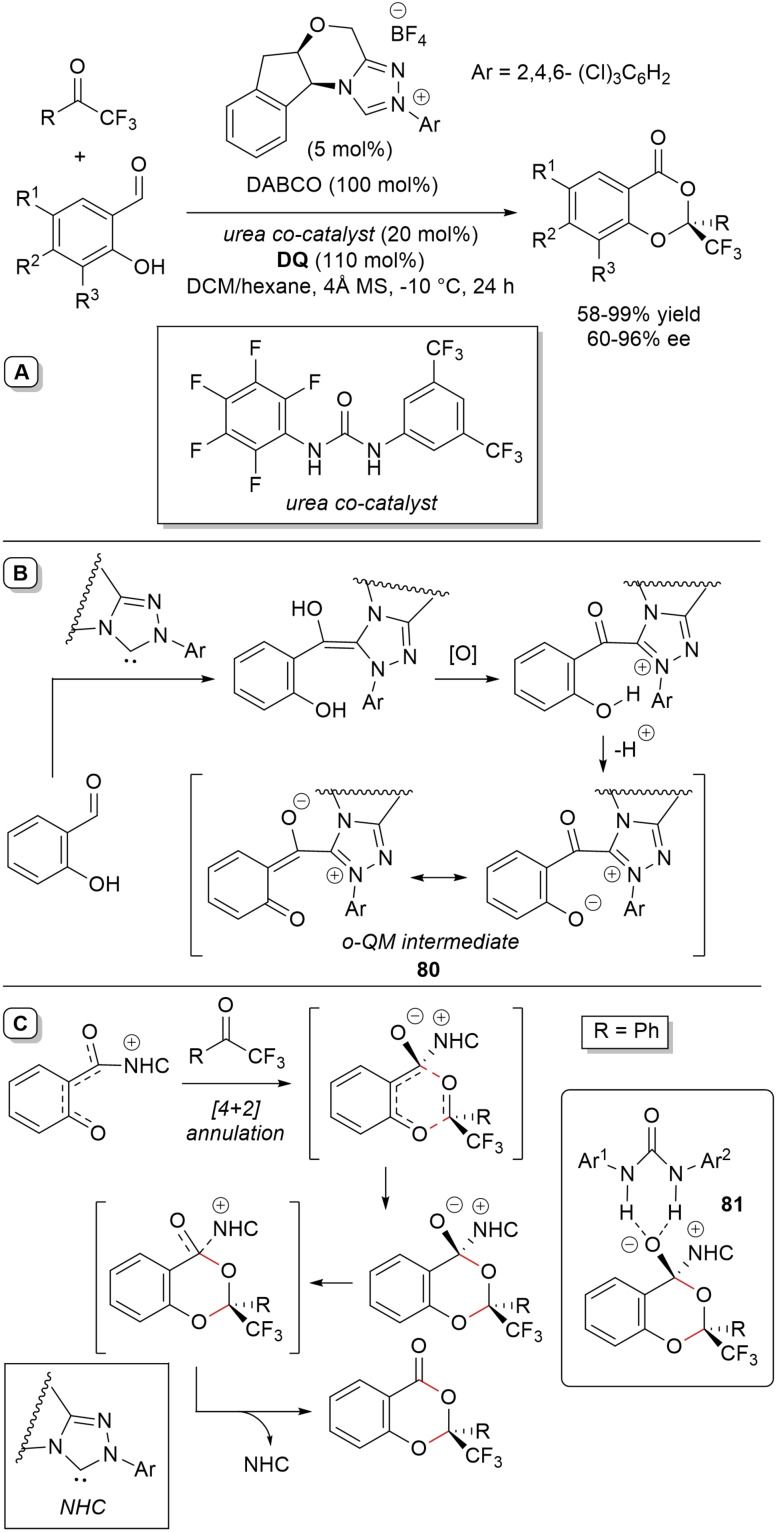
Synthesis of ketal‐like adducts via NHC‐bound *o*‐QM intermediate.

In 2017, the group of Hirao and Chi realized a new mode of NHC‐catalysis relying on NHC‐bound *ortho*‐quinone methide (*o*‐QM) intermediate for remote activation of oxygen atom as reactive centre.^[111]^ Such a strategy is found in the reactions of 2‐hydroxy aryl aldehydes with (hetero)aryl/alkyl trifluoromethyl ketones promoted by aminoindanol‐derived chiral triazolium salt (5 mol%), DABCO (100 mol%), achiral urea co‐catalyst (20 mol%), and **DQ** (110 mol%), which gave chiral products (60–96 % ee) with a ketal‐like structure (Scheme 76A). Then again, this method has been shown to be applicable to large‐scale preparations with only 1 mol% of catalyst loading, and moreover catalytic **DQ** (10 mol%) could be used combined with MnO_2_ (500 mol%) as the terminal oxidant.^[112]^

As shown in Scheme 76B for simple salicylaldehyde as the model example, once the Breslow intermediate is formed from the aromatic aldehyde and NHC, oxidation leads to the corresponding acyl azolium species which loses O−H proton: this results into the pivotal *o*‐QM species **80** appointed to the annulation reaction with the ketone component.

Very thorough DFT computations on the reaction between salicylaldehyde and trifluoromethyl phenyl ketone have made clear that the annulation process proceeds through a [4+2] mechanism consisting of two concerted steps, which is to say ring formation to which follows the (rate‐determining) NHC dissociation (Scheme 76C). For this last event, calculations in the presence of urea gave the lowest‐energy (favoured) transition state **81** (related to the major product enantiomer), which involves hydrogen bond interactions with the carbonyl oxygen atom of *o*‐QM and benefits from attractive π‐π stacking between the catalyst indane skeleton and the urea pentafluorophenyl group. As well as this, prominent attractive dispersion interactions between the ketone phenyl moiety and the urea group have been demonstrated, which likely determine the observed enantioselectivity.

**Scheme 77:**
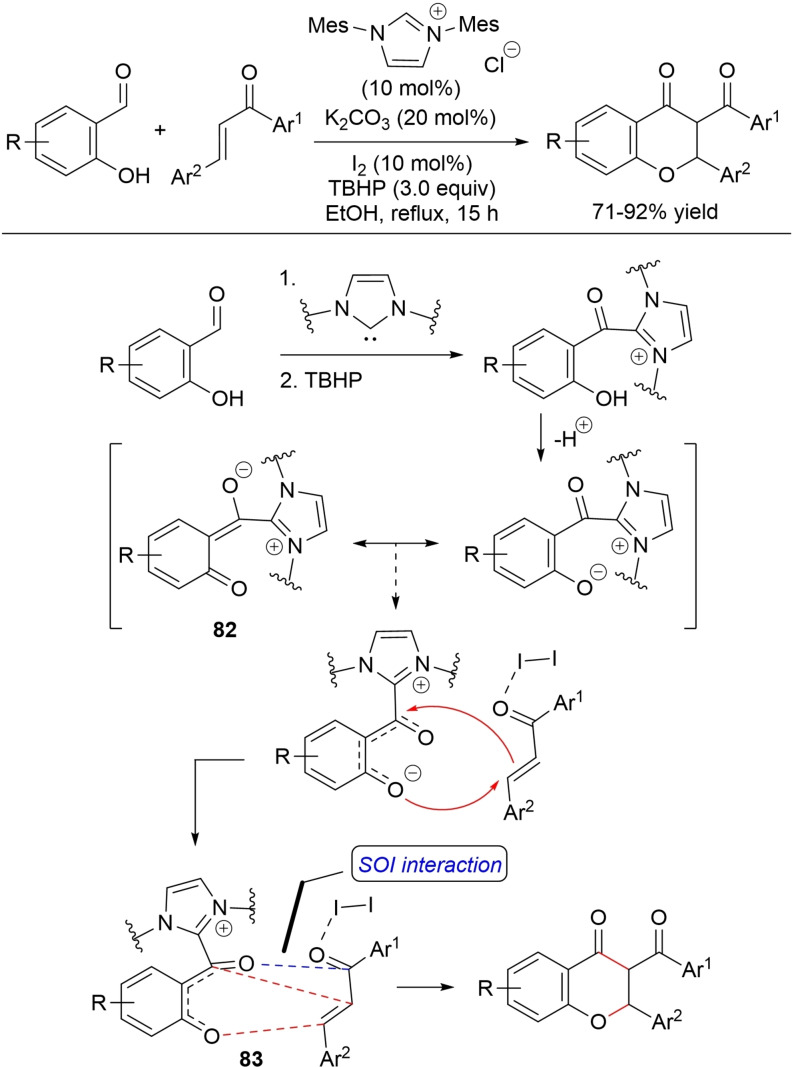
Synthesis of 3‐benzoyl flavanones via NHC‐bound *o*‐QM intermediate.

The potential of the approach based on remote OH activation of 2‐hydroxy aryl aldehydes by oxidative NHC‐catalysis has been recently tapped for the synthesis of bicyclic 3‐benzoyl flavanones.^[113]^ In that regard, salicylaldehyde and substituted analogues were made to react with chalcones by resorting to 1,3‐dimesityl imidazolium chloride (10 mol%), K_2_CO_3_ (20 mol%), I_2_ (10 mol%) as co‐catalyst, and TBHP oxidant (3.0 equiv.) (Scheme 77), with the annulated adducts (including a spiro one) obtained in good to excellent yields (71–92 %).

By analogy to Hirao and Chi findings,^[111]^ it may be deemed that the [4+2] cyclization with NHC‐bound *o*‐QM **82** comes from preferential attack of its *O*‐nucleophilic centre to the β‐carbon of the chalcone acceptor, seeing that the originated intermediate **83** would experience secondary orbital interaction (SOI),^[114]^ in cooperation with NHC/I_2_ co‐catalysis.

**Scheme 78:**
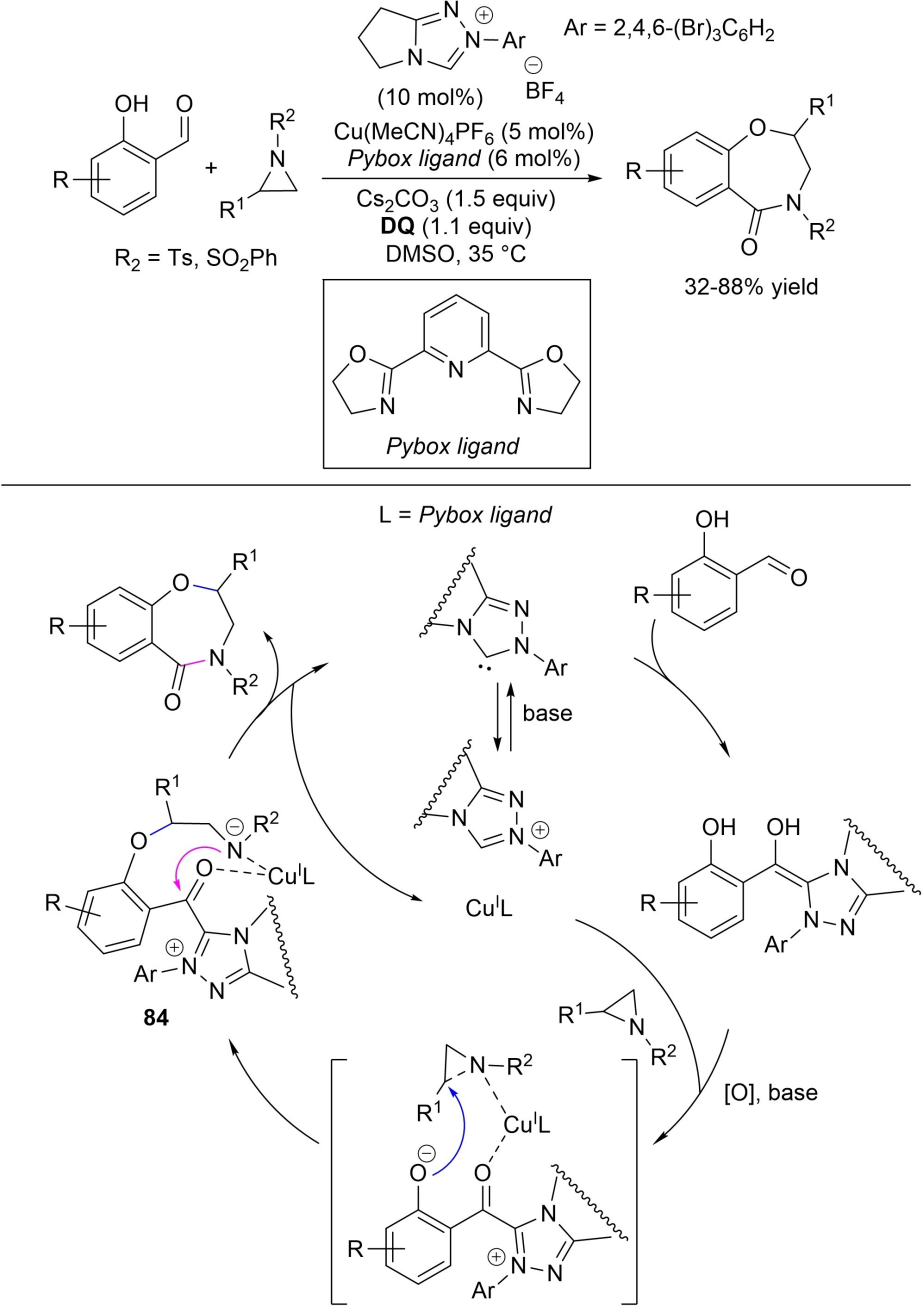
Synthesis of 1,4‐benzoxazepinones via NHC‐bound *o*‐QM intermediate under copper (I) co‐catalysis.

Recently, *o*‐QM intermediates have been involved in [4+3] annulation reactions with aziridines under copper (I) co‐catalysis.^[115]^ Going into detail, 2‐(hetero)aryl‐substituted *N‐*tosyl (benzenesulfonyl) aziridines could be reacted with mono‐ and disubstituted salicylaldehydes in the presence of *N*‐2,4,6‐tribromophenyl‐substituted triazolium pre‐catalyst (10 mol%), Cs_2_CO_3_ (1.5 equiv.), **DQ** (1.1 equiv.), Cu(MeCN)_4_PF_6_ (5 mol%), and Pybox ligand (6 mol%) (Scheme 78), giving 1,4‐benzoxazepinone products in moderate to good yields (32–88 %) and complete regioselectivity.

These conditions were suitable for gram‐scale preparation, retained efficiency (78 % yield) being demonstrated for the coupling between 2‐phenyl‐*N*‐tosyl aziridine (4.0 mmol) and salicylaldehyde (2.0 equiv.).

In all likelihood, formation of *o*‐QM species is followed by coordination of Cu(I) with both carbonyl group and aziridine, which is now activated against nucleophilic ring opening (attack on the more‐substituted carbon). The resultant intermediate **84** gives rise to lactamization to afford the annulation product, releasing the NHC catalyst.

**Scheme 79:**
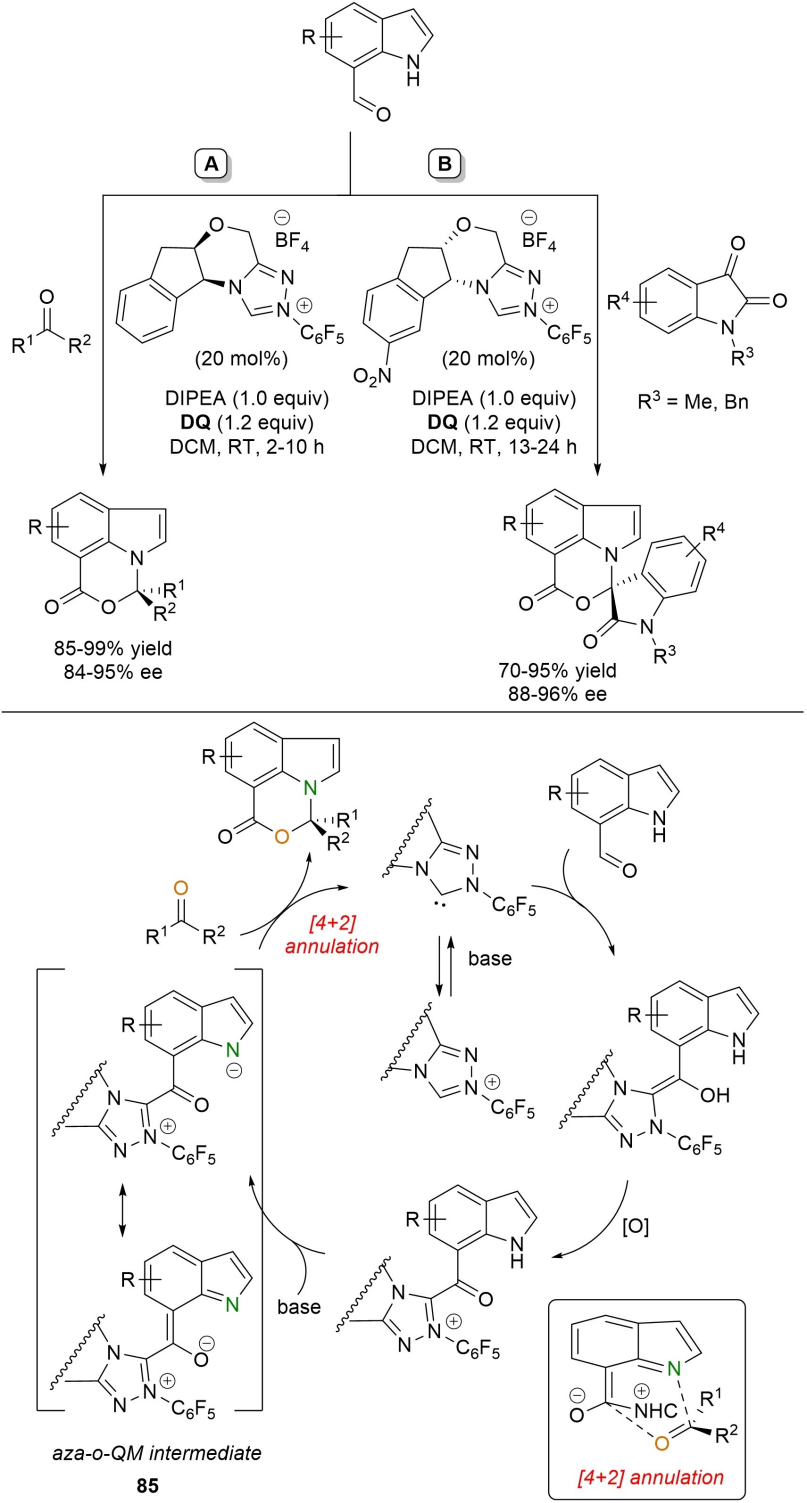
Annulation of indole‐7‐carboxaldehydes with ketones via NHC‐bound *aza*‐*o*‐QM intermediate.

Addition of NHC catalyst to the aldehyde moiety of indole‐7‐carboxaldehydes, followed by oxidation and deprotonation opened the way to analogous *aza*‐*o*‐QM intermediates **85**,^[116]^ aimed at use in formal [4+2] annulation reactions with ketones leading to pyrroloquinazoline or oxazinoindole products incorporating a *N*,*O*‐acetal unit (Scheme 79).^[117]^ Trifluoromethyl ketones with aromatic/aliphatic groups, difluoromethyl ketones and α‐ketoesters were reacted with indole‐7‐carboxaldehydes having substitutions at 3‐, 4‐, 5‐, and 6‐positions, with the system formed by *N*‐pentafluorophenyl‐substituted aminoindanol‐derived chiral triazolium pre‐catalyst (20 mol%), Hünig's base (1.0 equiv.) and **DQ** (1.2 equiv.) found as the most efficient one for obtaining the target products in excellent yields (85‐99 %) and enantioselectivities (84–95 % ee) (Scheme 79A). It is interesting to observe that the possible use of an imine as the electrophile has been demonstrated in the exact same conditions, while a scale‐up test using 6‐chloro indole‐7‐carboxaldehyde/trifluoroacetophenone couple gave comparable results (94 % yield, 93 % ee) by using 5 mol% catalyst. And then, similar reactions were carried out with *N*‐substituted isatins, enabling the construction of spiro‐cyclic *N*,*O*‐acetals (70–95 % yield, 88–96 % ee) on condition that nitro‐substituted aminoindanol‐based chiral triazolium was used as the NHC precursor (Scheme 79B).

**Scheme 80:**
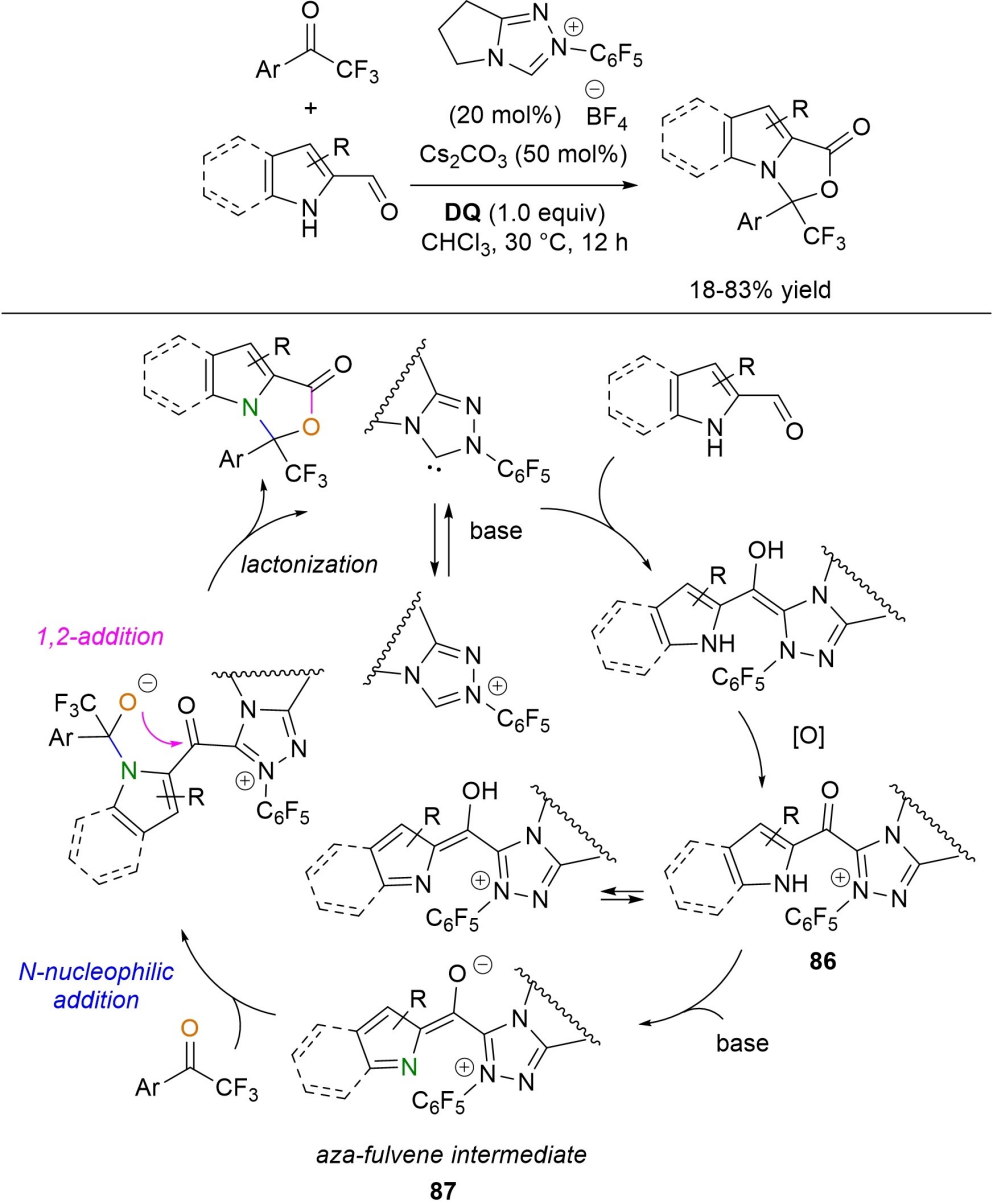
Reaction of *N*‐heterocyclic aldehydes with ketones via *aza*‐fulvene type intermediate.

Activation of the nitrogen atom of indole‐ and/or pyrrole‐2‐carboxaldehydes by oxidative NHC‐catalysis went through base‐promoted conversion of the initially formed acyl azolium species **86** (or its enol tautomer) into cross‐conjugated *aza*‐trienolate (*aza*‐fulvene type) intermediates of general formula **87** (Scheme 80).^[118]^ These were engaged in cyclization reactions with ketones to obtain *N*,*O*‐acetal products: a stepwise route most likely occurs involving *N*‐nucleophilic addition of the NHC‐bound *aza*‐fulvene to the ketone carbonyl group followed by intramolecular acylation/fragmentation (lactonization). This mechanistic assumption was provided by Biju, Jindal and co‐workers under cover of DFT studies on the reactions between the *N*‐heterocyclic aldehydes and α,α,α‐trifluoro (hetero)aryl ketones with achiral *N*‐perfluorophenyl‐substituted triazolium pre‐catalyst (20 mol%), Cs_2_CO_3_ (50 mol%), and equimolar **DQ**, giving *N*,*O*‐acetals in poor to good yields (18–83 %).

**Scheme 81:**
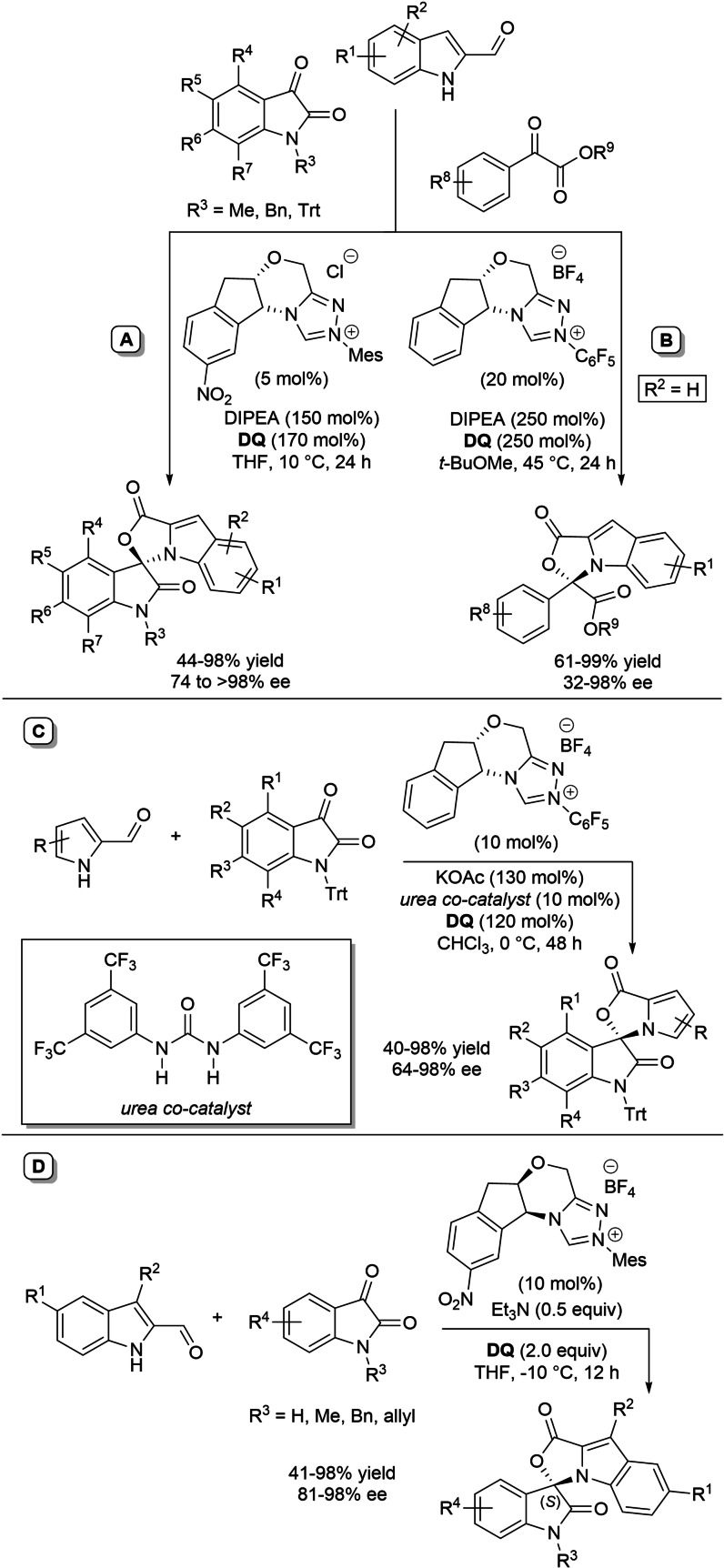
NHC‐catalyzed oxidative cyclization of *N*‐heterocyclic aldehydes with isatins and aryl α‐ketoesters.

In terms of NHC‐catalyzed oxidative asymmetric reactions, a large collection of enantioenriched *N*,*O*‐acetal indolines (44–98 % yield, 74 to >98 % ee) was obtained by the group of Jin through treatment of indole‐2‐carboxaldehyde and its derivatives (substituted on the benzene ring) with *N*‐protected isatins,^[119]^ with best conditions identified by using nitro‐substituted aminoindanol‐based chiral triazolium pre‐catalyst (5 mol%), Hünig's base (150 mol%) and **DQ** (170 mol%), in THF solvent (Scheme 81A). Equally, aryl α‐ketoesters were employed as electrophiles (61‐99 % yield, 32–98 % ee), but slightly adjusted conditions were needed employing *N*‐pentafluorophenyl aminoindanol‐derived chiral triazolium salt (20 mol%), greater amounts of both base and **DQ** (250 mol% each), and *tert*‐butyl methyl ether as the reaction medium (Scheme 81B).

Speaking instead of pyrrole‐2‐carboxaldehydes, their reaction with *N*‐trityl isatins was run under diverse conditions, which called for cooperative use of 1,3‐bis[3,5‐bis(trifluoromethyl)phenyl]urea (10 mol%) and the NHC derived from *N*‐pentafluorophenyl aminoindanol‐derived chiral triazolium (10 mol%) and KOAc (130 mol%), along with **DQ** (120 mol%) (Scheme 81C). It is to be noted that this was the case for which the results more closely depended on both type and position of substituent on the pyrrole aldehyde nucleus, an electron‐withdrawing group on C5 causing deactivation of the aromatic *N*‐nucleophile.

An identical approach was put in place by Hui and co‐workers for NHC‐catalyzed oxidative reactions of indole 2‐carboxaldehydes with *N*‐protected isatins, using the enantiomer of the NHC pre‐catalyst previously adopted by Jin and co‐workers,^[119]^ under somewhat different conditions (Scheme 81D).^[120]^ Employing 10 mol% of the triazolium salt (BF_4_^−^ counterion) in conjunction with Et_3_N (0.5 equiv.) and **DQ** (2.0 equiv.) in THF solvent at low temperature (‐10 °C), cyclic *N*,*O*‐aminal indoles with (*S*)‐configuration at the spiro quaternary stereocentre were formed in excellent yields (41–98 %) and stereoselectivities (81–98 % ee).

**Scheme 82:**
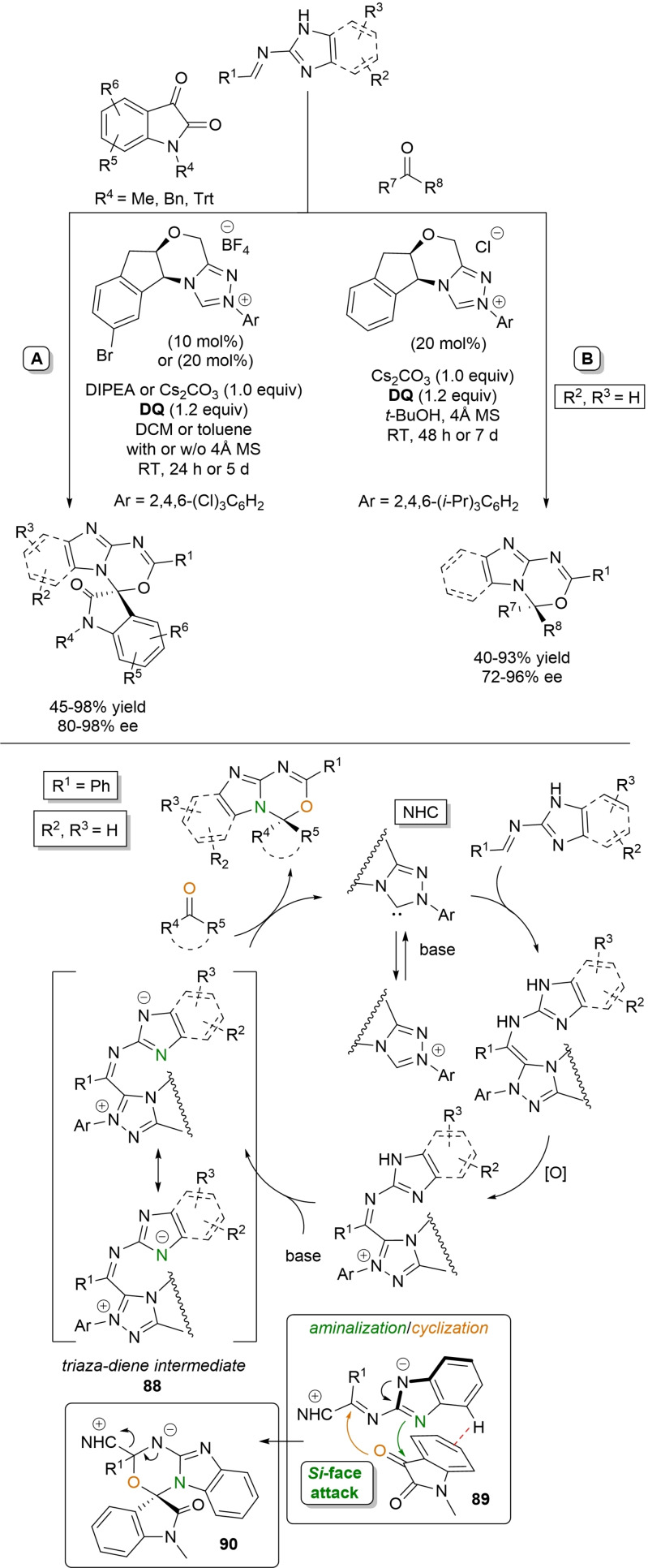
NHC‐catalyzed oxidative reaction of (benz)imidazole aldimines with ketones via triaza‐diene intermediate.

A further mode of remote *N*‐nucleophilic activation was achieved by a sequence of chiral NHC addition to (benz)imidazole‐based aldimines, oxidation and proton transfer to generate unusual triaza‐diene intermediates, then drawn into formal [4+2] ring‐forming reactions with ketone substrates for the synthesis of chiral non‐racemic polycyclic *N*,*O*‐acetals (Scheme 82).^[121]^

In close analogy to the works built around NHC‐bound *aza*‐*o*‐QM^[116]^ and *aza*‐fulvene intermediates,[^118^, ^119^, ^120^] both isatins (Scheme 82A) and acyclic ketones (Scheme 82B) have succeeded in reacting with the imines derived from 2‐amino(benz)imidazoles in a highly enantioselective fashion, giving a very broad range of polynitrogenated heterocyclic products (40–98 % yield, 72–98 % ee), and beyond that, inexpensive MnO_2_ could be used in lieu of **DQ** oxidant without any detrimental impact on yields and/or selectivities.

Supported on experimental investigations and DFT predictions pertaining to the reaction between 1‐methyl isatin and (*E*)‐*N*‐(1*H*‐benzo[*d*]imidazol‐2‐yl)‐1‐phenylmethanimine (benzaldehyde and 2‐aminobenzimidazole precursors), it was concluded that an imidoyl azolium species is actually formed (detection by high‐resolution mass spectrometry), then a smooth deprotonation occurs thanks to increased acidity of the remote N−H. It thus forms NHC‐bound triaza‐diene **88** that reacts with ketone partner through a concerted asynchronous addition (aminalization/cyclization sequence, transition state **89**), originating zwitterion **90** which expels NHC and forms the intended product. The observed stereocontrol comes as a result of *Si*‐face attack to isatin, displaying favourable dispersion energy between the parties involved in the ring‐forming reaction, due to CH‐π interaction among the isatin aromatic ring and the imine phenyl group.

**Scheme 83:**
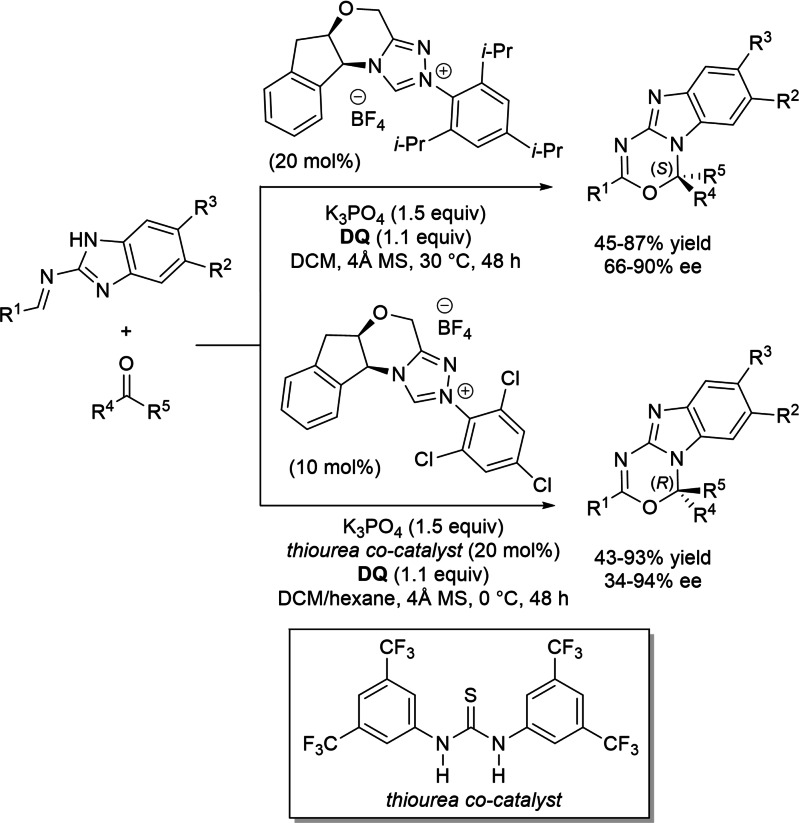
Oxidative NHC‐catalyzed enantiodivergent synthesis of heterocyclic fused *N*,*O*‐acetals.

Triaza‐diene intermediates fathered by benzimidazole‐structured aldimines and identically configured NHC catalysts have helped the enantiodivergent synthesis of heterocyclic fused tricyclic *N*,*O*‐acetals through 1,4‐dipolar cycloaddition with ketones.^[122]^ More to the point, *N*‐2,4,6‐triisopropylphenyl‐ and *N*‐2,4,6‐trichlorophenyl‐substituted chiral triazolium salts derived from (1*S*,2*R*)‐1‐amino‐2‐indanol were both used to promote the reaction of aldimines (derived from 2‐aminobenzimidazoles) with trifluoromethyl (hetero)aryl/alkyl ketones and phenyl ketones with CF_2_H, CF_2_Cl and CF_2_Br groups. Using the chiral triazolium pre‐catalyst with *N*‐2,4,6‐triisopropylphenyl substituent (20 mol%) jointly to K_3_PO_4_ (1.5 equiv.) and **DQ** (1.1 equiv.) gave products with (*S*)‐configuration at the quaternary stereocenter (45–87 % yield, 66–90 % ee), while the configurational stereoisomers (43–93 % yield, 34–94 % ee) were formed when *N*‐2,4,6‐trichlorophenyl‐substituted chiral triazolium salt (10 mol%) was used with 1,3‐bis[3,5‐bis(trifluoromethyl)phenyl]thiourea as a co‐catalyst (20 mol%) (Scheme 83).

The conditions used for the synthesis of (*R*)‐configured *N*,*O*‐acetals have proved effective even when using isatins as the ketone reagents, however it was not possible to realize the organocatalytic enantiodivergent protocol unlike acyclic ketones.

Here too, DFT simulations gave an indication of the key factors influencing the enantioselection. Taking the reaction of trifluoroacetophenone with the imine derived from 2‐aminobenzimidazole and benzaldehyde as the model, preferential formation of (*S*)‐stereoisomer (*N*‐nucleophilic attack from the ketone *Si*‐face) was confirmed, also as an effect of C−H‐ ‐ ‐F hydrogen bond interactions in the associated transition state. Weakening of these forces (by introduction of Cl groups in the catalyst structure) has been shown to cause a switch in enantioselectivity, these results altogether confirming the need for a CF_3_ moiety to realize the enantiodivergent strategy.

## 5 Non‐Acyl/Imidoyl Azolium Intermediates

**Scheme 84:**
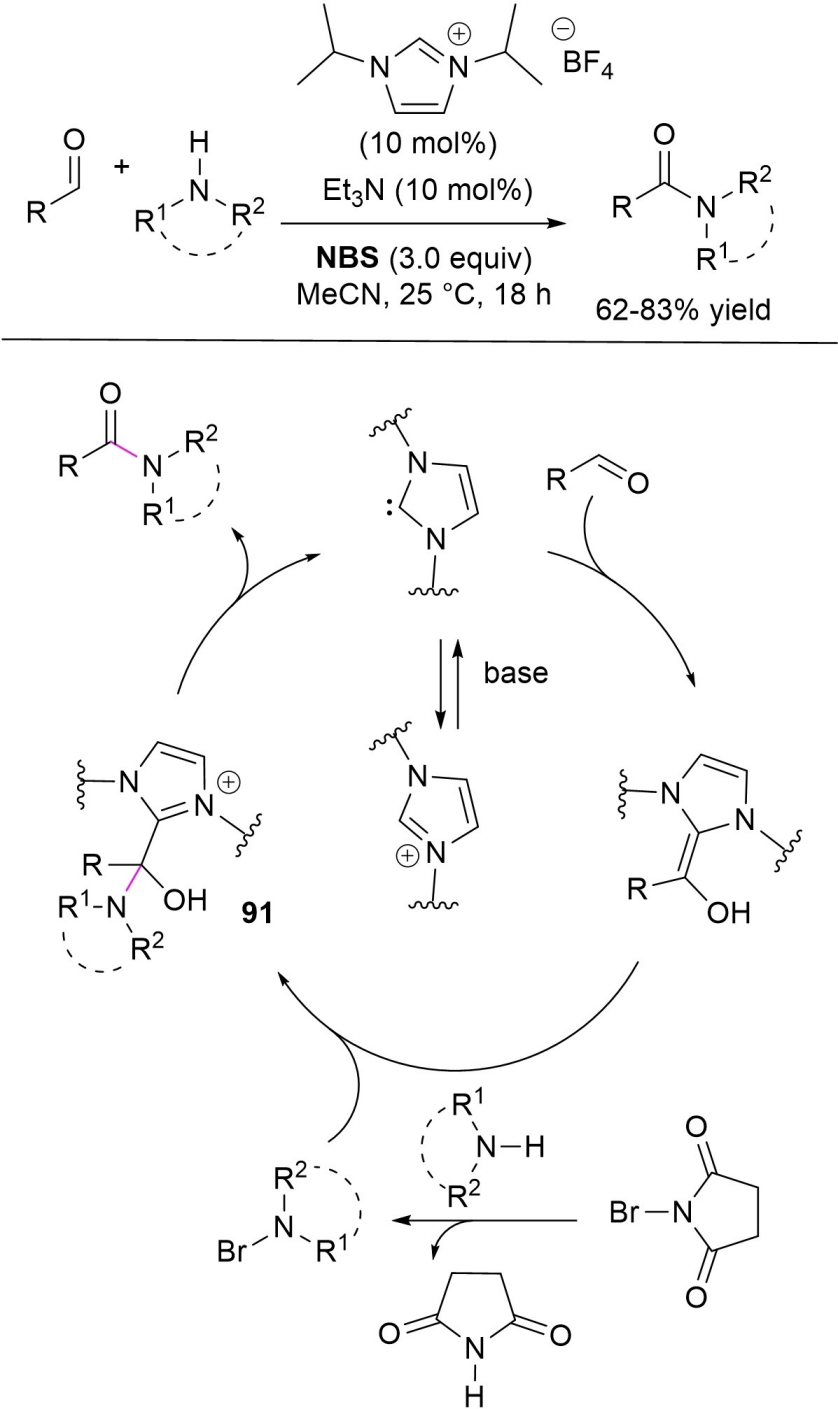
NHC‐catalyzed oxidative amidation of aldehydes with amines promoted by NBS.

In some cases, acyl/imidoyl azolium species are completely ruled out, and unconventional reaction intermediates/mechanisms have been demonstrated. If one speaks of (stoichiometric) external oxidants, it has been shown that the NHC‐catalyzed oxidative amidation of aldehydes with amines may occur with NBS (Scheme 84).^[123]^

A very probable mechanism for the NBS‐promoted oxidative amidation reaction includes the formation of Breslow intermediate and its subsequent reaction with transient *N*‐bromoamine, formed in situ from the amine and NBS. The aminal intermediate **91** is further oxidized to amide, and free NHC is regenerated. Aliphatic primary/cyclic secondary amines and (hetero)aromatic aldehydes were well suited for this type of transformation, with 1,3‐diisopropylimidazolium tetrafluoroborate (10 mol%) as the pre‐catalyst and triethylamine (10 mol%) as the base. Moreover, chiral amino acid (L‐valine, L‐leucine, L‐alanine) methyl esters have been used with success in reactions with benzaldehyde, with the corresponding amides obtained in good yields (68–78 %) and enantioselectivity (>98 % ee).

**Scheme 85:**
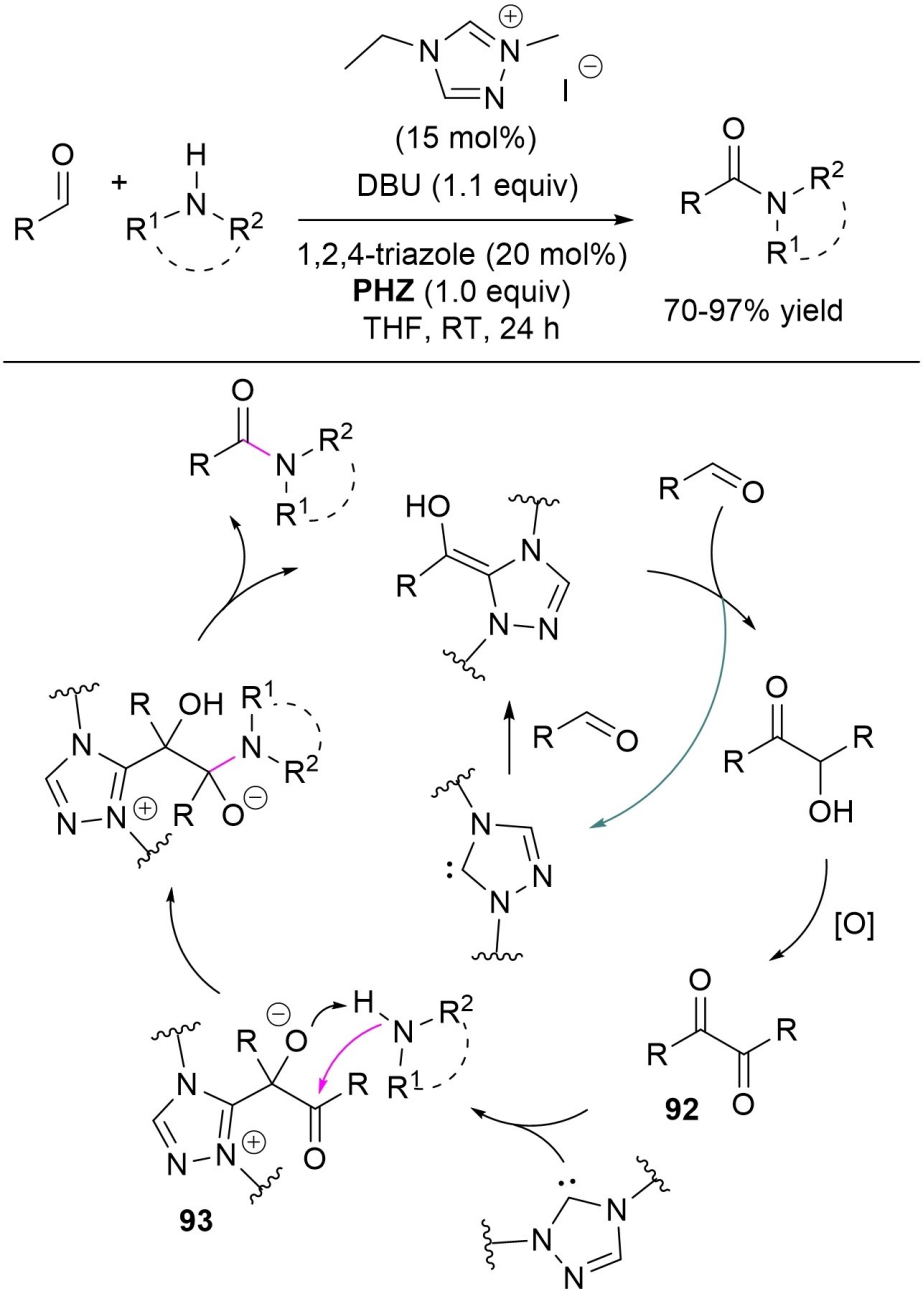
NHC‐catalyzed oxidative amidation of aldehydes with amines promoted by **PHZ**.

Untypical NHC‐catalyzed oxidative amidation of aldehydes with amines which does not involve an acyl azolium ion has been reported by Connon and Kumar.^[124]^ In this case, the aldehyde‐derived Breslow intermediate is turned into a benzil compound **92**, that represents the real acylating agent: it is attacked by the NHC catalyst, the adduct **93** that results is intercepted by the amine nucleophile, and ultimately the corresponding tetrahedral intermediate collapses to the amide product whilst liberating the Breslow intermediate (Scheme 85). This unconventional mechanism was hypothesised for the reactions of benzaldehydes (and heterocyclic variants) and primary/secondary amines with *N*‐ethyl‐*N*‐methyl triazolium iodide (15 mol%), DBU (1.1 equiv.), 1,2,4‐triazole co‐catalyst (20 mol%), and **PHZ** (1.0 equiv.), leading to amides in good to excellent yields (70–97 %). It certainly needs to be put in evidence that **PHZ** was more efficacious than the traditionally used **DQ**, and could be smoothly recycled by exposure of its reduced form (dihydrophenazine) to air.

The synthetic value of this methodology is just as interesting: the MAO‐inhibitor moclobemide could be prepared (92 % yield) by reaction of 4‐chlorobenzaldehyde and 2‐morpholinoethan‐1‐amine.

**Scheme 86:**
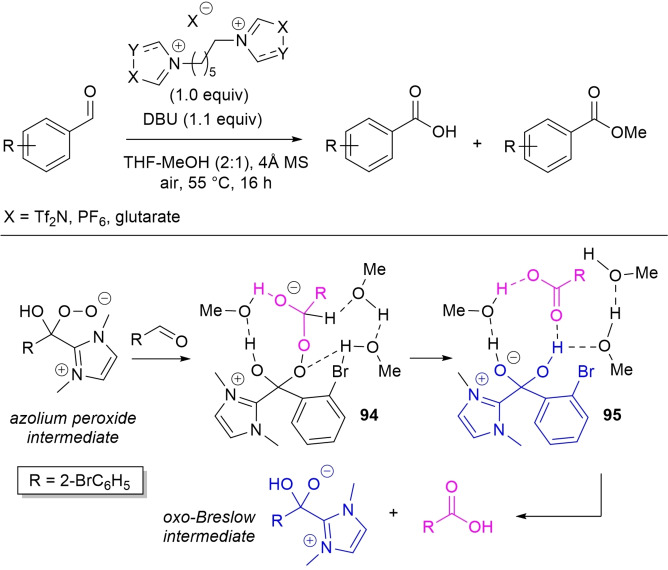
Mechanistic studies on NHC‐catalyzed aerobic oxidation‐esterification of substituted benzaldehydes.

Regarding instead oxygen (air) as the oxidant, the oxidation of Breslow intermediate along oxygenative and oxidative routes was studied in detail by Bortolini, Massi and co‐workers through the support of mass spectrometric (ESI/MS) analysis and gas‐phase/computational studies.[^6^, ^7^] For the purpose of accurate identification of the crucial intermediates already detailed in Scheme 1, bis‐diazolium salts with variable counterions (i. e., Tf_2_N^−^, PF_6_^−^, glutarate) were prepared and effectively used thanks to their dual role, that is NHC precursors and charge‐tags. These species were involved in aerobic oxidation‐esterification of substituted benzaldehydes (Scheme 86), giving results that depended upon the stereoelectronic features of the substrates: *meta*‐ and *para*‐substituted aldehydes were better substrates for esterification (dominant acyl azolium intermediate detection), while the oxygenative path was preferred for the *ortho*‐substituted ones (oxo‐Breslow preferred formation). Taking the reaction of 2‐bromobenzaldehyde as the benchmark (*N*,*N*‐dimethyl azolium pre‐catalyst), it has been calculated that a decisive role is exerted by the solvent/nucleophile (MeOH) molecules, as they stabilize (H‐bond interactions) the adduct deriving from nucleophilic attack of the azolium peroxide intermediate to a second aldehyde molecule (Scheme 86, species **94**). The following O−O bond cleavage takes place synchronously to a three H‐atoms (chained) transfer, the neighboring bromine atom seeming to have a stabilizing effect (Scheme 86, structure **95**): H‐bonded *o*‐bromobenzoic acid and *gem*‐diol (oxo‐Breslow) intermediate are given, with the latter expected to release the second carboxylic acid molecule and the NHC catalyst.

**Scheme 87:**
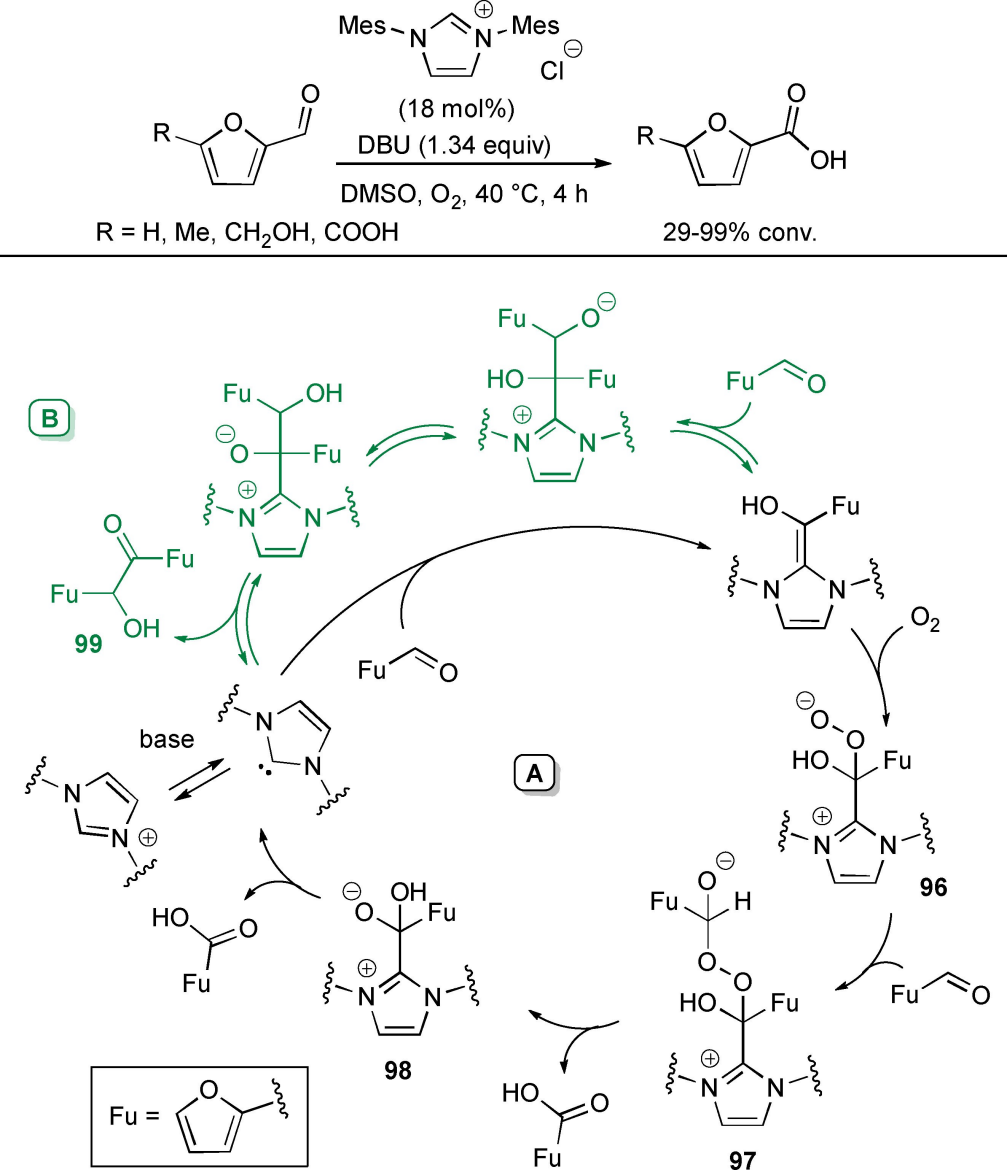
NHC‐catalyzed aerobic oxidation of furan aldehydes to furoic acids.

Biomass‐derived FF was selectively oxidized to furoic acid (>99 % conv., >99 % yield) by the NHC formed from 1,3‐dimesitylimidazolium chloride (18 mol%) and DBU (1.34 equiv.), with O_2_ as the oxidant, and just as efficient transformations (29‐99 % conv.) were demonstrated for furan‐based aldehydes, i. e. 5‐methyl furfural, 5‐formyl‐2‐furancarboxylic acid (FFCA), and HMF (Scheme 87).^[125]^

Detailed studies aimed at elucidating the mechanism of furoic acid formation showed that the typical oxygenative pathway likely operates, with complex **96** between Breslow intermediate and molecular O_2_ formed. A further furfural molecule reacts with **96** to give life to two units of furan‐2‐carboxylic acid via intermediates **97** and **98** (Scheme 87A, black route). However, a competitive road going through dimerization of Breslow intermediate with one furfural molecule to the furoin by‐product **99** shall not be excluded (Scheme 87B, green route). Considering that this last process is reversible,^[126]^ **99** may decompose into the Breslow intermediate and furan aldehyde that fall within the organocatalytic oxygenative cycle leading to the furoic acid.

It is noticeable here that the NHC system used for furoic acid synthesis proved superior to well‐established (supported) Au^[127]^ and Pb^[128]^ catalysts for aerobic oxidation of furfural, and on top of that direct formation of furoic acid (57 % yield) has been possible by a sequential strategy combining the dehydration of xylose to furfural (Amberlyst‐70 catalyst, 2 h) and NHC‐catalyzed aerobic oxidation (2 h) of the unpurified reaction mixture (after acid catalyst filtration).

**Scheme 88:**
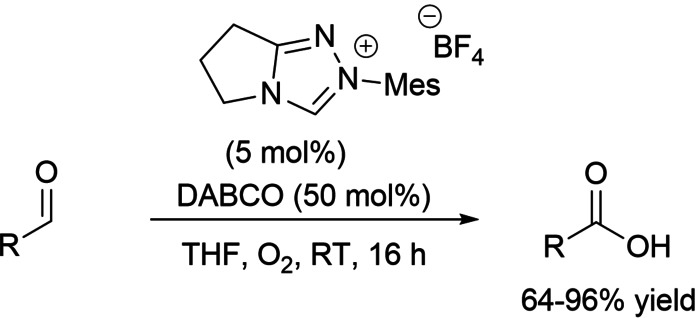
NHC‐catalyzed aerobic oxidation of aldehydes to carboxylic acids.

Looking more broadly towards the NHC‐catalyzed aerobic oxidation of aldehydes to carboxylic acids, one could mention the reactions of electron‐rich/electron‐deficient benzaldehydes, 1‐naphthaldehyde, anthracene‐9‐carboxaldehyde, heteroaryl aldehydes, and (α‐substituted) enals with oxygen gas catalyzed by pyrrolidine‐based *N*‐mesityl triazolium salt (5 mol%)/DABCO (50 mol%) system (Scheme 88), which gave products in fair to excellent yields (64‐96 %).^[129]^

Furthermore, aldehyde‐to‐ester conversion has been made possible by the use of tetraphenylphosphonium bromide (Ph_4_PBr) and arylboronic acids as unconventional electrophilic reagents for the azolium peroxidic intermediate that derives from Breslow species.

**Scheme 89:**
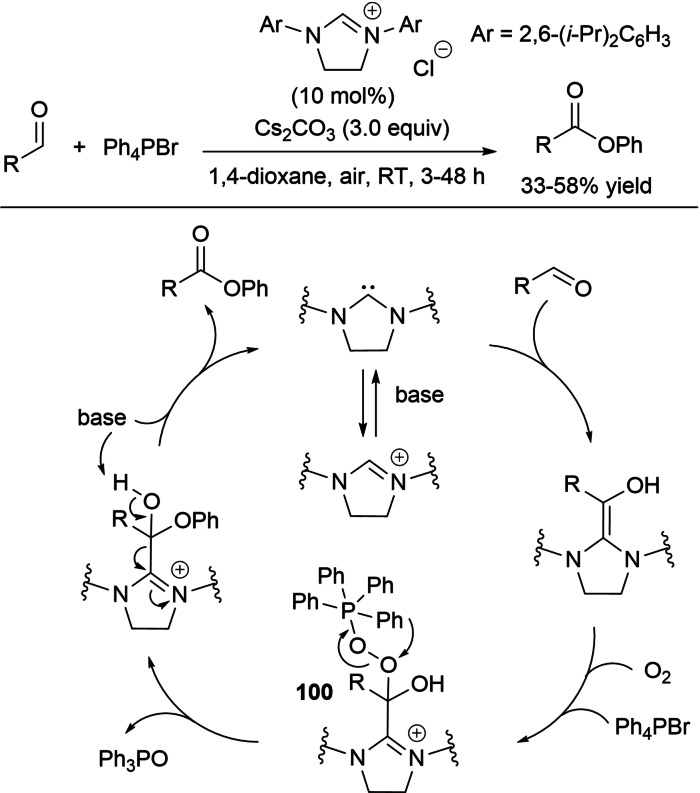
NHC‐catalyzed aerobic oxidative esterification of aldehydes with Ph_4_PBr.

In the first case, the catalyst formed from 1,3‐bis(2,6‐diisopropylphenyl)‐4,5‐dihydroimidazolium chloride (10 mol%) and Cs_2_CO_3_ (3.0 equiv.) has promoted transformations of (hetero)aromatic aldehydes into phenyl esters, albeit in moderate yields (Scheme 89).^[130]^ Concerted reaction of the critical Breslow intermediate with O_2_ and Ph_4_PBr engenders the cationic compound **100** which quickly breaks down by expelling triphenylphosphine oxide (Ph_3_PO). A following deprotonation closes the organocatalytic cycle, with the ester product released along with free NHC.

**Scheme 90:**
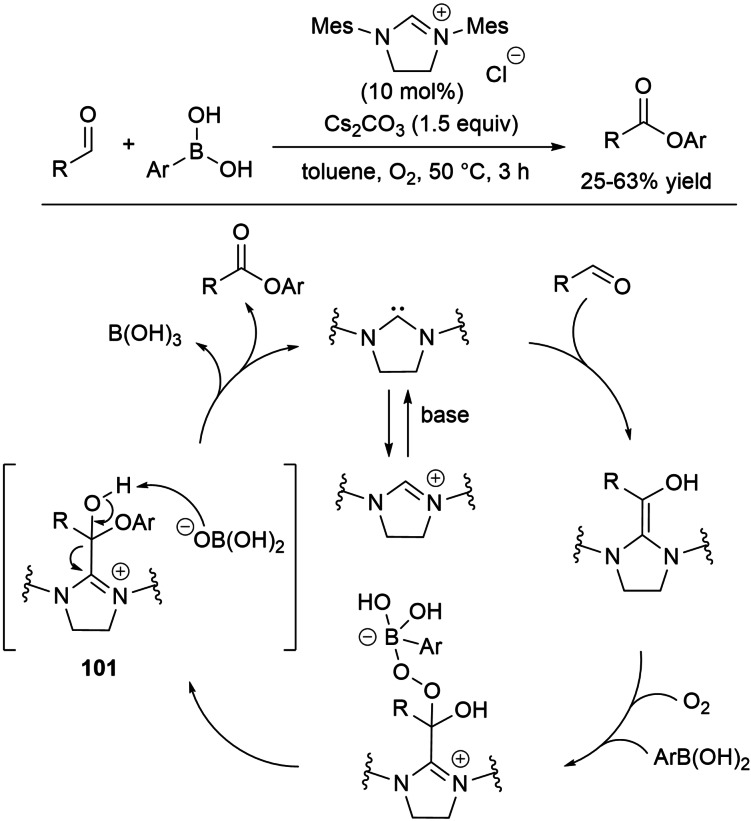
NHC‐catalyzed aerobic oxidative esterification of aldehydes with arylboronic acids.

A mirror path has been hypothesised for the reactions of 4‐tolyl‐ or naphthalen‐1‐yl boronic acid with (hetero)aromatic, ferrocenyl, and α,β‐unsaturated aldehydes under catalysis of the NHC generated from 1,3‐dimesityl‐4,5‐dihydroimidazolium chloride (10 mol%) and Cs_2_CO_3_ (1.5 equiv.) (Scheme 90).^[131]^

Intermediate **101** is supposed to be formed by decomposition of the adduct arising from the Breslow species, O_2_, and aryl boronic acid, then a final proton‐transfer step liberates the expected ester, boron trihydroxide by‐product and the catalyst.[^132^, ^133^]

**Scheme 91:**
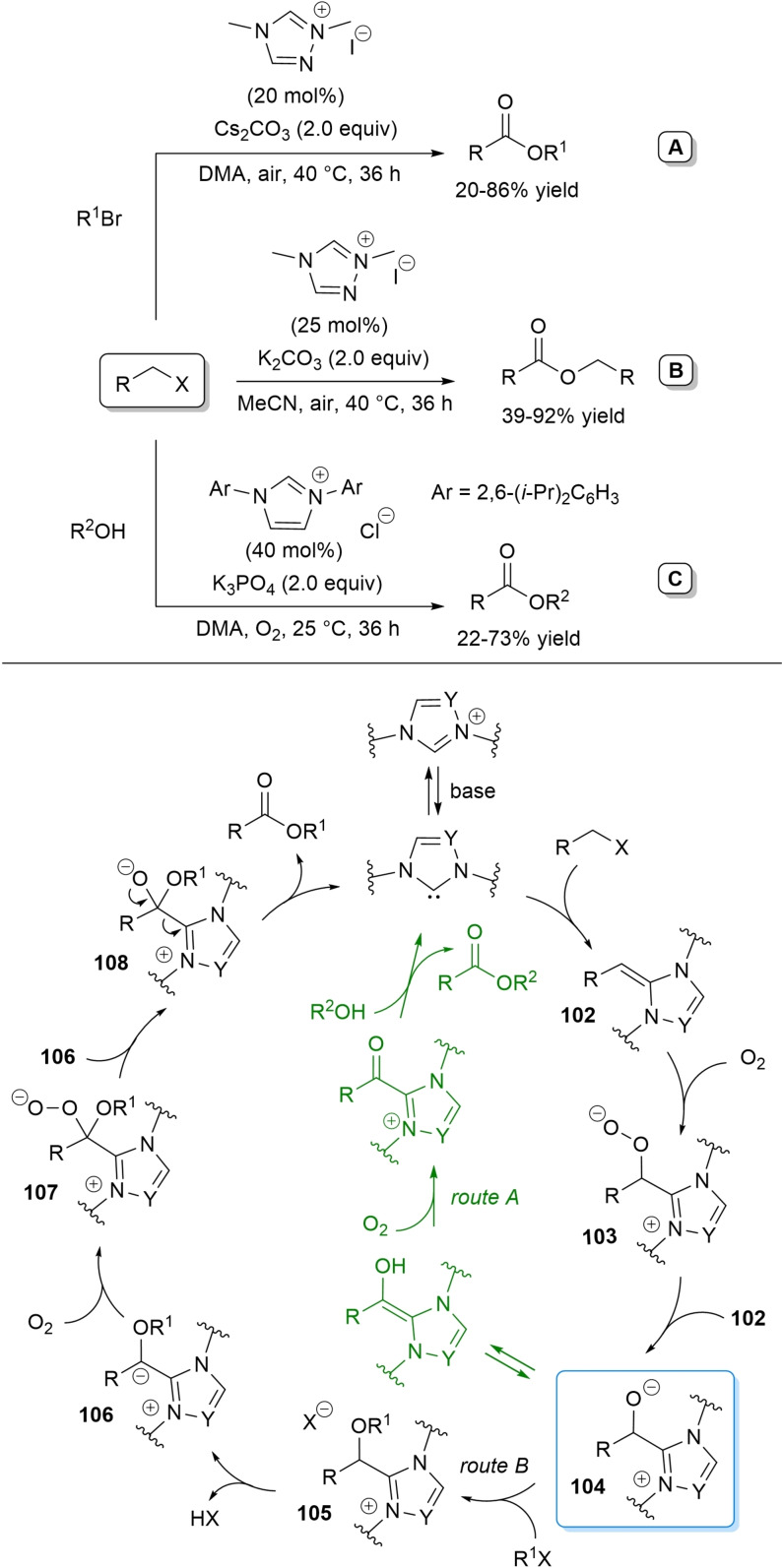
NHC‐catalyzed aerobic oxidative cross/self‐esterification of organic halides.

A novel NHC‐catalyzed oxidative synthesis of esters from benzylic/non‐benzylic halides has been recently devised by the group of Jiao.^[134]^ Efficient protocols under different catalytic conditions were found for cross‐esterification with alkyl bromides (Scheme 91A, 20–86 % yield), self‐esterification (Scheme 91B, 39–92 % yield), and cross‐esterification with aliphatic alcohols (Scheme 91C, 22–73 % yield).

Very detailed studies made clear that *i)* both O‐atoms of the ester products originate from dioxygen and *ii)* deoxy Breslow intermediate **102** is involved as the central species in these transformations. Its oxygenation with O_2_ and reaction with the ensuing peroxide anion **103** generate zwitterion **104**, which may take one of two different directions. On the one side, it turns into Breslow intermediate which gets involved in a classical oxygenative process leading to the ester product via acyl azolium/alcohol reaction (Scheme 91, green route). On the other, **104** is intercepted by the alkyl halide to form azolium ether **105**, with subsequent deprotonation to **106** and oxygenation yielding peroxide anion **107**. Reaction of the latter with **106** affords intermediate **108**, which closes the organocatalytic cycle by expelling the ester compound concomitantly with regeneration of the NHC catalyst (Scheme 91, black route).

The participation of a NHC‐bound peroxide species has been shown to occur also in the case of *aza*‐Breslow intermediates, giving rise to amide compounds.

**Scheme 92:**
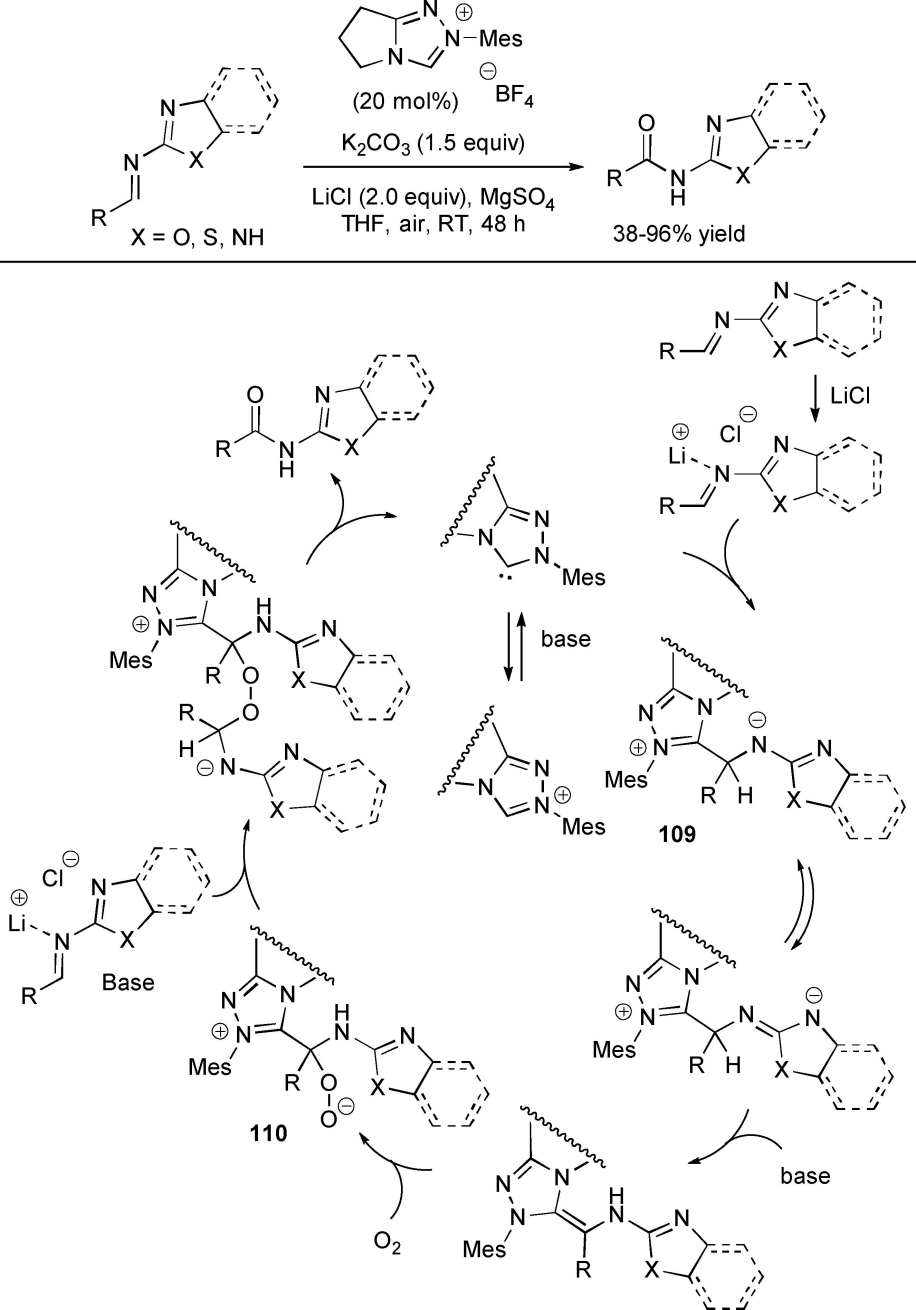
NHC‐catalyzed aerobic oxidative amidation of aldimines.

In 2017, Fu and Huang demonstrated this in their studies on the aerobic oxidative amidation of aldimines through NHC/LiCl cooperative catalysis.^[135]^ As described in Scheme 92, a series of imines arising from (hetero)aryl/α,β‐unsaturated aldehydes and heteroaryl amines (i. e., 2‐aminobenzothiazole, 2‐aminobenzoxazole, 2‐aminobenzimidazole, 2‐aminothiazole) have undergone treatment with achiral *N*‐mesityl pyrrolidine‐based NHC pre‐catalyst (20 mol%), K_2_CO_3_ (1.5 equiv.), and LiCl additive (2.0 equiv.) under air, and corresponding amide products were derived in moderate to excellent yields (38–96 %).

What is of interest here is that convincing outcomes (70–88 % yield) have been realized by a one‐pot approach directly starting from aldehyde and amine reagents (1 : 1 molar ratio), with the aldimines formed in situ used at once without being isolated (purified).

Background experiments have brought the authors to establish a mechanistic rationale for the aerobic oxidative amidation of imines, wherein the aldimine/NHC addition adduct **109** is formed, as confirmed by X‐ray diffraction analysis of the intermediate obtained from benzaldehyde‐derived benzothiazole‐based imine. Dearomatization of **109** and subsequent proton transfer lead to the *aza*‐Breslow intermediate, which adds to dioxygen forming the zwitterionic intermediate **110**. Its reaction with a second imine molecule and O−O bond cleavage release two amide units and the NHC promoter.

**Scheme 93:**
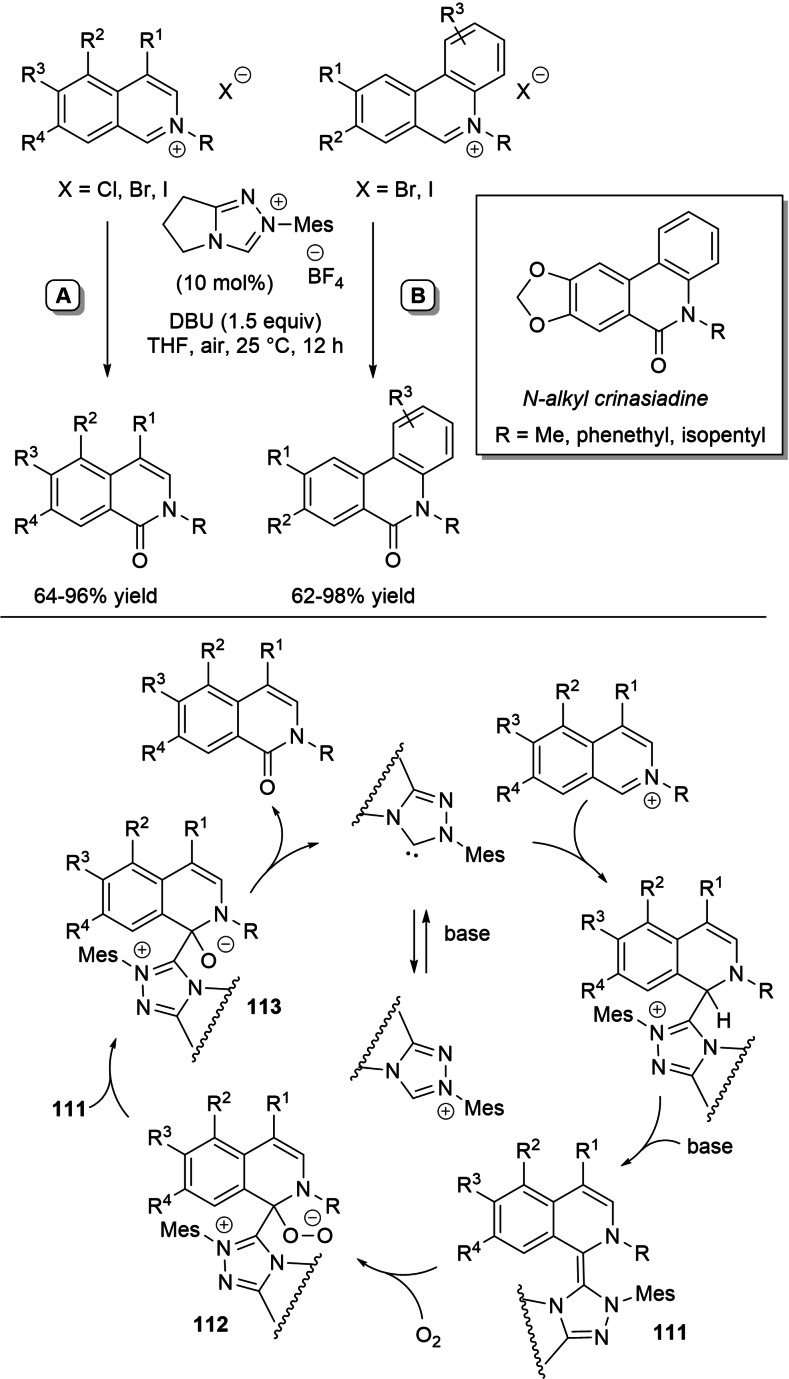
NHC‐catalyzed aerobic oxidation of isoquinolinium/phenanthridinium salts.

One year later, the same authors have capitalized the reaction between an *aza*‐Breslow intermediate and dioxygen for the synthesis of isoquinolinones and phenanthridinones, respectively from isoquinolinium and phenanthridinium salts.^[136]^ Getting into specifics, a great assortment of 4‐, 5‐, 6‐, and 7‐substituted 2‐alkyl isoquinolinium halide salts were tested in aerobic reactions with the same triazolium pre‐catalyst used for imines oxidative amidation (10 mol%), using DBU as the base (1.5 equiv.) (Scheme 93A). Good to excellent yields (64‐96 %) of isoquinolinone products were gained, and exactly alike worked a gram‐scale reaction of 2‐methylisoquinolinium iodide (20 mmol, 1 mol% catalyst, 99 % yield).

The same catalytic system was also suitable for transformations of phenanthridinium bromide/iodide salts (62–98 % yield), with the three natural products *N*‐methyl, *N*‐phenethyl, and *N*‐isopentyl crinasiadine effectively constructed by this strategy (Scheme 93B).

Proved by control experiments, the reaction pathway is assumed to start with NHC addition to the cyclic iminium salt, then deprotonation and O_2_ addition to the formed *aza*‐Breslow intermediate **111** go to azolium peroxidic species **112**. The latter is supposed to couple with a second *aza*‐Breslow molecule generating anion **113** from which derives the final amide.

**Scheme 94:**
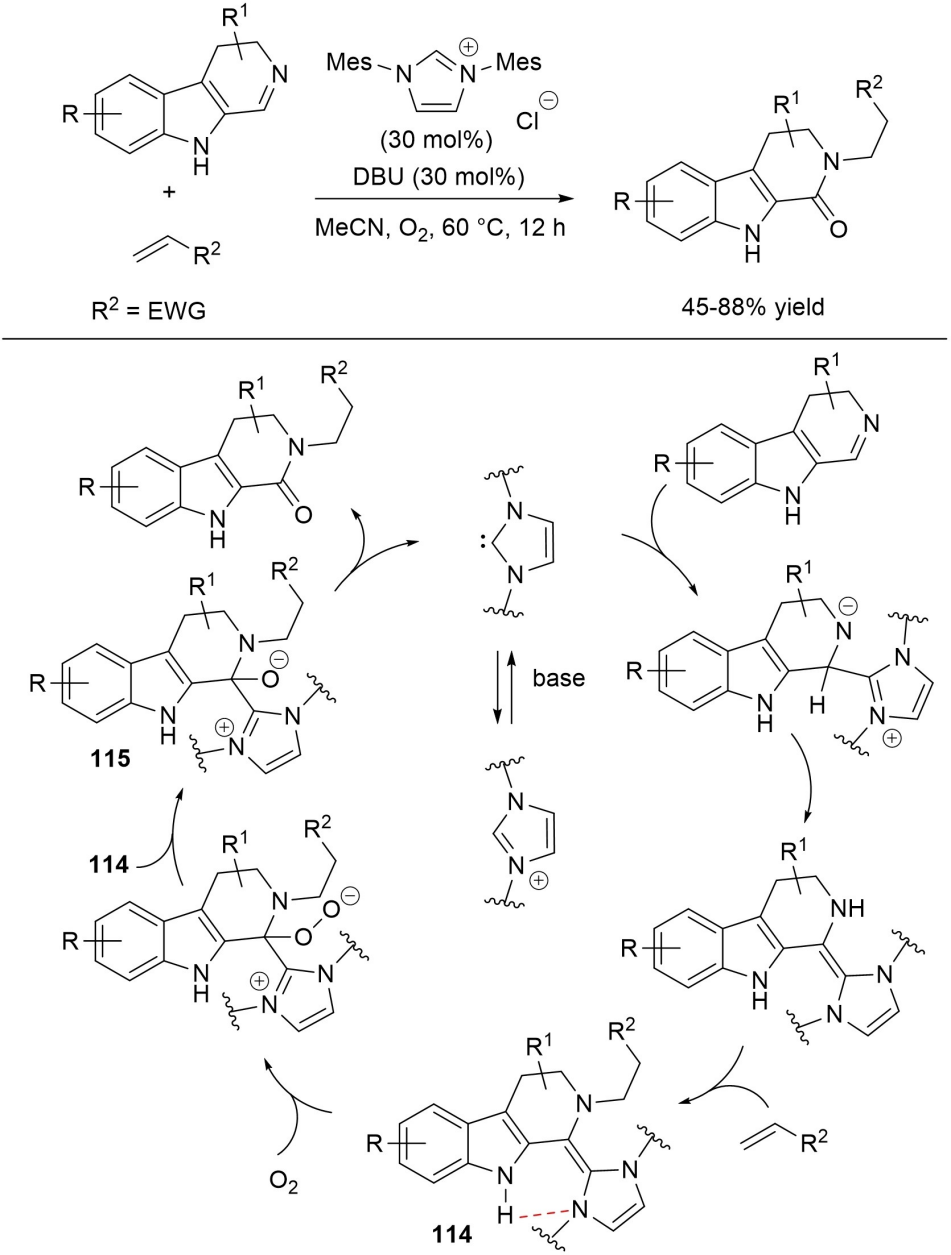
NHC‐catalyzed aerobic oxidation of β‐carboline‐based cyclic imines.

Recently, conversion of β‐carboline‐based cyclic imines to *N*‐substituted cyclic amides has been described through an *aza*‐Breslow intermediate amenable to *aza*‐Michael addition and oxidation with molecular oxygen.^[137]^ 1,3‐Dimesityl imidazolium chloride (30 mol%), DBU (30 mol%), and O_2_ represented the optimized set for making react different Michael acceptors (acrylates, acryl amides, vinyl ketones, acrylonitrile, phenyl vinyl sulfone) with dihydro‐β‐carboline imines having substituents on the dihydropyridine or indole ring, or both (Scheme 94), supplying dihydro‐β‐carboline‐1‐ones in moderate to good yields (45–88 %).

A reasonable reaction scheme expects that the initially formed *aza*‐Breslow intermediate undergoes *aza*‐Michael addition to deliver *N*‐protected *aza*‐Breslow species **114**, likely stabilized by intramolecular hydrogen bonding interactions involving the free NH indole group.^[138]^ At this moment, sequential intervention of O_2_ and a second molecule of **114** drive to zwitterion **115**, precursor of the final amide.

**Scheme 95:**
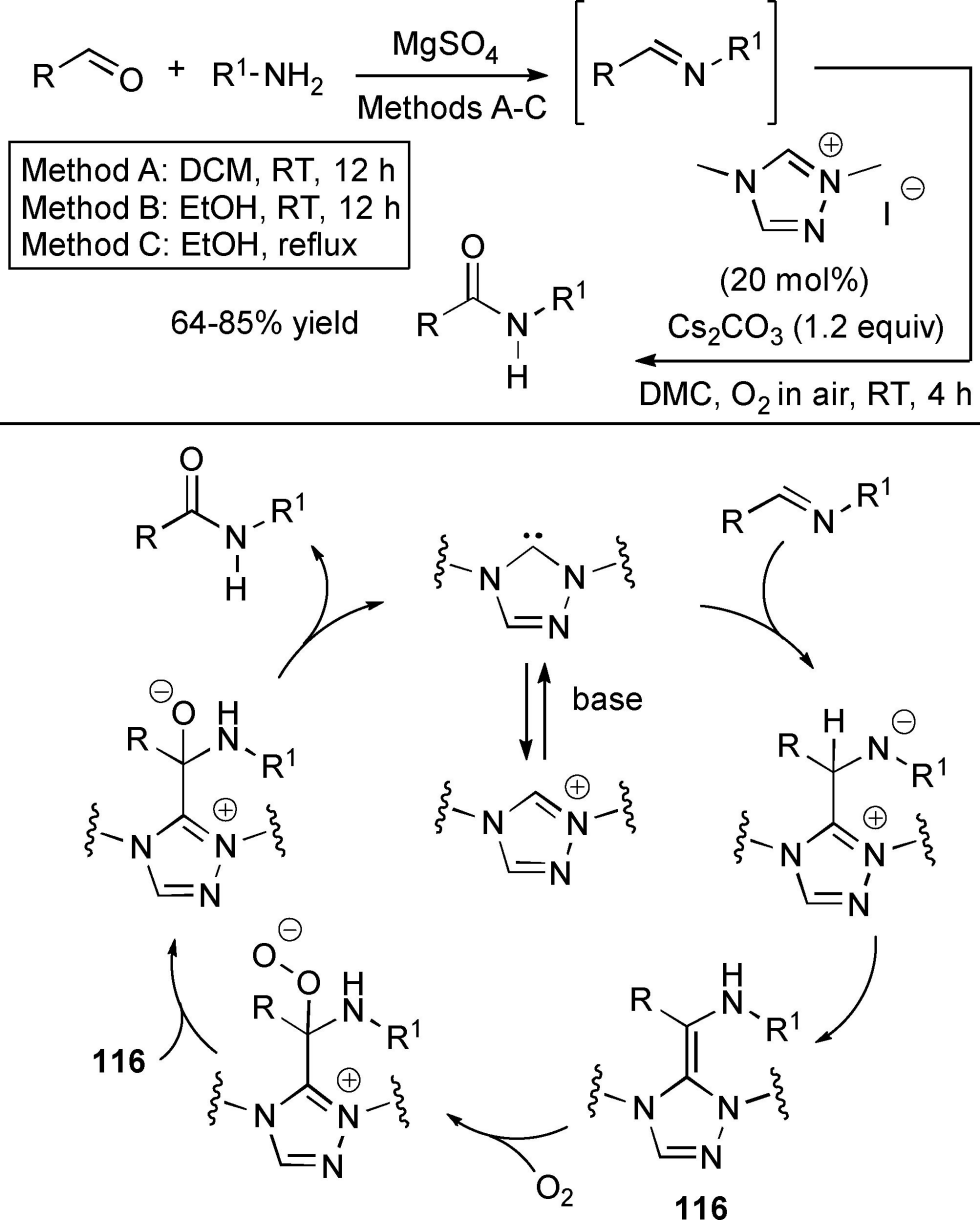
Eco‐friendly NHC‐catalyzed aerobic oxidation of unactivated imines.

The synthesis of amides through NHC‐catalyzed aerobic oxidation of unactivated imines via their *umpolung* (*aza*‐Breslow intermediate) has been improved further by an environmentally friendly method using DMC as a green solvent.^[139]^ Hence, the crude imines prepared from (hetero)aromatic/vinyl aldehydes and (hetero)aromatic amines have been turned into the respective amides in 64–85 % yield when exposed to dimethyl triazolium iodide (20 mol%), Cs_2_CO_3_ (1.2 equiv.) in the presence of air (Scheme 95), and 10 mmol‐scale reactions under identical conditions have been easily achievable (66–70 % yield).

The well‐established mechanism which goes through formation of an azolium peroxidic species (from *aza*‐Breslow intermediate **116**) and its reduced form has been presented.

**Scheme 96:**
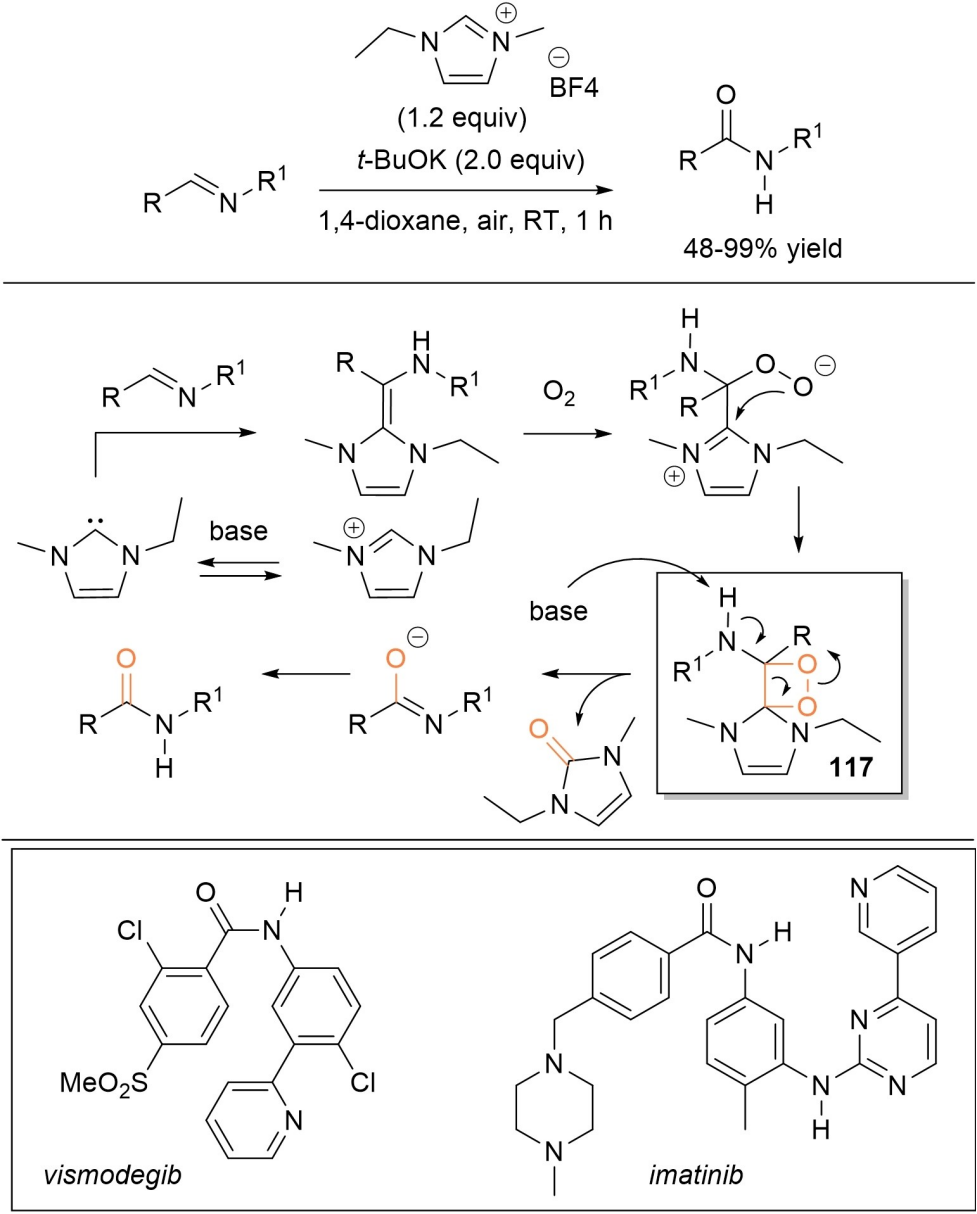
NHC‐catalyzed aerobic oxidation of imines to amides via NHC‐bound oxetane intermediate.

A lot recently, Wang and co‐workers suggested that imine‐to‐amide oxidation under aerobic NHC‐catalysis might proceed through an unusual NHC‐bound 1,2‐dioxetane species **117**: this likely arises from intramolecular cyclization of the *aza*‐Breslow‐derived azolium peroxidic intermediate (Scheme 96),^[140]^ similar to what happens with the process behind firefly luciferase bioluminescence.^[141]^ Eventually, collapse of **117** is accompanied by formation of a 1,3‐dihydro‐2*H*‐imidazol‐2‐one by‐product which originates from the NHC portion.

The mechanism shown in Scheme 96 was put forward for the reactions of imines with imidazolium pre‐catalyst (1.2 equiv.) and *t*‐BuOK (2.0 equiv.) under air, with option of using very variable substitution patterns on acyclic imines, including *N*‐protection with Ts‐ and Boc‐groups, but also diimines and cyclic imines.

It should be emphasized that a one‐pot oxidation protocol starting from imines generated in situ from aldehydes and amines (equimolar ratio) has resulted in similar outcomes to those arising from pre‐formed imines (91–93 % yield of amides), with the anticancer drugs vismodegib (96 % yield) and imatinib (86 % yield) effectually obtained by this ploy.

## 6 Conclusions

This work unequivocally demonstrates the crucial synthetic value of oxidative NHC‐catalysis as an important part of the wider picture of NHC‐catalysis. At its core is the non‐*umpolung* tactics involving various NHC‐bound azolium intermediates that comply with electrophilic and/or nucleophilic features, where appropriate, opening avenues for construction of a wide variety of valuable derivatives through diverse reactivity patterns (e. g., ordinary nucleophilic additions, domino reactions, annulation reactions). For the most part, organic oxidants, and in particular the Kharasch reagent, are still the protagonists, but the need for greater sustainability is increasingly felt. This translates both in the using of oxygen (air) as the terminal oxidant or in the experimenting with bio‐based (renewable) chemicals as the starting materials and/or green solvents. And in this view, also very remarkable are the increasing efforts to implement oxidative NHC‐catalysis with protocols under heterogeneous catalytic conditions, both in batch and continuous‐flow regime, also with a view to process intensification. With such premises, it is believed that oxidative NHC‐catalysis is destined to be enriched with progressively more innovative contributions in the near future and beyond.

## Conflict of interest

The authors declare no conflict of interest.

7

## Biographical Information

*Carmela De Risi graduated in Chemistry (1992) at the University of Ferrara (Italy) and received her PhD in Organic Chemistry in 1996. That same year, she joined the group of Prof. P. Vogel at the University of Lausanne (Switzerland) where she spent one year research as a grant holder. Then, she moved back to the University of Ferrara where she performed postdoctoral studies (1997–1999). Since November 1999 she has been Research Associate at the University of Ferrara. Her main research interests focus on synthesis and modification of biologically active compounds, general synthetic methodologies, organocatalysis, biomass valorization*.

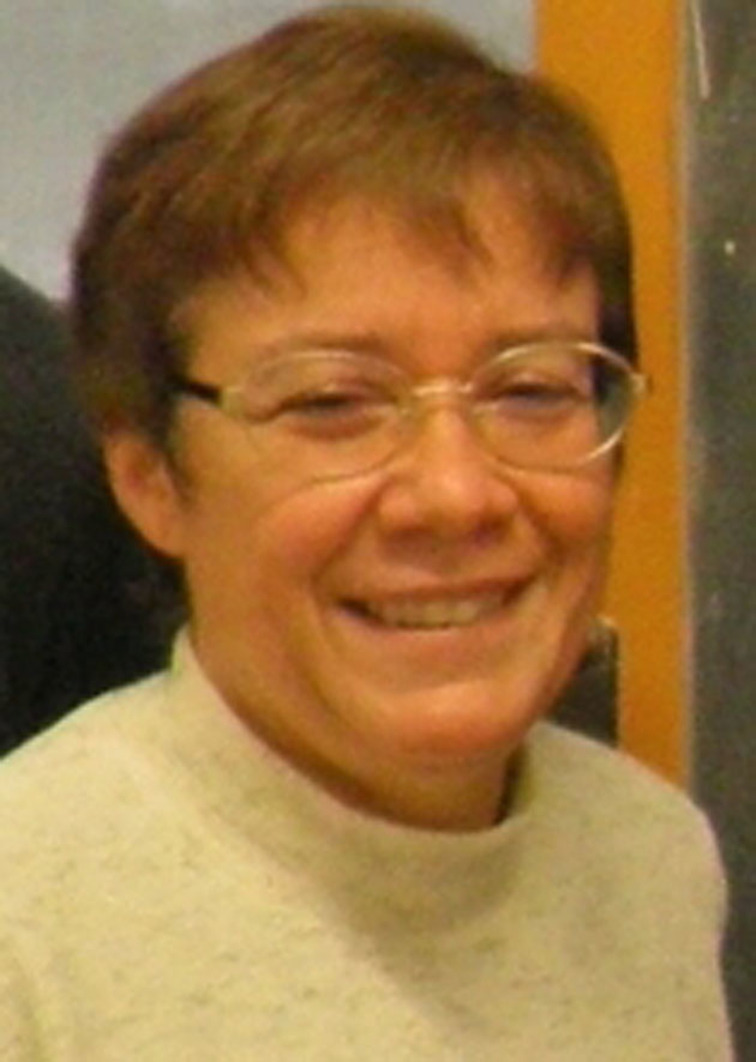

## Biographical Information

*Arianna Brandolese obtained her PhD in 2021 at the University of Ferrara (Italy) working in the group of Prof. Olga Bortolini on processes promoted by homogeneous and heterogeneous organocatalysts. In 2020, she pursued her studies at the University of St. Andrews (UK) working in the group of Prof. Andrew Smith. Later in 2021, she joined the research team of Prof. Arjan Kleij at ICIQ (Tarragona, Spain) where she worked on the development of new biobased polymers. Since July 2022 she has been working at the University of Ferrara, focusing on biomass valorisation and on the preparation of new bio‐based polymers*.

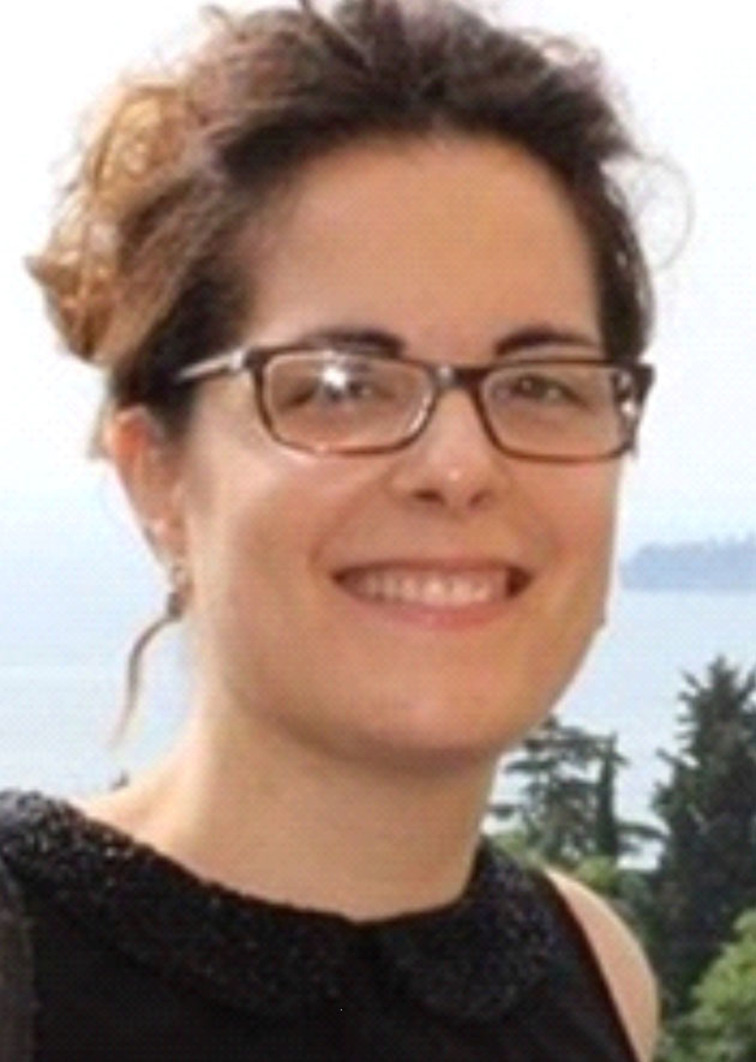

## Biographical Information

*Graziano Di Carmine obtained his PhD in Chemistry in 2019 at the University of Ferrara (Italy) under the supervision of Prof. Olga Bortolini, working on umpolung reactivity promoted by organocatalysts and enzymes. He was research fellow from 2018 to 2019 at ISOF CNR of Bologna and then he moved to Manchester at the CEAS (Department of Chemical Engineering and Analytical Sciences) as Research Associate in the group of Dr. Carmine D′Agostino, working on NMR methods for the investigation of reactions promoted by solid‐supported organocatalysts. He is currently a Research Fellow at the University of Ferrara*.

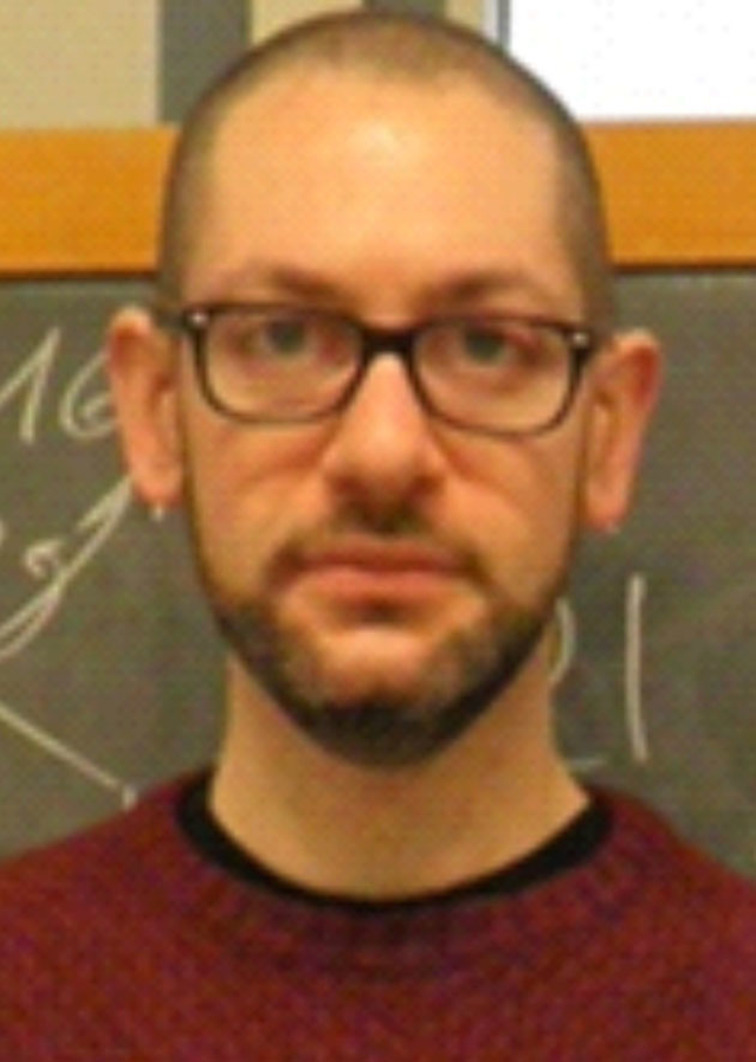

## Biographical Information

*Daniele Ragno received his PhD in Chemistry in 2016 from the University of Ferrara (Italy), under the supervision of Prof. Alessandro Massi. In 2015 he worked in the research group of Prof. Albrecht Berkessel at the Institute of Organic Chemistry of the University of Cologne (Germany) as a visiting PhD student. He was Research Associate from 2018 to 2022 at the Department of Chemical, Pharmaceutical and Agricultural Sciences of the University of Ferrara. Since 2022 he works as Assistant Professor at the same Department. His main research interests include organocatalysis, heterogeneous catalysis, flow chemistry, green chemistry and polymer chemistry*.

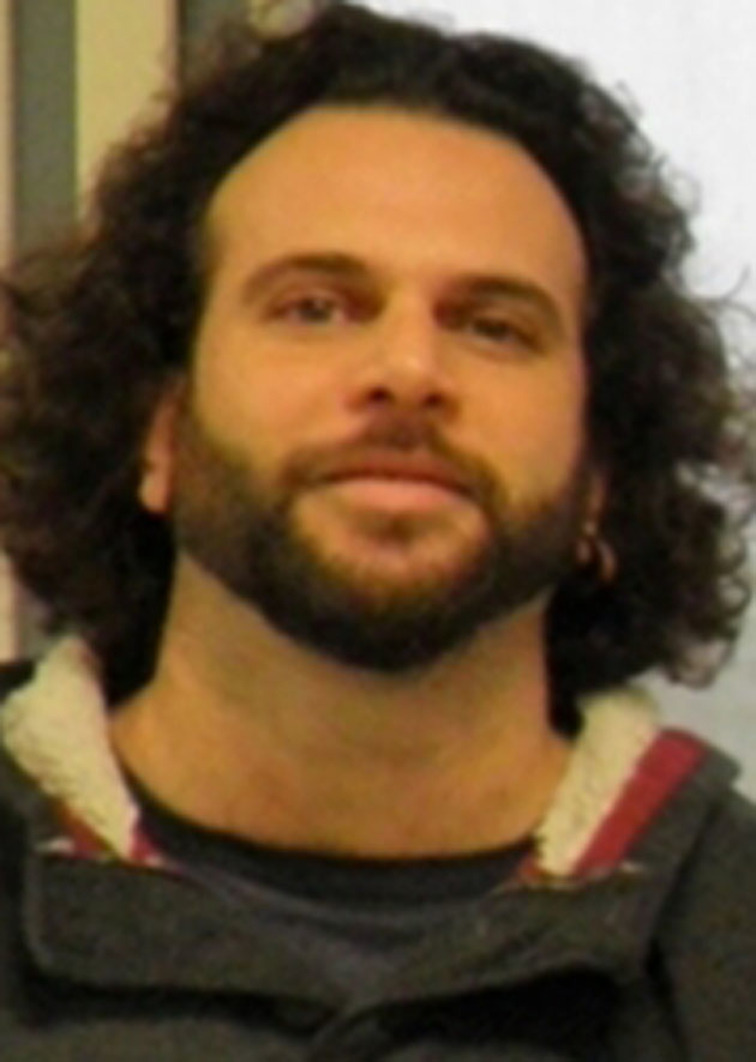

## Biographical Information

*Alessandro Massi received his PhD (1999) from the University of Ferrara (Italy). He then joined the group of Prof. S. V. Ley at the University of Cambridge (UK) as post‐doctoral fellow (1999–2000). Then, he moved back to the University of Ferrara where he currently holds the position of Full Professor of Organic Chemistry at the Department of Chemical, Pharmaceutical and Agricultural Sciences. His recent research interests include heterogeneous catalysis, flow‐chemistry, and valorization of renewable resources*.

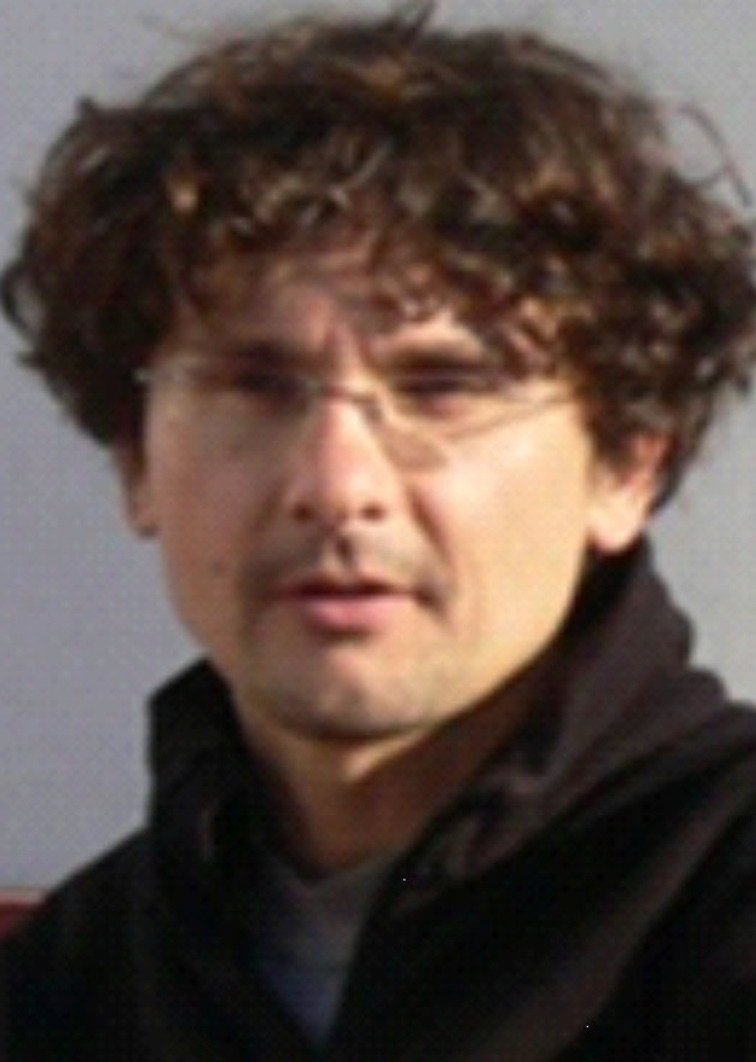

## Biographical Information

*Olga Bortolini received a Laurea degree in Chemistry from the University of Padova (1979). She was CNR research fellow (1983–1987), Associate Professor of Organic Chemistry at the University of Ferrara (1987–2003), Professor of Organic Chemistry at the University of Calabria (2003–2010) and currently at the University of Ferrara. She is presently head of the Department of Environment and Prevention Sciences. Her main research interests include studies of reaction mechanisms in solution (metal‐catalyzed oxidation systems) and in the gas phase (organocatalyzed reactions), ionic liquids, N‐heterocyclic carbenes and bio‐equivalents for new C−C bond formation*.

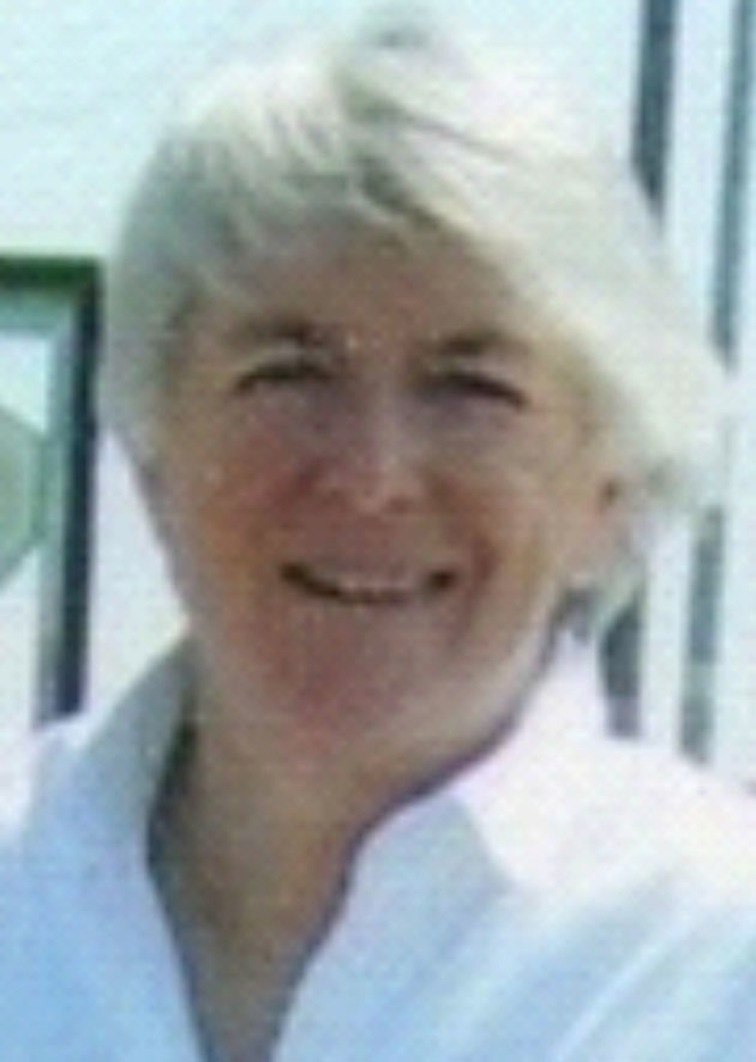

## Data Availability

Data sharing is not applicable to this article as no new data were created or analyzed in this study.
